# On-demand synthesis of phosphoramidites

**DOI:** 10.1038/s41467-021-22945-z

**Published:** 2021-05-12

**Authors:** Alexander F. Sandahl, Thuy J. D. Nguyen, Rikke A. Hansen, Martin B. Johansen, Troels Skrydstrup, Kurt V. Gothelf

**Affiliations:** 1 Interdisciplinary Nanoscience Center, iNANO, Aarhus University, Aarhus C, Denmark; 2 Department of Chemistry, Aarhus University, Aarhus C, Denmark

**Keywords:** DNA, Synthetic chemistry methodology, Polymer synthesis

## Abstract

Automated chemical synthesis of oligonucleotides is of fundamental importance for the production of primers for the polymerase chain reaction (PCR), for oligonucleotide-based drugs, and for numerous other medical and biotechnological applications. The highly optimised automised chemical oligonucleotide synthesis relies upon phosphoramidites as the phosphate precursors and one of the drawbacks of this technology is the poor bench stability of phosphoramidites. Here, we report on the development of an on-demand flow synthesis of phosphoramidites from their corresponding alcohols, which is accomplished with short reaction times, near-quantitative yields and without the need of purification before being submitted directly to automated oligonucleotide synthesis. Sterically hindered as well as redox unstable phosphoramidites are synthesised using this methodology and the subsequent couplings are near-quantitative for all substrates. The vision for this technology is direct integration into DNA synthesisers thereby omitting manual synthesis and storage of phosphoramidites.

## Abstract (web summary)

The poor bench stability of phosphoramidites is a drawback for fast automised chemical oligonucleotide synthesis. Here, the authors report a method for on-demand flow synthesis of phosphoramidites within short reaction times, in near-quantitative yields and sufficient purity for integration with DNA synthesizers.

## Introduction

Synthetic oligonucleotides are essential for a range of different areas and millions of oligonucleotides are synthesized daily for use in research laboratories, hospitals, and industry. The availability of arbitrary oligonucleotide sequences through chemical synthesis is one of the cornerstones of biotechnology and a prerequisite for technologies such as PCR^1^, DNA-sequencing^2^, synthetic biology^3^, and CRISPR-Cas9^4^. Among its many applications PCR is one of the key technologies used for identification of pathogens, such as the current SARS-CoV-2 virus^5^. With the development of chemical modifications of oligonucleotides, the application of antisense oligonucleotide-based strategies for treatment of diseases has become possible^6–9^. Libraries of vast numbers of oligonucleotide aptamers^6^, microarrays of thousands of oligonucleotides on surfaces^7^, and building block sequences for DNA nanostructures are also prepared by chemical synthesis^10–13^. Common for all the oligonucleotides described above is that they are synthesized from nucleoside phosphoramidite building blocks.

**Fig. 1:**
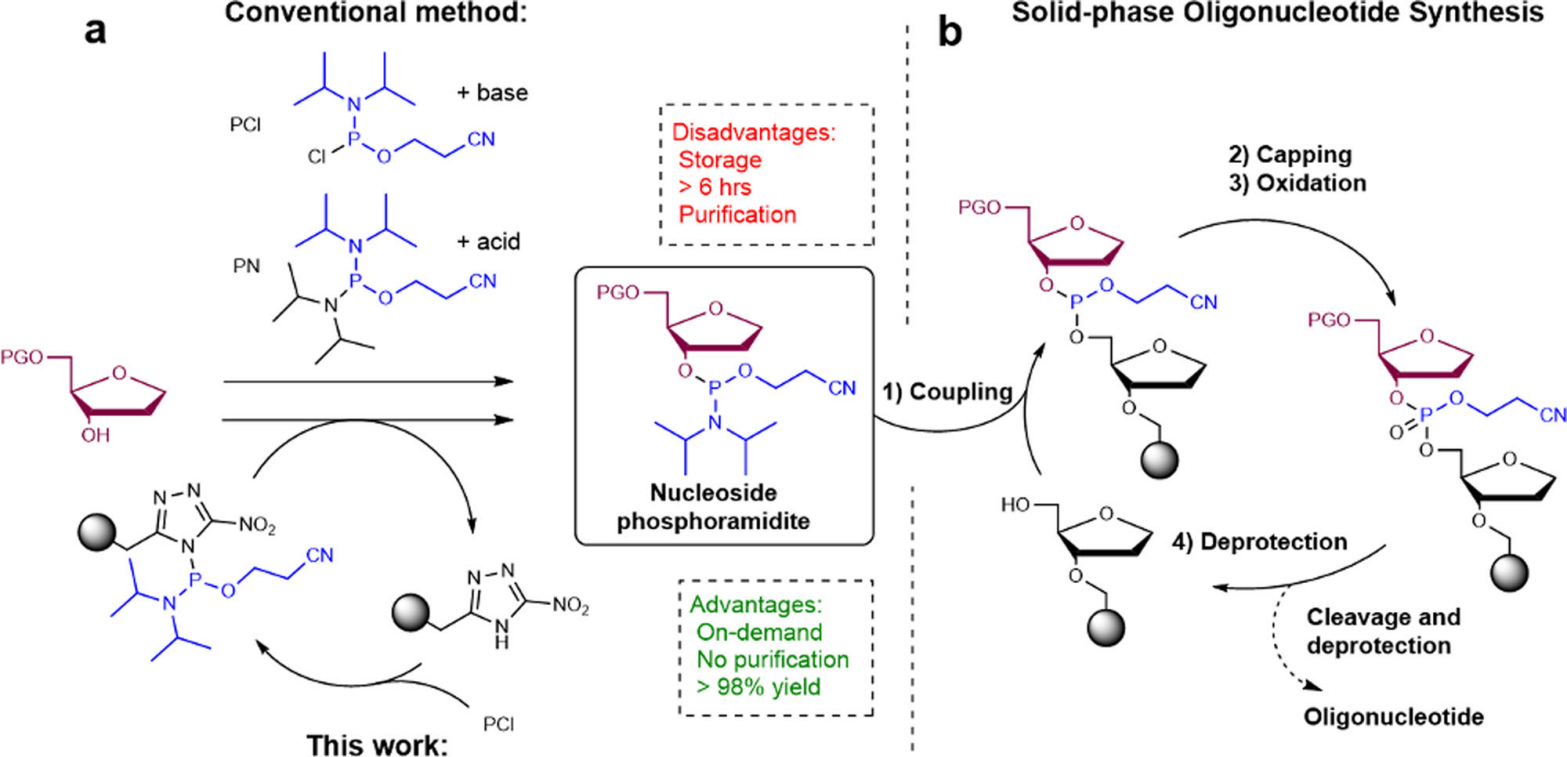
Strategy for phosphoramidite synthesis in a flow-based setup. **a** Binding of P(III)-species to the resin followed by transfer to a desired alcohol followed by, **b** direct submission to solid-phase oligonucleotide synthesis. PG protecting group.

Since the introduction of phosphoramidite chemistry for DNA synthesis by Caruthers in 1981^8^, it has become the golden standard for synthesis of oligonucleotides. The key step in this synthetic approach for DNA synthesis is the reaction of the nucleoside phosphoramidite building block, with the terminal 5′-OH of the oligonucleotide (Fig. 1b). Through further development^9,14^, automated oligonucleotide synthesis on solid support has evolved to the classical 4-step synthesis cycle shown in Fig. 1b. With the commercialization of automated DNA synthesizers, chemical oligonucleotide synthesis has become a commodity for many research institutes, and several companies have specialized in custom oligonucleotide synthesis. The catalog of commercially available nucleoside and non-nucleoside phosphoramidite building blocks ranging from fluorophores and other dyes to protein ligands and redox tags (https://www.glenresearch.com/site-content) has expanded to several hundred over the past decades. While the conditions and the protecting groups of the building blocks used in the solid phase synthesis cycle have been optimized for reaction times, mildness and yields^10^, the chemical reactions of the original 4-step synthesis cycle have largely remained unchanged. Other recent noteworthy examples include the use of mechanochemistry^11^ or the use of P(V) chemistry to form the internucleosidic P–O bond^12,13^.

The crucial phosphoramidite building blocks are still commonly prepared according to the protocols published by Caruthers in 1981^8^ by use of the chlorophosphoramidite reagent: 2-cyanoethyl diisopropylchlorophosphoramidite (PCl)^15^. or by the phosphorodiamidite reagent: 2-cyanoethyl N,N,N′,N′-tetraisopropylphosphorodiamidite (PN)^16,17^. The two methods complement each other since phosphitylation with the PCl reagent takes place under mild basic conditions, while the similar reaction with the PN reagent occurs under mild acidic conditions. In both cases the reaction times described in the literature are typically 1–5 h^18,19^, for nucleosides and the product requires purification by flash column chromatography with triethylamine as part of the eluent to prevent hydrolysis by exposure to silica gel. Subsequent removal of triethylamine is crucial since it would otherwise neutralize the tetrazole in the coupling step during solid phase oligonucleotide synthesis. This adds up to at least 12 h for the synthesis and purification of phosphoramidites before being submitted to oligonucleotide synthesis.

Phosphoramidites are preferably stored under inert atmosphere and at −20 °C to minimize oxidation and hydrolysis. It is however only practical to store phosphoramidites for routine use in solution on oligonucleotide synthesizers at ambient temperature, and this leads to degradation of the phosphoramidites, especially guanosine, by different autocatalytic (acrylonitrile elimination—Arbuzov Michael addition) and water-catalyzed pathways^20,21^.

To circumvent the problems related to the stability of phosphoramidites, it has previously been attempted to prepare phosphoramidites in situ by using PN^22–24^, PCl^25^ or similar reagents. However, the procedures have never gained broad use, since any residues of the PN reagent react with the 5′-OH of the oligonucleotide on the solid support, and as a consequence excess of the nucleoside is required and long reaction times are needed for complete consumption of PN, in particular for guanosine. Furthermore, a filtration step is often required which makes it difficult to implement the procedure in an automated setup^26^.

In this work, we propose a method for preparation of phosphoramidites, where the phosphitylating reagent is immobilized on a solid support, which allows rapid phosphitylation of nucleosides eluting through the solid support, immediately before submission to oligonucleotide synthesis. The method circumvents the problems related to storing phosphoramidites and the challenges of the previous in situ methods.

## Results and discussion

### Immobilization of the azole activator on a solid support

We were inspired by the mechanism of phosphitylation when using the PN reagent^27^. It proceeds via a two-step process involving activation of PN to give an intermediate P(III)-azolide followed by phosphitylation of the alcohol. We envisioned to integrate this in a flow process in which the activator is immobilized on a reusable solid support. When the heterocyclic activator is immobilized on a solid support, it becomes possible to load the resin with a given P(III)-reagent and remove salts and excess reagents by simple washing. Addition of the alcohol leads to transfer of the P(III)-reagent and elution of the desired phosphoramidite without leftovers of excess reagents and salts (since the leaving group is resin-bound) (Fig. 1a).

**Fig. 2:**
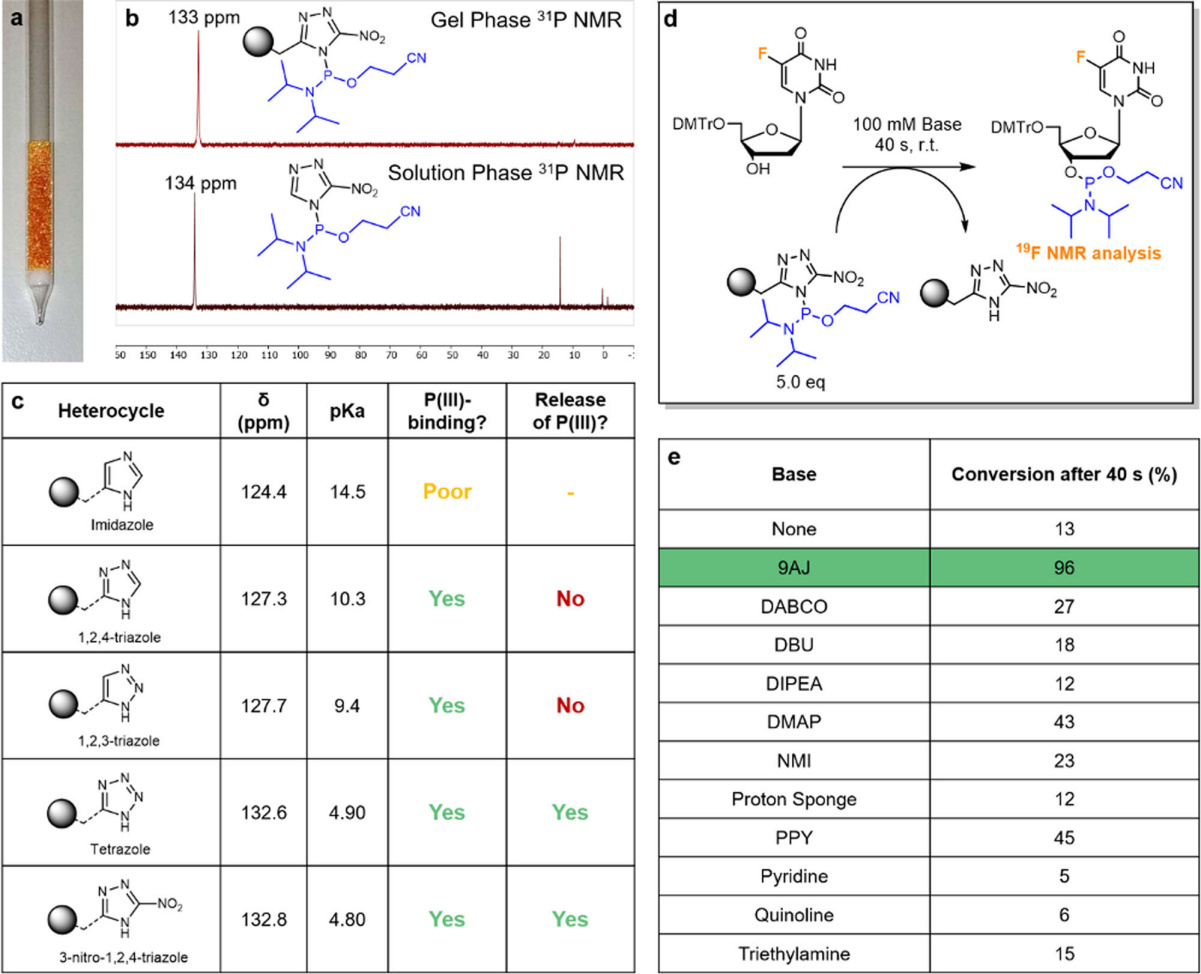
Optimization of the solid-phase azole and base additives. Initial study of resin-bound P((III) species with different azoles. **a** Picture of the modified NMR tube (figure by Alexander F. Sandahl). **b** Stacked spectra of resin-bound and solution phase P(III)-nitrotriazolide. **c** Summary of resin-bound azoles ability to bind and release P(III) upon elution with MeOH as measured by ^31^P NMR analysis. **d** Scheme depicting transfer of P(III) to alcohol using five equivalents resin-bound P(III) in the modified NMR tube with floxuridine as screening substrate for ^19^F NMR analysis. **e** Summary of conversion obtained after 40 s reaction time with various bases based on the amounts of product and starting material by ^19^F NMR analysis. 9AJ (highlighted in green) gave the highest yield for this study. 9AJ 9-azajulolidine, DABCO 1,4-diazabicyclo[2.2.2]octane, DBU 1,8-diazabicyclo[5.4.0]undec-7-ene, DIPEA *N*,*N*-diisopropylethylamine, DMAP 4-dimethylaminopyridine, NMI *N*-methylimidazole, PPY 4-pyrrolidinopyridine.

As the solid support we first used the TentaGel® resin (TG), which was functionalised with different azoles and these were treated with PCl in the presence of DIPEA (Fig. 2). A modified NMR tube (Fig. 2a) was used to monitor the loading and release of resin-bound P(III)-reagent by gel phase ^31^P NMR (Fig. 2b)^19,24,27^. The modified NMR tube is a useful analytical tool for monitoring resin-bound moisture and air-sensitive species. A series of azoles with different pKa values and with a suitable side chain for functionalization were selected (Fig. 2c and Supplementary Information 10–12). Studies on the reaction with PCl and subsequent release by reaction with methanol showed that the more acidic azoles, tetrazole and 3-nitro-1,2,4-triazole (nitrotriazole), were able to both bind and release the P(III)-species. The less acidic azoles reacted with the PCl but were not able to release the resin-bound P(III)-reagent again.

After screening different high-loading resins, the combination of nitrotriazole and commercially available (aminomethyl)polystyrene (AM-PS) showed the best properties in terms of hygroscopicity and P(III)-loading ability (Supplementary Information 14–24). The transfer of the loaded resin-bound P(III)-reagent to alcohols was however poor without any additives. The addition of base improved the yield and screening of several bases revealed that the series of 4-aminopyridines; 4-dimethylaminopyridine (DMAP), 4-pyrrolidinopyridine (PPY) and 9-azajulolidine (9AJ) are the best candidates for this. The trend of increasing yields (9AJ > PPY > DMAP) also matches their reactivity in acyl substitutions where these compounds are commonly used^27^. Optimal reaction rates are achieved when using 9AJ where a conversion of 96% was observed after 40 s when performed with five equivalents resin-bound P(III) in the NMR tube (Fig. 2d, e). Interestingly, it was observed that when mixing PCl and one of the three 4-aminopyridines a new broad upfield peak appeared on the ^31^P NMR spectra (Supplementary Information S33) likely corresponding to cationic P(III)-species. These may be the active phosphitylating species present during the transfer reaction generated by rapid reaction with the resin-bound P(III)-nitrotriazolide. However, the intermediate was not observed when performing a test reaction in solution with a preformed P(III)-nitrotriazolide (Supplementary Information S34).

### Synthesis of phorphoramidites in a flow system

**Fig. 3:**
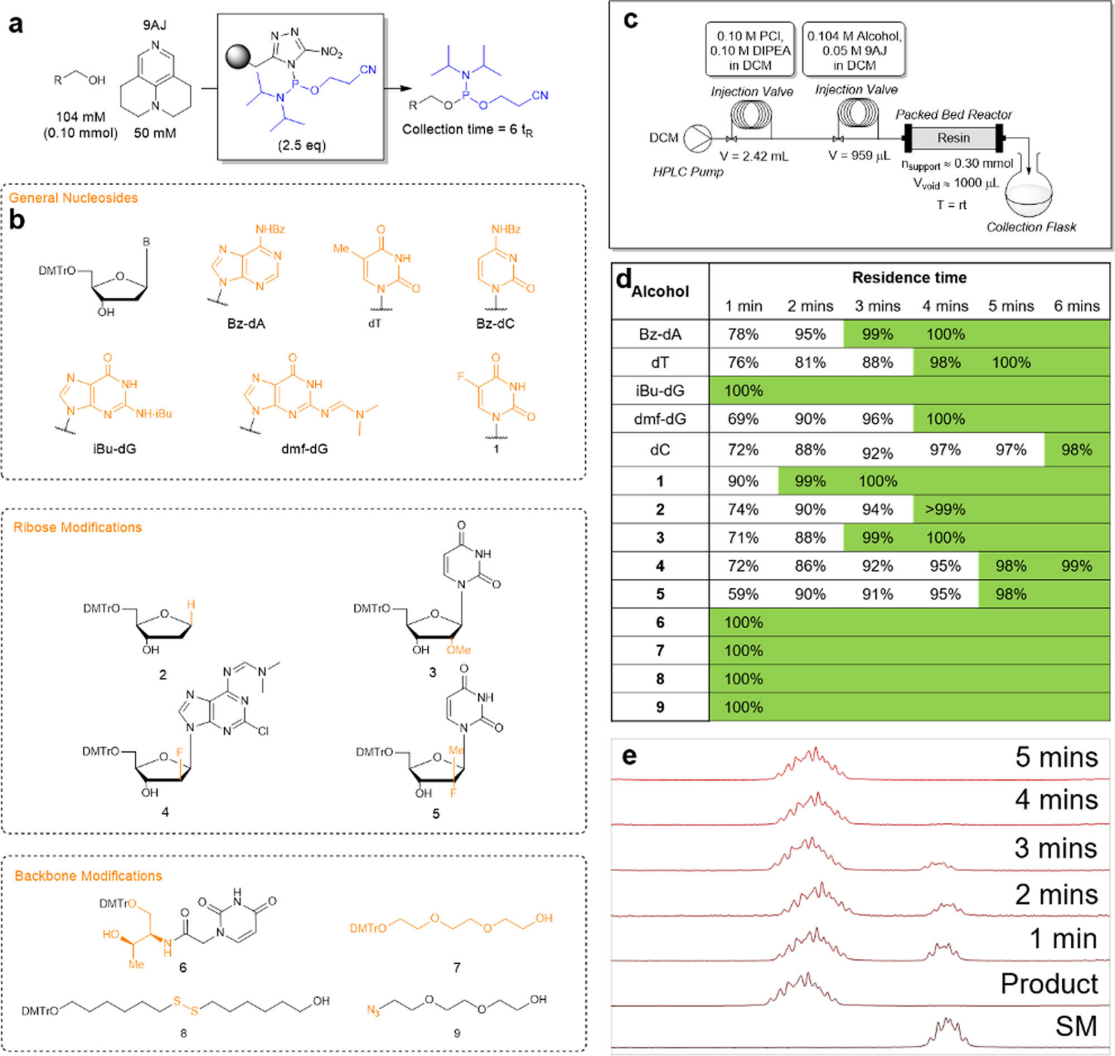
Residence time study. **a** General reaction scheme for the residence time study. **b** Structure of the 14 alcohols. **c** Schematic representation of the flow setup. **d** Residence time yields for the different alcohols based on the amount of product and starting material. Yields ≥98% have been highlighted in green. **e** Example of thymidine flowthrough residence time ^1^H NMR analysis. SM starting material.

In order to integrate the solid phase reaction in a flow system, the resin was packed into an HPLC column, working as a packed bed reactor. The reactor was coupled to a flow setup (Fig. 3c) consisting of a pump and two injection valves in series consisting of a PCl loop for loading the resin and an alcohol loop (containing 0.10 mmol alcohol). The whole system was backflushed with anhydrous CH_2_Cl_2_ stored under Ar atmosphere to ensure inert conditions. When the synthesis was finished, the PCl loop could be reloaded with MeOH/CH_2_Cl_2_ (1/9) to remove remaining resin-bound P(III)-species. The same packed bed reactor and resin batch were used for the whole study (>80 synthetic cycles) emphasizing the reusability of the resin.

The synthesis of ten nucleoside and four non-nucleoside phosphoramidites from the respective alcohols in the flow system was then demonstrated (Fig. 3). Residence times were tested for all substrates and they showed >98% conversion within 6 min, including the sterically hindered nucleoside analogues; 2′-O-methyluridine (**3**), protected clofarabine (**4**) and 2′-deoxy-2′-fluoro-2′-methyluridine (**5**) (Fig. 3b). It was also useful for the synthesis of phosphoramidites of the acyclic threoninol nucleic acid (aTNA) analogue **6** and the common TEG linker **7**. Furthermore, it was possible to synthesize the redox-unstable disulphide **8** and azide **9**. The azide-containing phosphoramidite **9-P** would otherwise not have been accessible by the conventional synthetic method as the product would degrade during purification. It was observed that **9-P** rapidly degrades if left standing in solution making this phosphoramidite especially unfeasable for storage (Supplementary Information 58–59). In general, there appears to be two types of reactivities observed; high reaction rate (residence time ≤ 1 min) or intermediate reaction rate (residence time ≥ 3 min), where the four non-nucleoside alcohols have a high reaction rate and the nucleosides have an intermediate reaction rate (Fig. 3d). Interestingly, iBu-dG also reacts with high reaction rate. We hypothesized this was due to an intramolecular hydrogen-bond from the exocyclic amide N–H of iBu-dG to the 5′-O stabilizing the 3′-endo conformation of the nucleoside, thus increasing the nucleophilicity of the 3′-OH by making it equatorial (Supplementary Information 47). This would not be favorable if the hydrogen-bond donor is removed which is the case for dmf-dG, where reactivity resembles the other nucleosides, and these may likely be in a 2′-endo conformation with axial and thereby less nucleophilic 2′-OH.

We hypothesize that the observed reactivites correlate with the nucleophilicity of the given alcohol, which is determined by the sterics around the functional group and the conformation of the whole molecule. It is however not possible to give a detailed explanation to why the pyridimidine-nucleosides dT and Bz-dC react slower than the purine-containing nucleosides Bz-dA and dmf-dG.

The eluate only contained the desired phosphoramidite, 9AJ and hydrolysis side products from the reaction between residual water and the resin-bound P(III)-reagent. In general, the crude ^31^P NMR purity was high except for dmf-dG where other signals at 145–146 ppm appeared likely corresponding to the phosphitylation of O6 of guanine, which has also been reported as a problem for the in situ method^26^.

### Using on-demand synthesized phosphoramidites in solid phase oligonucleotide synthesis

**Fig. 4:**
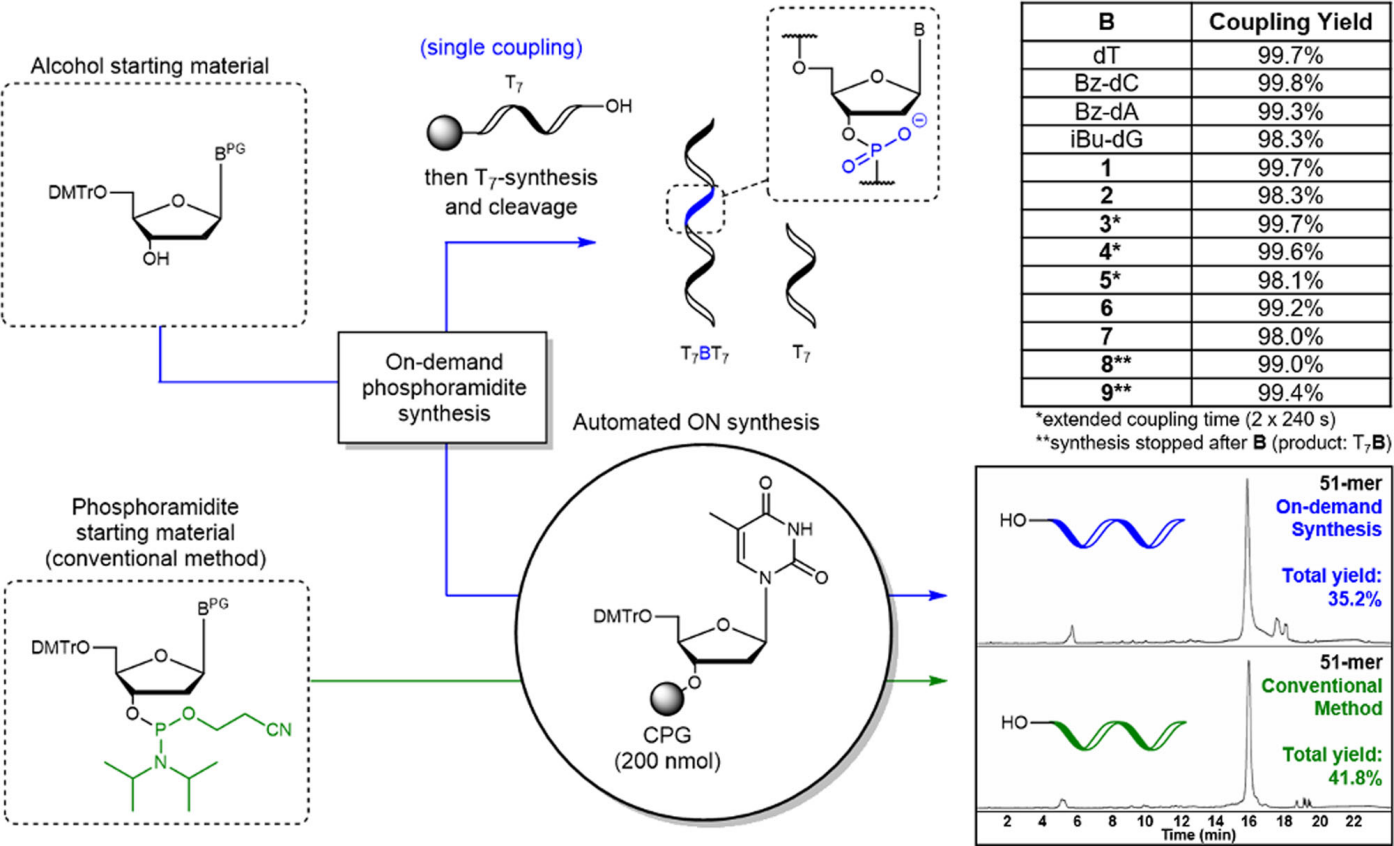
Application of on-demand synthesized phosphormamidites in oligonucleotide synthesis. Top: Single coupling of phosphoramidites prepared on-demand to a resin-bound T_7_ followed by elongation with another T_7_ segment and cleavage from the resin. Bottom: Synthesis of a 51-mer using solely the on-demand approach. HPLC chromatograms of crude material: by conventional solid-phase synthesis with stored phosphoramidites (green) or with the on-demand synthesized phosphoramidites (blue). 51-mer sequence: 5′CCG CTT TCT AGT TCG TCC TCC ATA ATT AAT TTC CTA GAG TCC TAC GTG CTC 3′. B (on-demand synthesized phosphoramidite), CPG (controlled pore glass).

Finally, the on-demand synthesized phosphoramidites were used in oligonucleotide synthesis and the overall scheme is shown on Fig. 4. We first investigated the yields of incorporation of a single on-demand synthesized phosphoramidite in the synthesis of a 15-mer oligonucleotide (T_7_-B-T_7_), where conventional phosphoramidites are used for the center position. In all schemes the flowthrough fractions were concentrated and otherwise used directly in a single coupling step during the automated oligonucleotide synthesis to determine the yield. All phosphoramidites showed more than 98% coupling yield and the four canonical nucleoside-phosphoramidites provided up to 99.8% coupling yield. For the sterically hindered phosphoramidites, **3-P**, **4-P**, and **5-P**, increased coupling times were required to achieve high coupling yields. It should be noted that 9AJ does not interfere with the coupling step and it has even been shown to be a beneficial additive for the reaction. Coupling was performed using 0.5 M activator as reported to be the optimal concentration when DMAP is present^28,29^.

Using solely on-demand prepared phosphoramidites, synthesized individually immediately before each coupling step, we synthesized a 51-mer oligonucleotide consisting of the four canonical nucleotides (Fig. 4, bottom). The average cycle yield of the syntheses with on-demand prepared phosphoramidites was 98.0% (35.2% total yield). We also prepared the same sequence by the conventional method using commercially available phosphoramidites. A slightly higher average yield of 98.3% (41.8% total yield) was obtained by the conventional method. When comparing the HPLC chromatograms of the oligonucleotides it appears that the the two methods provide very similar crude purity.

The on-demand synthesis method we have developed offers an attractive alternative to the conventional methods for synthesis of phosphoramidites in particular with regard to duration of the protocol and purity of the product. Since the on-demand method is significantly faster than the conventional method it may also be applied for the preparation of unstable phosphoramidites, such as the disulphide **8-P**, and azide **9-P**, which can then be applied to oligonucleotide synthesis before it decomposes. In terms of resources, the method is favorable for the small-scale synthesis of precious phosphoramidites, since there is no need for column chromatography and thereby no waste from silica and excessive use of solvents. However, for routine large-scale synthesis of standard phosphoramidites the conventional method is preferred since purification does not involve column chromatography but rather simple precipitation.

It is our belief that the on-demand synthesis of phosphoramidites has the potential for full integration in automated oligonucleotide synthesis. We have shown that the subsequent oligonucleotide synthesis tolerates the presence of 9AJ and residues of hydrolysis side products from the on-demand synthesis. Importantly, the nitrotriazole-AM-PS resin was highly recyclable, and the same resin was used for >80 synthesis cycles of phosphoramidites, where it was reactivated by PCl after each synthesis, without any signs of degradation. The remaining challenge to be solved is to integrate an inline concentration step in the flow system before submission of phosphoramidites to solid-phase synthesis and such concentration technology has been reported previously^26^. This may render the stable 5′-protected nucleosides as starting materials for oligonucleotide synthesis, and thereby eliminate all the issues concerning the synthesis and storage of phosphoramidites.

To conclude, the technology allows rapid synthesis of phosphoramidites from the corresponding alcohols without the need of intermediate purification before being submitted to automated oligonucleotide synthesis. The resin can be used for easy synthesis of phosphoramidites and it also has the potential of being fully integrated into an automated setup, where the nucleosides are stored and directly being converted to the phosphoramidites in a matter of minutes before being used in the subsequent coupling step. The setup is easy-to-use and could potentially be automated and operated by non-chemists.

## Methods

### Synthesis of the nitrotriazole-functionalized resin (AM-PS-Het5.1)

In CH_2_Cl_2_ (5 mL) was dissolved 2-(5-Nitro-4H-1,2,4-triazol-3-yl)acetic acid (172 mg, 1.00 mmol, 10 eq) and DIC (0.155 mL, 1.00 mmol, 10 eq), DIPEA (0.524 mL, 3.00 mmol, 30 eq), and HOBt·H_2_O (152 mg, 1.00 mmol, 10 eq) were added. The mixture was stirred at rt for 20 min and then added to Aminomethyl-Polystyrene resin (100 mg, 0.100–0.150 mmol, 1–1.5 mmol/g, 1 eq) in a plastic column (PD-10 from GE Lifesciences) and shaken overnight at rt. The resin was then washed with MeOH, CH_2_Cl_2_, and Et_2_O. The beads were analysed by Kaiser test which gave negative results for amines suggesting quantitative coupling yields.

### Description of the flow system

The tubing throughout the system contained of stainless steel tubing (1/16” OD × 0.75 mm ID) and connections were made with PEEK or stainless steel HPLC fittings (all with 1/16” ID). A HPLC pump (Knauer Azura P 4.1S) was used to pump CH_2_Cl_2_ through the reactor system that consisted of one backpressure regulator (PBR, 100 psi), two injections valves (2 position: load and inject, 6-port, 1/16”, Vici) in series, and a packed bed reactor prepared with resin. The exiting fluid was collected in a roundbottomed flask under argon atmosphere. To the two injection valves were connected two loops for loading of reagents: an alcohol loop (0.959 mL) and a PCl/DIPEA loop (2.42 mL). An empty HPLC column of stainless steel (76 mm length, 4.6 mm ID) was filled with **AM-PS-Het5.1** resin (approx. 250 mg, 1.00–1.50 mmol/g, 0.25–0.375 mmol) and sealed functioning as the packed bed reactor used for the whole study. The void volume determined to be 0.890 mL.

### General synthetic cycle for production phosphoramidites

The synthetic cycle used for general synthesis and screening of residence times consisted of a loading step (2.4 mL, 0.1 M PCl, 0.1 M DIPEA in CH_2_Cl_2_, with *t*_R_ = 1 min for 10 min) followed by a washing step (CH_2_Cl_2_, *t*_R_ = 1 min for 5 min) and at last transfer of resin-bound P(III) to the alcohol (0.959 mL, 0.104 M alcohol (0.100 mmol), 0.050 M 9AJ, in CH_2_Cl_2_, *t*_R_ = substrate-dependent, collected for six *t*_R_). The collected eluate was concentrated under reduced pressure and redissolved in anhydrous MeCN to reach a final concentration of 0.10 M, which could be used directly during automated oligonucleotide synthesis.

### Automated oligonucleotide synthesis

The synthetic cycle used for automated oligonucleotide synthesis is described in Table S7 (Supporting Information 60). For the single coupling study a T_7_-oligonucleotide was synthesized and then coupled with the an on-demand prepared phosphoramidite. Afterwards another T_7_-oligonucleotide was synthesized onto the oligonucleotide followed by cleavage and deprotection of the product and truncated oligonucleotide using AMA (1:1 40% methylamine/30–33% ammonium hydroxide) for 30 min at 50 °C. The yield of the single coupling reaction was determined from the ratio between the full-length product and the truncated T_7_-oligonucleotide. Furthermore, a 51-mer was produced using solely on-demand synthesized phosphoramidites and the yield determined based on isolated amounts of product oligonucleotide.

## Supplementary information

Supplementary Information

## Data Availability

The data that support the findings of this study are available from the corresponding author upon reasonable request.
