# Supplementary material for: On-demand synthesis of phosphoramidites
Source: Nat Commun. 2021 May 12;12:2760. doi: 10.1038/s41467-021-22945-z (PMC8101336; doi:10.1038/s41467-021-22945-z)
Supplement: Supplementary file 1 — Supplementary Information [file 41467_2021_22945_MOESM1_ESM.pdf]

Supplementary Information for

On-Demand Synthesis of Phosphoramidites

Alexander F. Sandahl, Thuy J. D. Nguyen, Rikke A. Hansen, Martin B. Johansen, Troels Skrydstrup, Kurt V. Gothelf

Correspondence to: kvg@chem.au.dk

Table of Contents

Supplementary Methods	3
Data Availability	3
Solution Phase Reference Results	4
Synthesis of Heterocycles	10
Functionalization of Resins	11
Modification of NMR Tube	13
Loading Studies	14
Hygroscopicity Studies	20
Overlay of Reference Spectra for ^{19}F NMR Analysis	25
Base Screen	26
^{31}P NMR Study of Aminopyridine Intermediates	33
Flow System Setup	35
PCl Stability	37
Diffusion Study	38
Concentration Screen of 9AJ	39
Synthesis of Starting Material Alcohols	40
Residence Time Study	47
Stability of Phosphoramidite **9-P** in Solution	58
Synthesis and Characterization of Reference Oligonucleotides	60
Single Coupling Reactions	64
Oligonucleotide Synthesis	79
Synthesis of Reference Phosphoramidites	80
NMR Spectra of P(III)-loaded Resins	85
NMR Spectra of Synthesized Starting Materials and Reference Products	90
NMR Spectra of 9AJ and 2-cyanoethyl *N*,*N*-diisopropylphosphonamidate	112
NMR Spectra (Full) of Flowthrough (Stacked with References)	114
Supplementary References and Notes	128

Supplementary Methods

All chemicals were purchased from Sigma-Aldrich, Carbosynth, and Link Technologies Ltd. in Scotland and used without further purification. Solvents were of HPLC grade and anhydrous solvents were purchased in Sure/Seal bottles with inert atmosphere or dried prior use by an M-BRAUNsolvent purification system. Yields refer to mass of isolated compounds unless otherwise stated. Reactions were monitored by thin-layer chromatography (TLC) on Merck silica 60 F254 plates and visualised by exposure to UV (254 nm) or by staining with solutions of molybdic acid, potassium permanganate, ninhydrin, vanillin, or *p*-anisaldehyde. Flash column chromatography was performed using Merck silica gel 60 (230-400 mesh) as stationary phase. NMR spectra were recorded on a Bruker BioSpin GmbH AscendTM 400 and were calibrated using deuterated solvents (MeCN, DMSO, Chloroform). ^{1}H NMR was recorded at 400 MHz, ^{13}C NMR was recorded at 101MHz, ^{19}F NMR was recorded at 376 MHz and ^{31}P NMR was recorded at 162 MHz. Chemical shifts are reported in parts per million and following abbreviations were used to explain multiplicities: s = singlet, d = doublet, t = triplet, q = quartet, m = multiplet. Coupling constants are reported in Hz. HRMS was performed using electrospray ionization on a Bruker Daltonics MicrOTOF.

Oligonucleotides were synthesised in house on a BioAutomation MerMade-12 automated oligonucleotide synthesiser using reagents and preloaded 1000 Å CPG columns purchased from Link Technologies Ltd. in Scotland. Phosphoramidites were synthesised in house or purchased from Link Technologies Ltd. in Scotland. Oligonucleotide synthesis was carried out under standard conditionsunless otherwise states. Synthesised oligonucleotides were cleaved from solid support using AMA (1:1 40% methylamine/30-33% ammonium hydroxide). The mass was confirmed by UHPLC-ESI-TOF on a Shimadzu LCMS-2020 system. All oligonucleotides were HPLC purified on a Hewlett-Packard Agilent Expand C-18 stationary column using the following methods (Solvent A: 0.1 M Triethylammonium acetate, pH = 7; Solvent B: MeCN; Gradient: 5% to 20% B over 15 mins, 20% to 70% B 15-20 mins or 5% to 15% B over 15 mins, 15% to 70% B 15-20 mins).

Solution Phase Reference Spectra

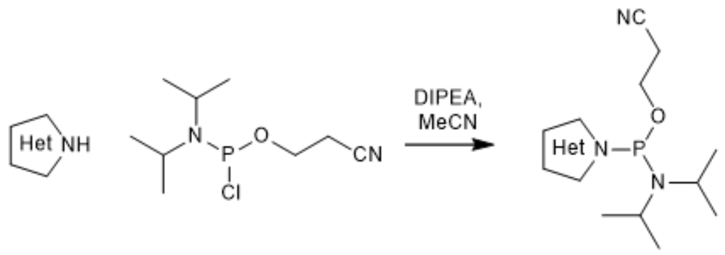

General procedure:

Heterocycles (0.10 M, 0.10 mmol) were dissolved in MeCN (1.0 mL) and DIPEA (0.030 M, 0.030 mmol) was added along with PCl (0.020 M, 0.020 mmol). NMR spectra were recorded after 10 mins.

Imidazole

^{31}P NMR (162 MHz, MeCN) δ_P (ppm) 124.6

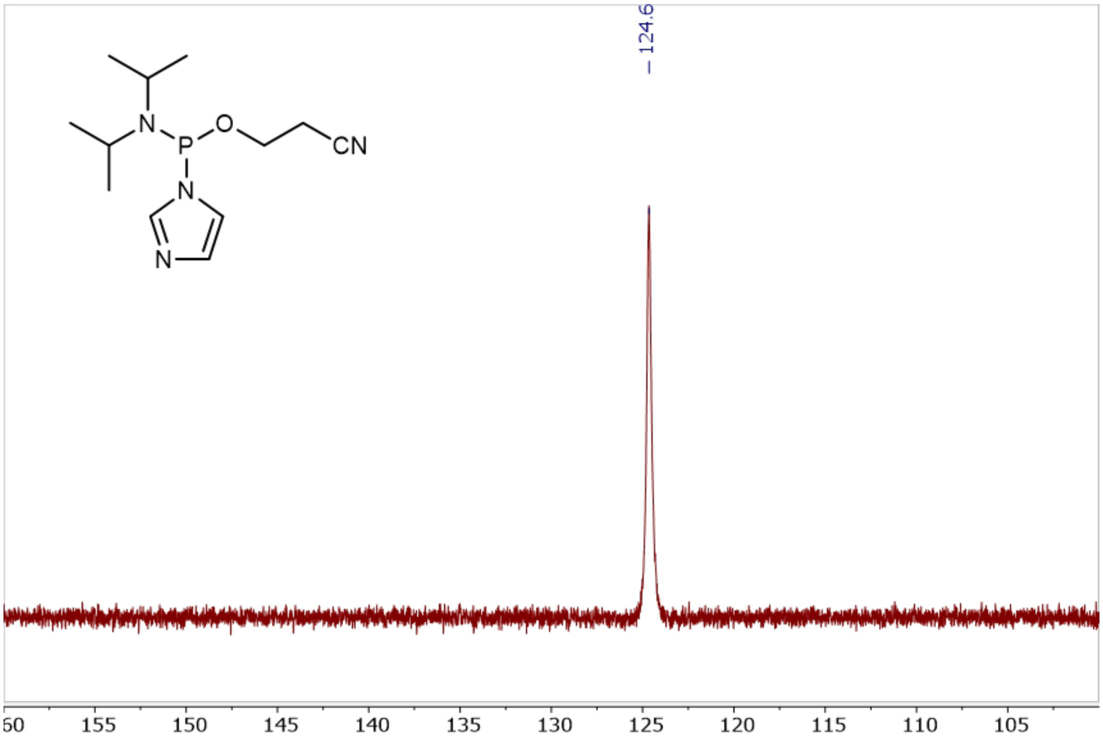

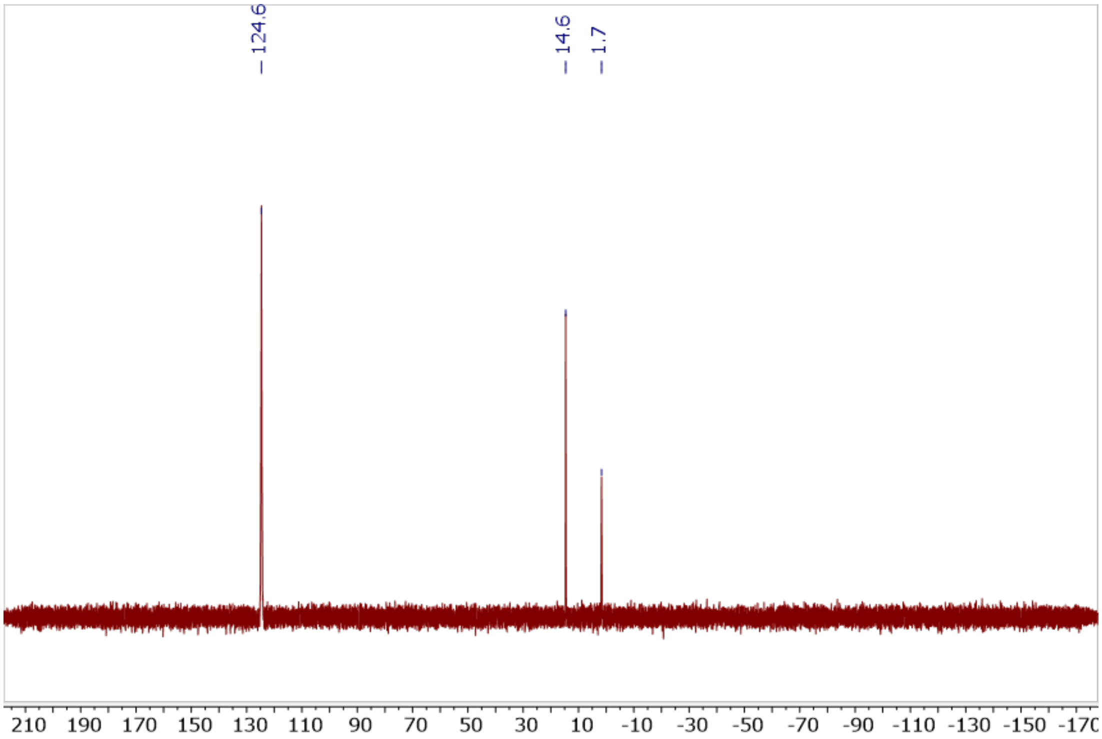

1,2,4-Triazole

^{31}P NMR (162 MHz, MeCN) δ_P (ppm) 126.9

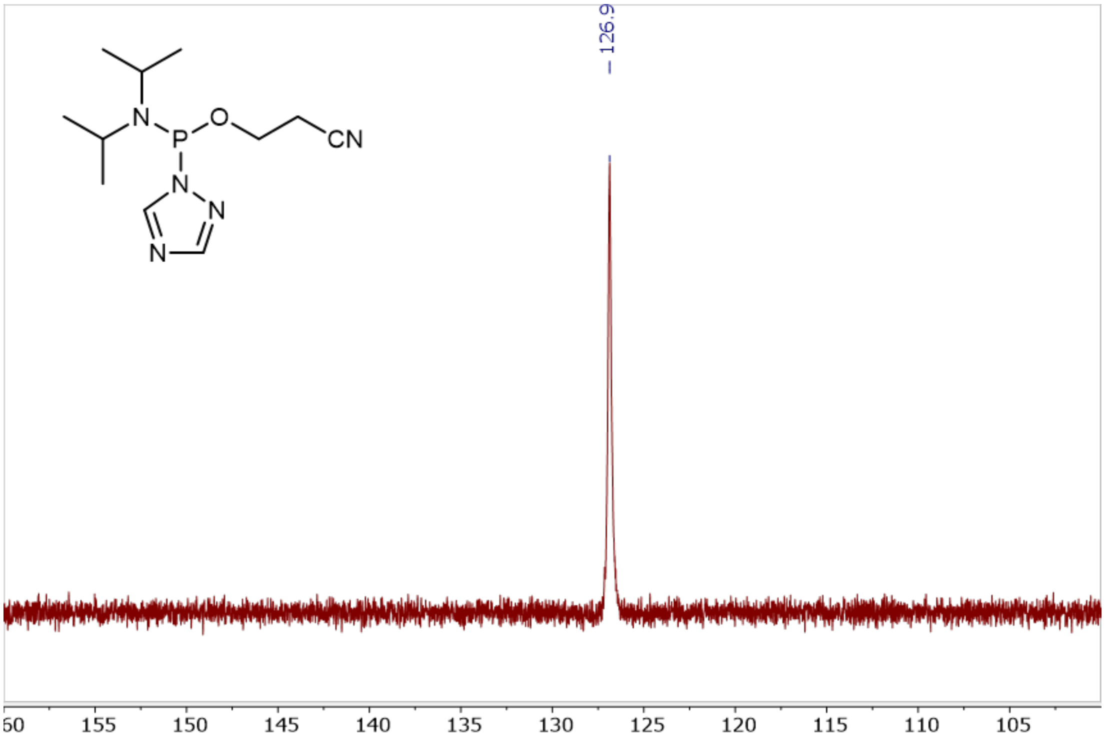

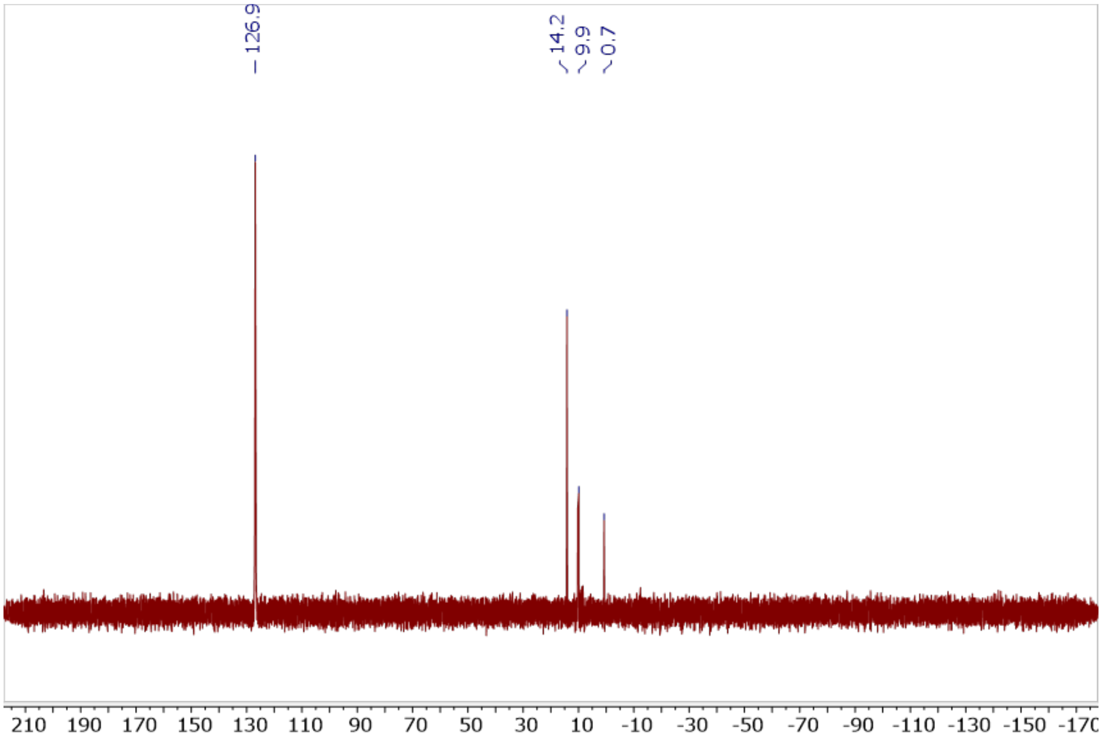

1,2,3-Triazole

^{31}P NMR (162 MHz, MeCN) δ_P (ppm) 130.6

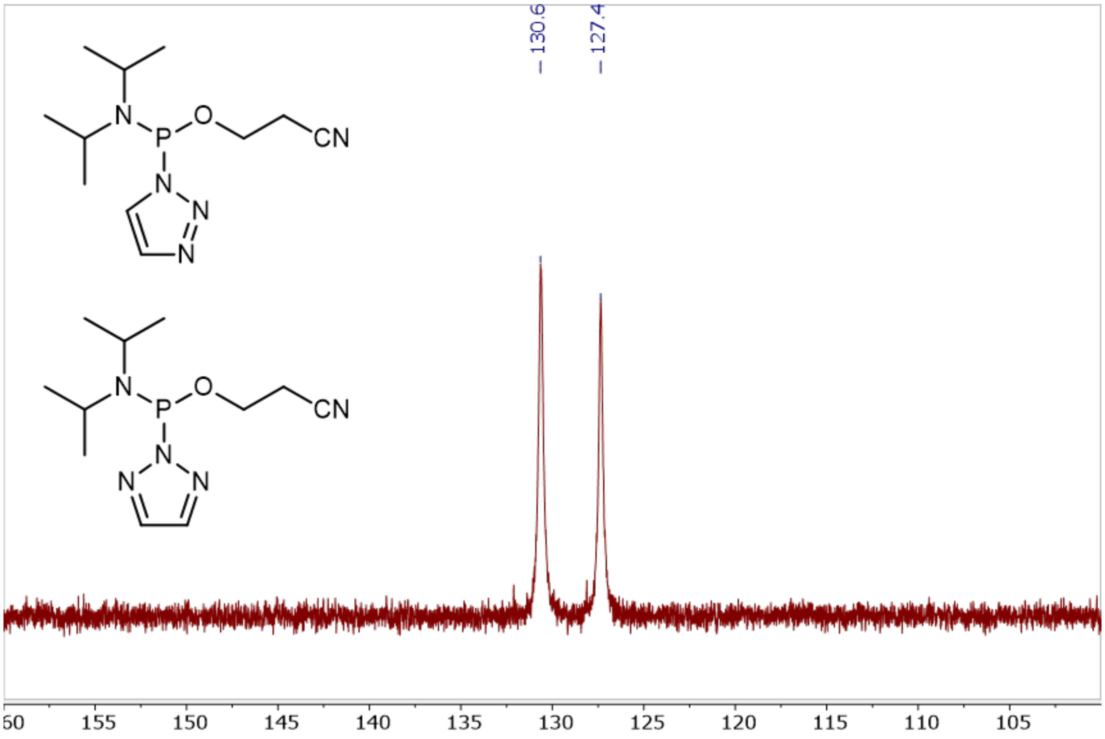

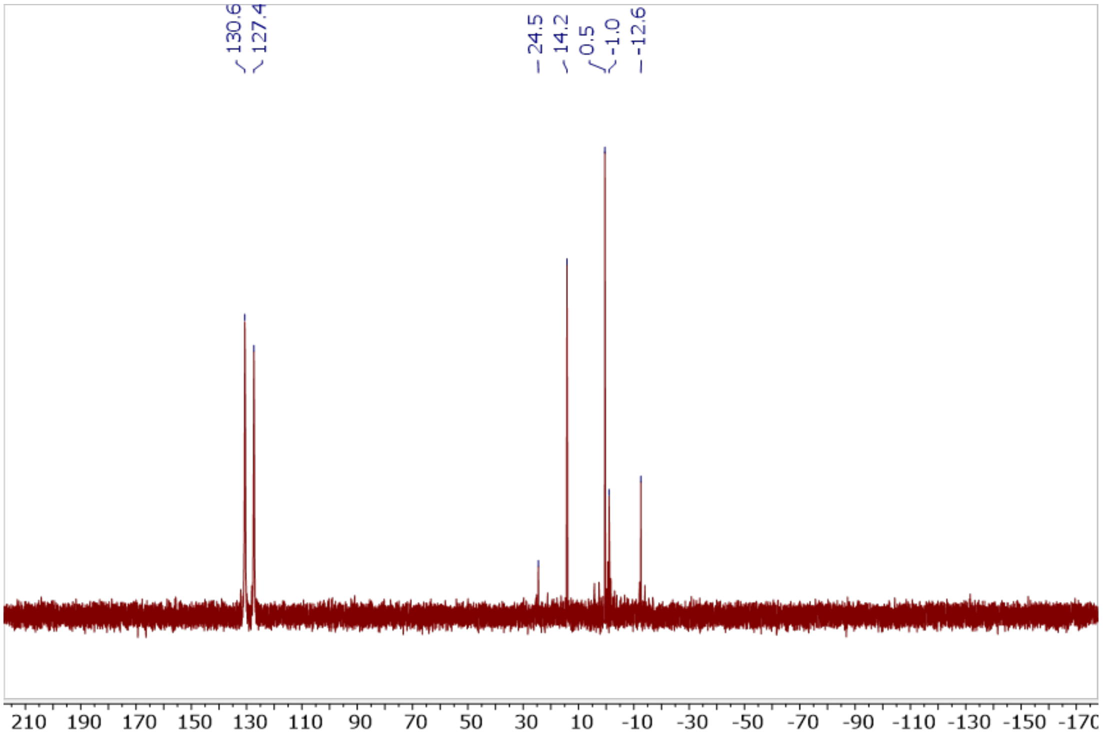

Tetrazole

^{31}P NMR (162 MHz, MeCN) δ_P (ppm) 132.2

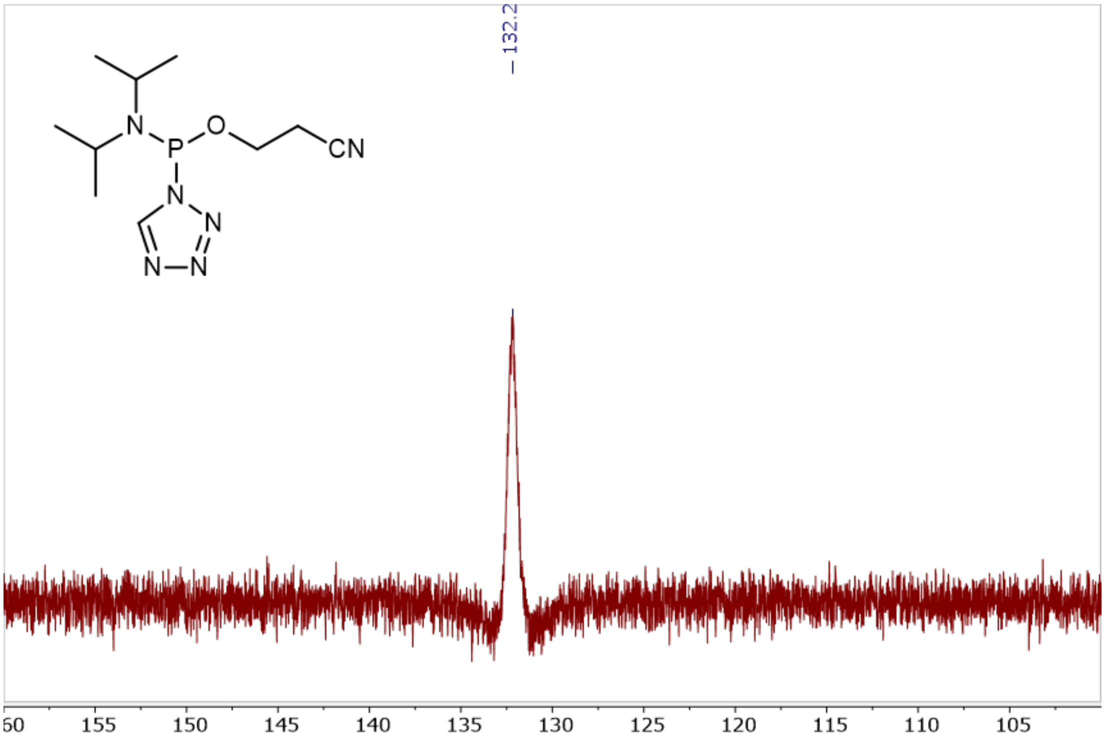

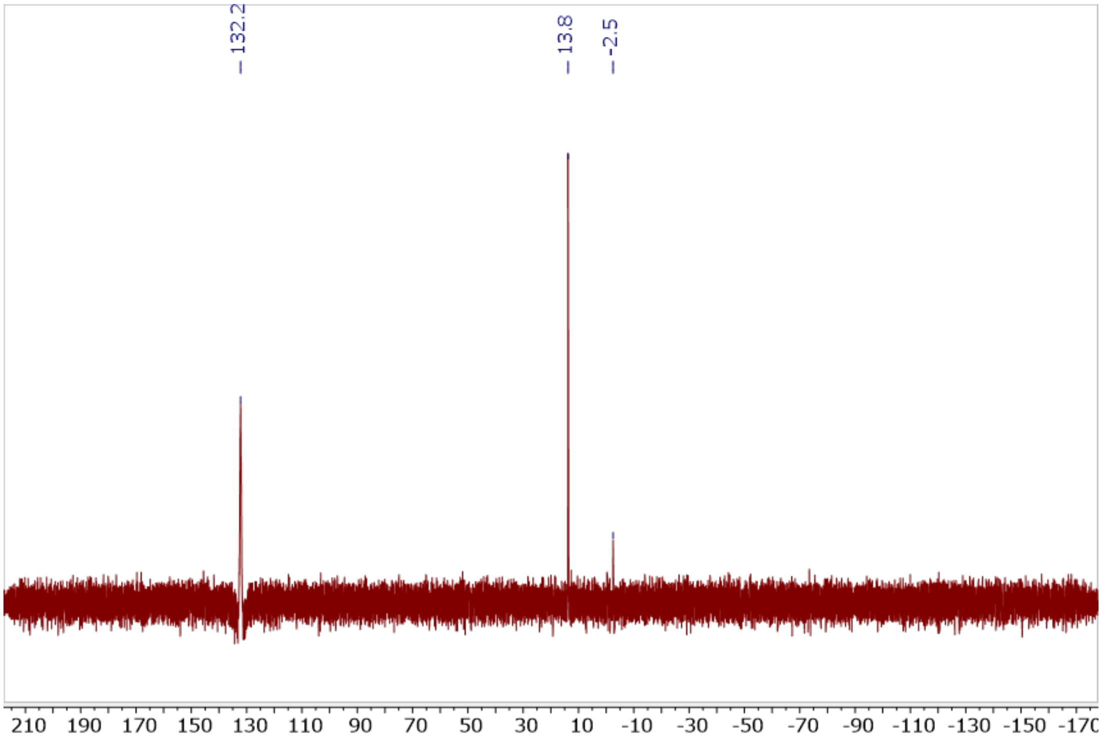

3-Nitro-1,2,4-triazole

^{31}P NMR (162 MHz, MeCN) δ_P (ppm) 134.7

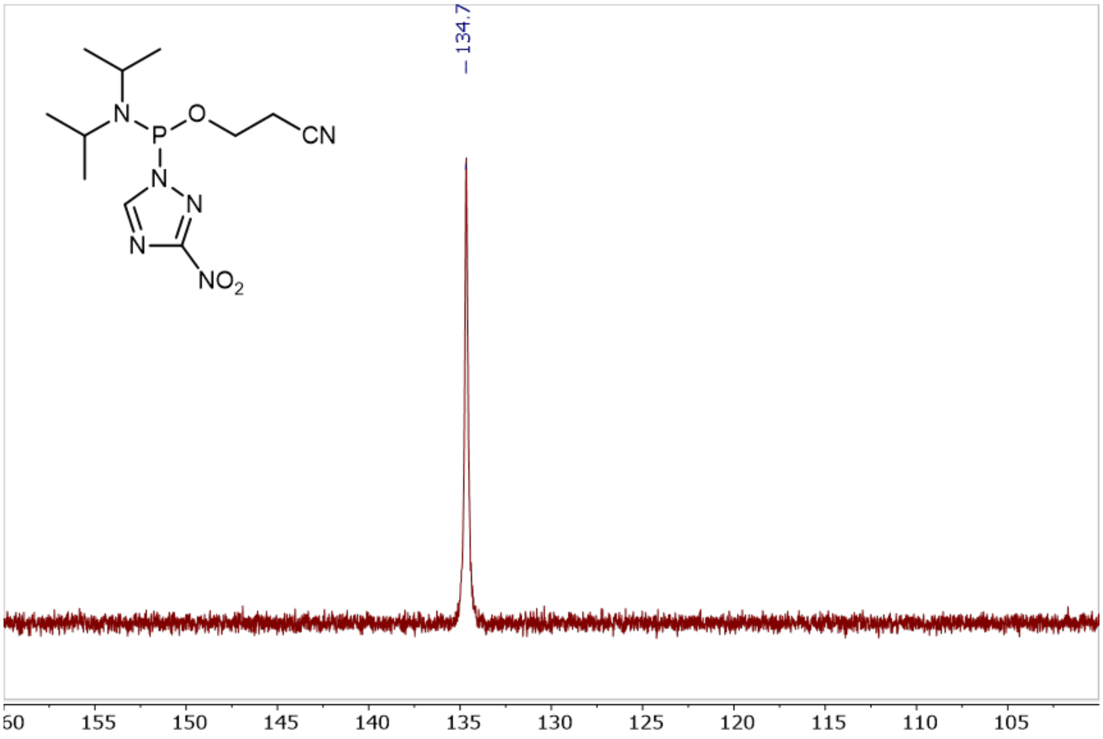

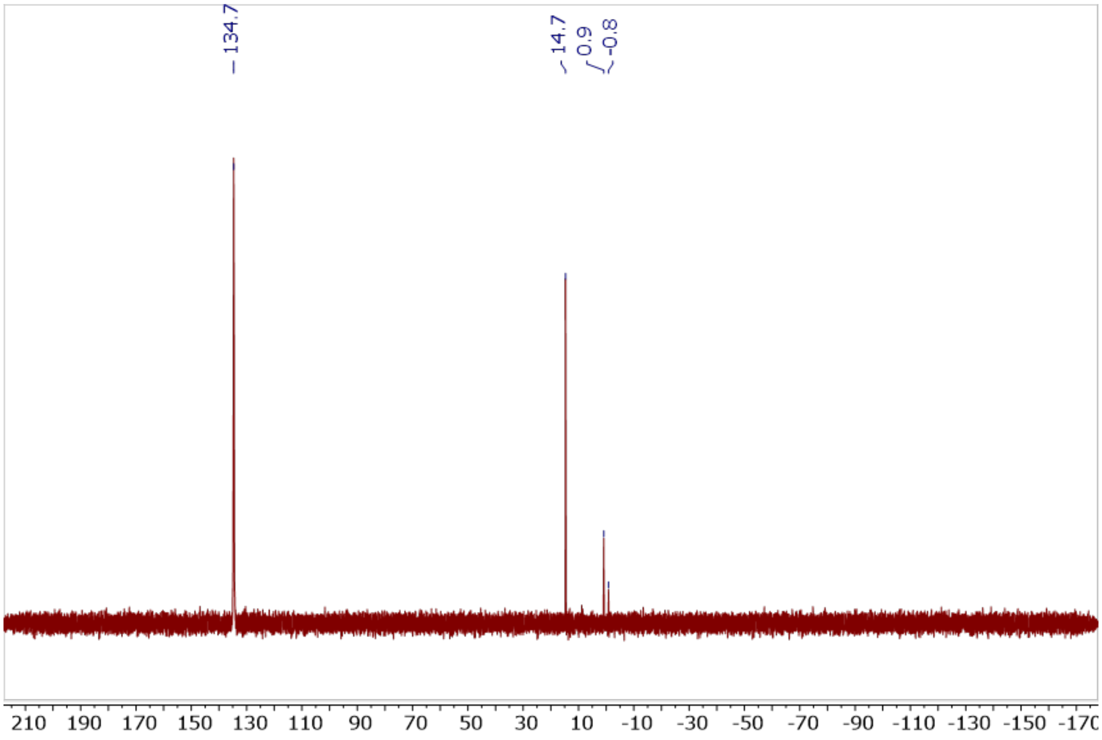

Synthesis of Heterocycles

Synthesis of **Het3**

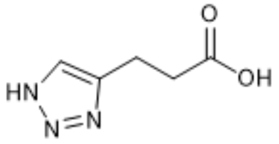

3-(1H-1,2,3-Triazol-4-yl)propanoic acid was prepared according to previously published procedures and reported data are consistent with reported values.[1]

Synthesis of **Het5**

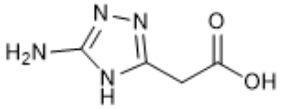

5-Amino-1,2,4-3yl-acetic acid was prepared according to previously published procedures and data are consistent with reported values.[2]

Synthesis of **Het5.1**

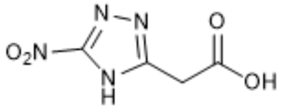

2-(5-Nitro-4H-1,2,4-triazol-3-yl)acetic acid was prepared according to previously published procedures and data are consistent with reported values.[3]

Functionalization of Resins

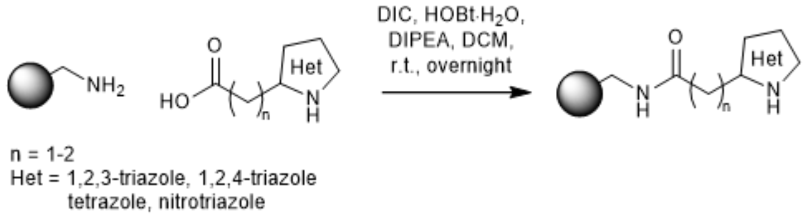

General procedure for coupling between amine-functionalised resins and carboxylic acids

The carboxylic acid (0.205 M, 1.03 mmol, 10 eq) was dissolved in 5 mL CH_2Cl_2 and DIC (0.205 M, 1.03 mmol, 10 eq), DIPEA (0.615 M, 3.08 mmol, 30 eq), and HOBt·H_2O (0.205 M, 1.03 mmol, 10 eq) were added. The mixture was stirred at rt for 20 mins and then added to the amine-functionalised resin (0.103 mmol, 1 eq) in a plastic column (PD-10 from GE Lifesciences) and shaken overnight at rt. The resin was then washed with MeOH, CH_2Cl_2, and Et_2O. The beads were analysed by Kaiser test which gave negative results for amines suggesting quantitative coupling yields.

This procedure was performed for the following resin and heterocycle combinations:

TentaGel™ S-NH_2 (TG) was functionalised with
2-(4H-1,2,4-triazol-3-yl)acetic acid (**TG-Het2**)
3-(1H-1,2,3-triazol-5-yl)propanoic acid (**TG-Het3**)
2-(1H-tetrazol-5-yl)acetic acid (**TG-Het4**)
2-(5-nitro-4H-1,2,4-triazol-3-yl)acetic acid (**TG-Het5.1**)

Amino-SynBase™ Controlled Pore Glass 3000/110, LCAA (CPG) was functionalised with
2-(1H-tetrazol-5-yl)acetic acid (**CPG-Het4**)
2-(5-nitro-4H-1,2,4-triazol-3-yl)acetic acid (**CPG-Het5.1**)

(Aminomethyl)polystyrene (AM-PS) was functionalised with
2-(1H-tetrazol-5-yl)acetic acid (**AM-PS-Het4)**
2-(5-nitro-4H-1,2,4-triazol-3-yl)acetic acid (**AM-PS-Het5.1)**

PEGA was functionalised with
2-(1H-tetrazol-5-yl)acetic acid (**PEGA-Het4**)

Aminomethyl ChemMatrix® (AM-CM) was functionalised with
2-(1H-tetrazol-5-yl)acetic acid (**AM-CM-Het4**)

TentaGel® XV HMPA (TG-XV) Resin was functionalised with
2-(1H-tetrazol-5-yl)acetic acid(**TG-XV-Het4**)

HypoGel® RAM Resin (HypoGel) was functionalised with
2-(1H-tetrazol-5-yl)acetic acid (**HypoGel-Het4**)

Aminobutyl Polystyrene (AB-PS) was functionalised with
2-(1H-tetrazol-5-yl)acetic acid (**AB-PS-Het4**)

Imidazole functionalization of TentaGel™ S-NH_2 (**TG-Het1**)

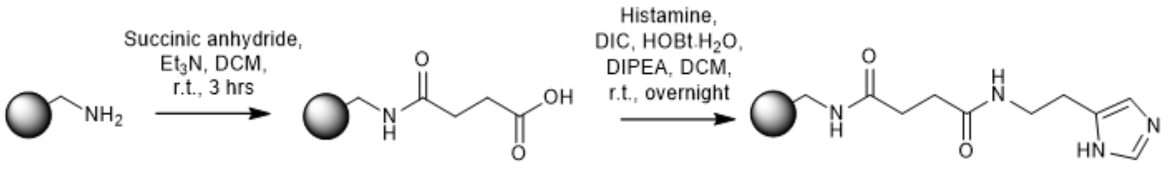

Succinic anhydride (100 mg, 1.0 mmol, 7.4 eq) was dissolved in CH_2Cl_2 (5 mL) and Et_3N (0.138 mL, 1.0 mmol, 7.4 eq) was added. The mixture was added to TentaGel™ S-NH_2 (300 mg, 0.45 mmol/g, 0.135 mmol, 1.0 eq) in a plastic tube and shaken at rt for 3 hrs. The resin was washed with CH_2Cl_2 and Et_2O. The beads were analysed by Kaiser test which gave negative results for amines meaning quantitative coupling yield.

A solution of DIC (0.11 mL, 0.68 mmol, 5.0), HOBt·H_2O (103 mg, 0.24 mmol, 1.8 eq), and DIPEA (0.35 mL, 0.72 mmol, 5.3 eq) in CH_2Cl_2 (4 mL) was added to the resin and shaken at rt for 30 mins. Histamine (75 mg, 0.68 mmol, 5.0 eq) was then added to the mixture and tube was shaken overnight at rt The beads were then washed with MeOH, CH_2Cl_2 and Et_2O.

Modification of NMR Tube

The bottom of an NMR tube (outer diameter: 5 mm) was cut off using a diamond tipped glass cutter (**1**). The glass was then melted under a torch and pulled with a tweezer (**2**). After cooling, a filter was added to the NMR tube (**3**). The resin could then be added and used for the following experiments (**4**).

Instructions on how to make modified NMR tube

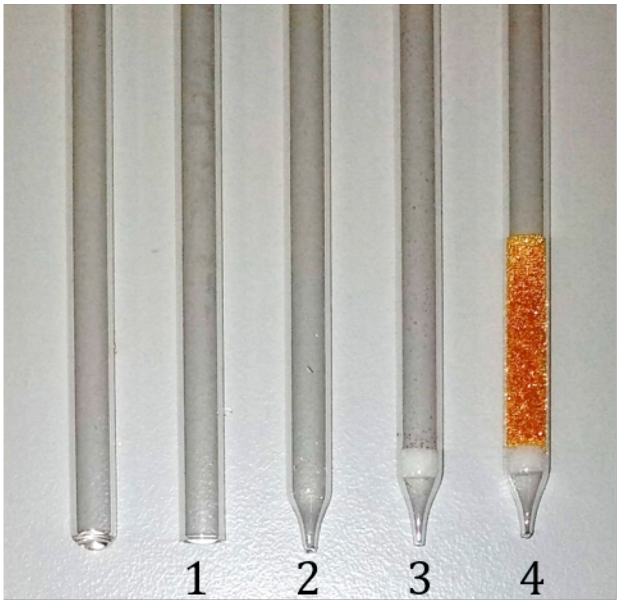

Figure S1| Step-by-step guide on how to prepare the modified NMR tubes. Figure by Alexander F. Sandahl

Loading Studies

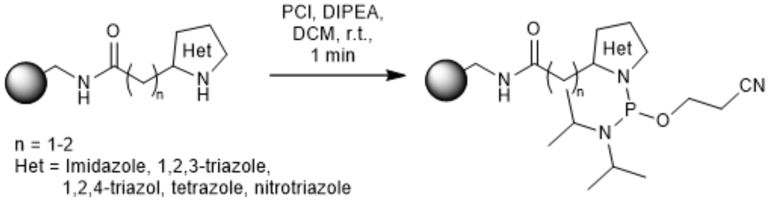

A modified NMR tube was loaded with TG-heterocycle resin (50 mg, 0.4 mmol/g, 0.2 mmol). The resin was washed with CH_2Cl_2 (2 mL), and a solution of PCl (0.10 M, 0.20 mmol) and DIPEA (0.10 M, 0.20 mmol) in CH_2Cl_2 (2 mL) was eluted over 1 min. The resin was then washed with CH_2Cl_2 (2 mL) and a Gel Phase ^{31}P NMR spectrum was recorded. MeOH (1 mL) was then eluted through the resin followed by CH_2Cl_2 (2 mL) and a new Gel Phase ^{31}P NMR spectrum was recorded.

It should be noted that a signal around 143-144 ppm is observed for imidazole (**TG-Het1**) and tetrazole (**TG-Het4**) which does not correlate with the P(III)-azolide. A likely structure may be the P(III) bound to the neighbouring amide to give a P(III)-imidate.

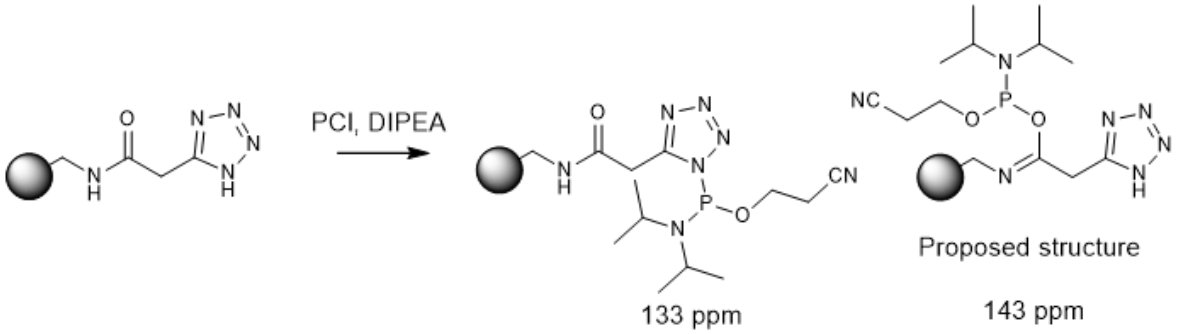

TG-Het1 (Imidazole)

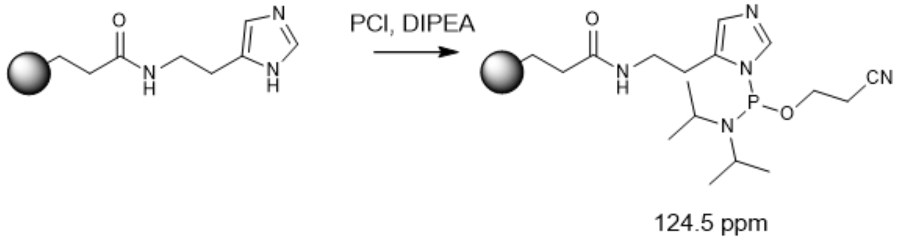

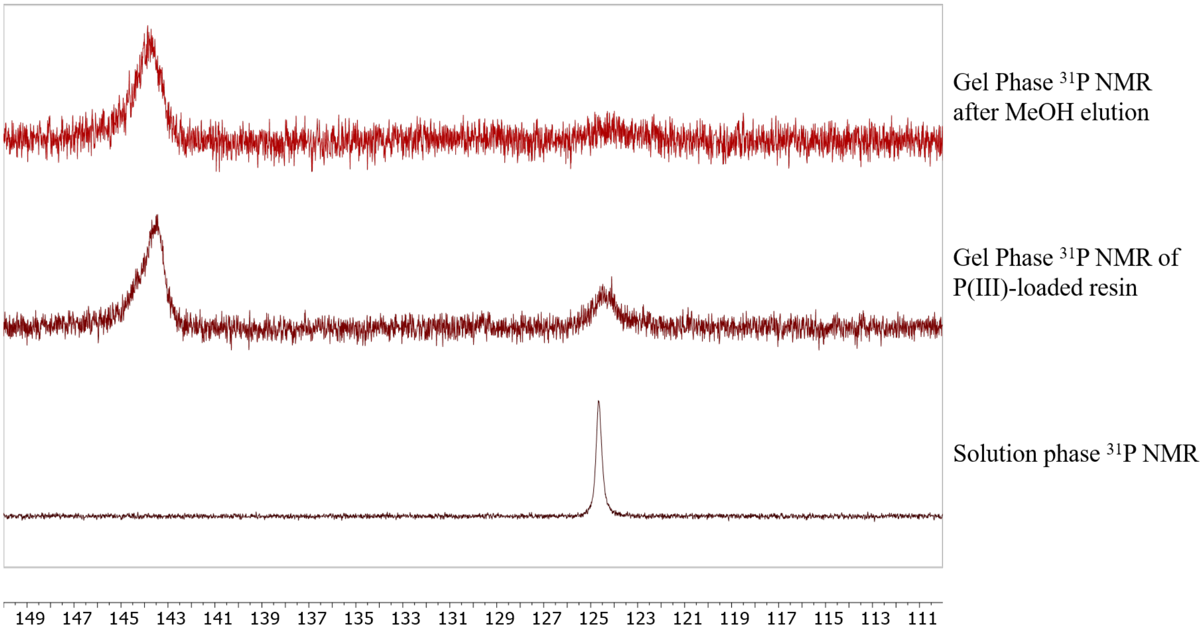

TG-Het2.1 (1,2,3-Triazole)

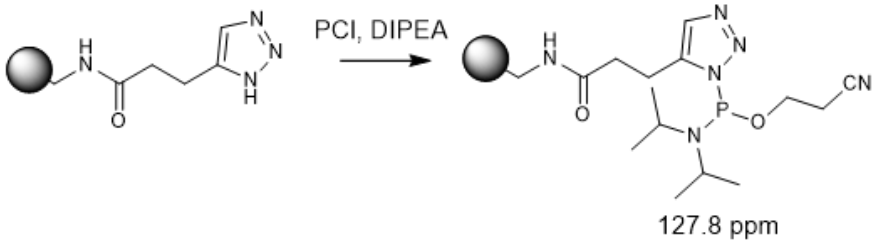

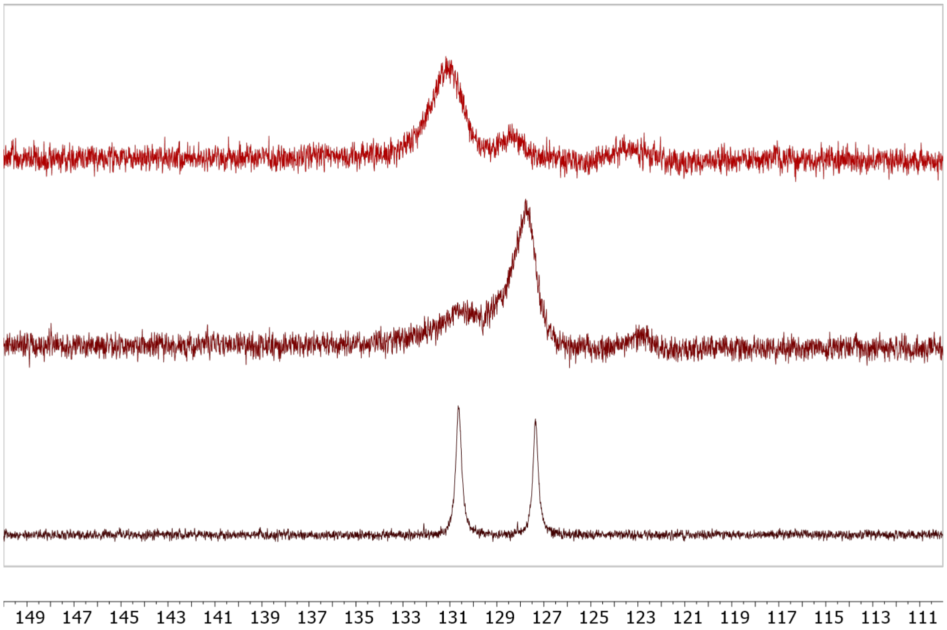

Gel Phase ^{31}P NMR after MeOH elution

Gel Phase ^{31}P NMR of P(III)-loaded resin

Solution phase ^{31}P NMR

TG-Het3 (1,2,4-Triazole)

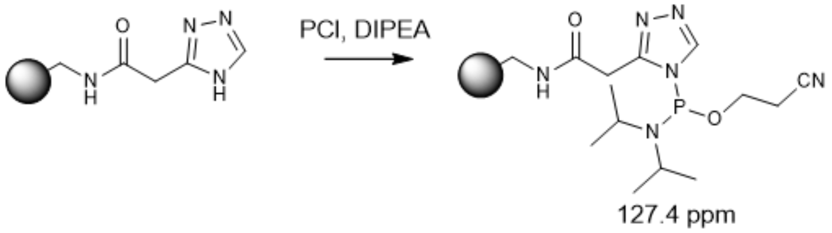

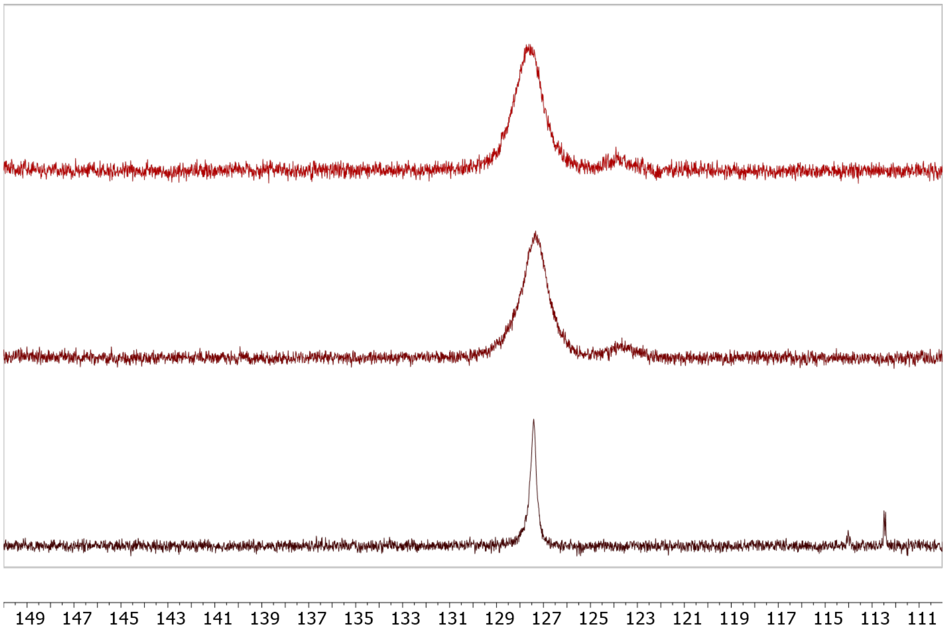

Gel Phase ^{31}P NMR after MeOH elution

Gel Phase ^{31}P NMR of P(III)-loaded resin

Solution phase ^{31}P NMR

TG-Het4 (Tetrazole)

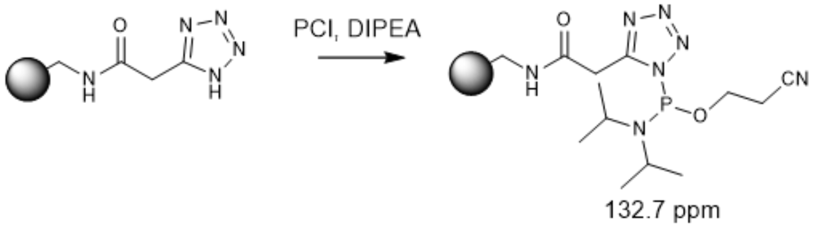

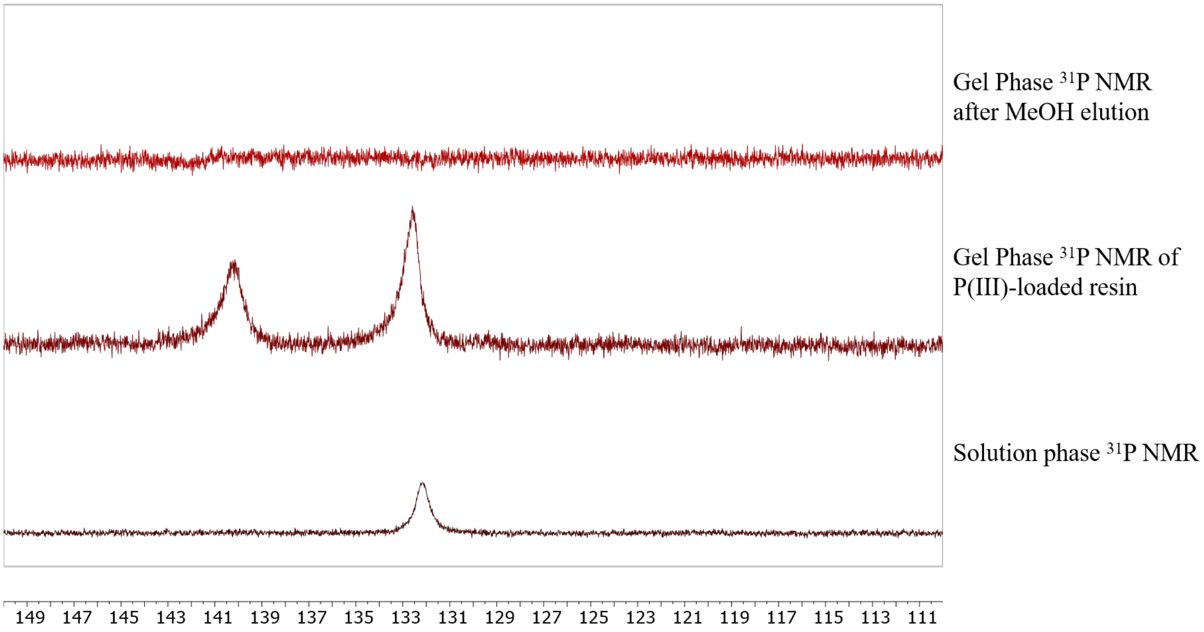

TG-Het5.1 (Nitrotriazole)

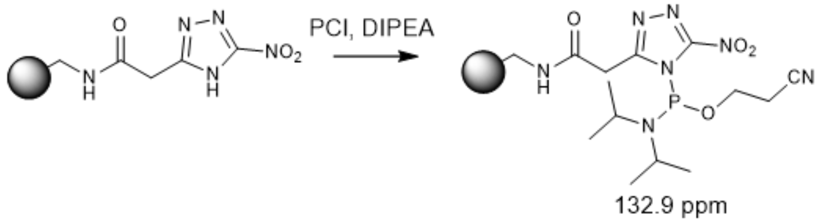

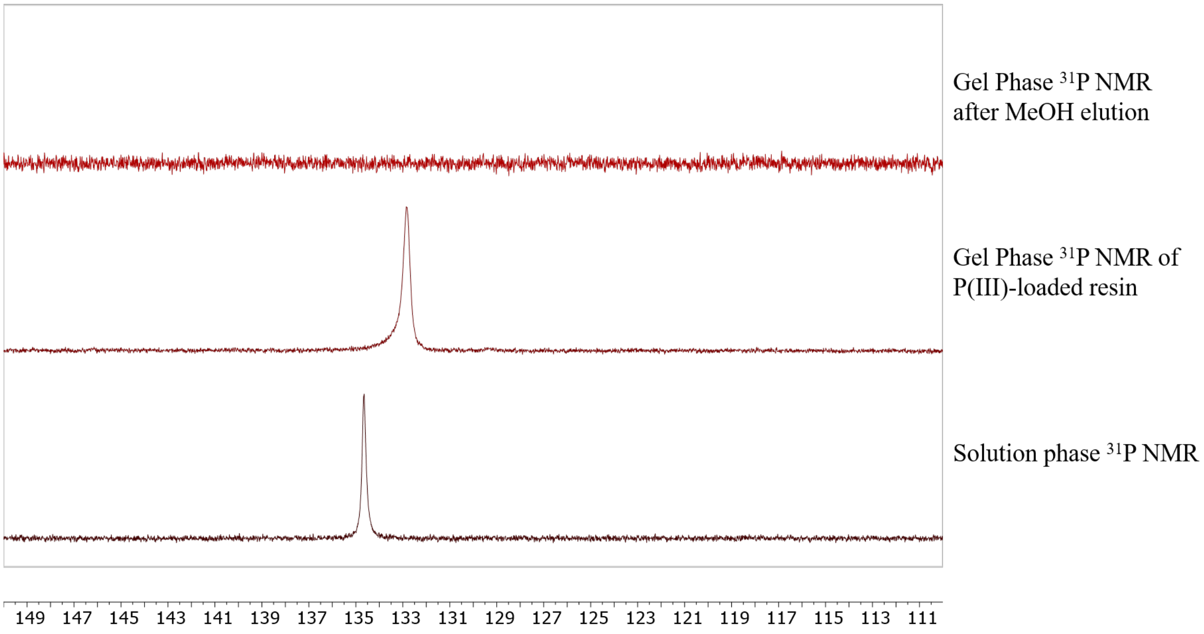

Hygroscopicity Studies

Modified NMR tube was loaded with tetrazole-functionalised resin (50 mg, 0.050-0.075 mmol tetrazole). The resin was washed with CH_2Cl_2 (2 mL) and then loaded with with PCl (0.10 M, 0.20 mmol) and DIPEA (0.1 M, 0.20 mmol) in CH_2Cl_2 (2 mL) over 1 min. The resin was then washed with CH_2Cl_2 (2 mL) and Gel Phase ^{31}P NMR spectra were recorded over time. Signals in the range of 120-140 ppm correspond to the P(III)-azolides. The signal around 15 ppm corresponds to the H-phosphonate from hydrolysis and the signals around 0 ppm corresponds to P(V) species through oxidation.

AM-PS-Het4

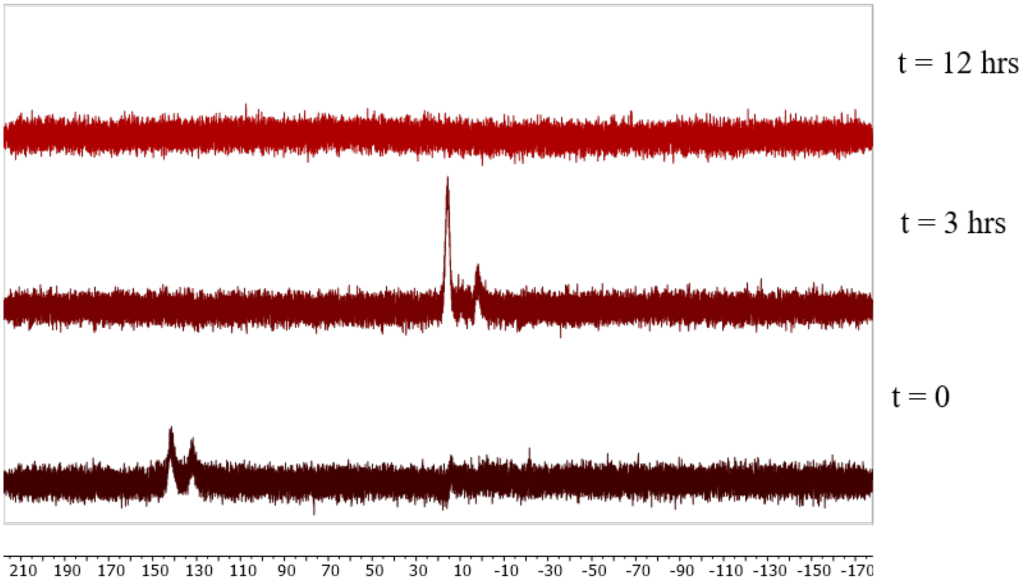

PEGA-Het4

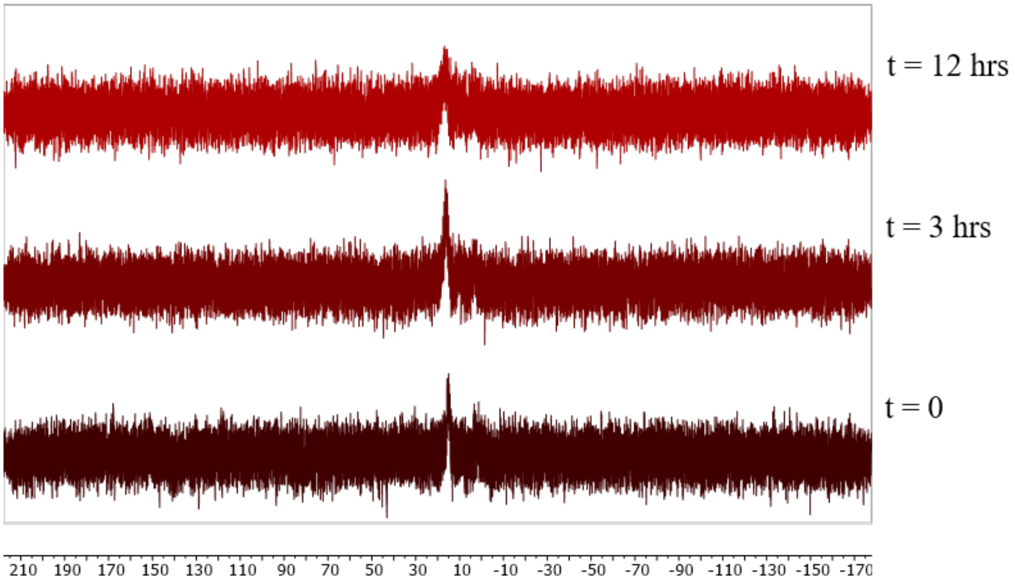

AM-CM-Het4

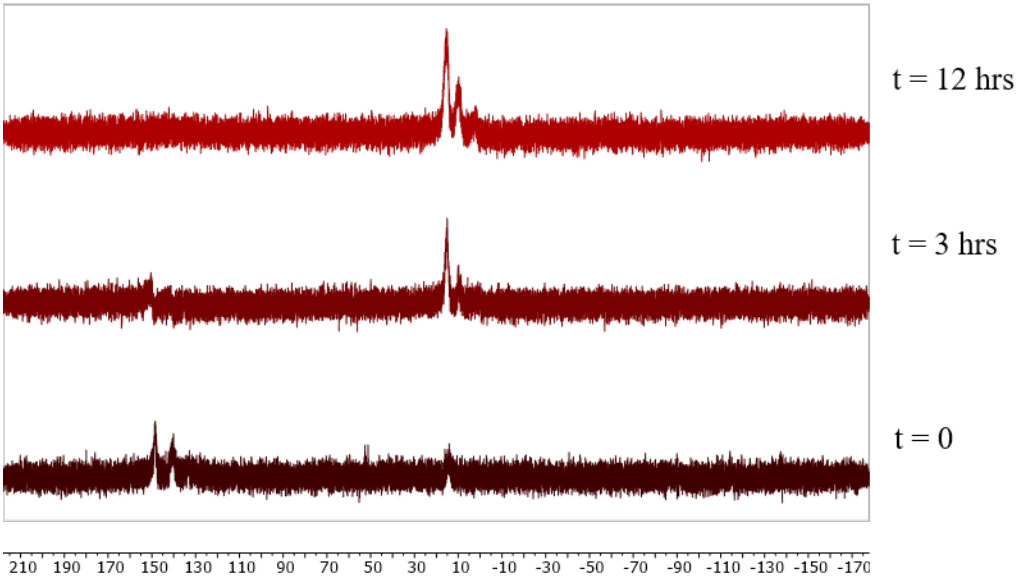

AB-PS-Het4

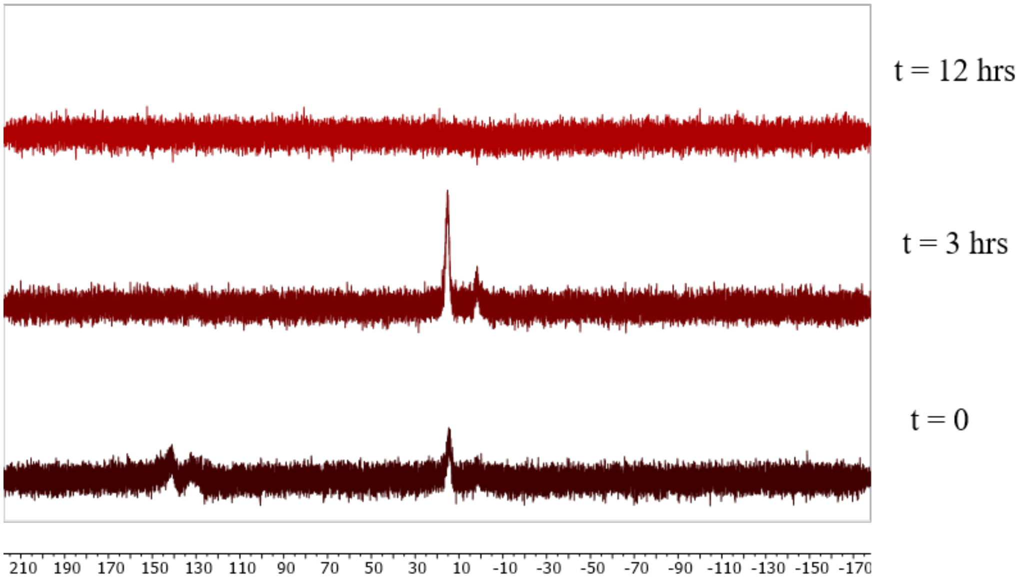

HypoGel-Het4

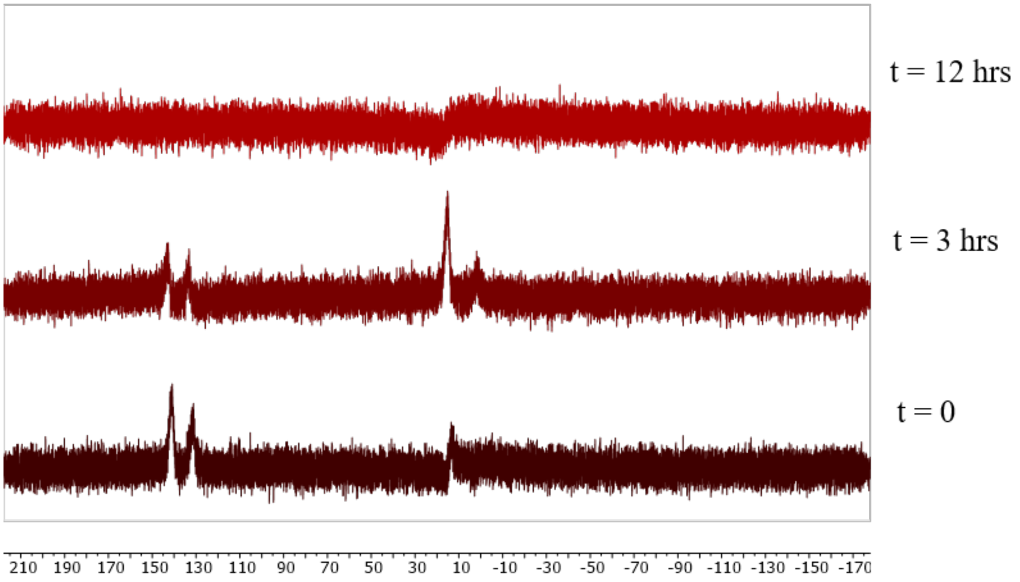

TG-XV-Het4

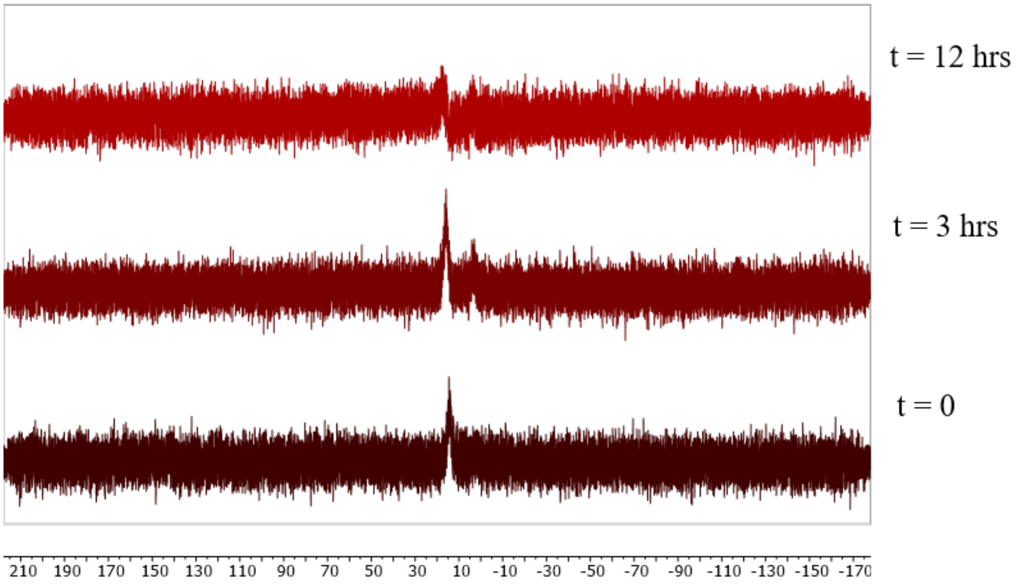

A modified NMR tube was loaded with **AM-PS-Het5.1** or **HypoGel-Het5.1** (50 mg). The resin was washed with CH_2Cl_2 (2 mL) and then loaded with with PCl (0.10 M, 0.20 mmol) and DIPEA (0.10 M, 0.20 mmol) in CH_2Cl_2 (2 mL) over 1 min. The resin was then washed with CH_2Cl_2 (2 mL) and ^{31}P Gel Phase NMR spectra were recorded over time.

HypoGel-Het5.1

Table S1. Summary of results for hygroscopicity study of **HypoGel-Het5.1**

Time / hours	0	1	3	5	7
Amount of P(III)-N	81%	68%	38%	18%	0%

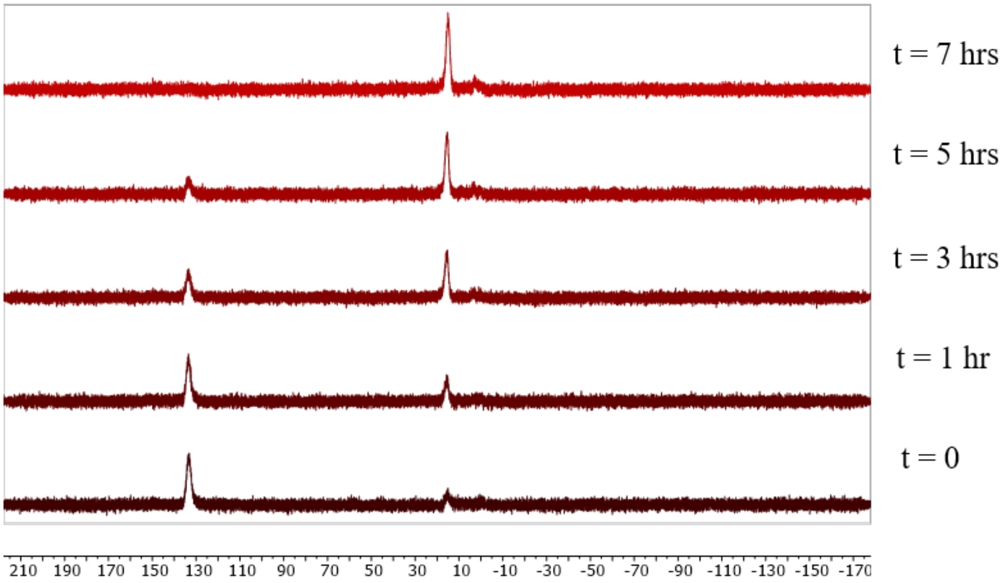

AM-PS-Het5.1

Table S2. Summary of results for hygroscopicity study of **AM-PS-Het5.1**

Time / hours	0	1	3	5	7
Amount of P(III)-N	94%	78%	49%	34%	24%

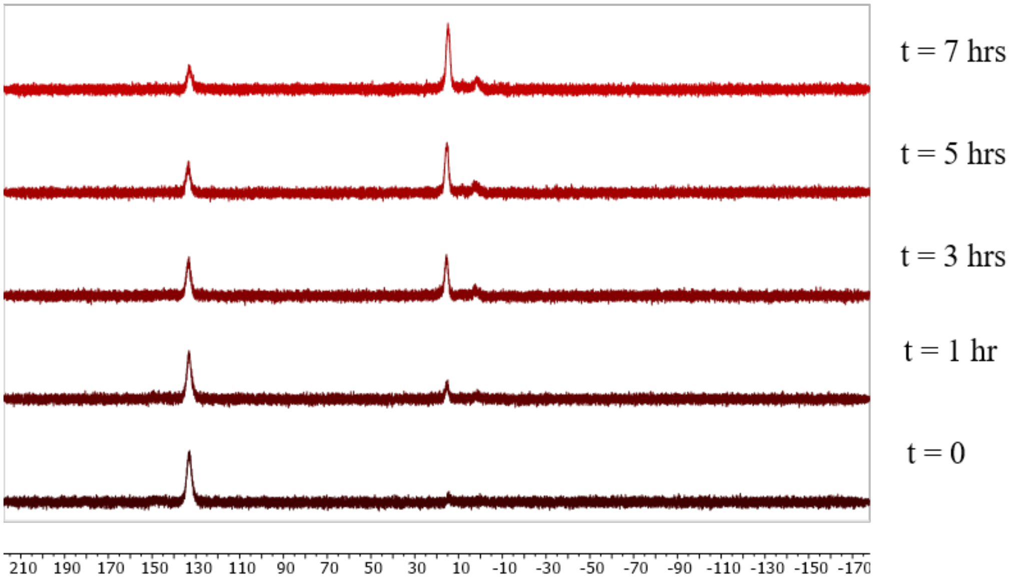

Overlay of Reference Spectra for ^{19}F NMR Analysis

Structures of 5’DMTr-floxuridine (**1**) and 3’-phosphoramidite-5’DMTr-floxuridine (**1-P**)

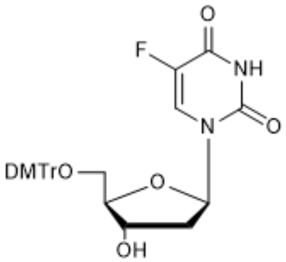

1

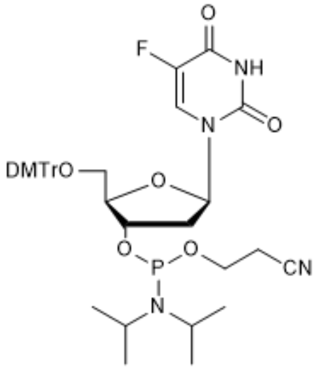

1-P

^{19}F NMR Spectrum 1: **1-P** in CH_2Cl_2
^{19}F NMR Spectrum 2: Mixture of **1-P** and **1** in CH_2Cl_2
^{19}F NMR Spectrum 3: **1** in CH_2Cl_2

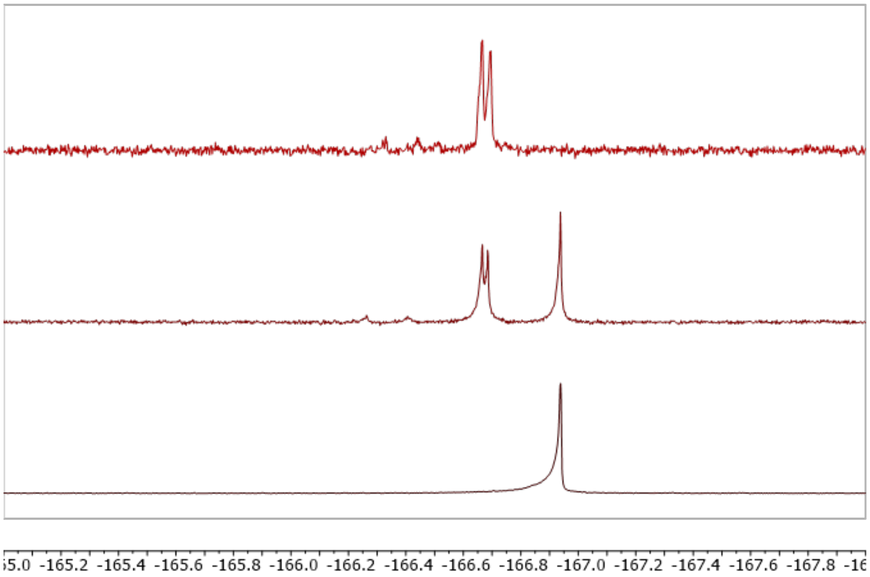

1-P

1-P and **1**

1

Base Screen

A modified NMR tube was loaded with **AM-PS-Het5.1** (50 mg, 0.50-0.75 mmol nitrotriazole). The resin was washed with CH_2Cl_2 (2 mL) and then loaded by elution of PCl (0.10 M, 0.20 mmol) and DIPEA (0.10 M, 0.02 mmol) in CH_2Cl_2 (2 mL) over 1 min. The resin was washed with CH_2Cl_2 (2 mL) before elution and collection of a solution of **1** (9 mg, 0.025 M, 0.025 mmol) with base (0.10 M, 0.10 mmol) in CH_2Cl_2 (1 mL) over 40 s. The eluate was directly analysed by ^{19}F NMR spectroscopy.

Table S3. Results of base screen

Base	^{19}F NMR Conversion* / %
-	13
9AJ	96
DABCO	27
DBU	18
DIPEA	12
DMAP	42
NMI	23
PPY	45
Proton Sponge	12
Pyridine	5
Quinoline	6
Triethylamine	15

*Based on product/starting material ratio.

Abbreviations: 9AJ (9-azajulolidine), DABCO (1,4-diazabicyclo[2.2.2]octane), DBU (1,8-diazabicyclo[5.4.0]undec-7-ene), DIPEA (*N*,*N*-diisopropylethylamine), DMAP (4-dimethylaminopyridine), NMI (*N*-methylimidazole), PPY (4-pyrrolidinopyridine).

No base

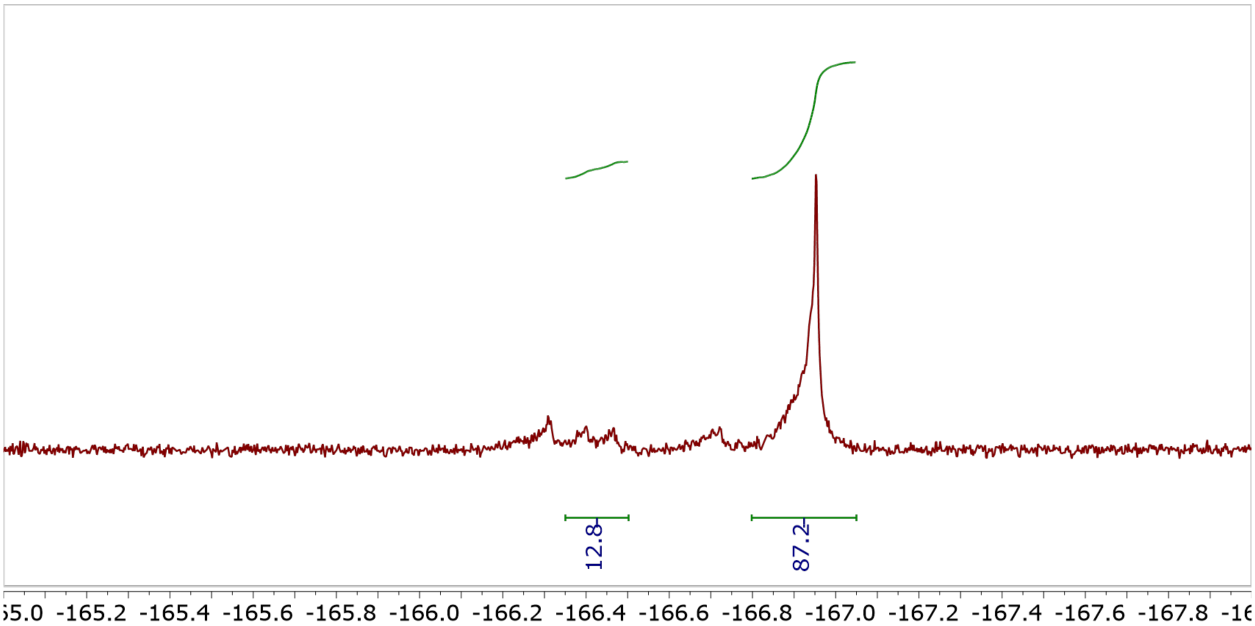
12.8
87.2
5.0 -165.2 -165.4 -165.6 -165.8 -166.0 -166.2 -166.4 -166.6 -166.8 -167.0 -167.2 -167.4 -167.6 -167.8 -16

9AJ

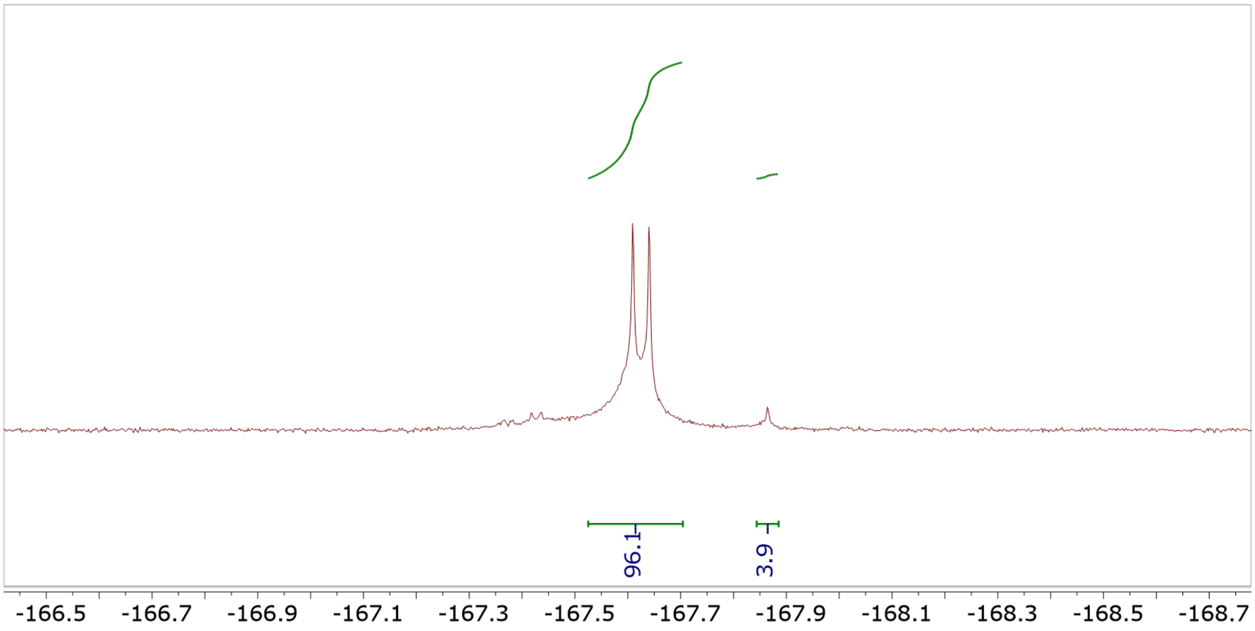
96.1
3.9
-166.5 -166.7 -166.9 -167.1 -167.3 -167.5 -167.7 -167.9 -168.1 -168.3 -168.5 -168.7

DABCO

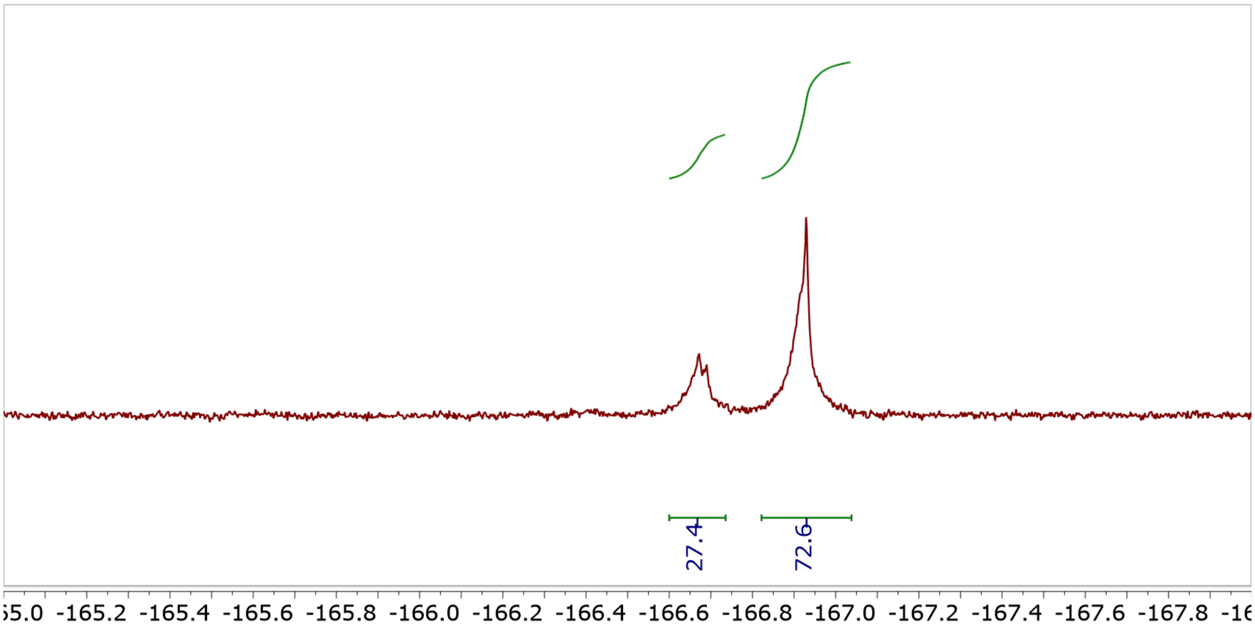
27.4
72.6
5.0 -165.2 -165.4 -165.6 -165.8 -166.0 -166.2 -166.4 -166.6 -166.8 -167.0 -167.2 -167.4 -167.6 -167.8 -16

DBU

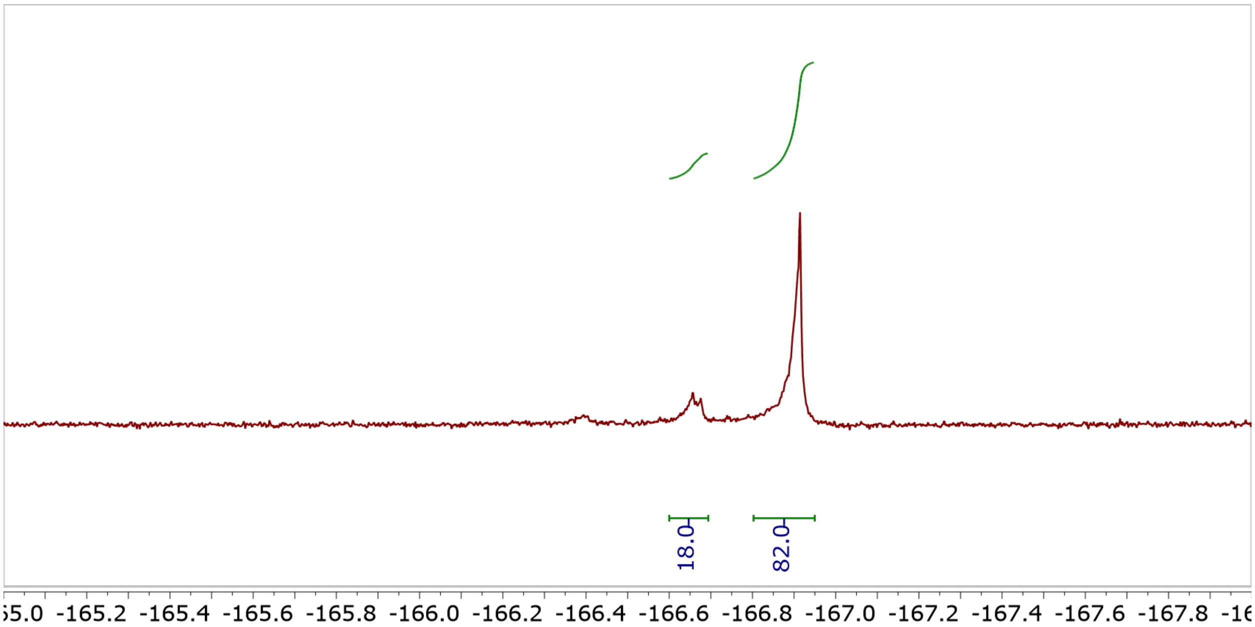
18.0
82.0
5.0 -165.2 -165.4 -165.6 -165.8 -166.0 -166.2 -166.4 -166.6 -166.8 -167.0 -167.2 -167.4 -167.6 -167.8 -16

DIPEA

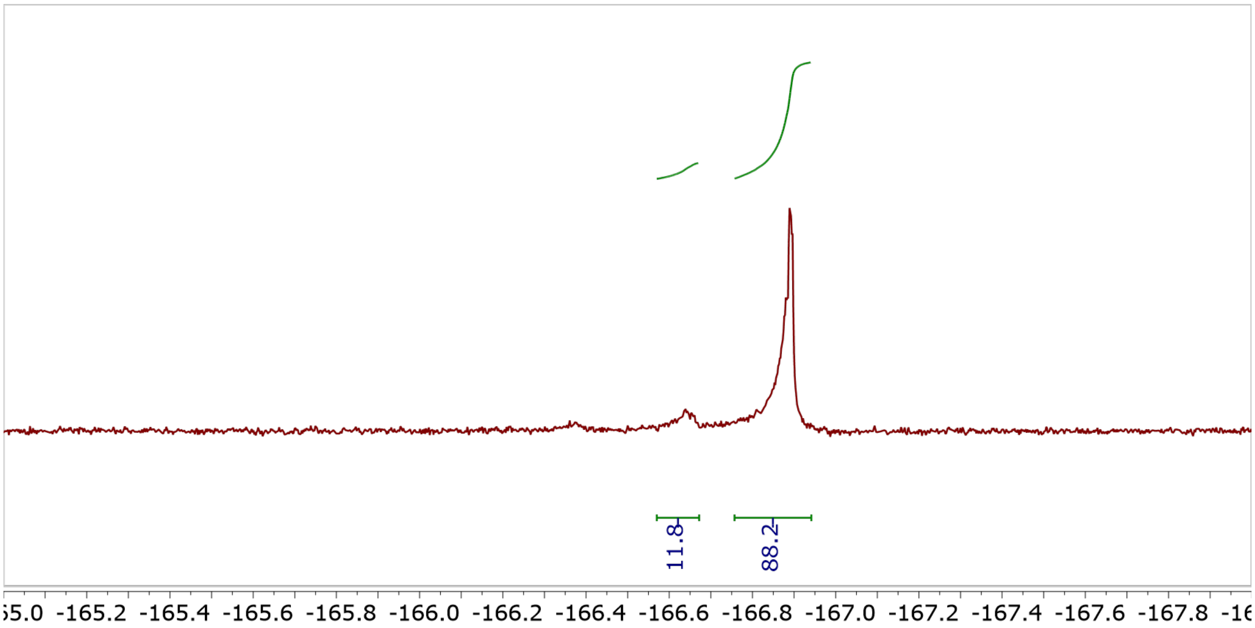

11.8
88.2
-165.0 -165.2 -165.4 -165.6 -165.8 -166.0 -166.2 -166.4 -166.6 -166.8 -167.0 -167.2 -167.4 -167.6 -167.8

DMAP

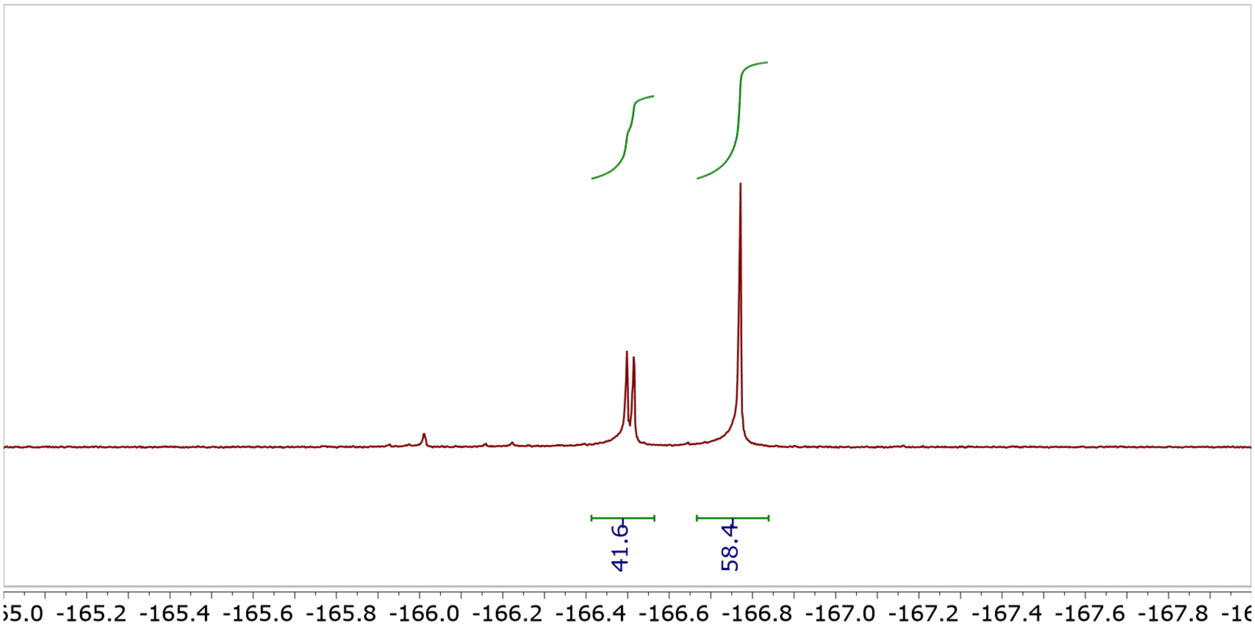

41.6
58.4
-165.0 -165.2 -165.4 -165.6 -165.8 -166.0 -166.2 -166.4 -166.6 -166.8 -167.0 -167.2 -167.4 -167.6 -167.8

NMI

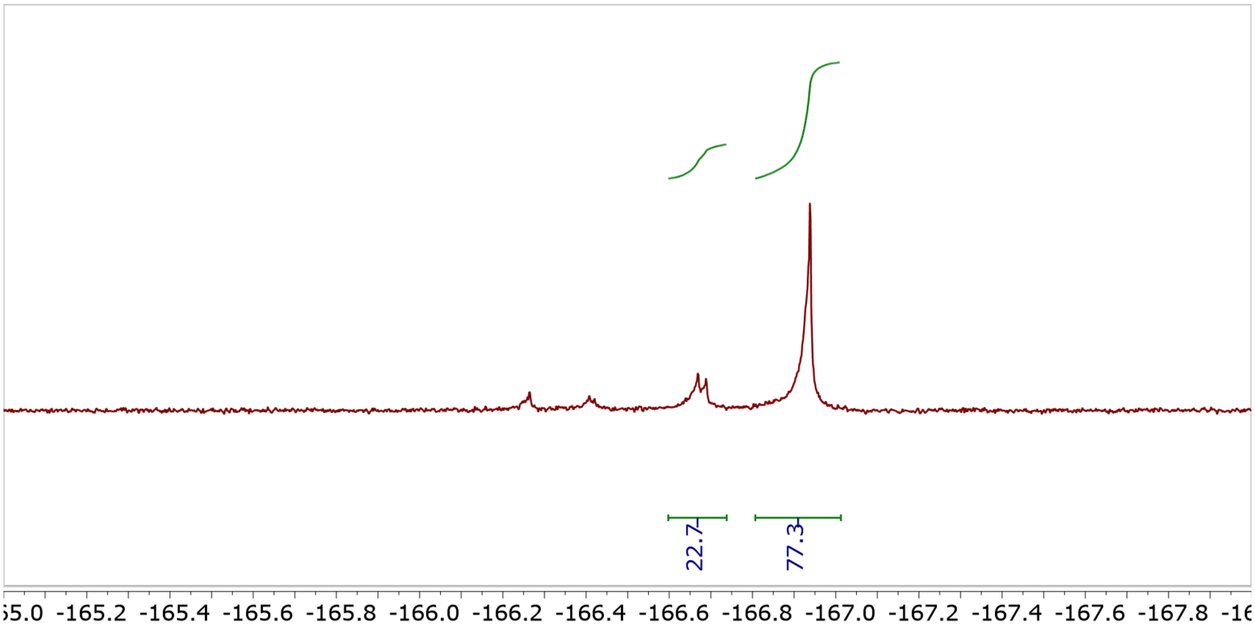
22.7
77.3
-165.2 -165.4 -165.6 -165.8 -166.0 -166.2 -166.4 -166.6 -166.8 -167.0 -167.2 -167.4 -167.6 -167.8

PPY

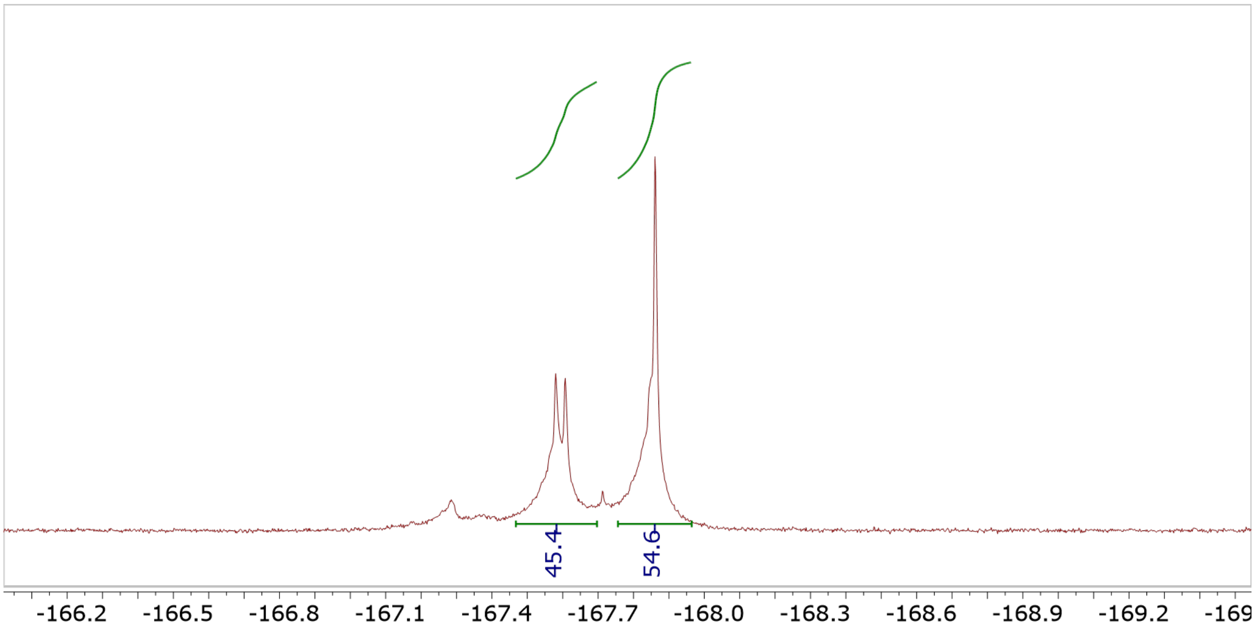
45.4
54.6
-166.2 -166.5 -166.8 -167.1 -167.4 -167.7 -168.0 -168.3 -168.6 -168.9 -169.2

Proton sponge

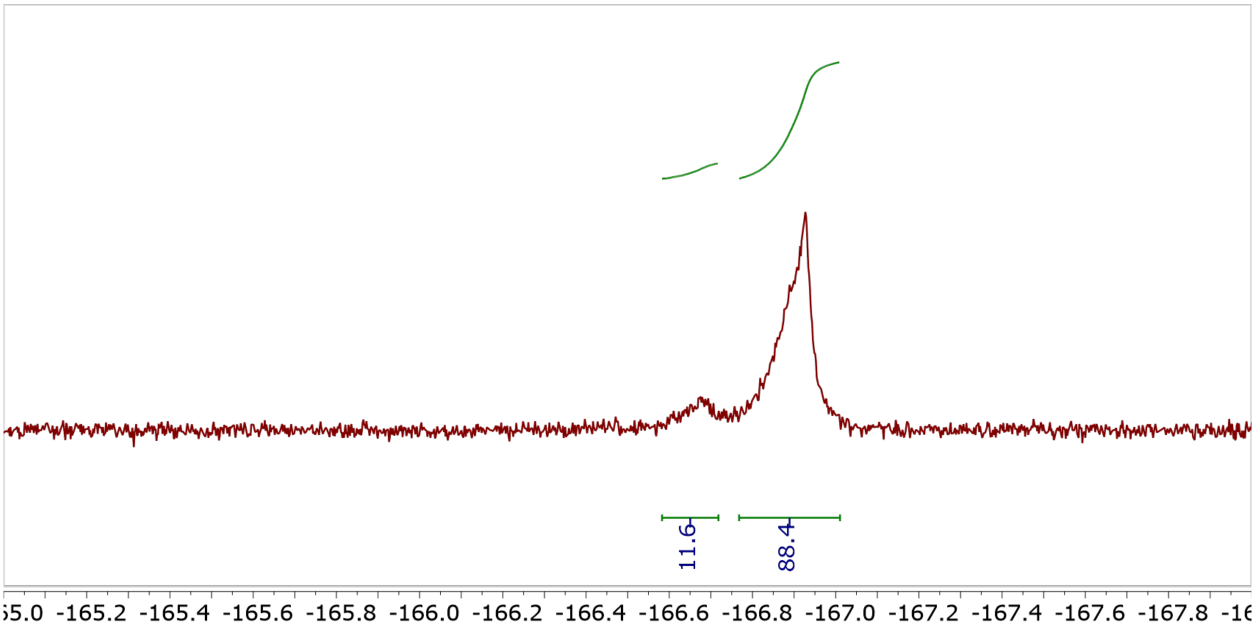

Pyridine

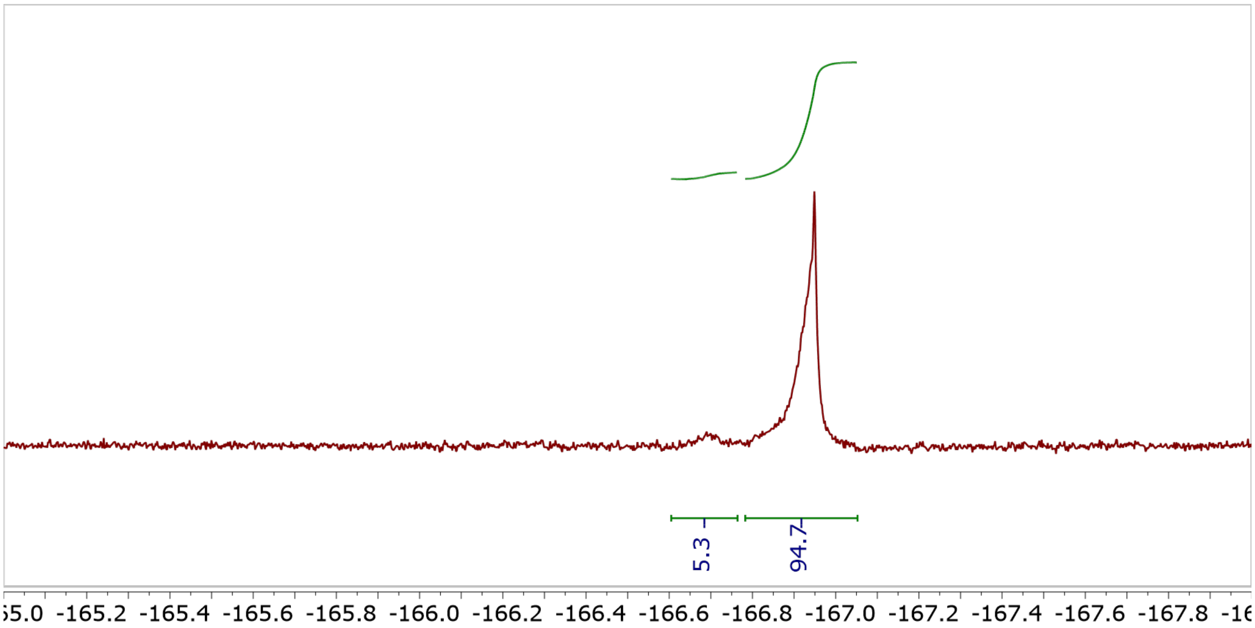

Quinoline

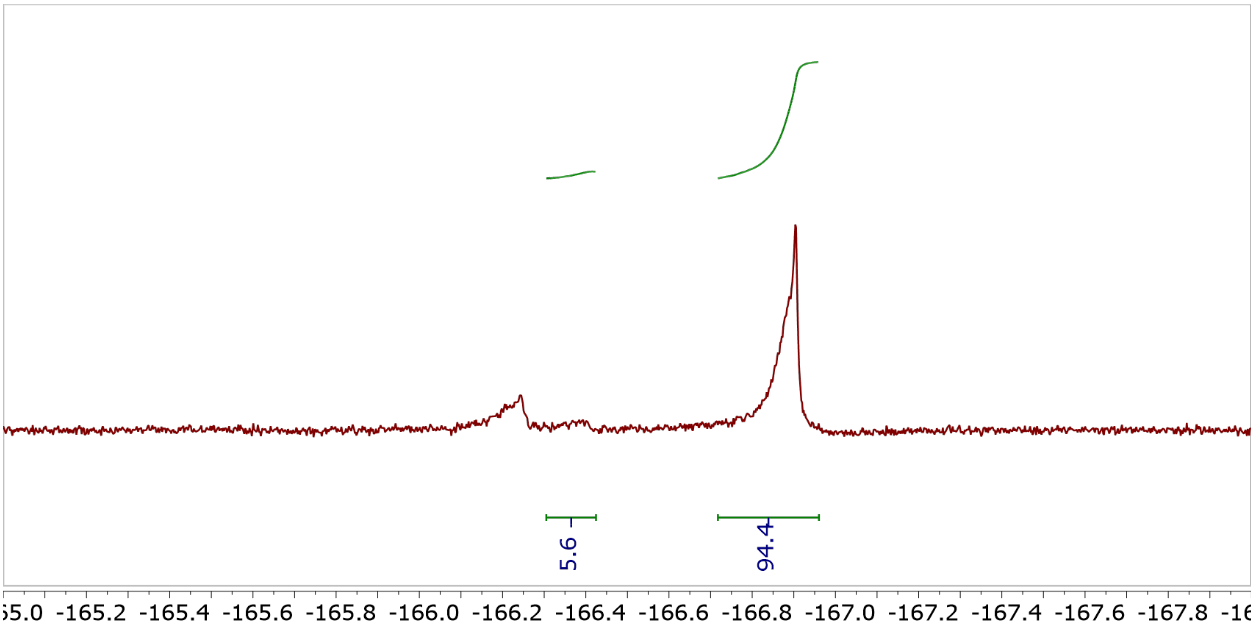

Triethylamine

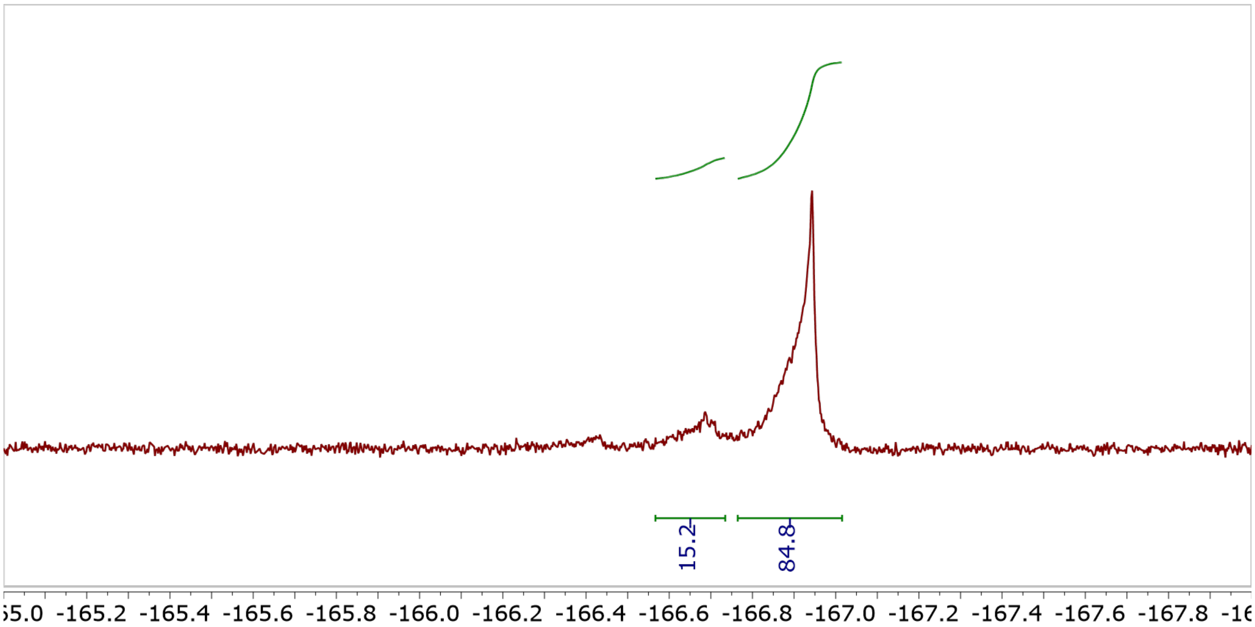

^{31}P NMR Study of Aminopyridine Intermediates

Formation of P(III)-Aminopyridine species

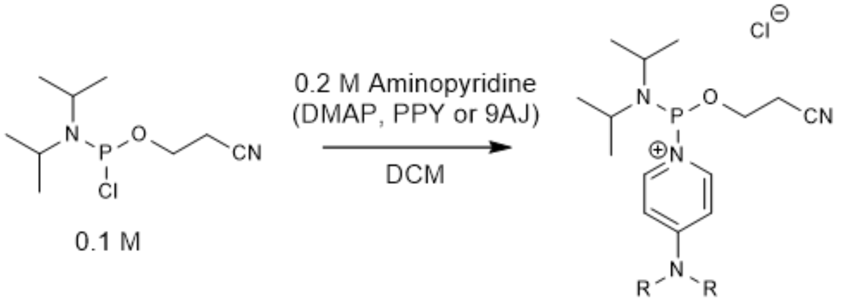

To a solution of PCl (0.5 mL, 0.1 M) dissolved in CH_2Cl_2 in an NMR tube was added either DMAP, PPY or 9AJ (0.5 mL, 0.2 M) in CH_2Cl_2. ^{31}P NMR spectra were then recorded.

DMAP
^{31}P NMR (162 MHz, CH_2Cl_2) δ_P (ppm) 164.7

PPY
^{31}P NMR (162 MHz, CH_2Cl_2) δ_P (ppm) 158.2

9AJ
^{31}P NMR (162 MHz, CH_2Cl_2) δ_P (ppm) 142.0

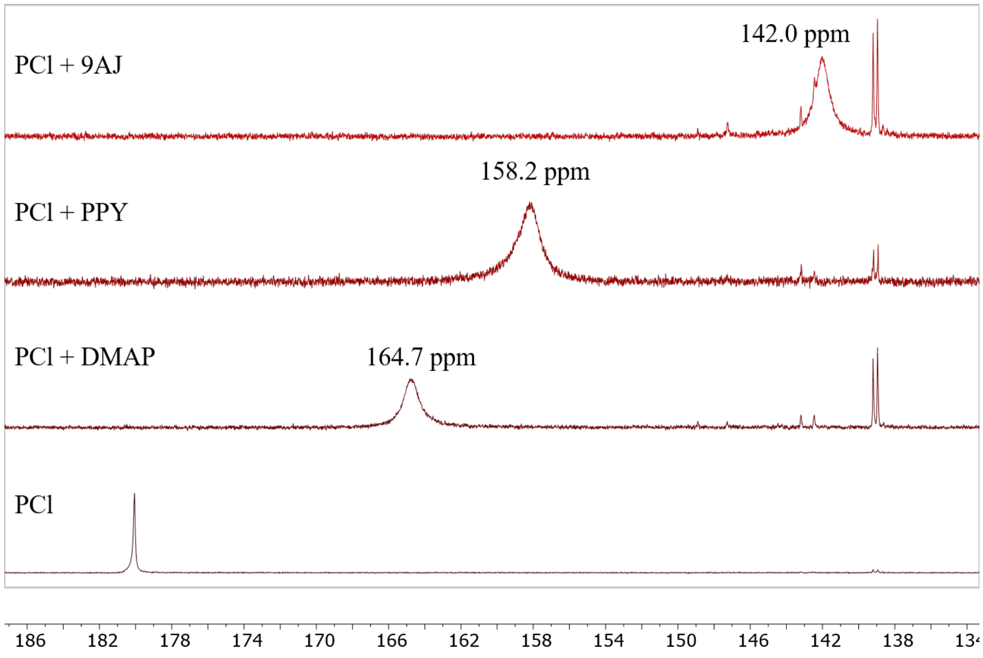

Competition with between 9AJ and already formed P(III)-nitrotriazole

A solution of PCl (0.1 M) and nitrotriazole (0.2 M) in CH_2Cl_2 (0.5 mL) was prepared and ^{31}P NMR spectra was recorded to confirm the formation of the P(III)-nitrotriazolide. Then a solution of 9AJ (0.2 M) in CH_2Cl_2 (0.5 mL) was added to the NMR tube and ^{31}P NMR spectra was recorded. However, no change was observed upon addition of 9AJ.

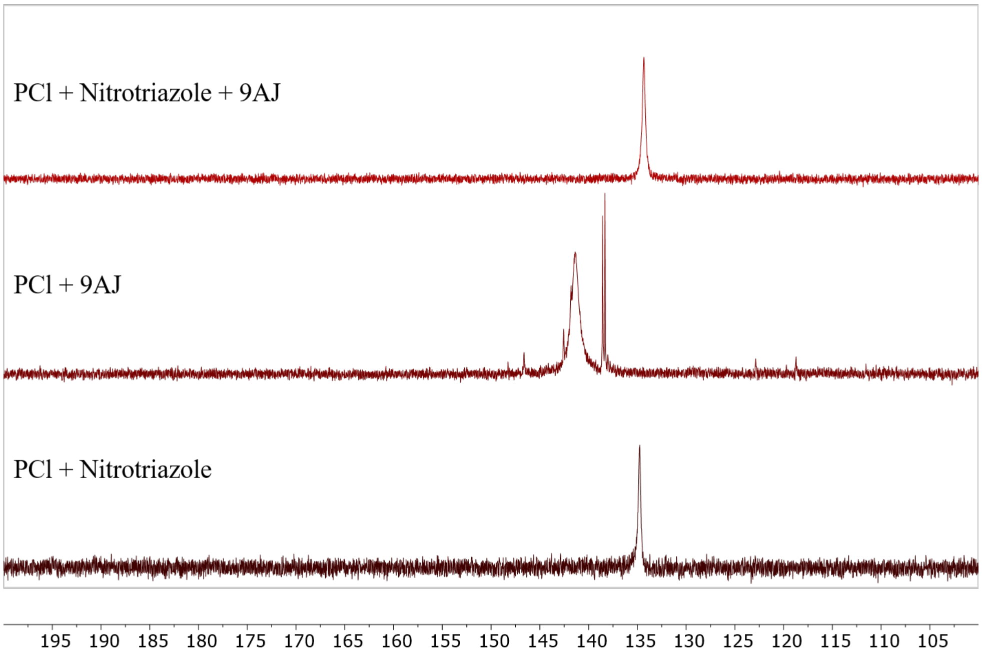

Flow System Setup

Flow System Description

The tubing throughout the system contained of stainless steel tubing (1/16'' OD x 0.75 mm ID) and connections were made with PEEK or stainless steel HPLC fittings (all with 1/16'' ID). A HPLC pump (Knauer Azura P 4.1S) was used to pump CH_2Cl_2 through the reactor system that consisted of one backpressure regulator (PBR, 100 psi), two injections valves (2 position: load and inject, 6-port, 1/16'', Vici) in series, and a packed bed reactor prepared with resin. The exiting fluid was collected in a roundbottomed flask under argon atmosphere, unless otherwise stated. To the two injection valves were connected two loops for loading of reagents: an alcohol loop (0.959 mL) and a PCl/DIPEA loop (2.42 mL).

Preparation of Packed Bed Reactor

An empty HPLC column of stainless steel (76 mm length, 4.6 mm ID) was filled with **AM-PS-Het5.1** resin (approx. 250 mg, 1.00-1.50 mmol/g, 0.25-0.375 mmol) and sealed.

Determination of void volume

The internal volume of a freshly prepared packed bed reactor or of an empty sample loop was determined by weighing the freshly prepared packed bed reactor or empty sample loop, m_1. Then solvent was pumped through the packed bed reactor or sample loop to fill the void with solvent before it was weighed again, m_2. For our determination we used CH_2Cl_2 and toluene as the two solvent. The reactor or sample loop void volume, V_{void}, was determined by:

$$V_{void} = \frac{m_2 - m_1}{\Delta\rho_{solvent}} = \frac{m_{CH_2Cl_2 + resin + column} - m_{Toluene + resin + column}}{\rho_{CH_2Cl_2} - \rho_{Toluene}} = \frac{43.550 \text{ g} - 43.140 \text{ g}}{1.33 \frac{\text{g}}{\text{mL}} - 0.867 \frac{\text{g}}{\text{mL}}}$$
$$= 0.890 \text{ mL}$$

The packed bed reactor with V_{Void} = 0.890 mL was used for all the following studies.

The flow system

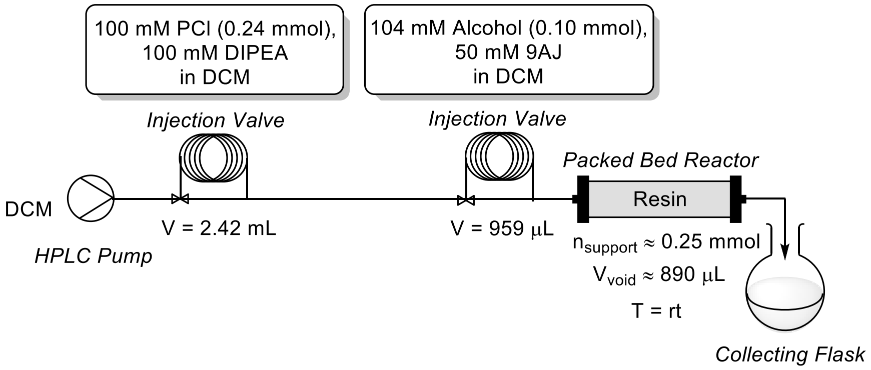

Figure S2 | Schematics of the flow setup. Figure by Martin B. Johansen

The total backpressure was usually in the range of 30-60 bar

Picture of the used flow system.

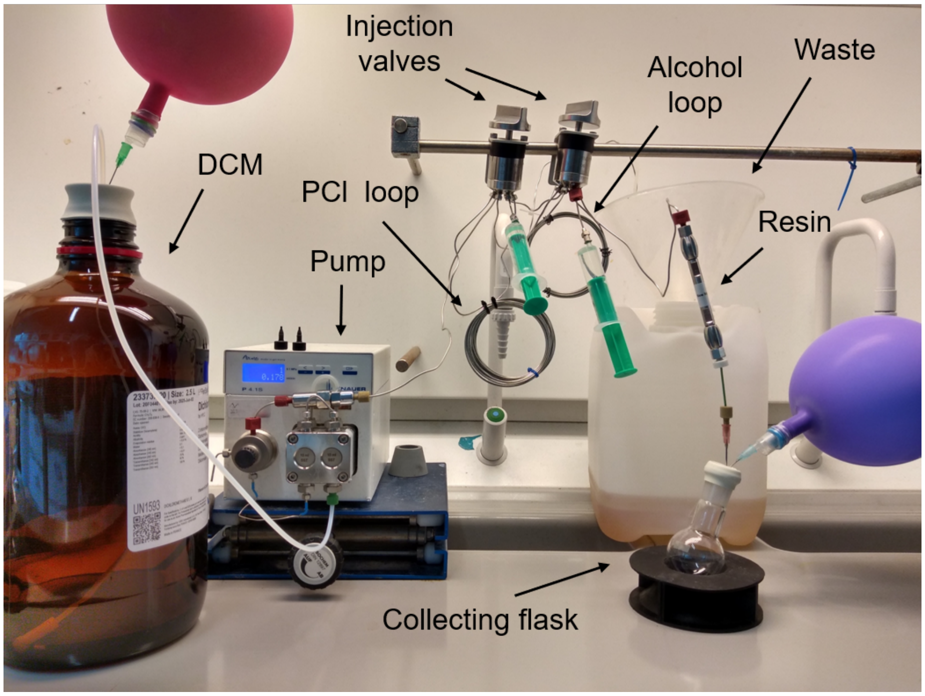

Figure S3 | Image of the flow setup in a fume hood. Figure by Alexander F. Sandahl

PCl stability

A solution of PCl (0.1 M) and DIPEA (0.1 M) in dry DCM was prepared and ^{31}P NMR spectra were recorded over time.

	Fresh sample	24 hrs	96 hrs
Amount of PCl remaining	95%	91%	83%

^{31}P NMR Spectra of the PCl solution over time

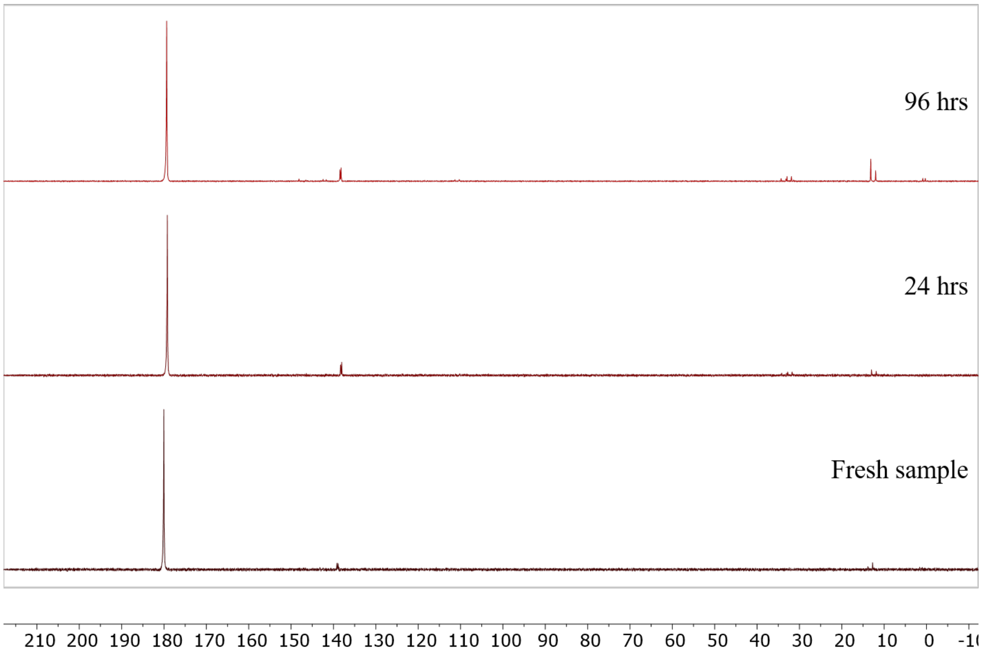

Diffusion Study

Using the PCl loop, the resin was loaded by elution of PCl (0.10 M, 0.24 mmol) and DIPEA (0.10 M, 0.24 mmol) in CH_2Cl_2 (2.4 mL) with flow rate of 0.89 mL/min (t_R = 1 min) for 15 mins. Using the Alcohol loop, 5'DMTr-thymidine (0.104 M, 0.10 mmol) and 9AJ (0.05 M, 0.045 mmol) dissolved in CH_2Cl_2 (0.890 mL) was eluted through the resin with different retention times (t_R = 1-3 mins). Up to 10 fractions were collected each corresponding to 1 void volume. The fractions were then subjected to TLC analysis (EtOAc/pentane = 2/1 + 1% Et_3N).

TLC analysis of the elution profile when eluting the alcohol through the resin.

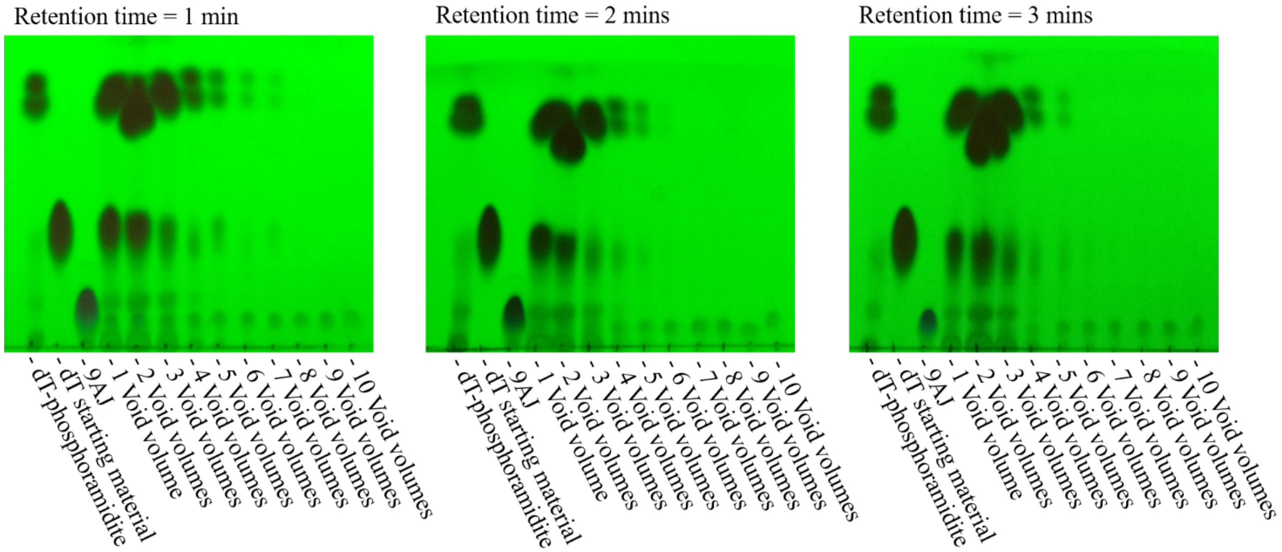

It was concluded that the compounds had eluted after 6 residence times giving a total collection volume of 5.34 mL.

Concentration Screen of 9AJ

The resin was loaded with PCl/DIPEA (flow rate = 1 V_{void}/min, 15 mins including washing step) and 5'DMTr-thymidine (0.10 mmol, 0.104 M, 0.96 mL) and 9AJ (varying concentration) was eluted through the resin (flow rate = 1 V_{void}/min, 6 mins) and the eluate was collected in a flask. The eluate was concentrated under reduced pressure and analysed by ^{1}H NMR to determine the reaction yield.

Table S4. Results of concentration screen

c(9AJ)	^{1}H NMR Yield* / %
5 mM	52
10 mM	60
50 mM	78
75 mM	80
100 mM	89

*Based on product/starting material ratio.

We decided to continue with a concentration of 50 mM 9AJ for the rest of the studies described.

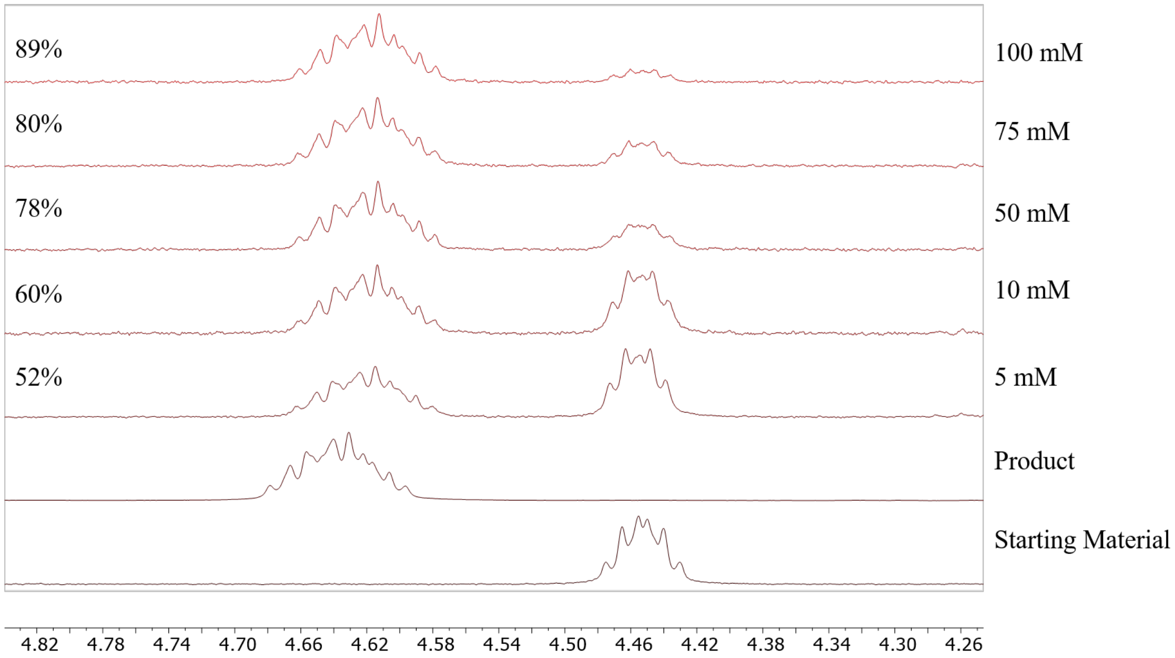

Synthesis of Starting Material Alcohols

2-dmf-2'-Deoxyguanisine

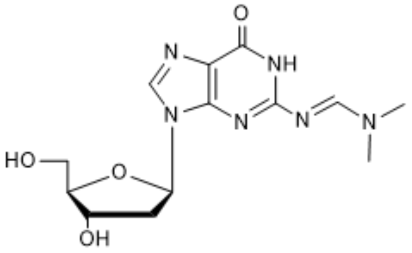

To a solution of 2′-deoxyguanosine (0.50 g, 1.87 mmol, 1.0 equiv.) in dry methanol (6 mL) was added *N*,*N*-dimethylformamide dimethyl acetal (1 mL, 7.48 mmol, 4.0 equiv.) over 5 mins. The resulting suspension was stirred at 55 °C for 16 hrs. The precipitate was collected by vacuum filtration, and the solid was washed with cold methanol to give the desired compound (520 mg, 1.61 mmol, 86%) as a white solid.

^{1}H NMR (400 MHz, DMSO) δ_H (ppm) 11.31 (s, 1H), 8.55 (s, 1H), 8.03 (s, 1H), 6.25 (dd, *J* = 7.81 Hz, 6.20 Hz, 1H), 5.29 (d, *J* = 3.91 Hz, 1H), 4.93 (t, *J* = 5.58 Hz, 1H), 4.39-4.35 (m, 1H), 3.85-3.81 (m, 1H), 3.61-3.47 (m, 2H), 3.16 (s, 3H), 3.03 (s, 3H), 2.63-2.55 (m, 1H), 2.23 (ddd, *J* = 9.03 Hz, 6.13 Hz, 2.87 Hz, 1H).

^{13}C NMR (101 MHz, DMSO) δ_C (ppm) 158.0, 157.6, 157.3, 149.6, 136.6, 119.7, 87.7, 82.8, 70.9, 61.8, 40.6, 34.6.

HRMS (ESI) *m/z* $[M+Na]^+$ calc. for $C_{13}H_{18}N_6O_4Na^+$ 345.1282, found 345.1289

2-dmf-5'-DMTr-2'-Deoxyguanisine (dmf-dG)

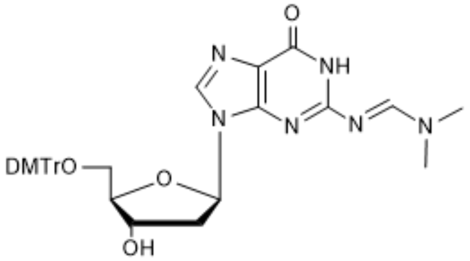

To a solution of 2-dmf-2'-deoxyguanisine (0.50 g, 1.55 mmol, 1.00 equiv.) in pyridine (10 mL) was added DMTrCl (0.630 g, 1.86 mmol, 1.30 equiv.) and the mixture was stirred overnight at rt. The volatiles were removed under reduced pressure and the residue subjected to flash column chromatography (0-5% MeOH in CH_2Cl_2 + 1% Et_3N) to give the desired product (0.57 g, 0.91 mmol, 59 %) as a white solid.

^{1}H NMR (400 MHz, CD_3CN) δ_H (ppm) 9.27 (s, 1H), 8.54 (s, 1H), 7.68 (s, 1H), 7.39 (d, *J* = 7.49 Hz, 2H), 7.28-7.21 (m, 8H), 6.83-6.76 (m, 4H), 6.28 (t, *J* = 6.51 Hz, 1H), 4.57-4.51 (m, 1H), 4.02-3.97 (m, 1H), 3.75 (s, 3H), 3.75 (s, 3H), 3.46 (d, *J* = 4.0 Hz, 1H), 3.24 (ddd, *J* = 16.70 Hz, 10.81 Hz, 5.87 Hz, 2H), 3.09 (s, 3H), 3.05 (s, 3H), 2.72 (dt, *J* = 13.22 Hz, 6.36 Hz, 1H), 2.37 (ddd, *J* = 11.40 Hz, 6.73 Hz, 4.66 Hz, 1H)

^{13}C NMR (101 MHz, CD_3CN) δ_C (ppm) 159.6, 159.1, 158.5, 158.2, 151.1, 146.1, 137.3, 136.8, 131.0, 130.9, 129.0, 128.7, 127.8, 121.3, 114.0, 87.1, 86.9, 84.3, 72.3, 65.1, 55.8, 41.5, 40.4, 35.2.

HRMS (ESI) *m/z* $[M+H]^+$ calc. for $C_{34}H_{36}N_6O_6H^+$ 625.2769, found 625.2780

Compound 1

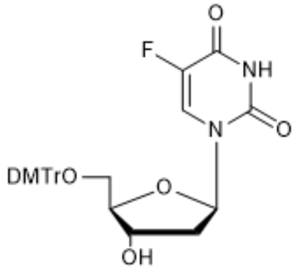

Floxuridine (750 mg, 3.05 mmol, 1.0 eq), DMTr-Cl (1.34 g, 3.96 mmol, 1.3 eq), and DMAP (74 mg, 0.61 mmol, 0.20 eq) were dissolved in pyridine (15 mL) and stirred overnight at rt The solvent was then removed under reduced pressure and the residue was redissolved in EtOAc (30 mL) and washed with water (30 mL) and brine (30 mL), dried over Na_2SO_4, filtered, and concentrated under reduced pressure. The residue was subjected to flash column chromatography (0-5% MeOH in CH_2Cl_2 + 1% Et_3N) to give the desired compound (1.256 g, 2.29 mmol, 75%) as a light pink foam.

^{1}H NMR (400 MHz, CD_3CN) δ_H (ppm) 9.27 (broad s, 1H), 7.74 (d, *J* = 6.77 Hz, 1H), 7.43 (d, *J* = 7.20 Hz, 2H), 7.36-7.29 (m, 6H), 7.24 (tt, *J* = 7.18, 2.02 Hz, 1H), 6.86 (dd, *J* = 7.64, 1.27 Hz, 4H), 6.14 (td, *J* = 6.45, 1.67 Hz, 1H), 4.47-4.42 (m, 1H), 3.94 (q, *J* = 4.16 Hz, 1H), 3.77 (s, 6H), 3.39 (broad s, 1H), 3.32 (dd, *J* = 10.74, 4.45 Hz, 1H), 3.24 (dd, *J* = 10.73, 2.97 Hz, 1H), 2.32-2.19 (m, 2H).

^{13}C NMR (100 MHz, CD_3CN) δ_C (ppm) 159.7, 158.1, 157.9, 149.9, 145.9, 142.7, 140.4, 136.8, 136.6, 131.0, 128.9, 127.9, 125.5, 125.2, 114.1, 87.5, 87.1, 86.1, 71.6, 64.3, 55.9, 41.1.

^{19}F NMR (376 MHz, CD_3CN) δ_F (ppm) -168.43.

HRMS (ESI) *m/z* $[M+Na]^+$ calc. for $C_{30}H_{29}FN_2O_7Na$ 571.1851, found 571.1852.

Compound 4

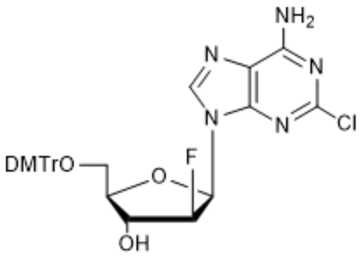

Clofarabine (500 mg, 1.65 mmol, 1.0 eq), DMTr-Cl (642 mg, 1.89 mmol, 1.15 eq) and DMAP (40 mg, 0.33 mmol, 0.20 eq) were dissolved in pyridine (10 mL) and stirred overnight at rt The solvent was then removed under reduced pressure and the residue was redissolved in EtOAc (25 mL) and washed with water (25 mL) and brine (25 mL), dried over Na_2SO_4, filtered and concentrated under reduced pressure. The residue was subjected to flash column chromatography (1/2 - 1/0 EtOAc/pentane + 1% Et_3N) to give the desired compound (852 mg, 1.40 mmol, 85%) as a white foam.

^{1}H NMR (400 MHz, CD_3CN) δ_H (ppm) 7.99 (d, *J* = 2.20 Hz, 1H), 7.44 (d, *J* = 7.35 Hz, 2H), 7.35-7.18 (m, 7H), 6.85-6.79 (m, 4H), 6.50 (broad s, 2H), 6.35 (dd, *J* = 15.23, 4.36 Hz, 1H), 5.14 (dt, *J* = 52.23, 4.02 Hz, 1H), 4.55 (d, *J* = 18.56 Hz, 1H), 4.11-4.04 (m, 2H), 3.74 (d, *J* = 1.04 Hz, 6H), 3.44 (dd, *J* = 10.40, 6.45 Hz, 1H), 3.34 (dd, *J* = 10.42, 3.55 Hz, 1H).

^{13}C NMR (100 MHz, CD_3CN) δ_C (ppm) 159.6, 157.7, 154.8, 151.8, 146.0, 141.4, 141.3, 136.8, 136.8, 131.0, 131.0, 129.0, 128.8, 127.8, 119.0, 114.0, 97.3, 95.3, 87.1, 83.5, 83.4, 83.3, 83.1, 75.2, 74.9, 64.2, 55.9.

^{19}F NMR (376 MHz, CD_3CN) δ_F (ppm) -199.04.

HRMS (ESI) *m/z* $[M+H]^+$ calc. for $C_{31}H_{30}ClFN_5O_5$ 606.1914, found 606.1923.

Compound 4.1

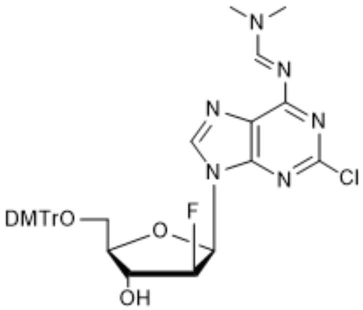

Compound **4** (780 mg, 1.29 mmol, 1.0 eq) was dissolved in dry MeOH (20 mL) and *N*,*N*-dimethylformamide dimethyl acetal (0.857 mL, 6.44 mmol, 5.0 eq) was added and the mixture was stirred overnight at rt. The mixture was then diluted with EtOAc (50 mL) and washed 5 times with water (50 mL), brine (50 mL), dried over Na_2SO_4, filtered and concentrated under reduced pressure to give the desired compound (851 mg, 1.29 mmol, quant.) as a white foam.

^{1}H NMR (400 MHz, CD$_3$CN) δ_H (ppm) 8.87 (s, 1H), 8.03 (d, *J* = 2.32 Hz, 1H), 7.43 (d, *J* = 7.11 Hz, 2H), 7.35-7.19 (m, 7H), 6.85-6.80 (m, 4H), 6.38 (dd, *J* = 15.42, 4.37 Hz, 1H), 5.16 (dt, *J* = 52.07 Hz 4.28 Hz, 1H), 4.57 (d, *J* = 18.55 Hz, 1H), 4.12-4.06 (m, 2H), 3.75 (d, *J* = 1.60 Hz, 6H), 3.42 (dd, *J* = 10.43, 6.42 Hz, 1H) 3.33 (dd, *J* = 10.87, 3.53 Hz, 1H), 3.19 (s, 3H), 3.16 (s, 3H).

^{13}C NMR (100 MHz, CD$_3$CN) δ_C (ppm) 161.7, 159.7, 159.7, 154.3, 153.7, 146.0, 142.3, 142.3, 136.8, 136.8, 131.0, 131.0, 129.0, 128.8, 127.9, 125.5, 114.0, 97.3, 95.4, 87.1, 83.5, 83.5, 83.2, 83.1, 75.2, 75.0, 64.2, 55.9, 41.8, 35.5.

^{19}F NMR (376 MHz, CD$_3$CN) δ_F (ppm) -199.0.

HRMS (ESI) *m/z* $[M+H]^+$ calc. for $C_{34}H_{35}ClFN_6O_5$ 661.2336, found 661.2339.

Compound 5

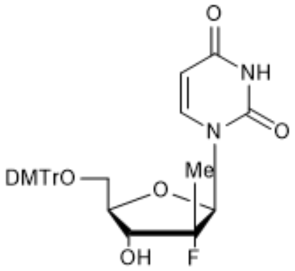

(2'*R*)-2'-Deoxy-2'-fluoro-2'-methyluridine (300 mg, 1.15 mmol, 1.0 eq), DMTrCl (508 mg, 1.50 mmol, 1.3 eq) and DMAP (28 mg, 0.23 mmol, 0.20 eq) were dissolved in pyridine (7 mL) and stirred overnight at rt The solvent was then removed under reduced pressure and the residue was redissolved in EtOAc (25 mL) and washed with water (25 mL) and brine (25 mL), dried over Na_2SO_4, filtered and concentrated under reduced pressure. The residue was subjected to flash column chromatography (1/2 - 1/0 EtOAc/pentane + 1% Et_3N) to give the desired compound (444 mg, 0.80 mmol, 69%) as a white foam.

^{1}H NMR (400 MHz, CD$_3$CN) δ_H (ppm) 9.12 (s, 1H), 7.86 (d, *J* = 8.15 Hz, 1H), 7.44 (d, *J* = 7.23 Hz, 2H), 7.36-7.25 (m, 7H), 6.90 (d, *J* = 8.73 Hz, 4H), 6.05 (d, *J* = 18.69 Hz, 1H), 5.12 (d, *J* = 8.17 Hz, 1H), 4.17 (dt, *J* = 24.07, 9.17 Hz, 1H), 4.01 (d, *J* = 9.68 Hz, 1H), 3.77 (s, 6H), 3.62 (d, *J* = 8.38 Hz, 1H), 3.48-343 (m, 2H), 1.37 (d, *J* = 10.82 Hz, 3H).

^{13}C NMR (100 MHz, CD$_3$CN) δ_C (ppm) 163.8, 159.8, 159.8, 151.5, 145.8, 136.6, 136.3, 131.2, 131.1, 129.1, 129.0, 128.1, 114.2, 114.2, 103.0, 102.8, 101.0, 87.7, 81.5, 72.8, 72.6, 61.8, 55.9, 17.0, 16.8.

^{19}F NMR (376 MHz, CD$_3$CN) δ_F (ppm) -162.34.

HRMS (ESI) *m/z* $[M+H]^+$ calc. for $C_{31}H_{32}FN_2O_7$, 563.2188, found 563.2189.

Compound 6

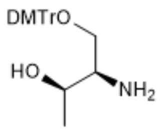

Compound **6** was prepared according to previously published procedures and data are consistent with reported values[4]

Compound 6.1

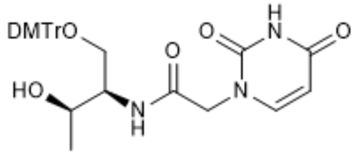

To a stirred solution of uracil acetic acid (2.29 g, 13.5 mmol, 1 equiv.), **6** (6.06 g, 14.9 mmol, 1.1 equiv.) and DIPEA (4.8 mL, 27.6 mmol, 2 equiv.) in dry DMF (48 mL) was added HBTU (7.69 g, 20.3 mmol, 1.5 equiv.) under argon atmosphere. The reaction stirred at rt overnight. The reaction mixture was diluted with EtOAc and washed with water followed by drying over Na_2SO_4 where after the solvent was evaporated *in vacuo*. The compound was purified by flash chromatography (0 – 3% MeOH in CH_2Cl_2+ 1% Et_3N) to yield a white foam (4.50 g, 8.04 mmol, 60%).

^{1}H NMR (400 MHz, *d6*-DMSO) δ_H (ppm) 11.27 (s, 1H), 8.00 – 7.97 (d, *J* = 9.2 Hz, 1H), 7.53 – 7.51 (d, *J* = 8.0 Hz, 1H), 7.39 – 7.19 (m, 9H), 6.90 – 6.87 (m, 4H), 5.56 – 5.53 (dd, *J* = 7.6, 2.0 Hz, 1H), 4.62 – 4.61 (d, *J* = 4.4 Hz, 1H), 4.41 (s, 2H), 3.94 – 3.87 (m, 2H), 3.73 (s, 6H), 2.88 – 2.85 (m, 1H), 0.96 – 0.95 (d, *J* = 6.4 Hz, 3H)

^{13}C NMR (100 MHz, *d6*-DMSO) δ_C (ppm) 170.8, 167.4, 164.4, 158.4, 151.4, 147.3, 145.5, 136.2, 130.2, 128.2, 127.0, 113.6, 100.7, 85.6, 65.3, 63.2, 60.2, 55.5, 54.7, 49.9, 46.2, 21.2, 20.7, 14.6, 9.1

HRMS (ESI) *m/z* $[M+Na]^+$ calc. for $C_{31}H_{33}N_3O_7Na$ 582.2216, found 582.2211.

Compound 8

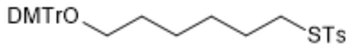

1,6-Hexanediol (17.4 g, 148 mmol, 10 eq) and triethylamine (2.26 mL, 16.2 mmol, 1.1 eq) were dissolved in THF (100 mL) and DMTr-Cl (5.00 g, 14.8 mmol, 1.0 eq) was added. The mixture was stirred at rt overnight. Et_2O (200 mL) was added and the organic phase was washed 3 times with water (100 mL) and once with brine (100 mL), dried over Na_2SO_4, filtered and concentrated under reduced pressure. The residue was dissolved in CH_2Cl_2 (50 mL) and cooled in an ice bath. *N*-Methyl morpholine (5.67 mL, 51.6 mmol, 3.5 eq) was added along with MsCl (1.37 mL, 17.7 mmol, 1.2 eq) and the mixture was stirred at 0 °C for 30 mins whereafter the reaction mixture

was allowed to warm to rt and stirred overnight. The reaction was quenched with water (50 mL) and the phases were separated. The organic phase was washed twice with water (50 mL), once with brine (50 mL) and then dried over Na_2SO_4, filtered and concentrated under reduced pressure. The residue was dissolved in MeCN (50 mL) along with potassium *p*-toluenethiosulphonate (4.12 g, 18.2 mmol, 1.23 eq) and the mixture was stirred at 75 °C overnight. The mixture was then diluted with Et_2O and washed with water and brine, dried over Na_2SO_4, filtered and concentrated under reduced pressure. The residue was purified with flash column chromatography (3/1 – 0/1 pentane/ CH_2Cl_2 gradient + 1% Et_3N) to give the desired compound (5.507 g, 9.32 mmol, 63% for 3 steps) as a clear oil.

^{1}H NMR (400 MHz, $CDCl_3$) δ_H (ppm) 7.77 (d, *J* = 8.29 Hz, 2H), 7.40 (d, *J* = 7.20 Hz, 2H), 7.32-7.23 (m, 8H), 7.18 (t, *J* = 7.20 Hz, 1H), 6.82 (d, *J* = 8.84 Hz, 4H), 3.77 (s, 6H), 2.99 (t, *J* = 6.43 Hz, 2H), 2.94 (t, *J* = 7.38 Hz, 2H), 2.41 (s, 3H), 1.59-1.48 (m, 4H), 1.33-1.19 (m, 4H).

^{13}C NMR (100 MHz, $CDCl_3$) δ_C (ppm) 158.5, 145.5, 144.7, 142.3, 136.8, 130.1, 129.9, 128.3, 127.8, 127.1, 126.7, 113.1, 85.8, 63.2, 55.3, 36.1, 29.9, 28.7, 28.5, 25.8, 21.7.

HRMS (ESI) *m/z* $[M-K]^+$ calc. for $C_{34}H_{38}O_5S_2K$ 629.1792, found 629.1785.

Compound 8.1

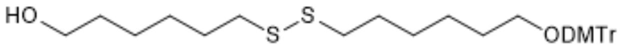

Compound **8** (484 mg, 819 µmol, 1.1 eq) was dissolved in CH_2Cl_2 (10 mL) and triethylamine (156 µL, 1.12 mmol, 1.5 eq) was added. 6-Mercapto-1-hexanol (102 µL, 745 µmol, 1.0 eq) was added and the mixture was stirred for 30 mins at rt. The solvent was then removed under reduced pressure and the residue was subjected to flash column chromatography (1/3 – 1/0 Et_2O/pentane + 1% Et_3N) to give the desired compound (396 mg, 693 µmol, 93%) as a clear oil.

^{1}H NMR (400 MHz, CD_3CN) δ_H (ppm) 7.42 (d, *J* = 7.38 Hz, 2H), 7.32-7.26 (m, 6H), 7.21 (t, *J* = 7.21 Hz, 1H), 6.86 (d, *J* = 8.86 Hz, 4H), 3.76 (s, 6H), 3.45 (q, *J* = 5.50 Hz, 2H), 3.00 (t, *J* = 6.46 Hz, 2H), 2.68 (q, *J* = 7.45 Hz, 4H), 2.46 (t, *J* = 5.33 Hz, 1H), 1.69-1.54 (m, 6H), 1.50-1.43 (m, 2H), 1.40-1.27 (m, 8H).

^{13}C NMR (100 MHz, CD_3CN) δ_C (ppm) 159.5, 146.7, 137.6, 130.9, 129.0, 128.7, 127.6, 113.9, 86.5, 64.0, 62.5, 55.9, 39.5, 33.5, 30.5, 29.9, 29.8, 29.0, 28.8, 26.6, 26.2.

HRMS (ESI) *m/z* $[M+Na]^+$ calc. for $C_{33}H_{44}O_4S_2Na$ 591.2573, found 591.2570.

Compound 9

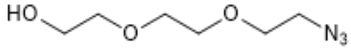

Potassium hydroxide (1.77 g, 31.47 mmol, 4.00 eq.) was added portionwise to a cooled (0 °C) solution of triethylene glycol (21.5 mL, 157 mmol, 20.0 eq.) and p-toluenesulfonyl chloride (1.50 g, 7.87 mmol, 1.00 eq.) in CH_2Cl_2 (25 mL). The reaction mixture was stirred for 3 hrs at 0 °C. Water (40 mL) was then added and the aqueous phase was extracted with CH_2Cl_2 (2 × 25 mL) and the organic phase was washed with water (40 mL) and brine (30 mL), dried over Na_2SO_4 and concentrated under reduced pressure to afford a yellow oil. Without further purification, the resulting residue was dissolved in 15 mL *N*,*N*-dimethylformamide and sodium azide (0.72 g, 11.0 mmol, 1.4 equiv.) was added. The resulting mixture was stirred at 80 °C for 16 hours. The solvent was removed under reduced pressure and the residue was subjected to flash column chromatography (0-5% MeOH in CH_2Cl_2) to give the desired product (1.03 g, 5.71 mmol, 73 %) as a colorless oil.

^{1}H NMR (400 MHz, CD_3CN) δ_H (ppm) 3.66-3.55 (m, 8H), 3.49 (t, *J* = 5.11 Hz, 2H), 3.37 (t, *J* = 4.86 Hz, 2H), 2.78 (s, broad, 1H)

^{13}C NMR (101 MHz, CD_3CN) δ_C (ppm) 73.3, 71.1, 71.0, 70.5, 61.9, 51.5

HRMS (ESI) *m/z* $[M+H]^+$ calc. for $C_6H_{13}N_3O_3H^+$ 176.1030, found 176.1033

Residence Time Study

The synthetic cycle used for screening of residence times is described in Table S5. The eluate was collected for 6 residence times and concentrated under reduced pressure. The crude mixtures were analysed ^{1}H and ^{19}F NMR (if possible) spectroscopy. The yields were calculated by ^{1}H NMR or ^{19}F NMR (if possible) integration of non-overlapping signals between starting material and product. If not commercially available, the products were synthesised according to the procedure included here (S79-S83). Results are given in Table S6. It should be noted that for compound **2** the integration from $^{13}C\underline{H}D_2CN$ overlaps with the starting material giving 0.55% additional integral of the residual solvent peak. This has been subtracted. Furthermore, compound **6** produces the product in 1:2 mixture of the 2 phosphoramidite diastereomers when eluted through the P(III)-loaded resin. When the reference product was synthesized by conventional batch method another ratio was isolated of the diastereomers giving a difference between the stacked NMR spectra.

Table S5. Summary of synthetic cycle.

Cycle step	Reagent(s)	Injection volume	Residence time (flow rate)	Time
Loading	0.1 M PCl, 0.1 M DIPEA in CH_2Cl_2	2.4 mL	1 min (0.89 mL/min)	10 mins
Wash	CH_2Cl_2		1 min (0.89 mL/min)	5 mins
Transfer	0.104 M Alcohol (0.10 mmol), 0.050 M 9AJ in CH_2Cl_2	0.96 mL	Substrate dependent	6 residence times

Below is depicted the proposed reasoning for effect observed by changing protecting group of guanosine. Isobutyryl protection allows internal hydrogen bond to 5'-OH thus changing the ribose conformation to 3'-endo giving an equatorial 3'-OH. Without the hydrogen bond donor the nucleobase would have an anti orientation thus giving a 2'-endo conformation of the ribose and an axial and less nucleophilic 3'-OH.

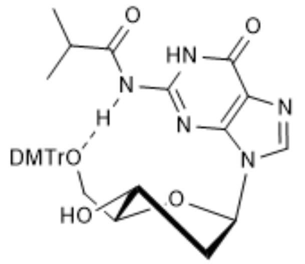

Equatorial 3'-OH

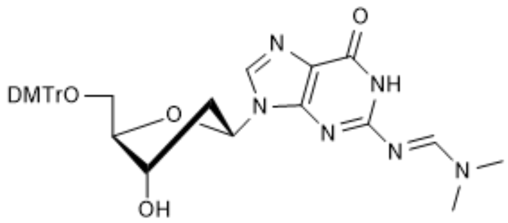

Axial 3'-OH

Table S6. Summary of results from residence time study given as distributions between phosphoramidite product and starting material. Values ≥ 98% have been marked light green.

Alcohol	Residence time						^{31}P NMR purity*	^{31}P NMR purity**
	1 min	2 mins	3 mins	4 mins	5 mins	6 mins		
Bz-dA	78%	95%	99%	100%			81.0%	96.9%
dT	76%	81%	88%	98%	100%		88.9%	94.9%
iBu-dG	100%						72.1%	93.5%
dmf-dG	69%	90%	96%	100%			72.6%	81.2%
Bz-dC	72%	88%	92%	97%	97%	98%	93.3%	97.6%
**1**	90%	99%	100%				93.3%	98.0%
**2**	74%	90%	94%	>99%			90.2%	97.7%
**3**	71%	88%	99%	100%			92.6%	98.8%
**4**	72%	86%	92%	95%	98%	99%	93.9%	98.5%
**5**	59%	90%	91%	95%	98%		94.9%	98.9%
**6**	100%						91.6%	97.5%
**7**	100%						92.3%	96.0%
**8**	100%						91.1%	96.3%
**9**	100%						91.1%	94.5%

*With lowest residence time where phosphoramidite/starting material distribution ≥ 98%
** Not counting the hydrolysis sideproduct at 13.8 ppm

Bz-dA, ^{1}H NMR (CD_3CN)

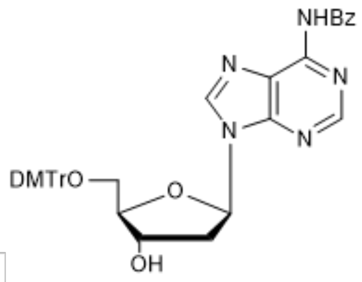

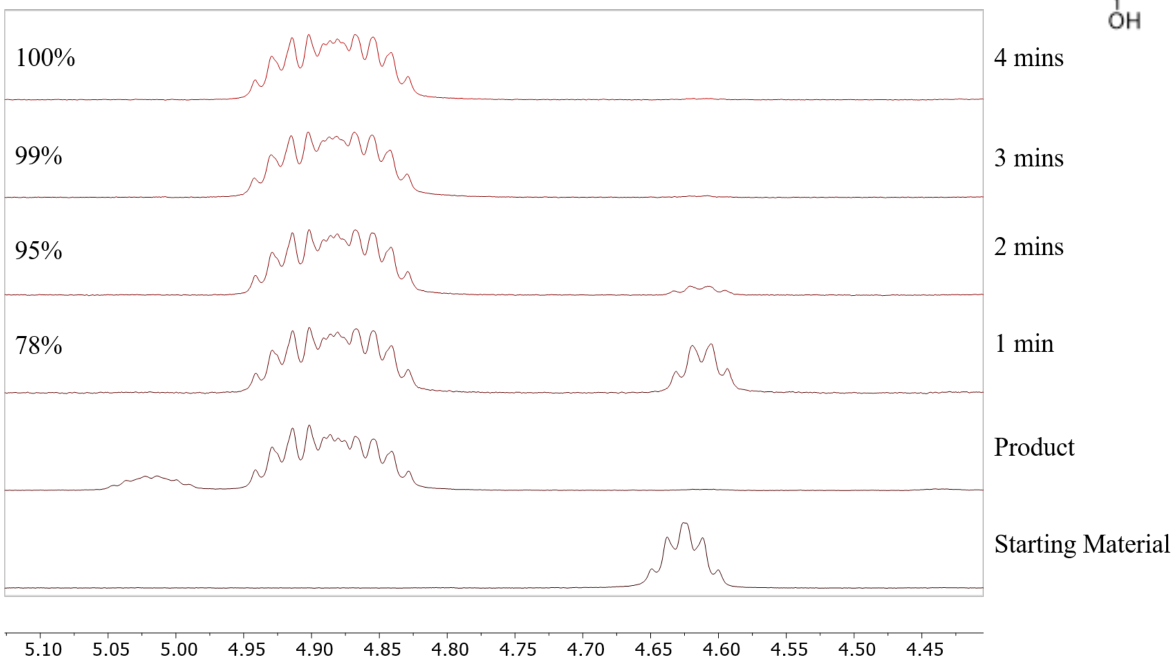

dT, ^{1}H NMR (CD_3CN)

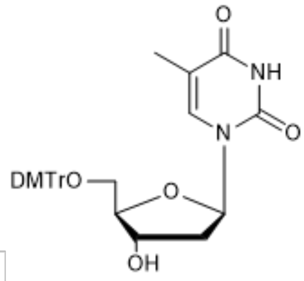

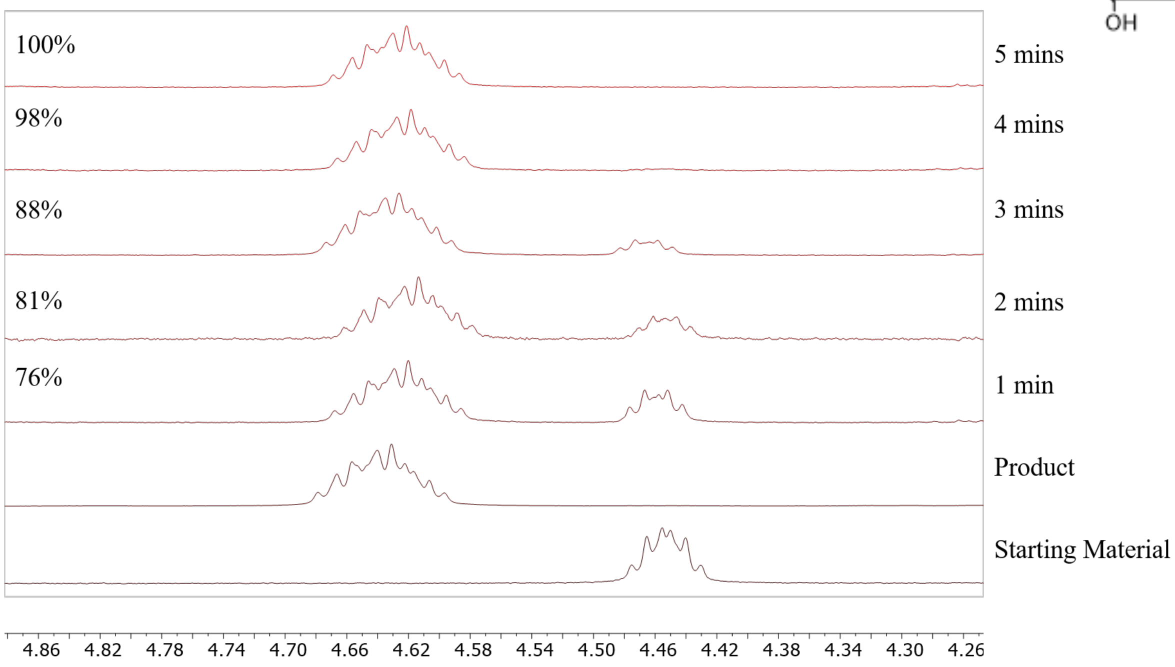

iBu-dG, ^{1}H NMR (CD_3CN)

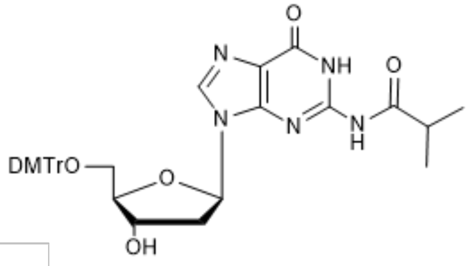

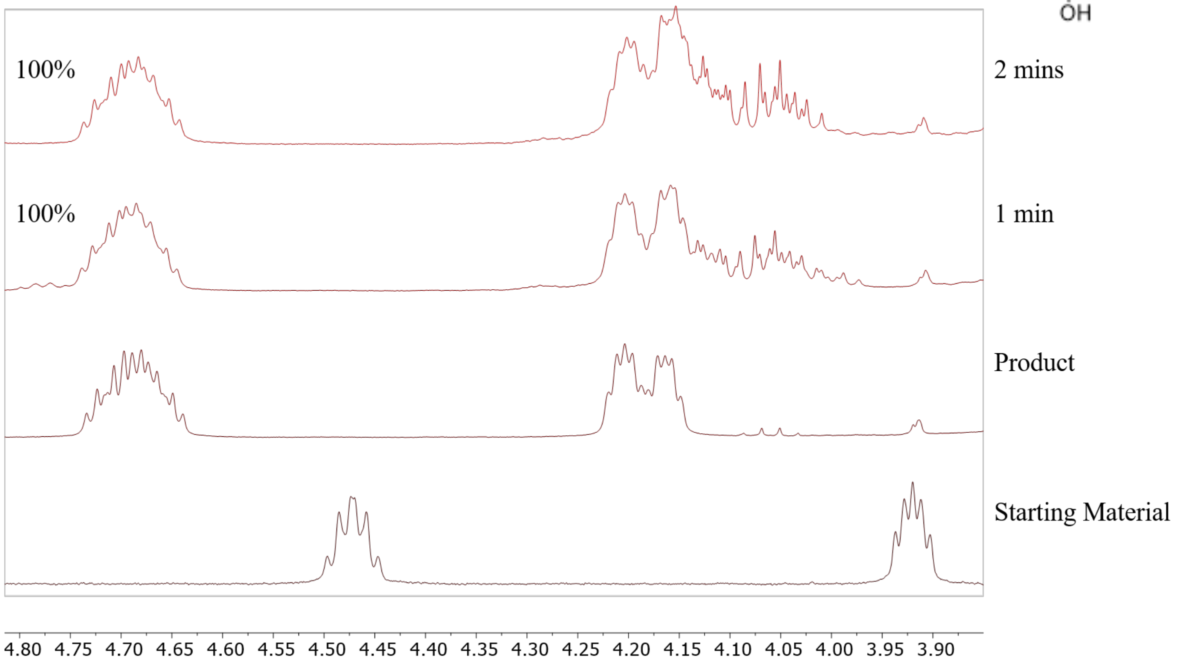

Bz-dC, ^{1}H NMR (CD_3CN)

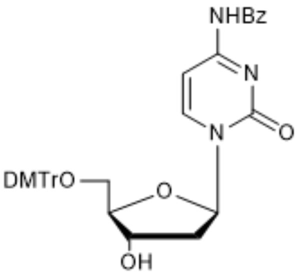

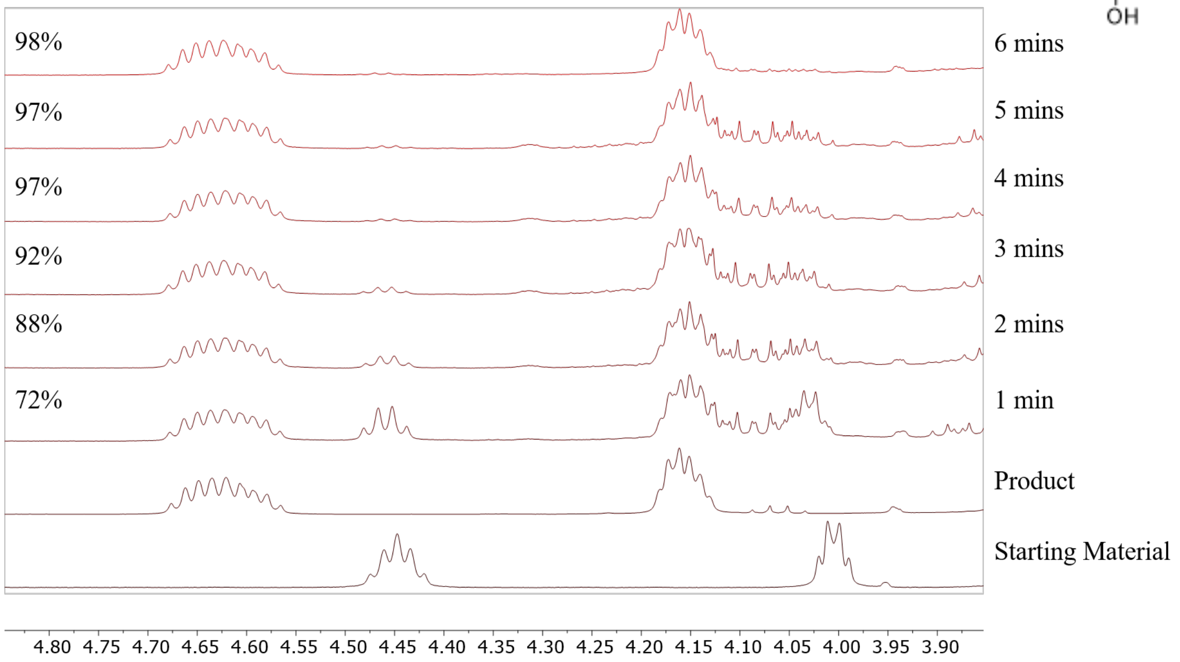

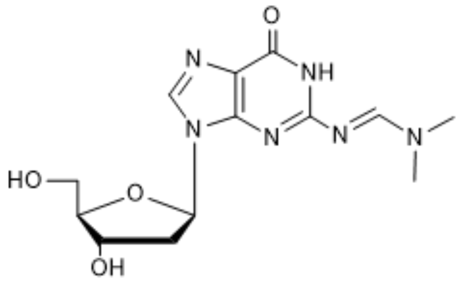

dmf-dG, ^1H NMR (CD_3CN)

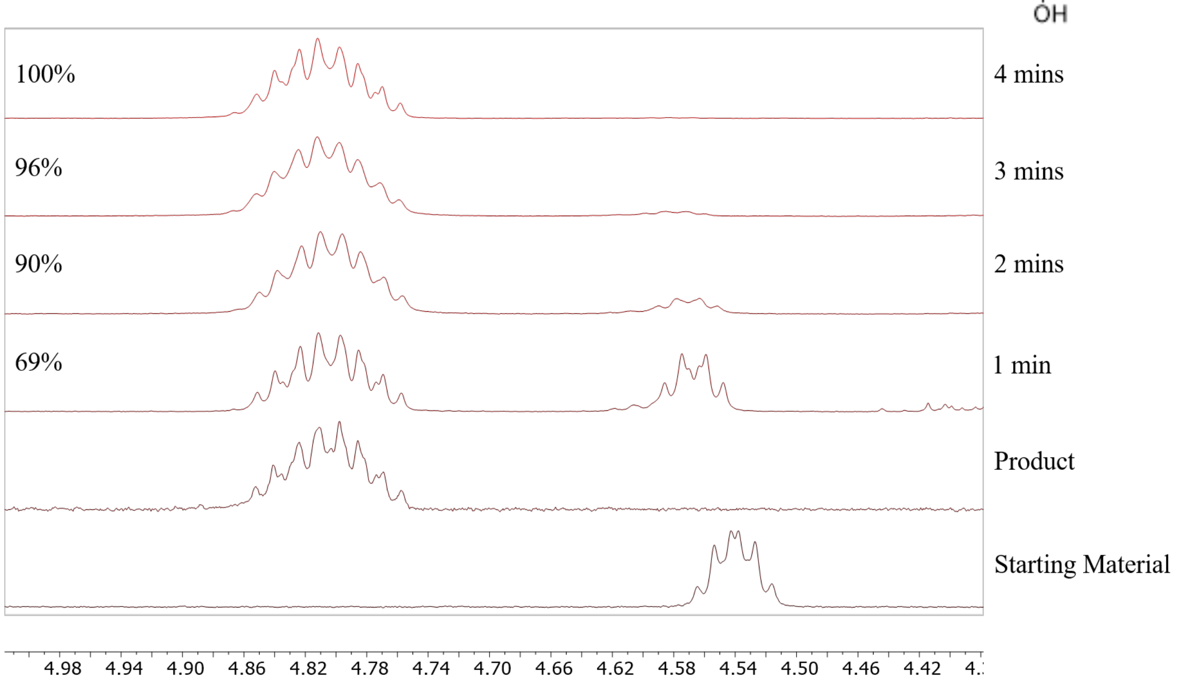

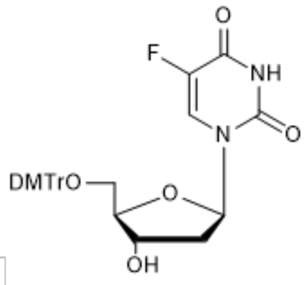

Compound **1**, ^{1}H NMR (CD_3CN)

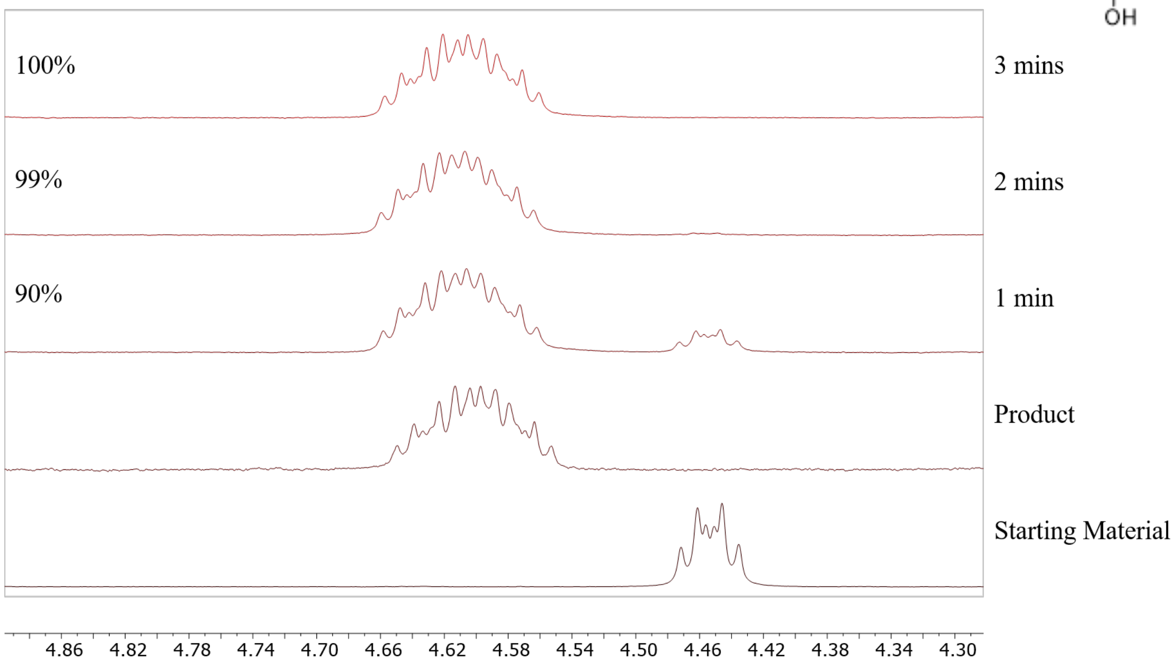

Compound **1**, ^{19}F NMR (CD_3CN)

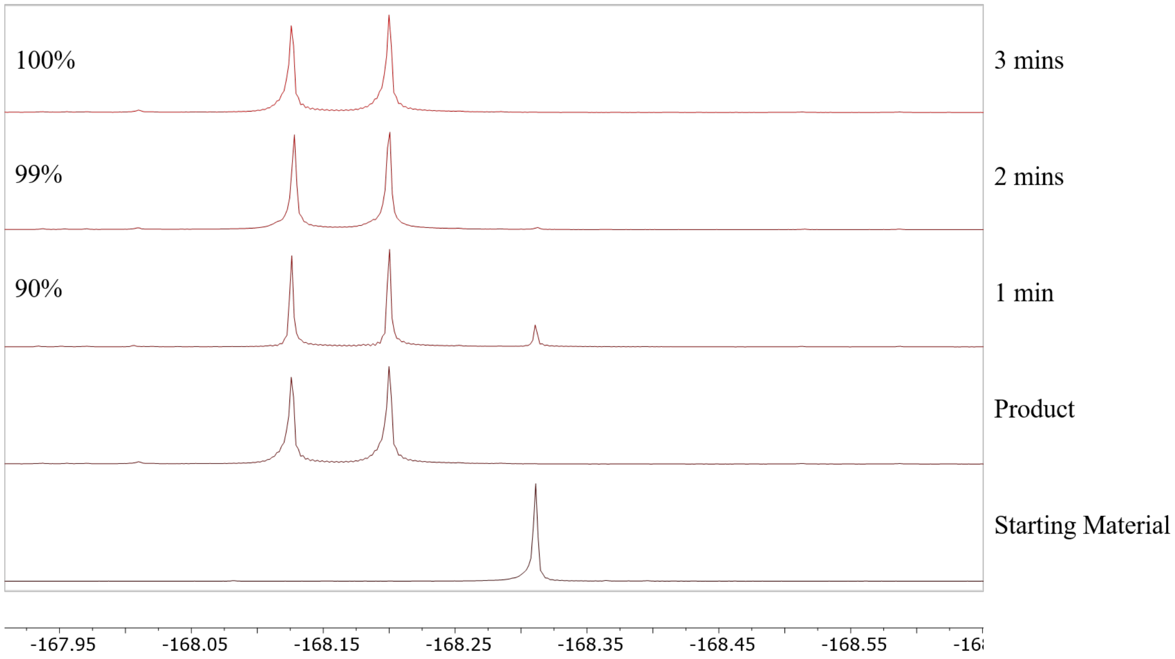

Compound **2**, ^{1}H NMR (CD_3CN)

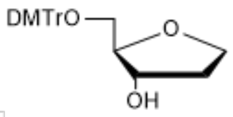

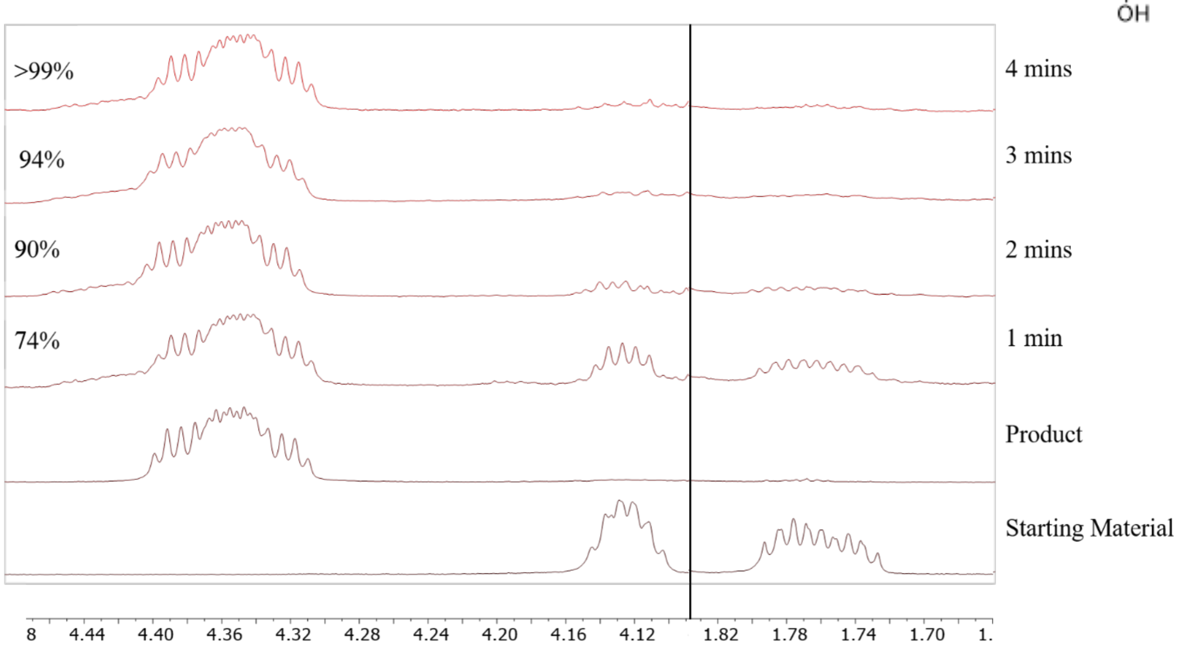

Compound **3**, ^{1}H NMR (CD_3CN)

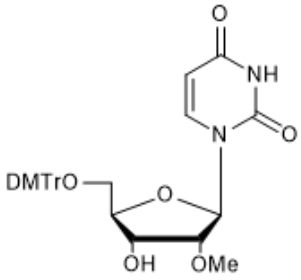

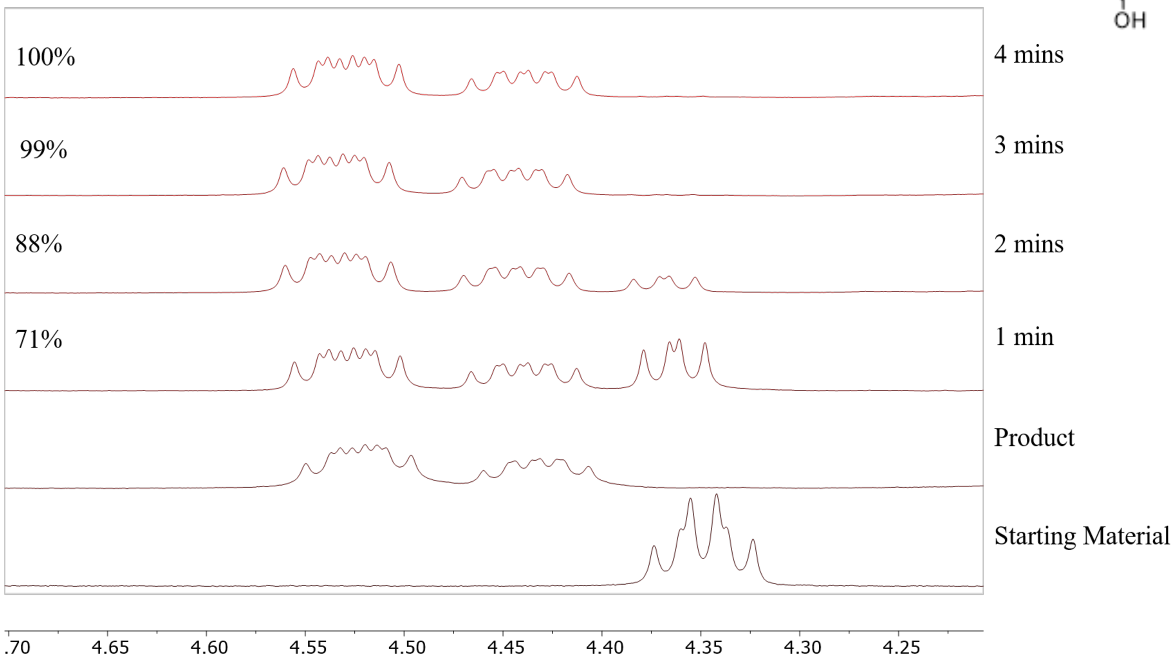

Compound **4**, ^{1}H NMR (CD_3CN)

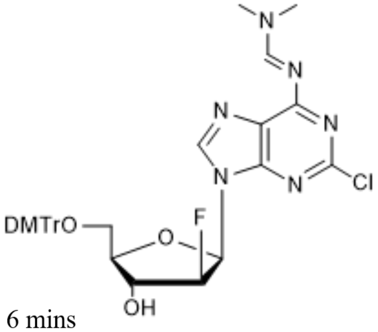

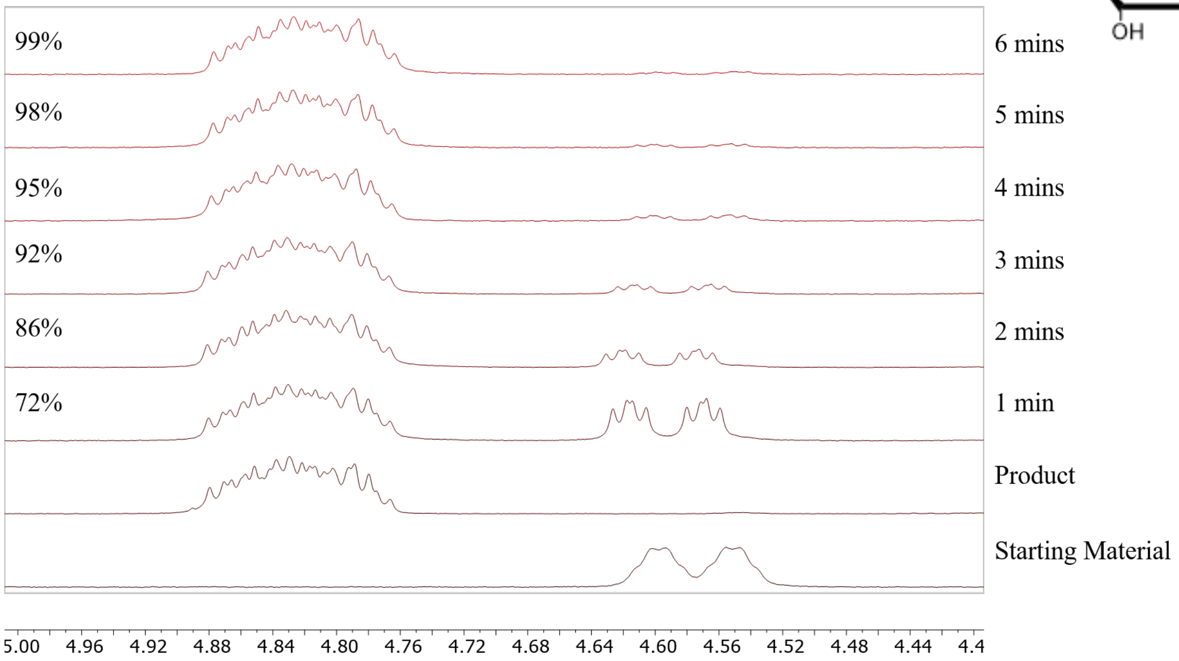

Compound **4**, ^{19}F NMR (CD_3CN)

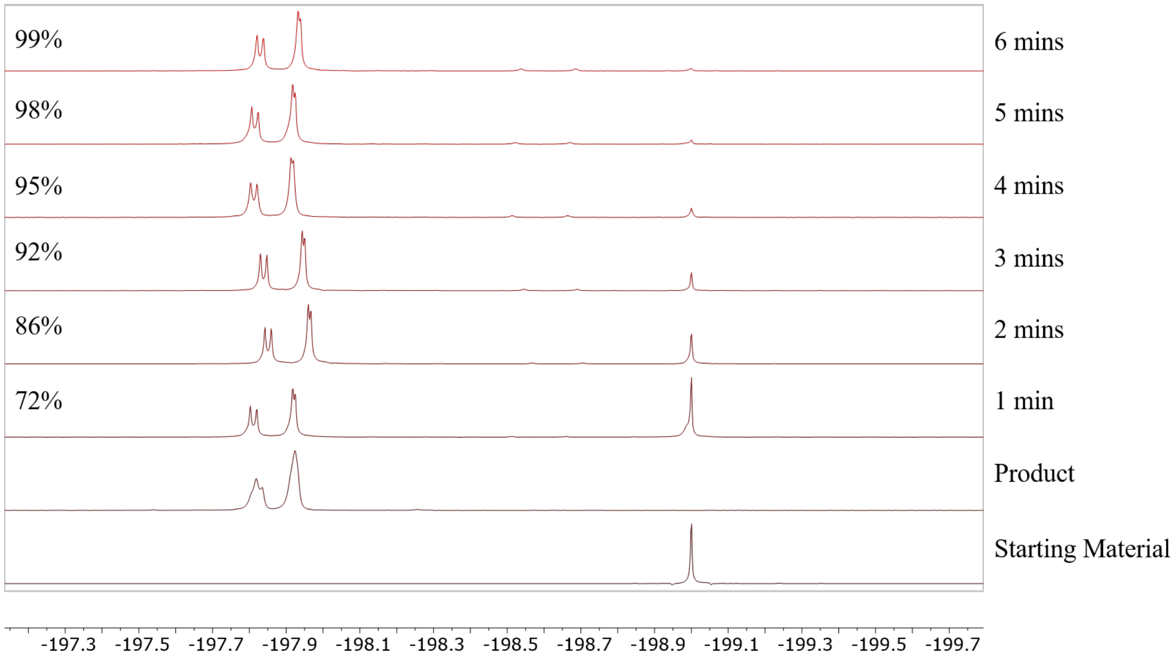

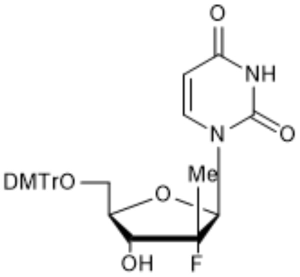

Compound **5**, ^{1}H NMR (CD_3CN)

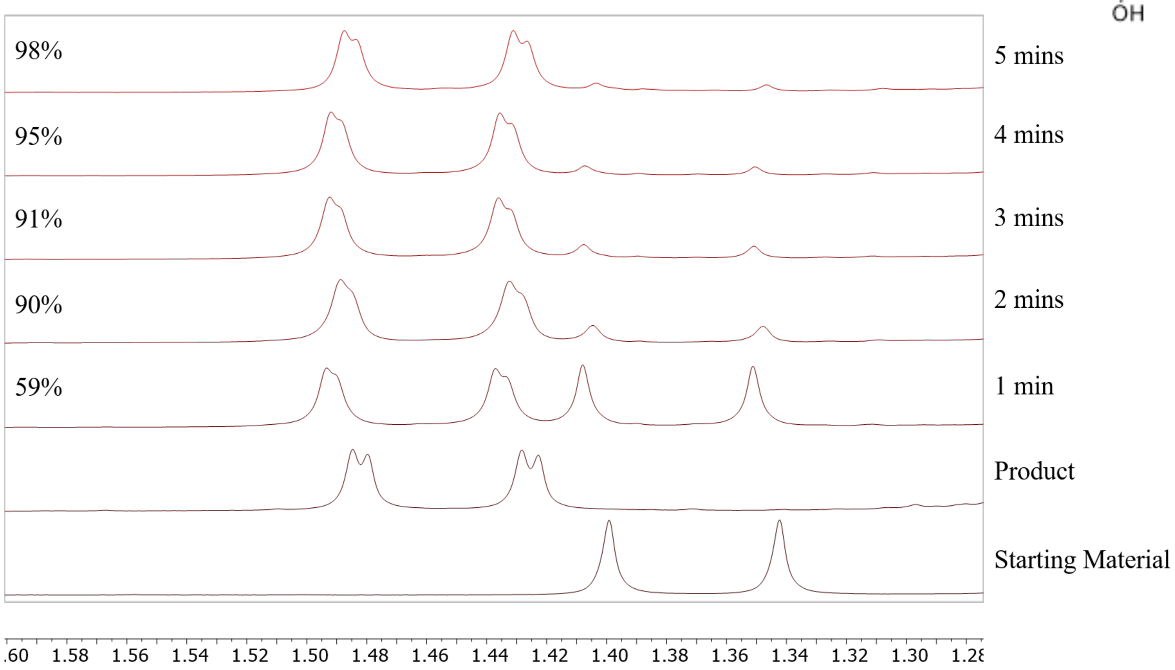

Compound **5**, ^{19}F NMR (CD_3CN)

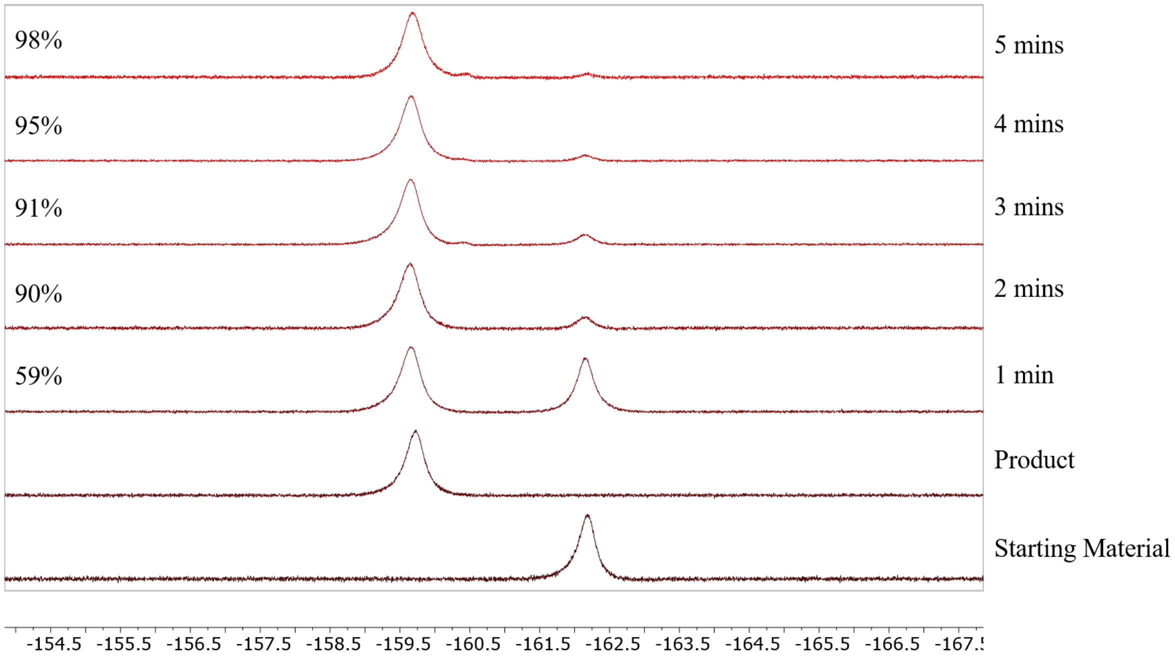

Compound **6**, ^{1}H NMR (CD_3CN)

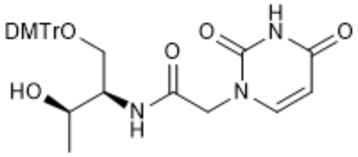

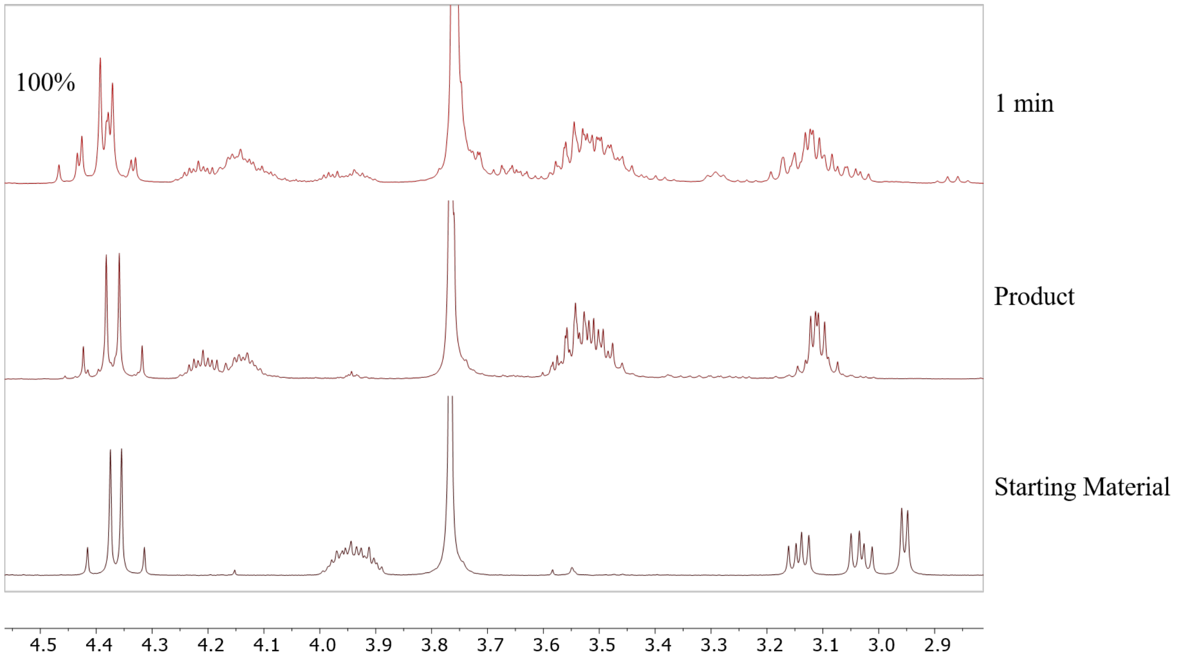

Compound **7**, ^{1}H NMR (CD_3CN)

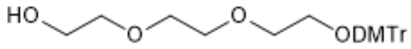

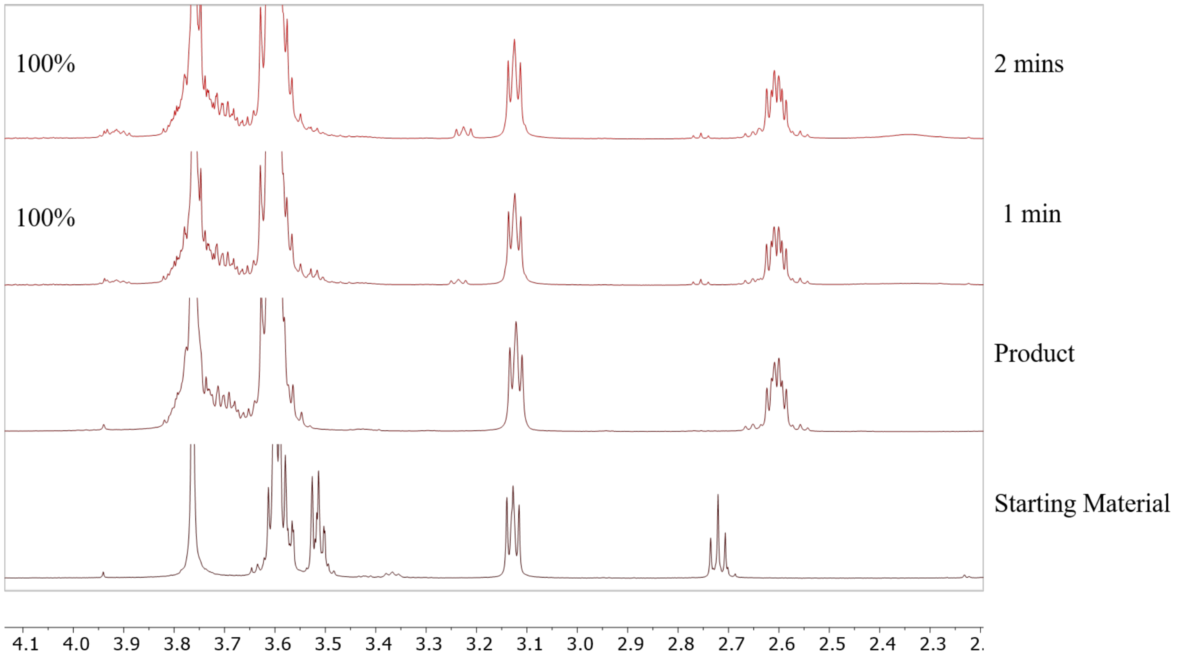

Compound **8**, ^{1}H NMR (CD_3CN)

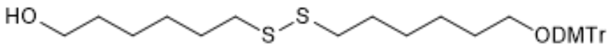

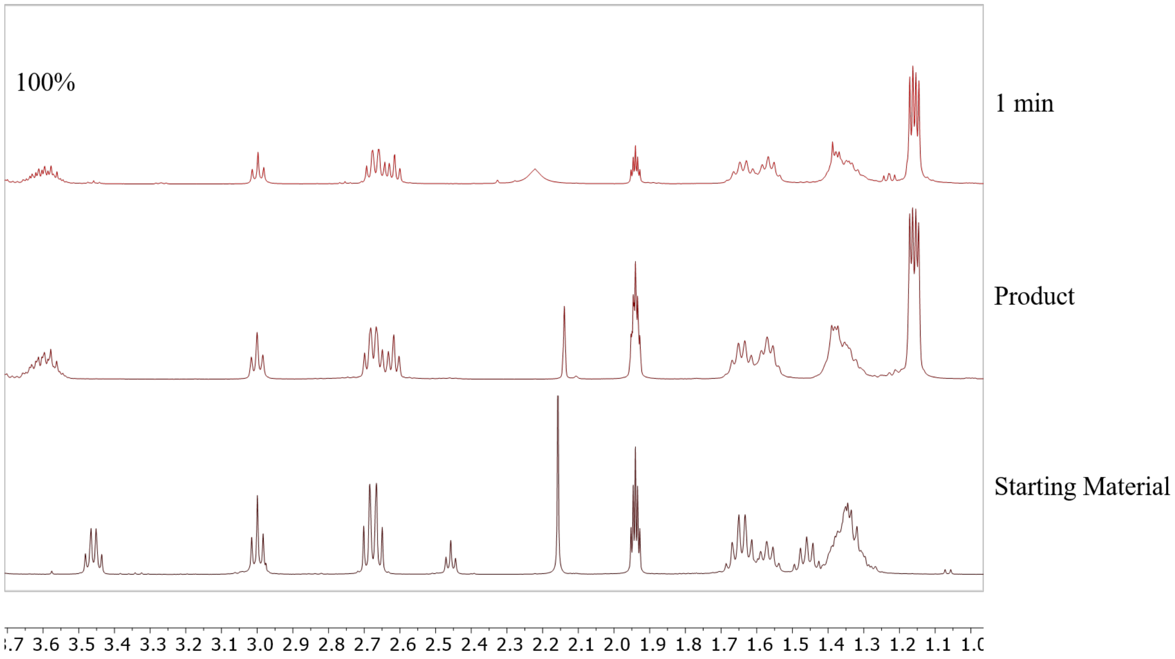

Compound **9**, ^{1}H NMR (CD_3CN)

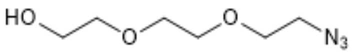

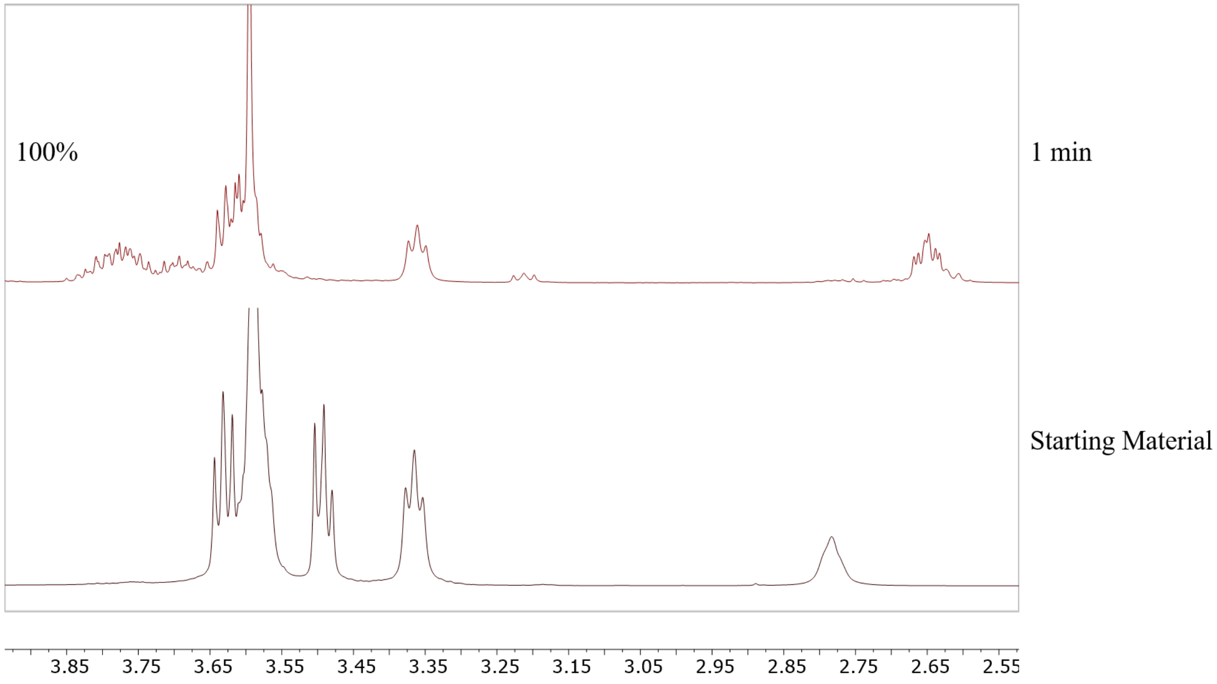

Stability of Phosphoramidite 9-P in Solution

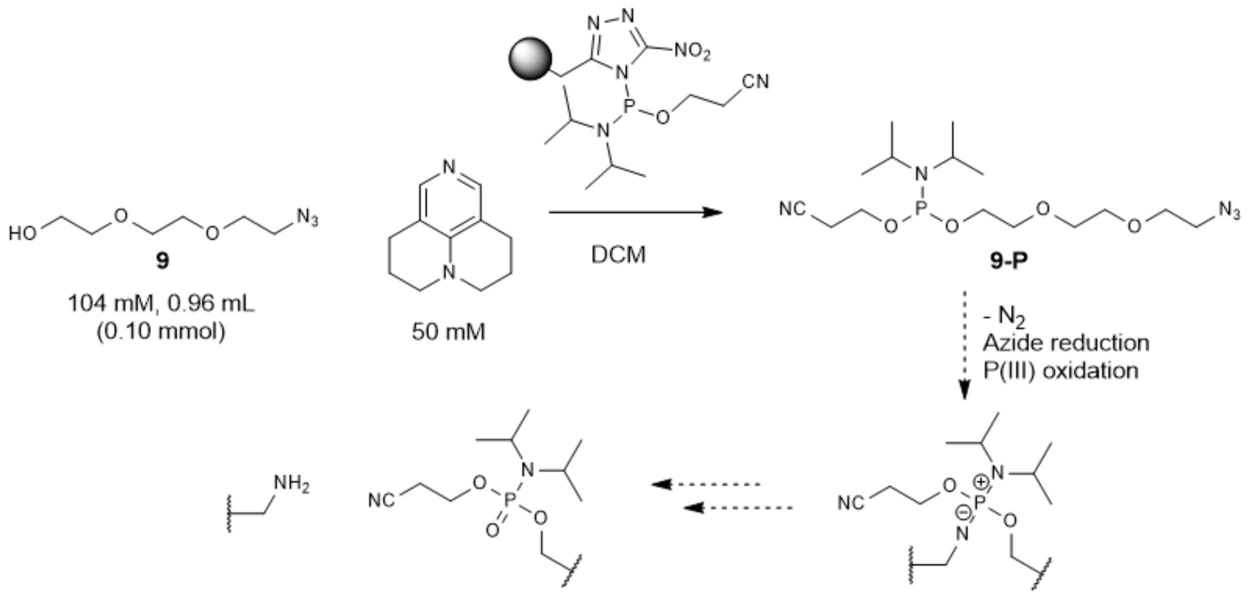

Azide linker **9** (0.1 mmol) with 50 mM 9AJ in CH_2Cl_2 were eluted through the P(III)-loaded flow system and collected in a flask. The collected eluate was concentrated under reduced pressure and the residue redissolved in CD_3CN (1.0 mL) to achieve a final concentration of 0.1 M of **9-P**. The stability of **9-P** was monitored by ^1H NMR and ^{31}P NMR over time. The amount of remaining **9-P** was determined through the ratio of the ^{31}P NMR signal at 147.9 ppm against the total amount of ^{31}P NMR signals related to the redox process and not counting the integral of the hydrolysis signal at 13.8 ppm which remained constant throughout this study.

New ^{31}P NMR signals appearing during storage: 139.9, 32.3, 31.8, 30.4, 17.4, 15.9, 15.8, 15.7, 15.7, 8.6, 5.7, 1.2.

Stacked ^{31}P NMR spectra (CD_3CN)

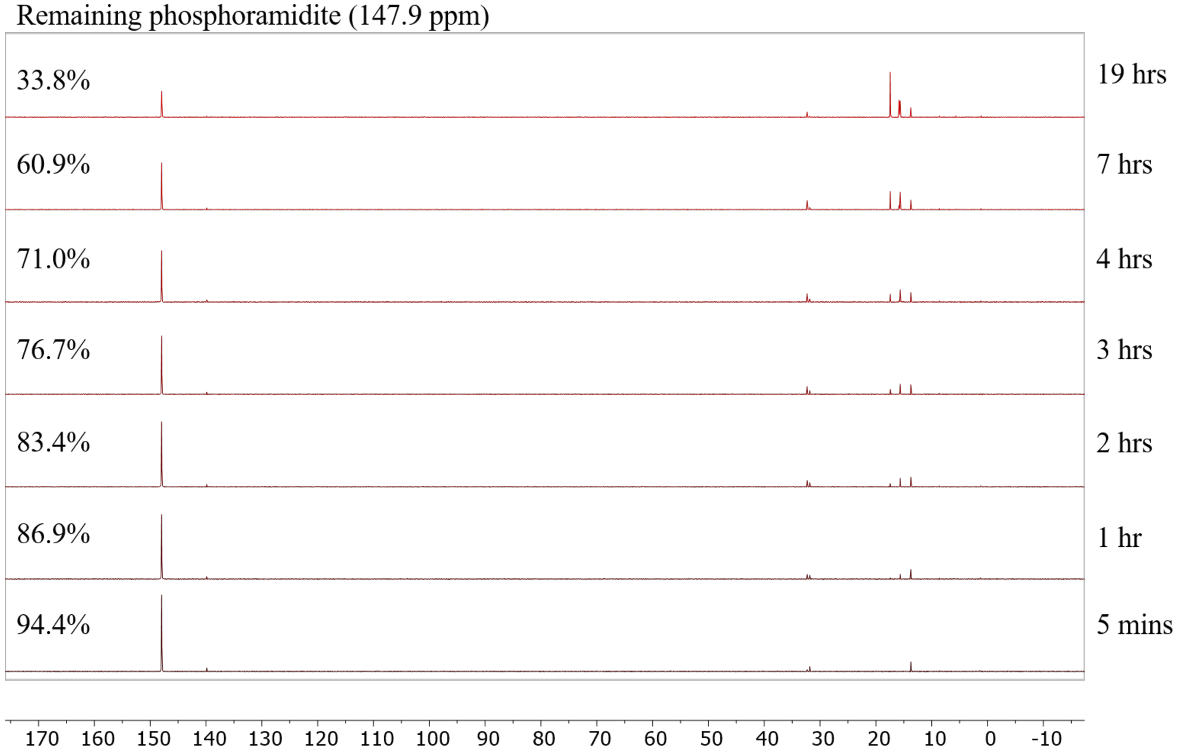

Stacked ^{1}H NMR spectra (CD_3CN)

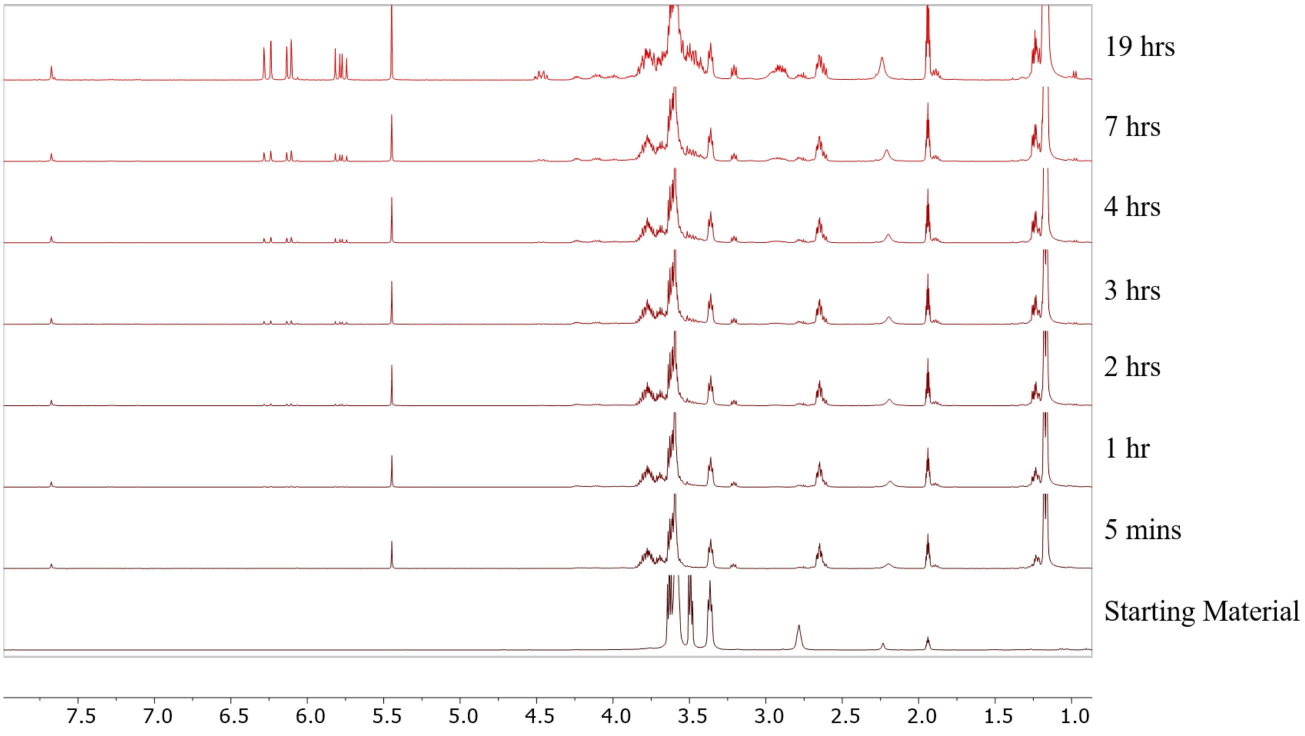

Synthesis and Characterization of Reference Oligonucleotides

Reference oligonucleotides (T_7, T_{15}, T_7AT_7, T_7CT_7, T_7GT_7, 51-mer) were synthesised using standard oligonucleotide synthesis conditions (Table S7). Yields were determined by measuring UV-analysis of collected fractions.

Table S7. Conditions for standard oligonucleotide synthesis.

Reaction step	Reagents	Volume	Coupling time
Wash	MeCN	150 µL	
Deblock	3% TCA in CH_2Cl_2 (w/v)	150 µL	60 s
Coupling	0.1 M phosphoramidite, 0.50 M ETT (1/1, v/v)	140 µL	2 x 60 s
Oxidation	0.02 M I_2, THF/pyridine/H_2O (7/2/1, v/v)	150 µL	60 s
Capping	THF/Ac_2O/NMI (18/1/1, v/v)	150 µL	60 s

HPLC purification and analysis was performed using the following methods:

Method 1:
Solvent A: 0.1 M Triethylammonium acetate, pH = 7
Solvent B: MeCN
Gradient: 5% to 20% B over 15 mins, 20% to 70% B 15-20 mins

Method 2:
Solvent A: 0.1 M Triethylammonium acetate, pH = 7
Solvent B: MeCN
Gradient: 5% to 15% B over 15 mins, 20% to 70% B 15-20 mins

HPLC Method	1
Sequence	5’ TTT TTT T 3’
Retention time	10.1 mins
Calculated mass	2067.4
Found mass (LCMS)	2066.7

HPLC chromatogram (260 nm) of crude 5’ TTT TTT T3’ after cleavage and concentration.

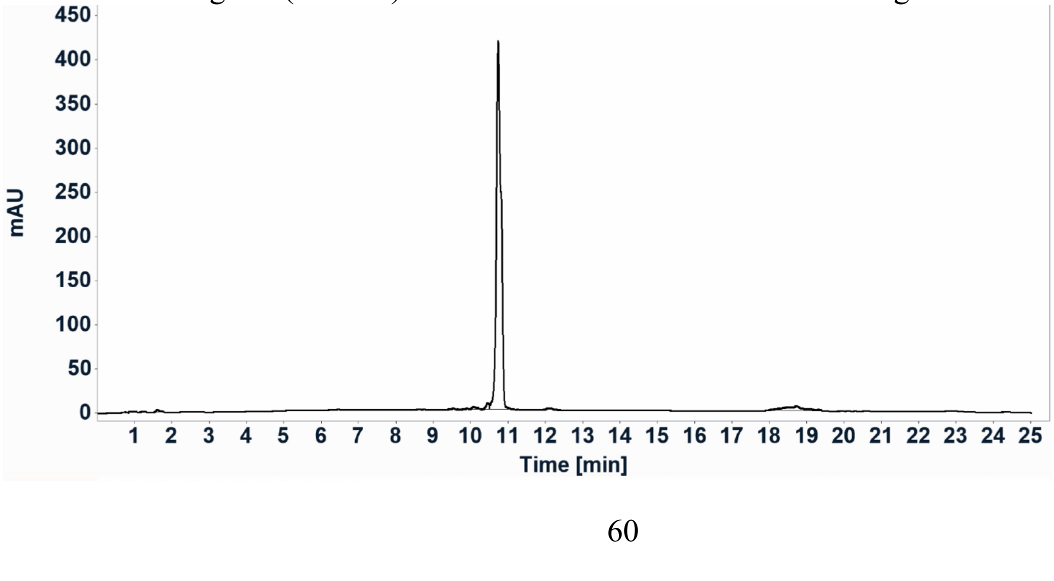

HPLC Method	1
Sequence	5’ TTT TTT TTT TTT TTT 3’
Retention time	12.2 mins
Calculated mass	4501.0
Found mass (LCMS)	4501.5

HPLC chromatogram (260 nm) of crude 5’ TTT TTT TTT TTT TTT 3’ after cleavage and concentration.

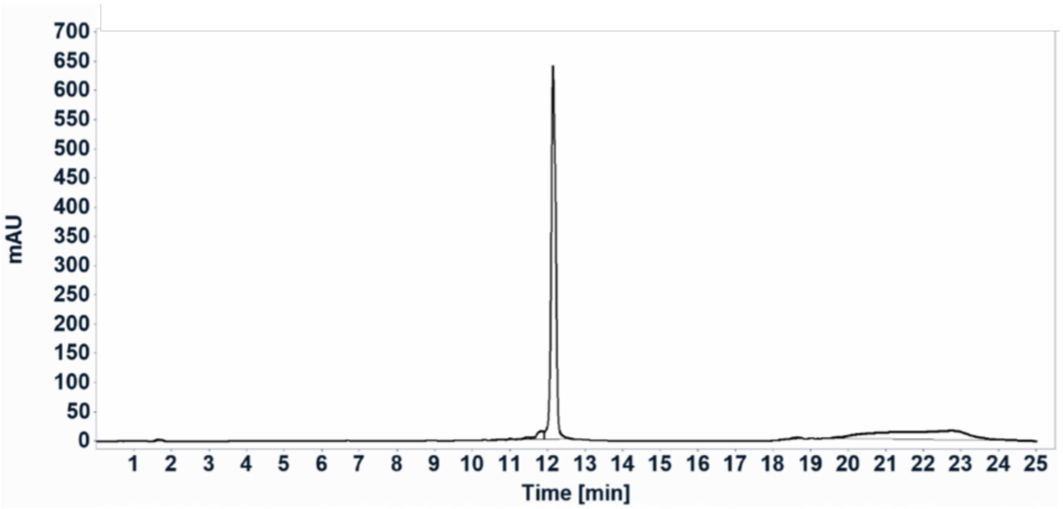

HPLC Method	1
Sequence	5’ TTT TTT TAT TTT TTT 3’
Retention time	12.1 mins
Calculated mass	4510.0
Found mass (LCMS)	4510.5

HPLC chromatogram (260 nm) of crude 5’ TTT TTT TAT TTT TTT 3’ after cleavage and concentration.

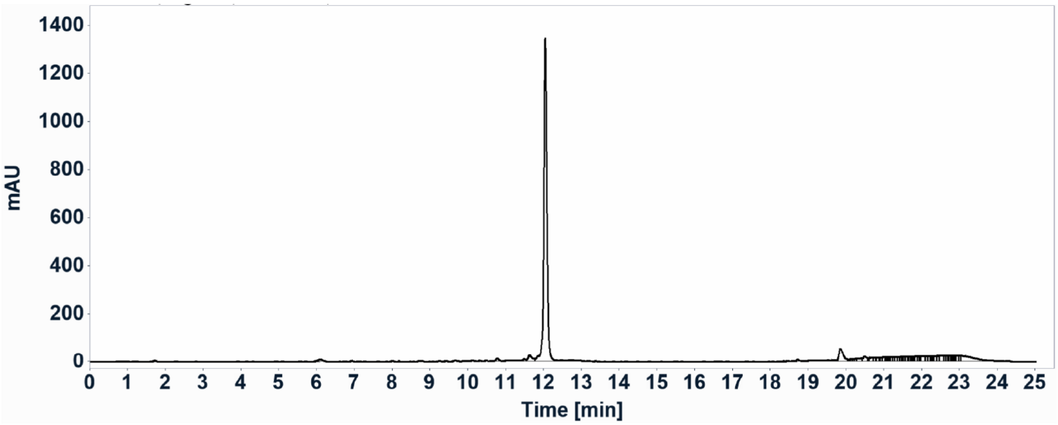

HPLC Method	1
Sequence	5’ TTT TTT TCT TTT TTT 3’
Retention time	12.0 mins
Calculated mass	4485.9
Found mass (LCMS)	4486.5

HPLC chromatogram (260 nm) of crude 5’ TTT TTT TCT TTT TTT 3’ after cleavage and concentration.

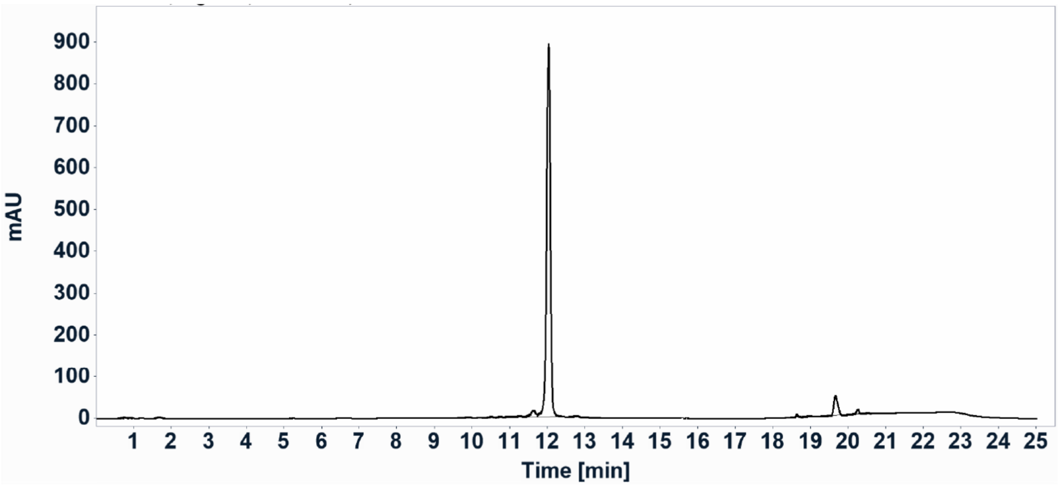

HPLC Method	1
Sequence	5’ TTT TTT TGT TTT TTT 3’
Retention time	11.9 mins
Calculated mass	4526.0
Found mass (LCMS)	4526.7

HPLC chromatogram (260 nm) of crude 5’ TTT TTT TGT TTT TTT 3’ after cleavage and concentration.

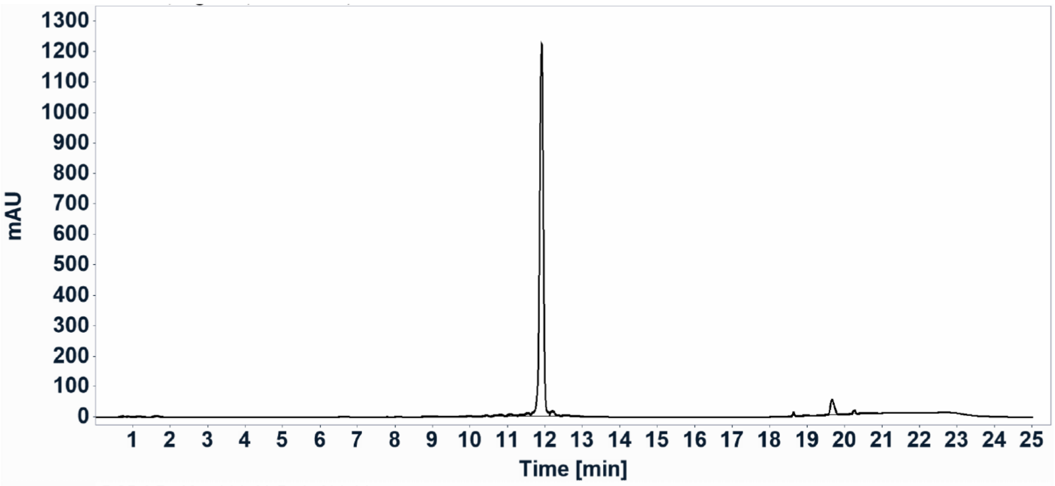

HPLC Method	2
Sequence	5'CCG CTT TCT AGT TCG TCC TCC ATA ATT AAT TTC CTA GAG TCC TAC GTG CTC 3'
Retention time	15.9 mins
Calculated mass	15467.9
Found mass (LCMS)	15467.6
Coupling time	2 x 60 s
Yield	41.8%
Average Cycle Yield	98.3%

HPLC chromatogram (260 nm) of 51-mer after cleavage and concentration.

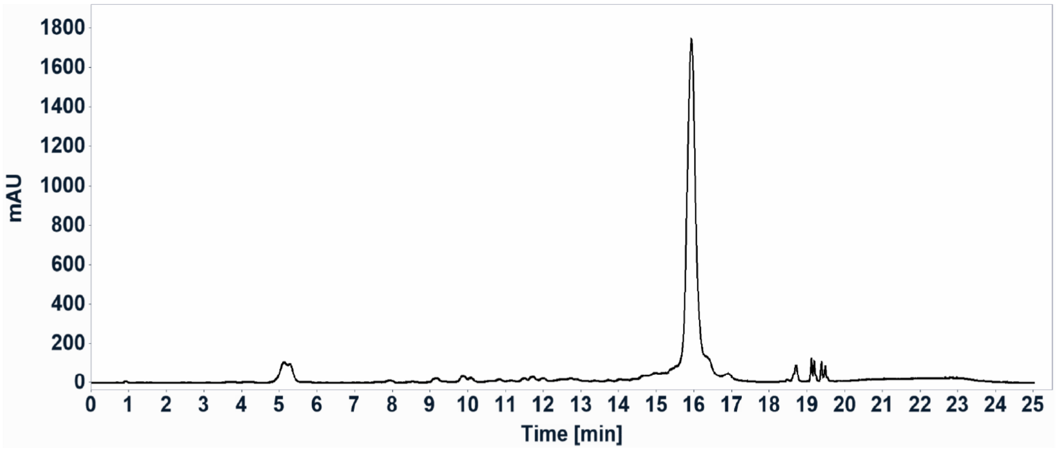

Single Coupling Reactions

The synthetic cycle described in Table S5 with optimal residence time for the given alcohols according to Table S6 was used for the synthesis of the phosphoramidites. The eluate was collected for 6 void volumes and concentrated under reduced pressure. The residue was redissolved in MeCN (1.0 mL) to reach a final concentration of 0.1 M. Standard coupling conditions (Table S7) were used for phosphoramidites synthesised by conventional methods.

Common Sequence (T_7**B**T_7): 5' TTT TTT T**B**T TTT TTT 3'

For compound **8-P** and **9-P**, oligonucleotide synthesis was stopped after coupling with **8-P** or **9-P** and oxidation. The coupling yield was based on integration ratio of absorbance at 260 nm between the truncated oligonucleotide (T_7): 5' TTT TTT T 3' and the desired full-length oligonucleotide (T_7XT_7) on HPLC chromatogram by the formula:

$$Yield = \frac{A_{T_7XT_7}}{A_{Total}} = \frac{A_{T_7XT_7}}{A_{T_7XT_7} + \frac{\varepsilon(T_7XT_7)_{260}}{\varepsilon(T_7)_{260}} \cdot A_{T_7}}$$

Where ε is the molar extinction coefficient for the given oligonucleotide. ε was calculated using http://www.molbiotools.com/dnacalculator.html.

For the nucleotides **1**, **3**, **5** and **6**, thymidine was used in the calculation of ε and for **4**, adenosine was used instead. For the calculation of oligonucleotides containing **2** and **7**, ε was calculated as 2 times ε_{T_7}.

The oligonucleotides were cleaved directly from the resin by treatment with AMA for 30 mins at 65 °C if G was present in the sequence and otherwise cleaved by treatment with concentrated aqueous NH_3 for 30 mins at 50 °C. The supernatant was concentrated under reduced pressure and the residue subjected to HPLC purification.

Table S8. Summary of results from single coupling study

Entry	Coupling time	Coupling yield
T	2 x 60 s	99.7%
Bz-dA	2 x 60 s	99.3%
Bz-dC	2 x 60 s	99.8%
iBu-dG	2 x 60 s	99.3%
**1**	2 x 60 s	99.7%
**2**	2 x 60 s	98.3%
**3**	2 x 60 s	99.2%
**3**	2 x 240 s	99.7%
**4**	2 x 60 s	94.8%
**4**	2 x 240 s	99.6%
**5**	2 x 60 s	70.9%
**5**	2 x 240s	98.1%
**6**	2 x 60 s	99.2%
**7**	2 x 60 s	98.0%
**8**	2 x 60 s	99.0%
**9**	2 x 60 s	99.4%

HPLC Method	1
Sequence	5’ TTT TTT T**T**T TTT TTT 3’
Retention time	12.0 mins
Calculated mass	4501.0
Found mass (LCMS)	4501.4
$\varepsilon(T_7)_{260}$	57300
$\varepsilon(T_7XT_7)_{260}$	122100
Coupling time	2 x 60 s
$A(T_7)$	20
$A(T_7XT_7)$	13360
Coupling Yield	99.7%

HPLC chromatogram (260 nm) of crude 5’ TTT TTT T**T**T TTT TTT 3’ after cleavage and concentration.

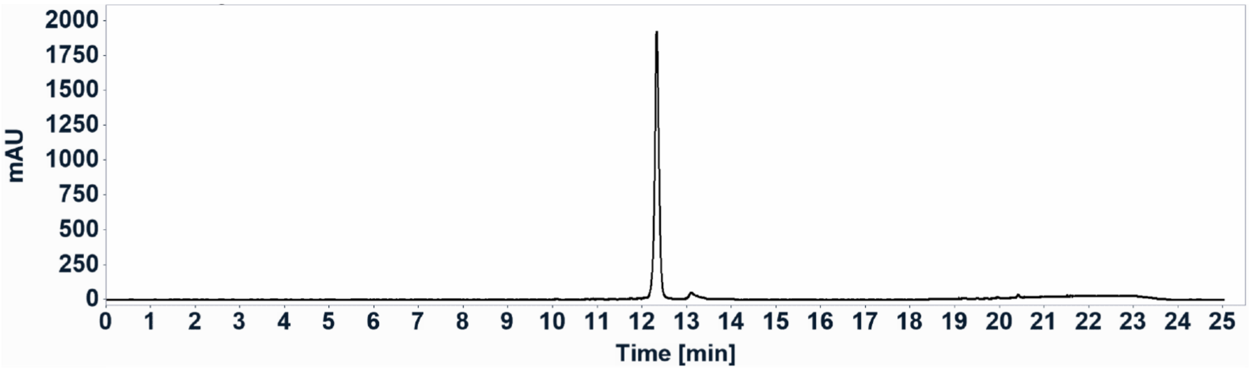

HPLC Method	1
Sequence	5’ TTT TTT T**A**T TTT TTT 3’
Retention time	12.2 mins
Calculated mass	4510.0
Found mass (LCMS)	4510.3
$\varepsilon(T_7)_{260}$	57300
$\varepsilon(T_7XT_7)_{260}$	128000
Coupling time	2 x 60 s
$A(T_7)$	35
$A(T_7XT_7)$	11047
Coupling Yield	99.3%

HPLC chromatogram (260 nm) of crude 5’ TTT TTT T**A**T TTT TTT 3’ after cleavage and concentration.

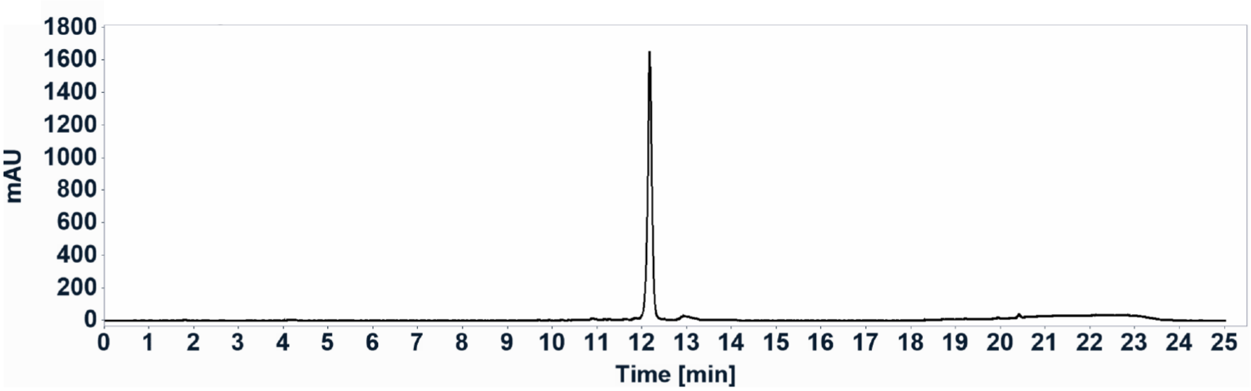

HPLC Method	1
Sequence	5' TTT TTT T**C**T TTT TTT 3'
Retention time	11.9 mins
Calculated mass	4485.9
Found mass (LCMS)	4486.3
$\varepsilon(T_7)_{260}$	57300
$\varepsilon(T_7XT_7)_{260}$	121200
Coupling time	2 x 60 s
$A(T_7)$	10
$A(T_7XT_7)$	11254
Coupling Yield	99.8%

HPLC chromatogram (260 nm) of crude 5' TTT TTT T**C**T TTT TTT 3' after cleavage and concentration.

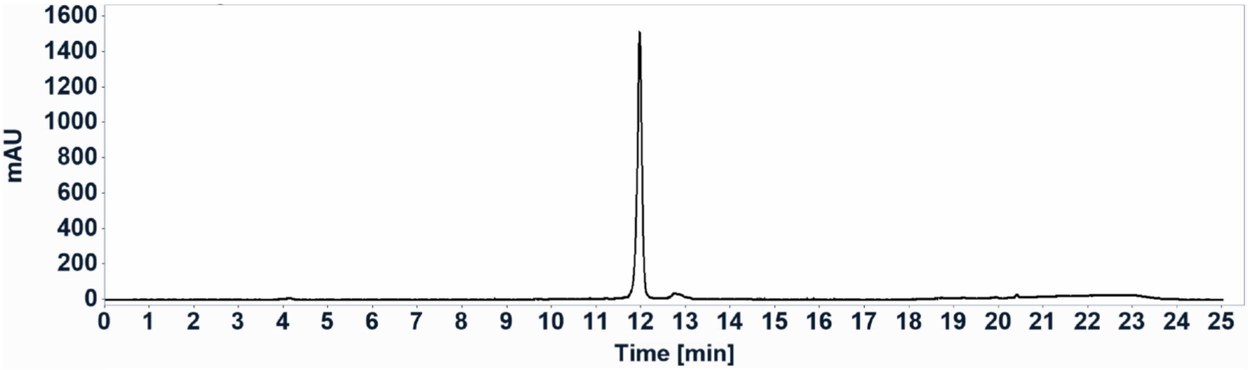

HPLC Method	1
Sequence	5' TTT TTT T**G**T TTT TTT 3'
Retention time	12.0 mins
Calculated mass	4526.0
Found mass (LCMS)	4526.4
$\varepsilon(T_7)_{260}$	57300
$\varepsilon(T_7XT_7)_{260}$	124700
Coupling time	2 x 60 s
$A(T_7)$	37
$A(T_7XT_7)$	11535
Coupling Yield	99.3%

HPLC chromatogram (260 nm) of crude 5' TTT TTT T**G**T TTT TTT 3' after cleavage and concentration.

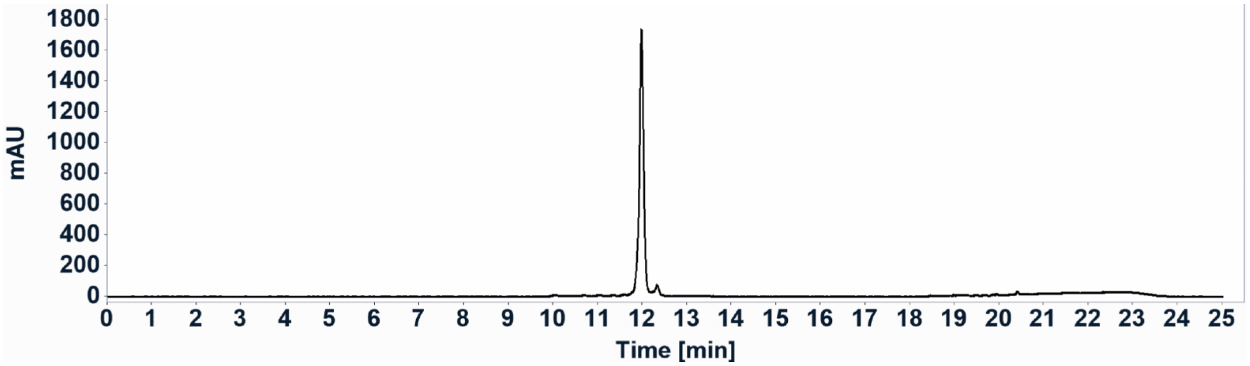

HPLC Method	1
Sequence	5' TTT TTT T**1**T TTT TTT 3'
Retention time	12.2 mins
Calculated mass	4504.9
Found mass (LCMS)	4504.1
$\varepsilon(T_7)_{260}$	57300
$\varepsilon(T_7XT_7)_{260}$	122100
Coupling time	2 x 60 s
$A(T_7)$	20
$A(T_7XT_7)$	13951
Coupling Yield	99.7%

HPLC chromatogram (260 nm) of crude 5' TTT TTT T**1**T TTT TTT 3' after cleavage and concentration.

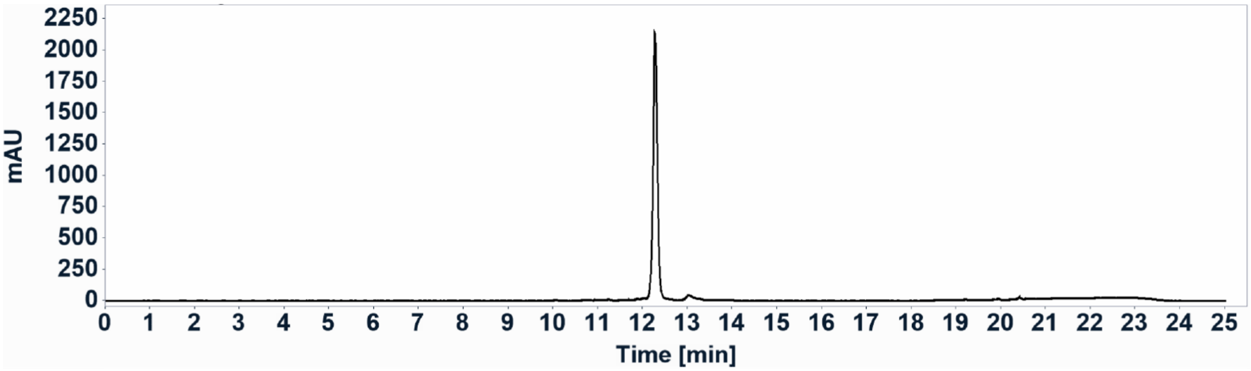

HPLC Method	1
Sequence	5’ TTT TTT T**2**T TTT TTT 3’
Retention time	12.1 mins
Calculated mass	4376.9
Found mass (LCMS)	4376.4
$\varepsilon(T_7)_{260}$	57300
$\varepsilon(T_7XT_7)_{260}$	114600
Coupling time	2 x 60 s
$A(T_7)$	75
$A(T_7XT_7)$	9389
Coupling Yield	98.4%

HPLC chromatogram (260 nm) of crude 5’ TTT TTT T**2**T TTT TTT 3’ after cleavage and concentration.

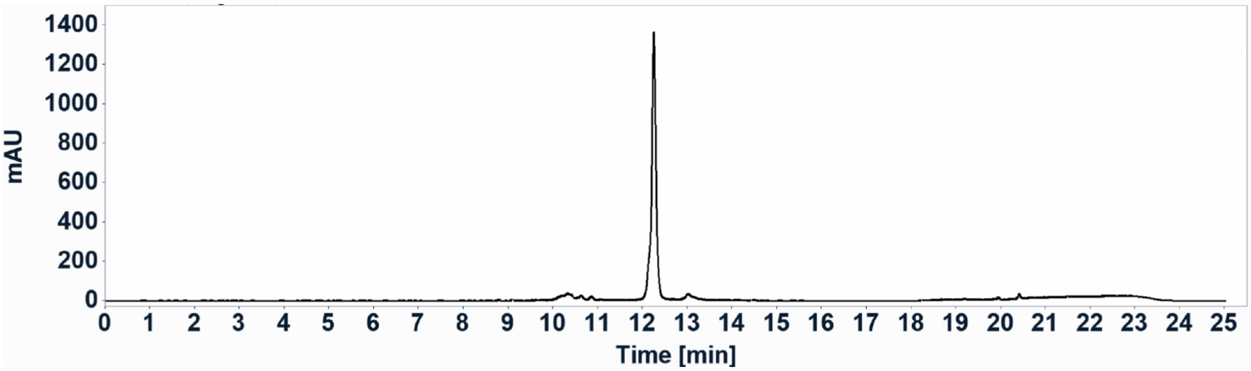

HPLC Method	1	
Sequence	5' TTT TTT T**3**T TTT TTT 3'	
Retention time	12.5 mins	
Calculated mass	4517.0	
Found mass (LCMS)	4516.4	
$\varepsilon(T_7)_{260}$	57300	
$\varepsilon(T_7XT_7)_{260}$	122100	
Coupling time	2 x 60 s	2 x 240 s
$A(T_7)$	52	15
$A(T_7XT_7)$	13888	12376
Coupling Yield	99.2%	99.7%

HPLC chromatograms (260 nm) of crude 5' TTT TTT T**3**T TTT TTT 3' after cleavage and concentration.

Coupling time = 2 x 60 s

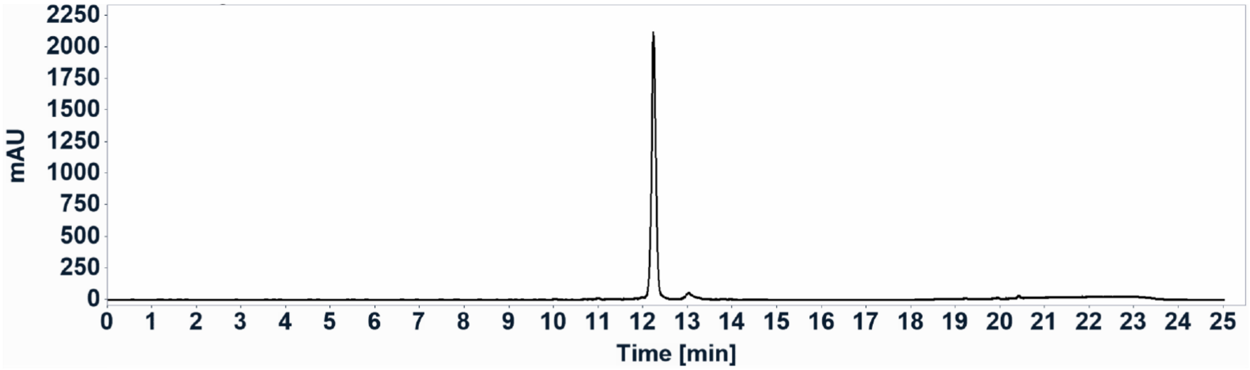

Coupling time = 2 x 240 s

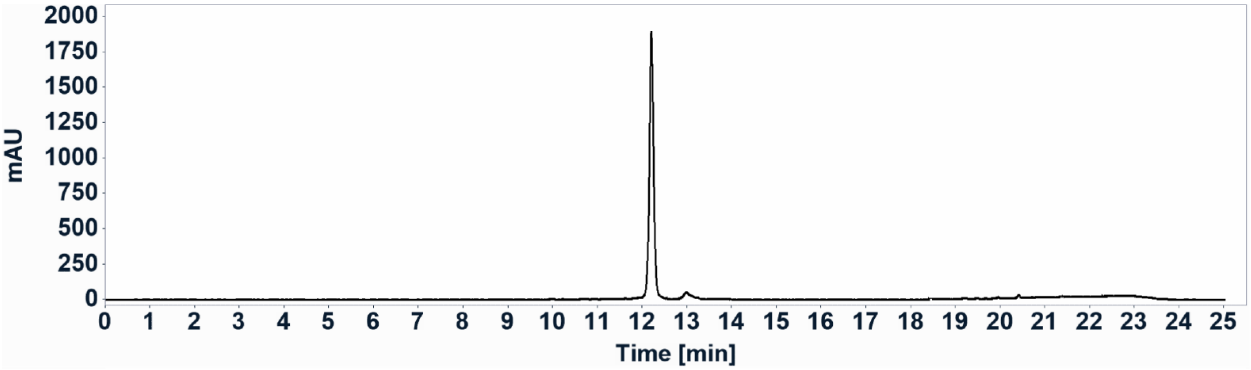

HPLC Method	1	
Sequence	5' TTT TTT T**4**T TTT TTT 3'	
Retention time	12.9 mins	
Calculated mass	4562.4	
Found mass (LCMS)	4561.8	
$\varepsilon(T_7)_{260}$	57300	
$\varepsilon(T_7XT_7)_{260}$	128000	
Coupling time	2 x 60 s	2 x 240 s
$A(T_7)$	267	20
$A(T_7XT_7)$	10799	11421
Coupling Yield	94.8%	99.6%

HPLC chromatograms (260 nm) of crude 5' TTT TTT T**4**T TTT TTT 3' after cleavage and concentration.

Coupling time = 2 x 60 s

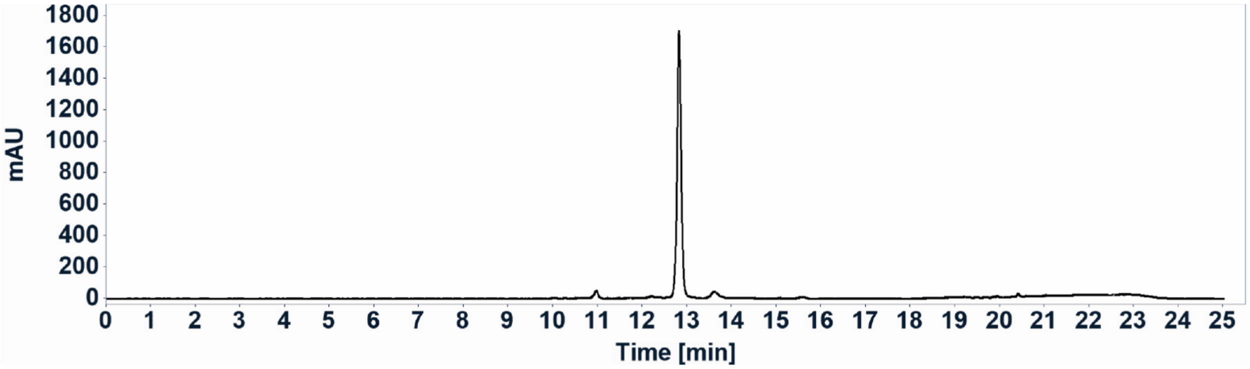

Coupling time = 2 x 240 s

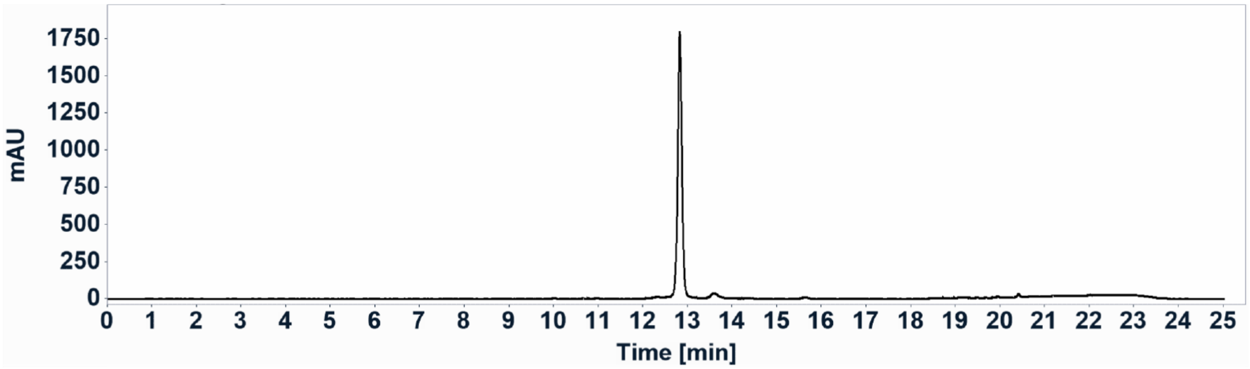

HPLC Method	1	
Sequence	5' TTT TTT T**5**T TTT TTT 3'	
Retention time	12.8 mins	
Calculated mass	4518.9	
Found mass (LCMS)	4518.3	
$\varepsilon(T_7)_{260}$	57300	
$\varepsilon(T_7XT_7)_{260}$	122100	
Coupling time	2 x 60 s	2 x 240 s
$A(T_7)$	1586	86
$A(T_7XT_7)$	8236	9345
Coupling Yield	70.9%	98.1%

HPLC chromatograms (260 nm) of crude 5' TTT TTT T**5**T TTT TTT 3' after cleavage and concentration.

Coupling time = 2 x 60 s

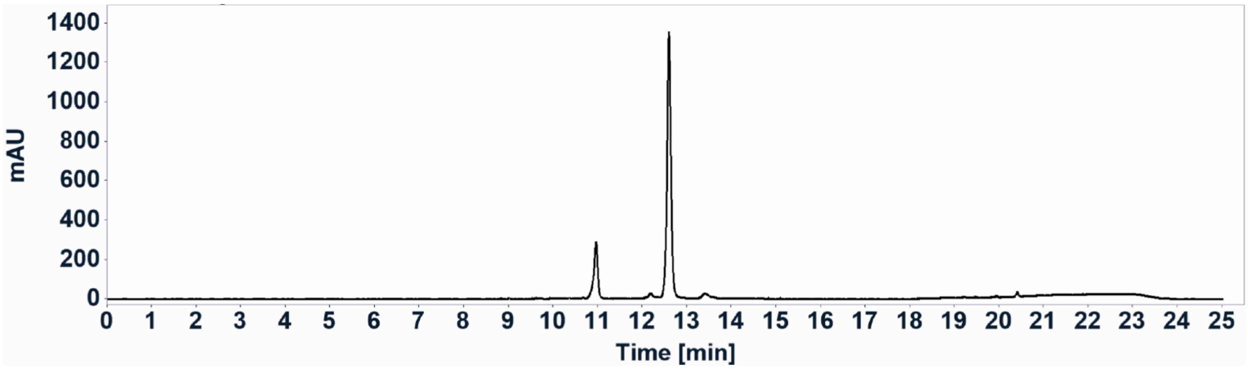

Coupling time = 2 x 240 s

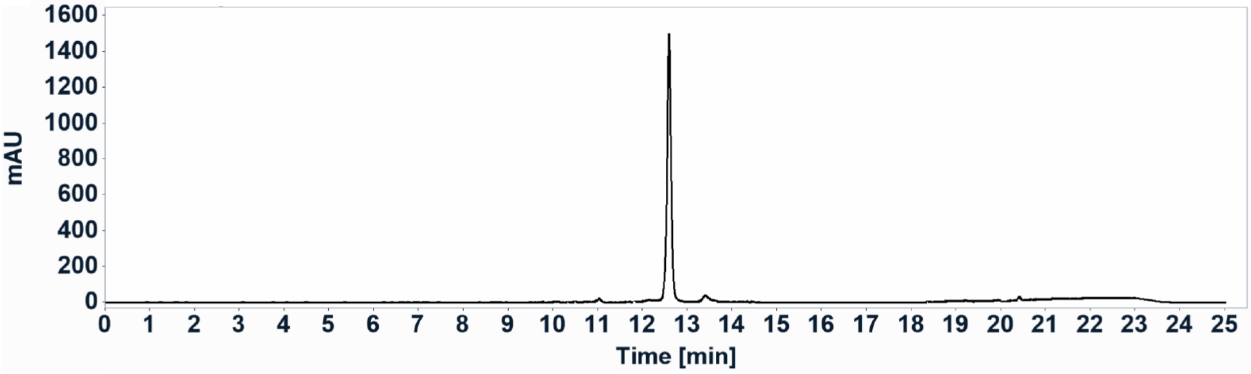

HPLC Method	1
Sequence	5’ TTT TTT T**6**T TTT TTT 3’
Retention time	11.9 mins
Calculated mass	4516.0
Found mass (LCMS)	4515.3
$\varepsilon(T_7)_{260}$	57300
$\varepsilon(T_7XT_7)_{260}$	122100
Coupling time	2 x 60 s
$A(T_7)$	49
$A(T_7XT_7)$	12431
Coupling Yield	99.2%

HPLC chromatogram (260 nm) of crude 5’ TTT TTT T**6**T TTT TTT 3’ after cleavage and concentration.

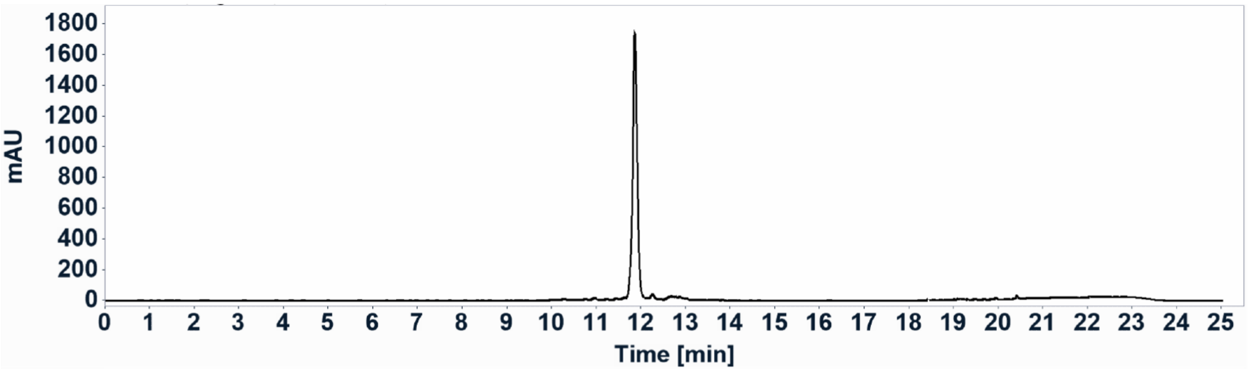

HPLC Method	1
Sequence	5' TTT TTT T**7**T TTT TTT 3'
Retention time	12.2 mins
Calculated mass	4408.9
Found mass (LCMS)	4408.5
$\varepsilon(T_7)_{260}$	57300
$\varepsilon(T_7XT_7)_{260}$	114600
Coupling time	2 x 60 s
$A(T_7)$	101
$A(T_7XT_7)$	9925
Coupling Yield	98.0%

HPLC chromatogram (260 nm) of crude 5' TTT TTT T**7**T TTT TTT 3' after cleavage and concentration.

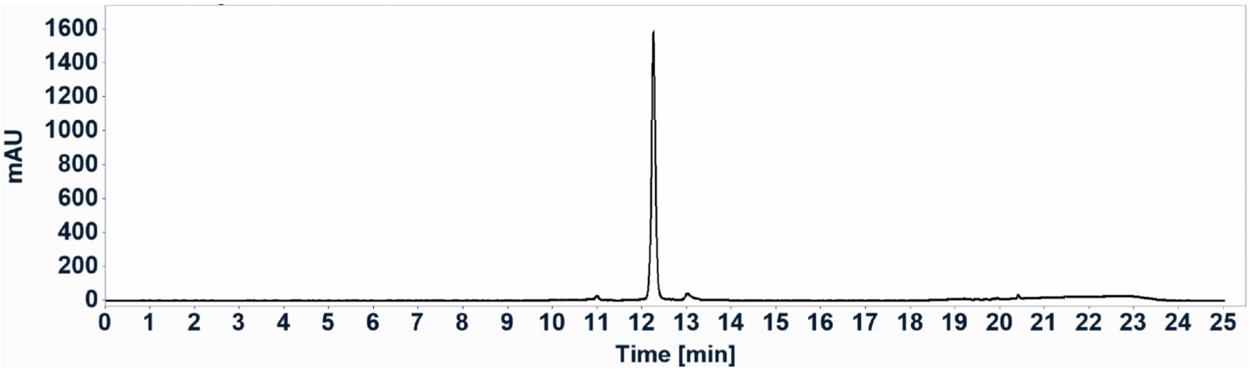

HPLC Method	1
Sequence	5' **8**TT TTT TT 3'
Retention time	18.7 mins
Calculated mass	2395.46
Found mass (LCMS)	2395.41
$\varepsilon(T_7)_{260}$	57300
$\varepsilon(T_7XT_7)_{260}$	57300
Coupling time	2 x 60 s
$A(T_7)$	66
$A(T_7XT_7)$	6426
Coupling Yield	99.0%

HPLC chromatogram (260 nm) of crude 5' **8**TT TTT TT 3' after cleavage and concentration.

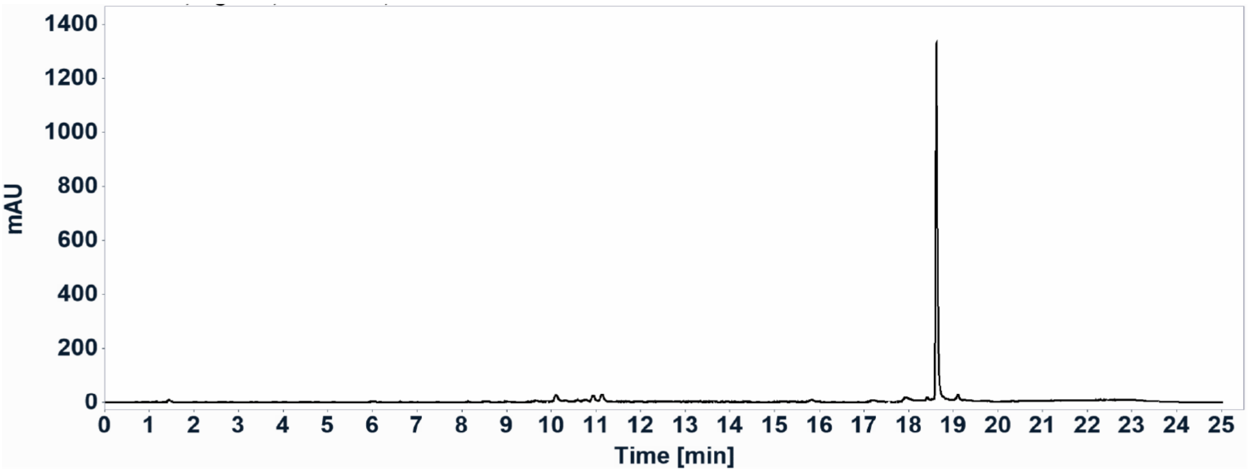

HPLC Method	1
Sequence	5’ **9**TT TTT TT 3’
Retention time	12.8 mins
Calculated mass	2304.6
Found mass (MALDI)	2309.8
$\varepsilon(T_7)_{260}$	57300
$\varepsilon(T_7XT_7)_{260}$	57300
Coupling time	2 x 60 s
$A(T_7)$	27
$A(T_7XT_7)$	4795
Coupling Yield	99.4%

HPLC chromatogram (260 nm) of crude 5’ **9**TT TTT TT 3’ after cleavage and concentration.

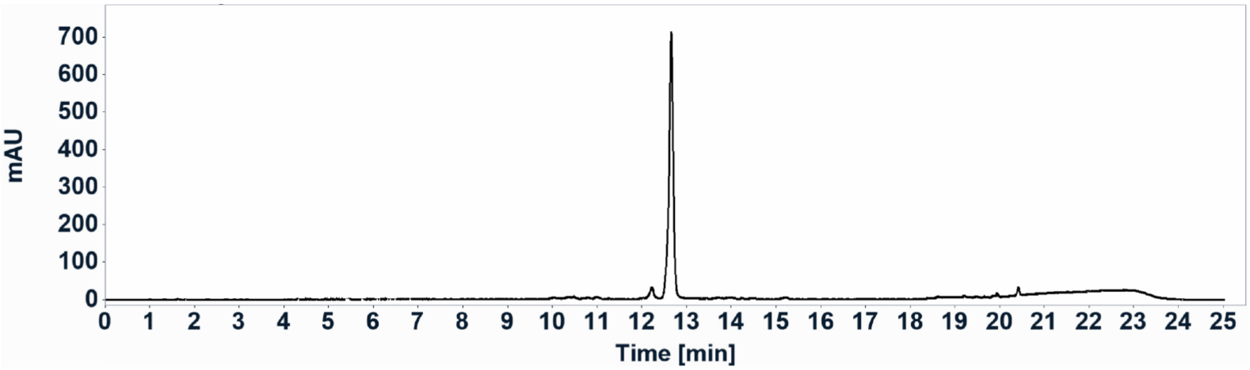

Oligonucleotide Synthesis

The synthetic cycle described in Table S5 with optimal residence times for the given alcohols according to Table S6 was used for the synthesis of the phosphoramidites. The eluate was collected for 6 void volumes and concentrated under reduced pressure. The residue was redissolved in MeCN (1.0 mL) to reach a final concentration of 0.1 M. Standard coupling conditions (Table S7) were used for phosphoramidites synthesised by conventional methods.

All phosphoramidites was synthesised by the flow-based method described herein and used for the synthesis of the following sequence:

51-mer Sequence: 5'CCG CTT TCT AGT TCG TCC TCC ATA ATT AAT TTC CTA GAG TCC TAC GTG CTC 3'

It should be noted that 3'C is bound to the resin as the first nucleotide giving a total of 50 cycles. The oligonucleotides were cleaved directly from the resin by treatment with AMA for 30 mins at 65 °C. The supernatant was concentrated under reduced pressure and the residue subjected to HPLC purification.

Sequence	5'CCG CTT TCT AGT TCG TCC TCC ATA ATT AAT TTC CTA GAG TCC TAC GTG CTC 3'
Coupling time	2 x 60 s
Retention time	16.2 mins
Calculated mass	15467.9
Found mass (LCMS)	15467.6
Yield	35.2%
Average Cycle Yield	98.0%

HPLC chromatogram (260 nm) of crude 51mer after cleavage and concentration.

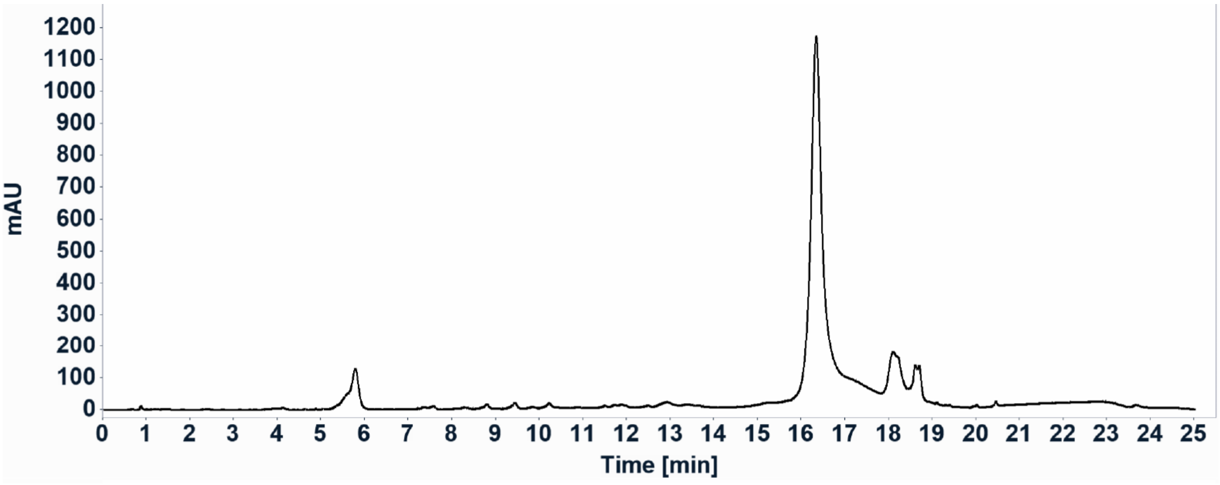

Synthesis of Reference Phosphoramidites

Non-commercial phosphoramidites were synthesised using the following protocol:

Alcohol (1.0 eq, 20 mM) was dissolved in CH_2Cl_2 and DIPEA (2.0 eq) was added along with PCl (1.5 eq) and the mixture was stirred for until full conversion (1-16 hrs) at rt The solvent was then removed under reduced pressure and the residue was subjected to flash column chromatography to give the desired compound.

Compound 1-P

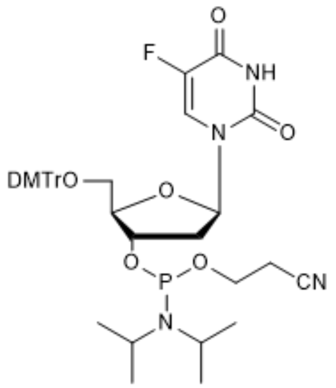

Yield: 106 mg, 0.144 mmol (79 %) from 100 mg, 0.182 mmol

^{1}H NMR (400 MHz, CD_3CN) 7.77 (dd, *J* = 12.76, 6.74 Hz, 1H), 7.47-7.42 (m, 2H), 7.36-7.28 (m, 6H), 7.27-7.21 (m, 1H), 6.91-6.84 (m, 4H), 6.17 (q, *J* = 8.70 Hz, 1H), 4.66-4.55 (m, 1H), 4.09 (dq, *J* = 15.69, 3.69 Hz, 1H), 3.77 (d, *J* = 1.92 Hz, 1H), 3.72-3.50 (m, 3H), 3.37-3.29 (m, 2H), 2.64 (t, *J* = 5.93 Hz, 1H), 2.53 (t, *J* = 5.92 Hz, 1H), 2.49-2.28 (m, 2H), 1.34-1.03 (m, 12H)

^{13}C NMR (100 MHz, CD_3CN) δ_C (ppm) 159.8, 158.0, 157.8, 149.7, 145.9, 142.7, 140.4, 136.6, 136.6, 136.5, 136.5, 131.1, 129.0, 128.9, 128.0, 125.4, 125.1, 119.4, 114.2, 114.1, 87.6, 86.2, 86.1, 86.0, 73.5, 73.3, 63.8, 59.6, 59.4, 55.9, 55.9, 44.1, 44.1, 44.0, 44.0, 40.2, 40.1, 24.9, 24.9, 24.9, 24.8, 21.0, 21.0

^{31}P NMR (162 MHz, CD_3CN) δ_P (ppm) 148.15, 148.08.

^{19}F NMR (376 MHz, CD_3CN) δ_F (ppm) -167.16, -167.19

HRMS (ESI) *m/z* $[M+Na]^+$ calc. for $C_{39}H_{47}FN_4O_8PNa$ 749.3110, found 749.3116

Compound 4-P

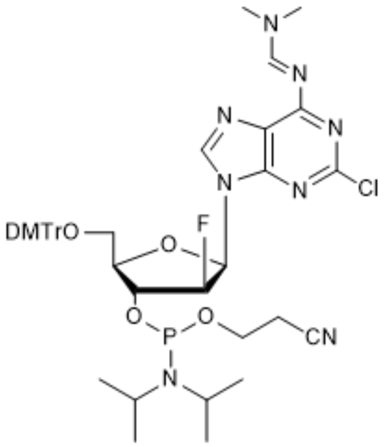

Yield: 88 mg, 0.102 mmol (90% yield) from 75 mg, 0.114 mmol

^{1}H NMR (400 MHz, CD_3CN) δ_H (ppm) 8.86 (s, 1H), 8.07 (dd, *J* = 6.85, 4.75 Hz, 1H), 7.45 (t, *J* = 6.51 Hz, 1H), 7.35-7.19 (m, 7H), 6.86-6.77 (m, 4H), 6.38 (2xdd, *J* = 4.45, 2.15 Hz 4.49, 1.58 Hz, 1H), 5.34 (2xdt, *J* = 19.57, 3.45 Hz 19.9, 3.86 Hz, 1H), 4.89-4.86 (m, 1H), 4.24-4.17 (m, 1H), 3.86-3.78 (m, 1H), 3.76-3.72 (m, 6H), 3.69-3.34 (m, 5 or 6H), 3.18 (s, 3H), 3.16 (s, 3H), 2.57 (2xt, *J* = 6.00 Hz 6.03 Hz, 2H), 1.20-1.02 (m, 12H).

^{13}C NMR (100 MHz, CD_3CN) δ_C (ppm) 161.7, 159.7, 159.7, 154.3, 154.3, 153.6, 153.6, 146.0, 146.0, 142.6, 142.5, 142.4, 142.4, 136.7, 136.7, 136.7, 136.6, 131.1, 131.0, 131.0, 130.9, 129.0, 128.9, 128.8, 128.8, 127.9, 127.8, 125.6, 125.5, 119.4, 119.3, 114.0, 114.0, 96.9, 96.8, 94.9, 94.9, 87.2, 87.1, 83.4, 83.3, 83.2, 83.0, 82.8, 82.8, 77.5, 77.4, 77.3, 77.1, 76.7, 76.6, 76.5, 76.3, 64.1, 63.8, 59.9, 59.8, 59.8, 59.6, 55.9, 55.9, 44.2, 44.2, 44.1, 44.1, 41.8, 35.5, 24.9, 24.9, 24.8, 24.8, 21.0, 20.9, 20.9.

^{31}P NMR (162 MHz, CD_3CN) δ_P (ppm) 150.26, 150.22, 150.18.

^{19}F NMR (376 MHz, CD_3CN) δ_F (ppm) -197.52, -197.54, -197.63, -197.64.

HRMS (ESI) *m/z* $[M+Na]^+$ calc. for $C_{43}H_{51}ClFN_8O_6PNa$ 883.3234, found 883.3237.

Compound 5-P

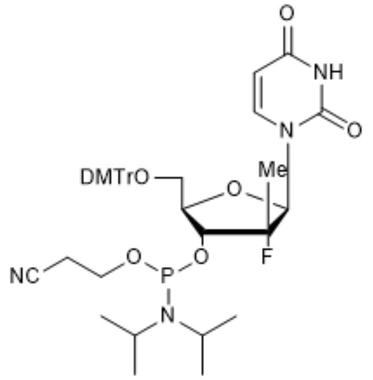

Yield: 50 mg, 0.066 mmol (74%) from 50 mg, 0.089 mmol

^{1}H NMR (400 MHz, CD_3CN) δ_H (ppm) 9.19 (s, 1H), 7.90 (dd, *J* = 11.48, 8.26 Hz, 1H), 7.43 (d, *J* = 7.66 Hz, 2H), 7.36-7.24 (m, 6 or 7H), 6.92-6.86 (m, 4H), 6.13 (d, *J* = 18.93 Hz, 1H), 5.06 (dd, *J* = 11.52, 8.25 Hz, 1H), 4.50-4.30 (m, 1H), 4.11 (t, *J* = 9.68 Hz, 1H), 3.89-3.81 (m, 1H), 3.77 (d, *J* = 2.43 Hz, 6H), 3.72-3.44 (m, 6H), 2.67 (t, *J* = 5.75 Hz, 1H), 2.44 (t, *J* = 4.46 Hz, 1H), 1.45 (dd, *J* = 22.58, 1.98 Hz, 3H), 1.19-0.97 (m, 12H).

^{13}C NMR (100 MHz, CD_3CN) δ_C (ppm) 163.6, 159.9, 159.8, 151.5, 145.7, 140.9, 136.4, 136.3, 136.3, 136.2, 131.4, 131.3, 131.3, 129.3, 129.2, 129.0, 129.0, 128.1, 128.1, 119.6, 119.4, 114.2, 114.1, 103.1, 103.1, 102.5, 102.3, 87.8, 7.8, 81.3, 81.2, 81.1, 74.7, 74.6, 74.2, 61.8, 61.4, 59.4, 59.2, 59.0, 58.9, 55.9, 55.9, 44.1, 44.0, 25.0, 24.9, 24.9, 24.8, 24.7, 21.0, 20.9, 20.9, 18.2, 18.1, 17.9, 17.9, 17.6, 17.3.

^{31}P NMR (162 MHz, CD_3CN) δ_P (ppm) 150.64, 149.31.

^{19}F NMR (376 MHz, CD_3CN) δ_F (ppm) -159.72.

HRMS (ESI) *m/z* $[M+H]^+$ calc. for $C_{40}H_{49}FN_4O_8P$ 763.3267, found 763.3271

Compound 6-P

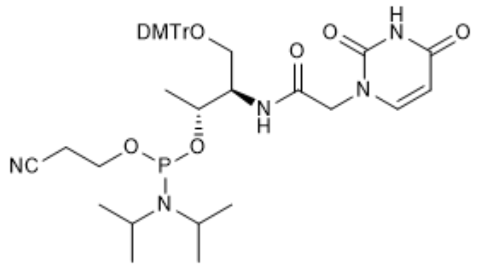

Yield: 0.95 g, 1.25 mmol (54%) from 1.30 g, 2.33 mmol

^{1}H NMR (400 MHz, CD_3CN) δ_H (ppm) 9.13 (broad s, 1H), 7.42 (d, *J* = 7.21 Hz, 2H), 7.33-7.27 (m, 7H), 7.23 (tt, *J* = 7.22 Hz, 1.16 Hz, 1H), 6.86 (d, *J* = 8.93 Hz, 4H), 6.53 (d, *J* = 9.12 Hz, 1H), 5.58 (d, *J* = 7.90 Hz, 1H), 4.38 (d, *J* = 16.40 Hz, 1H), 4.36 (d, *J* = 16.43 Hz, 1H), 4.25-4.10 (m, 2H), 3.77 (s, 6H), 3.59-3.45 (m, 4H), 3.15-3.06 (m, 2H), 2.48 (t, *J* = 5.97 Hz, 2H), 1.16-1.07 (m, 15H)

^{13}C NMR (100 MHz, CD_3CN) δ_C (ppm) 167.8, 164.4, 159.6, 151.9, 147.0, 146.9, 146.1, 137.0, 137.0, 131.0, 129.0, 128.8, 128.8, 127.7, 119.6, 114.0, 101.9, 86.8, 69.9, 69.7, 63.9, 59.5, 59.3, 55.9, 55.4, 55.4, 51.0, 43.9, 43.8, 25.0, 24.9, 24.7, 24.6, 21.0, 20.9, 19.5, 19.4

^{31}P NMR (162 MHz, CD_3CN) δ_P (ppm) 147.4, 146.9

HRMS (ESI) *m/z* $[M+Na]^+$ calc. for $C_{40}H_{51}N_5O_8P$ 760.3470, found 760.3471

2-Cyanoethyl *N*,*N*-diisopropylphosphonamidate Reference

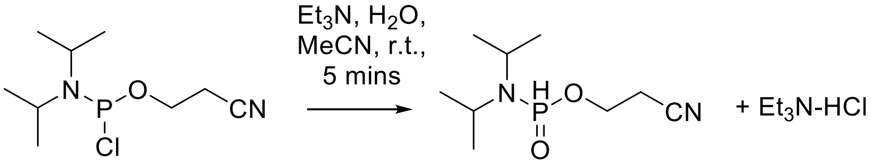

To a 4 mL flame-dried glass vial containing 2 mL dry MeCN was added PCl (22 µL, 0.10 mmol, 1.0 eq), Et_3N (70 µL, 0.50 mmol, 5.0 eq) and H_2O (9 µL, 0.50 mmol, 5.0 eq). The mixture was stirred 5 minutes at room temperature and then concentrated under reduced pressure to give the desired H-phosphonate and the triethylamine hydrochloride salt.

^{1}H NMR (400 MHz, CD_3CN) δ_H (ppm) 11.75 (s, broad, Et_3N-HCl) , 7.65+6.06 (d, $J_{P\text{-}H}$ = 636.10 Hz, 1H), 4.15-3.99 (m, 2H), 3.55-3.41 (m, 2H), 3.07-2.99 (m, 2H + Et_3N-HCl), 2.76 (t, *J* = 5.94 Hz, 2H), 1.28 (t, *J* = 7.30 Hz, Et_3N-HCl), 1.22 (d, *J* = 5.36 Hz, 6H), 1.22 (d, *J* = 5.33 Hz, 6H)

^{31}P NMR (162 MHz, CD_3CN) δ_P (ppm) 13.75

9AJ Reference

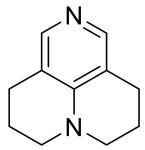

^{1}H NMR (400 MHz, CD_3CN) δ_H (ppm) 7.68 (s, 2H), 3.20 (t, *J* = 5.66 Hz, 4H), 2.62 (t, *J* = 6.33 Hz, 4H), 1.88 (quintet, *J* = 6.0 Hz, 4 H)

NMR Spectra of P(III)-loaded Resins

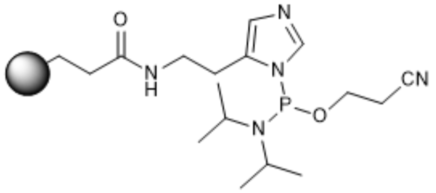

Gel Phase ^{31}P NMR (162 MHz, MeCN) of **TG-Het1** (loaded)

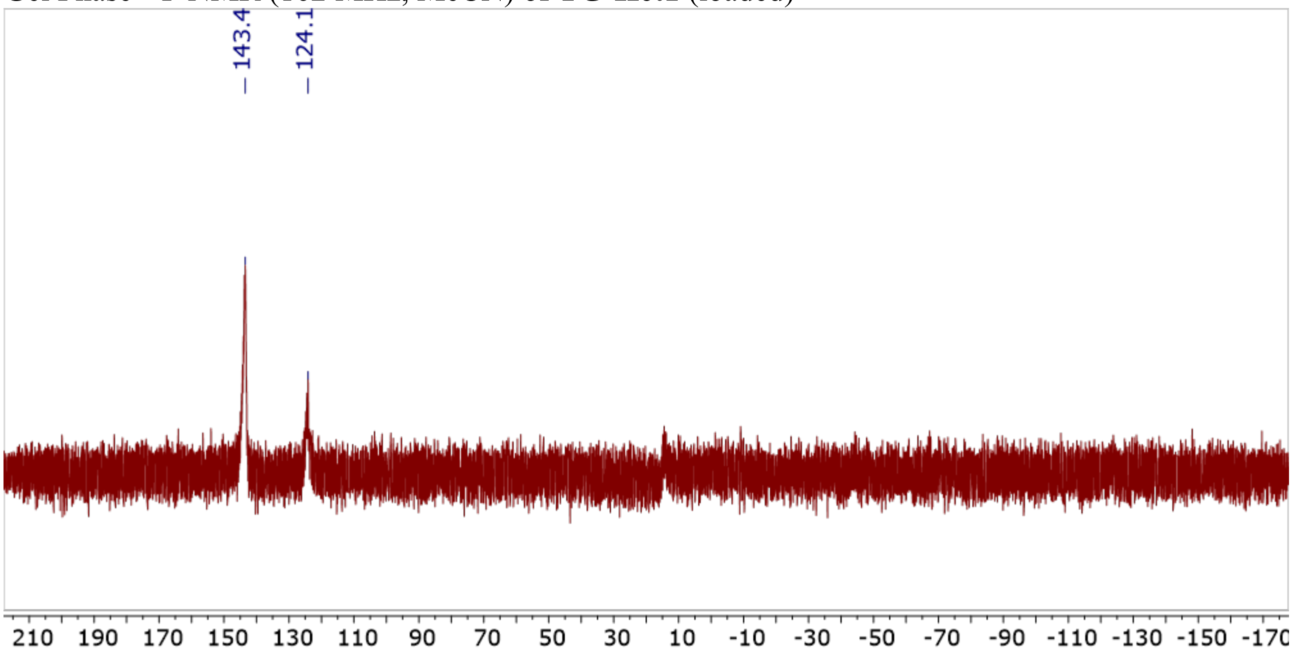

Gel Phase ^{31}P NMR (162 MHz, MeCN) of **TG-Het1** (after MeOH elution)

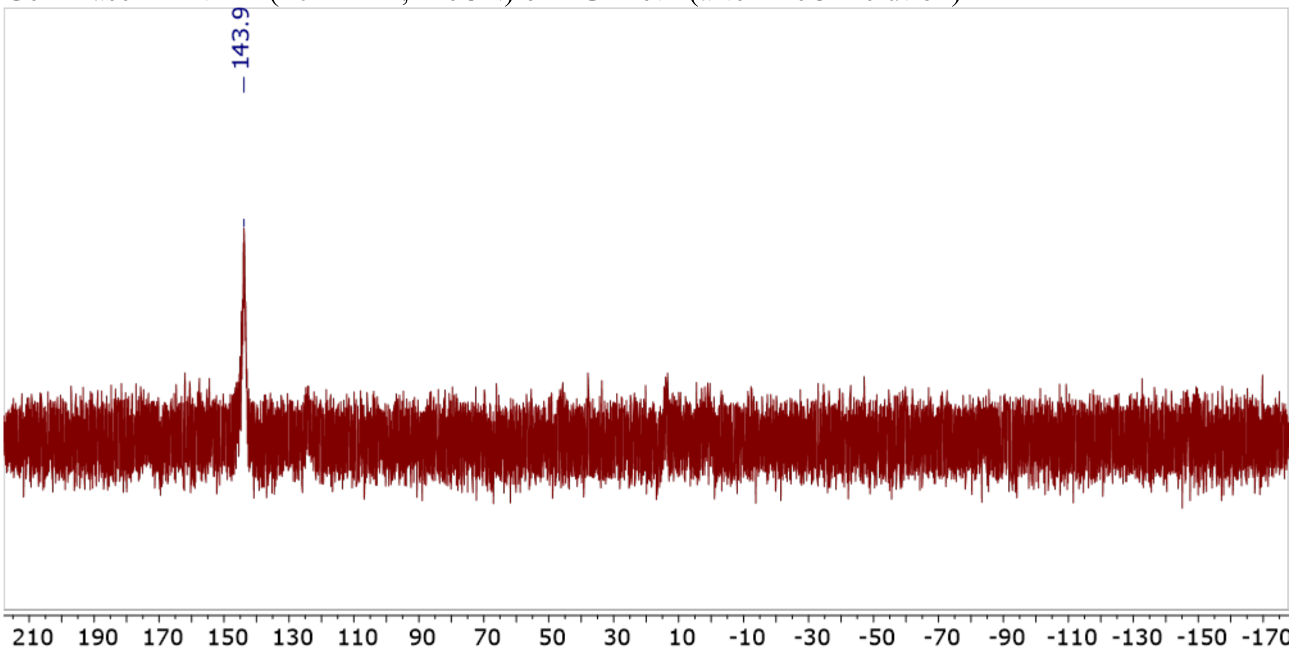

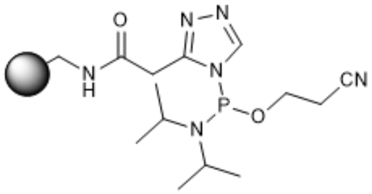

Gel Phase ^{31}P NMR (162 MHz, MeCN) of **TG-Het2** (loaded)

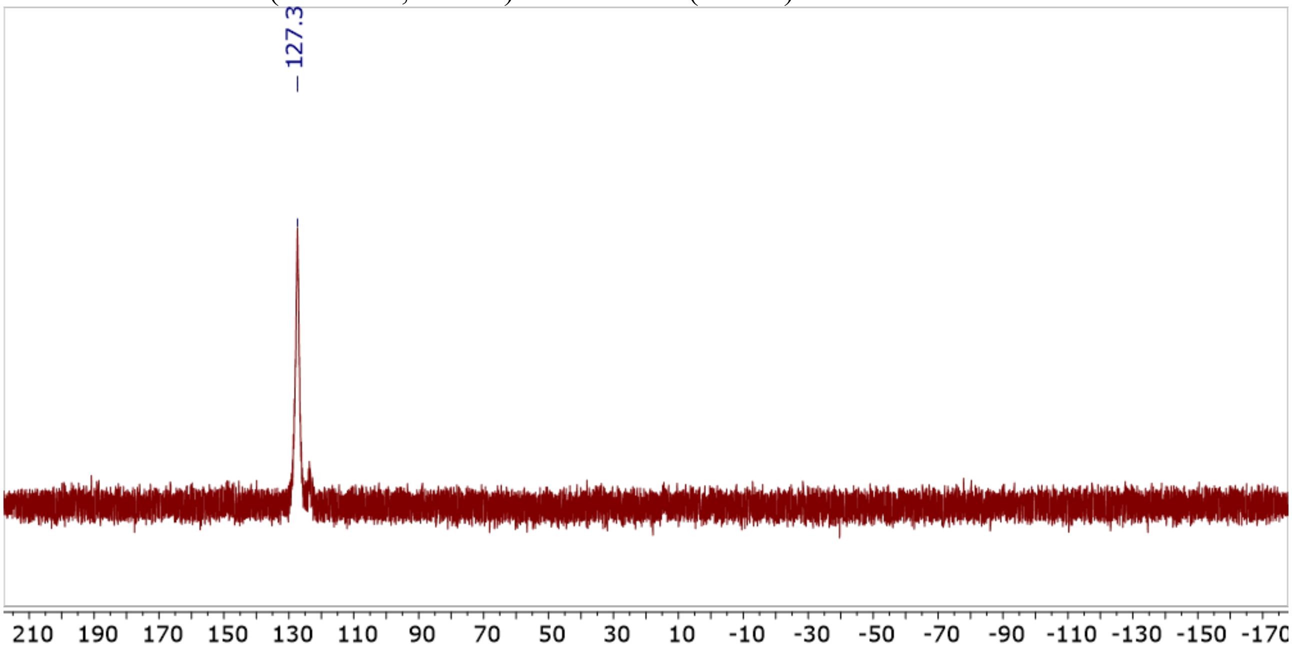

Gel Phase ^{31}P NMR (162 MHz, MeCN) of **TG-Het2** (after MeOH elution)

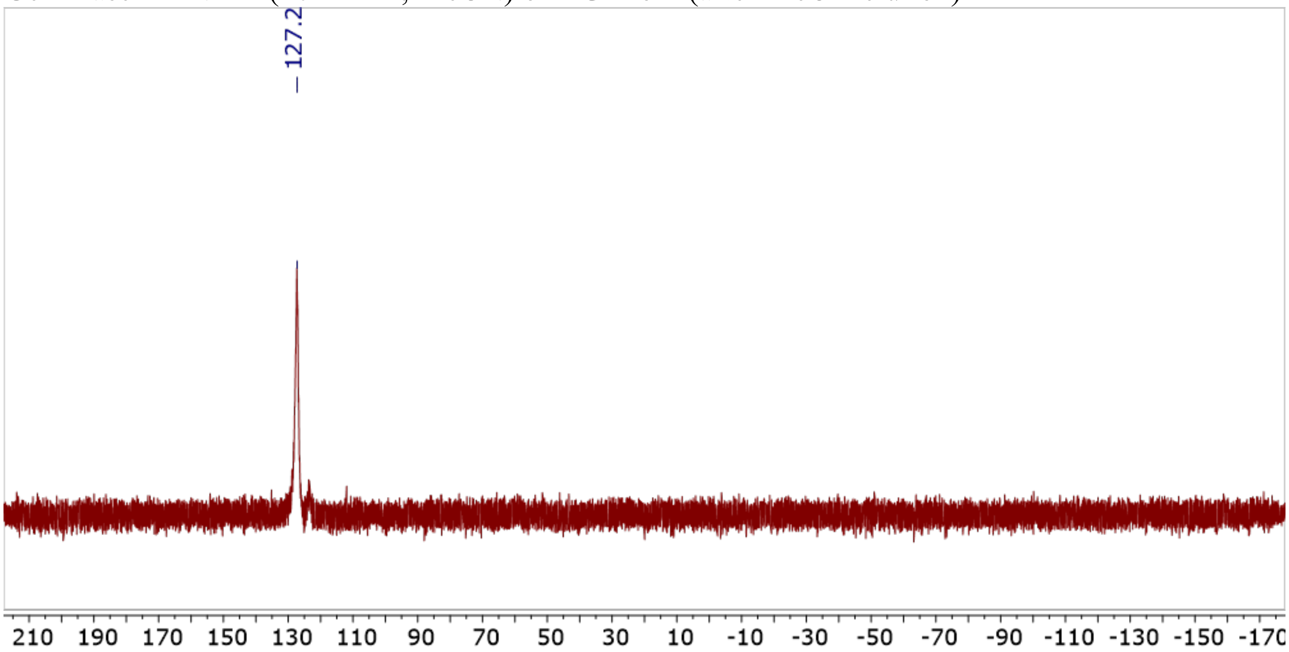

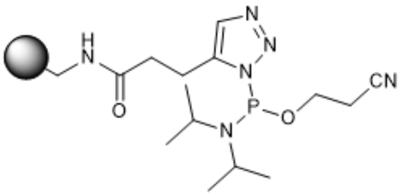

Gel Phase ^{31}P NMR (162 MHz, MeCN) of **TG-Het3** (loaded)

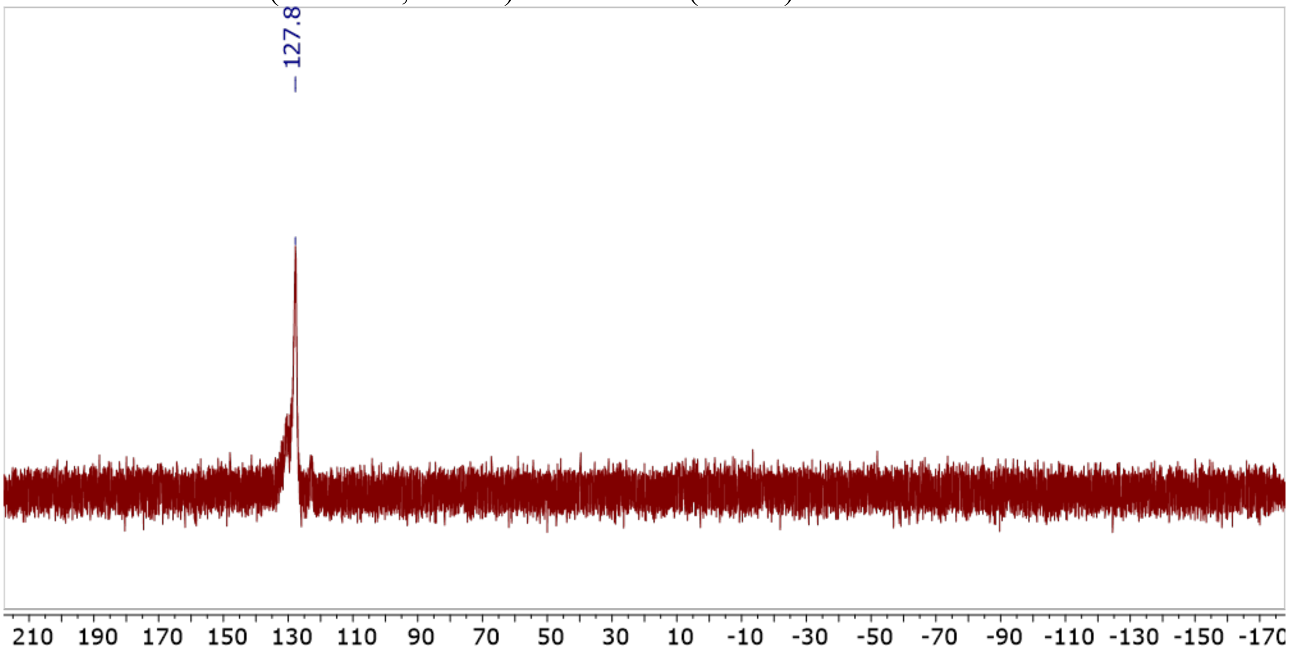

Gel Phase ^{31}P NMR (162 MHz, MeCN) of **TG-Het3** (after MeOH elution)

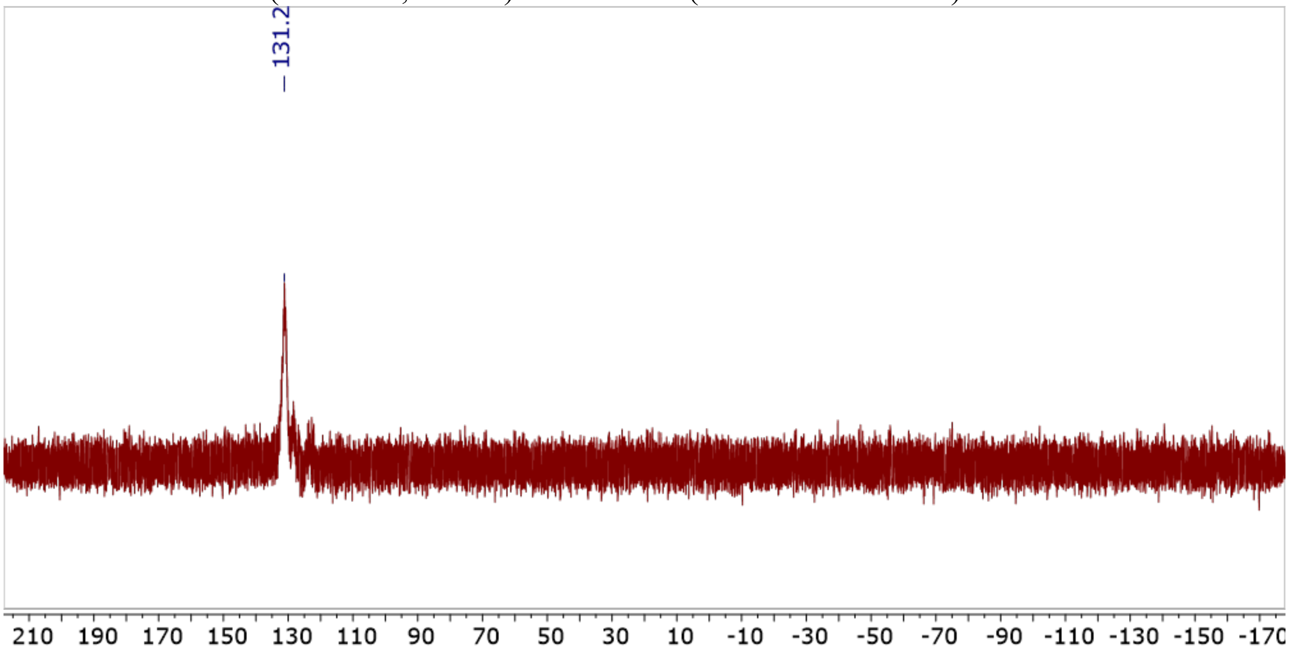

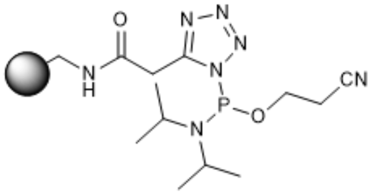

Gel Phase ^{31}P NMR (162 MHz, MeCN) of **TG-Het4** (loaded)

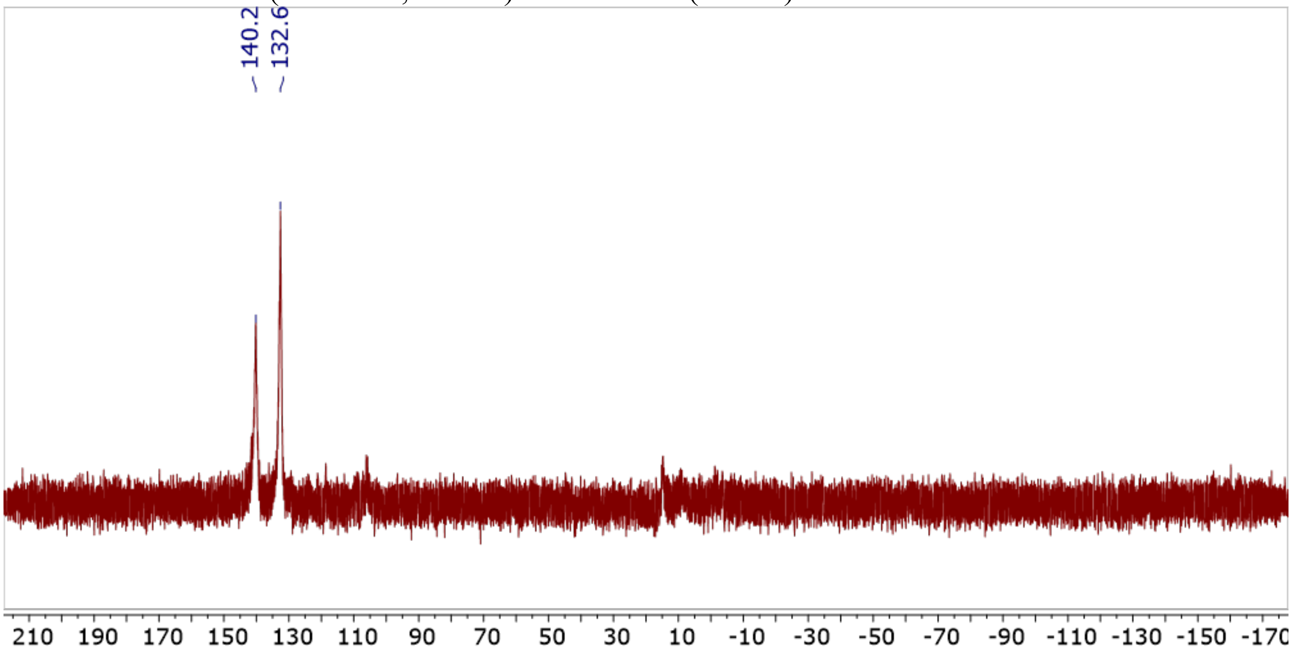

Gel Phase ^{31}P NMR (162 MHz, MeCN) of **TG-Het4** (after MeOH elution)

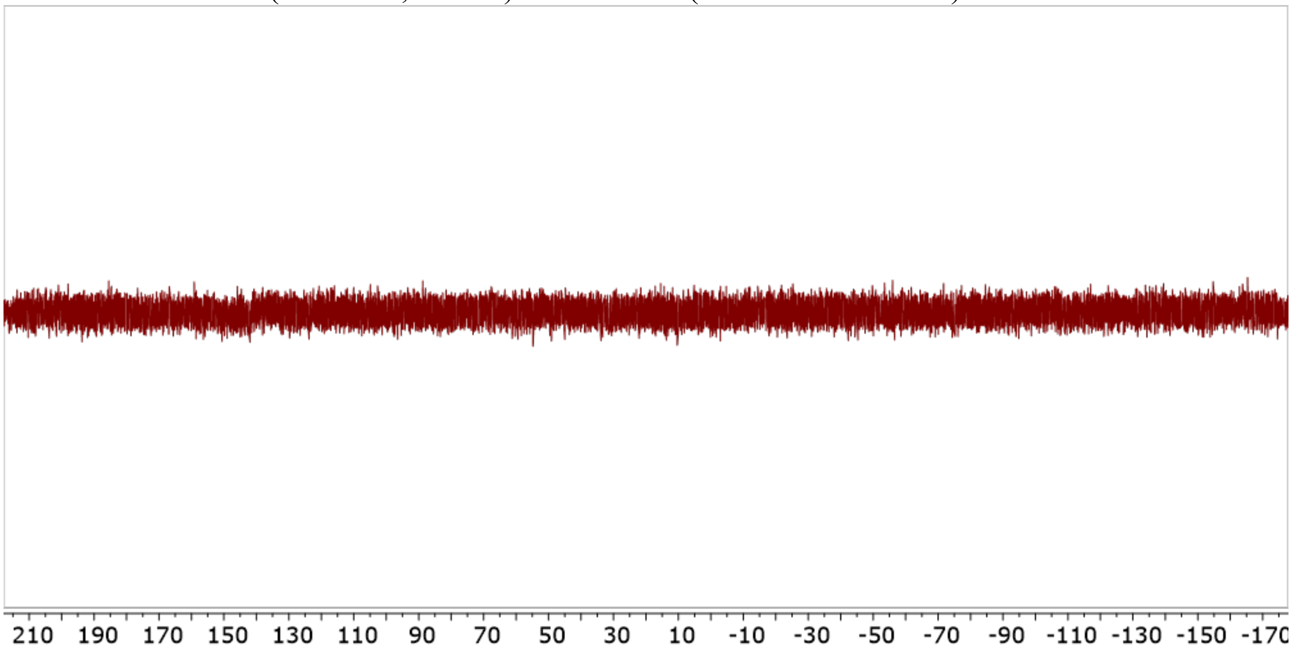

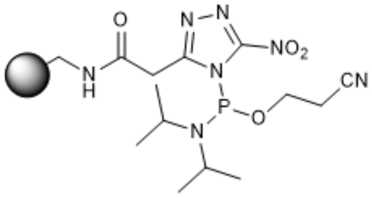

Gel Phase ^{31}P NMR (162 MHz, MeCN) of **TG-Het5.1** (loaded)

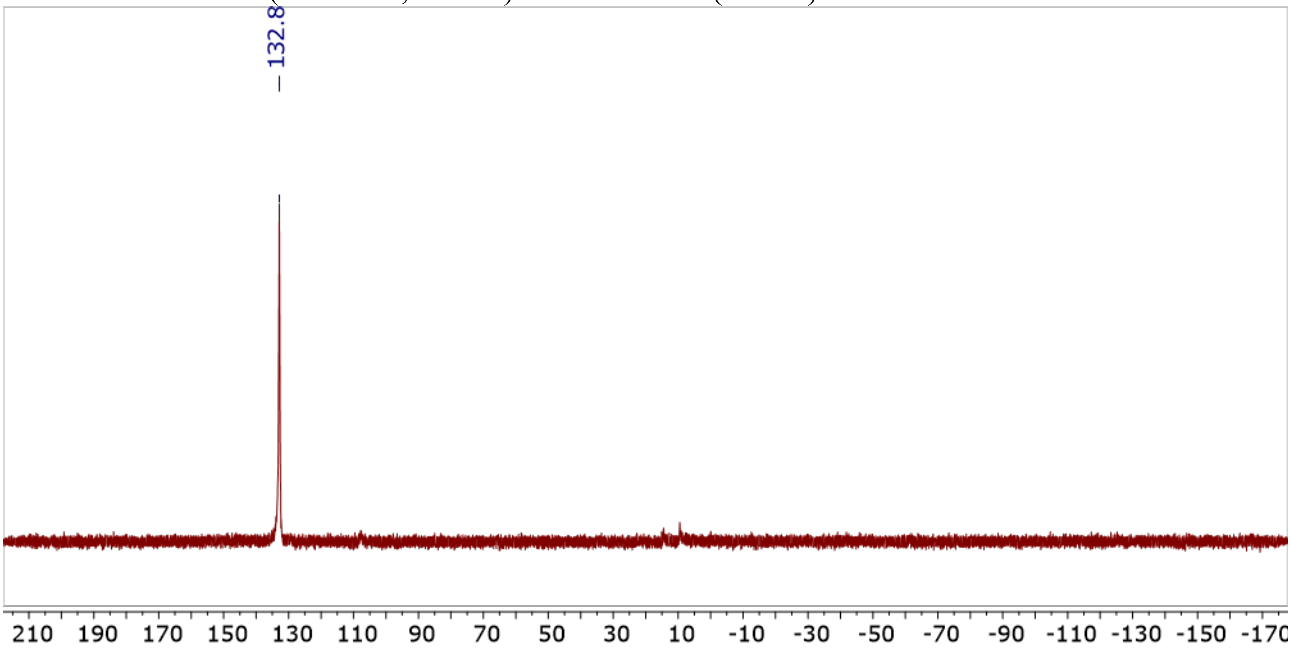

Gel Phase ^{31}P NMR (162 MHz, MeCN) of **TG-Het5.1** (after MeOH elution)

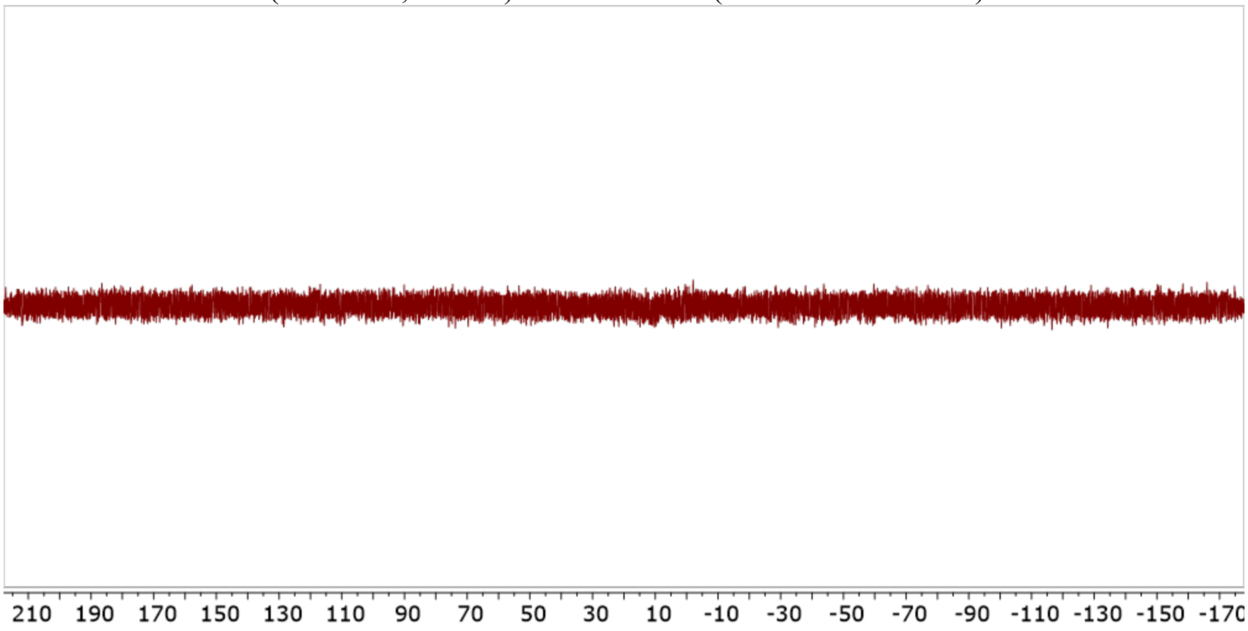

NMR Spectra of Synthesized Starting Materials and Reference Products

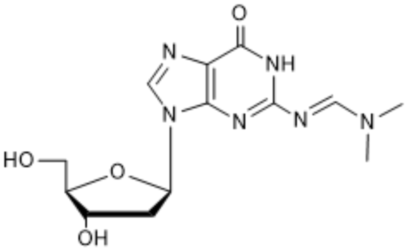

^{1}H NMR (CD_3CN) of 2-dmf-2'-Deoxyguanisine

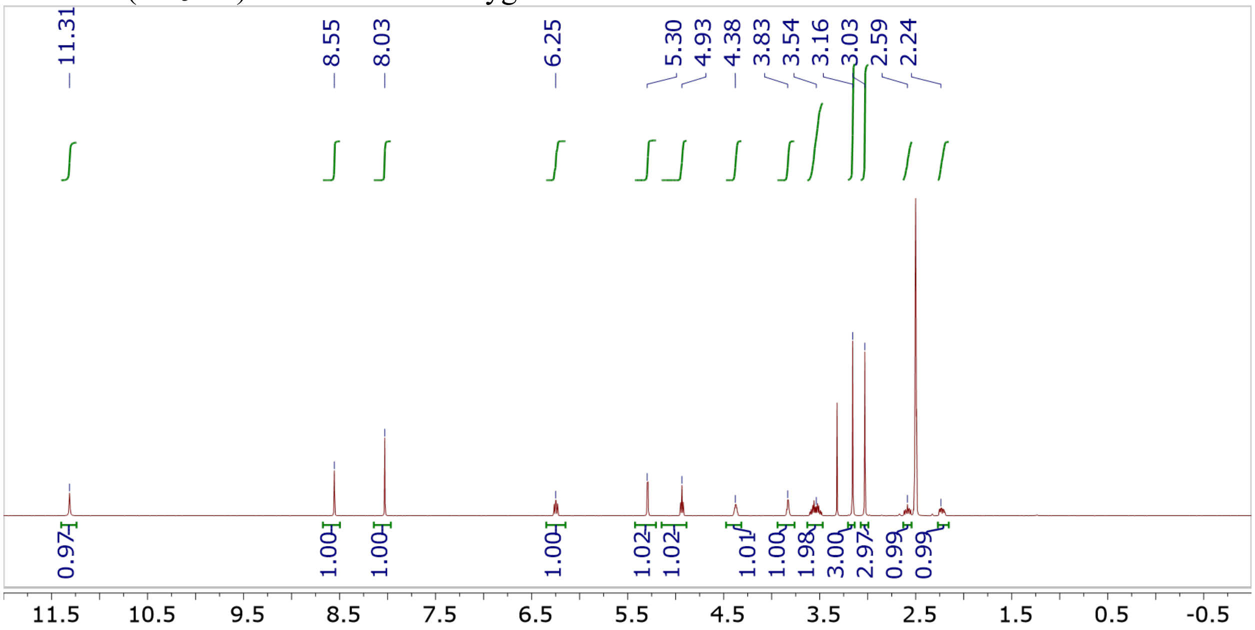

^{13}C NMR (CD_3CN) of 2-dmf-2'-Deoxyguanisine

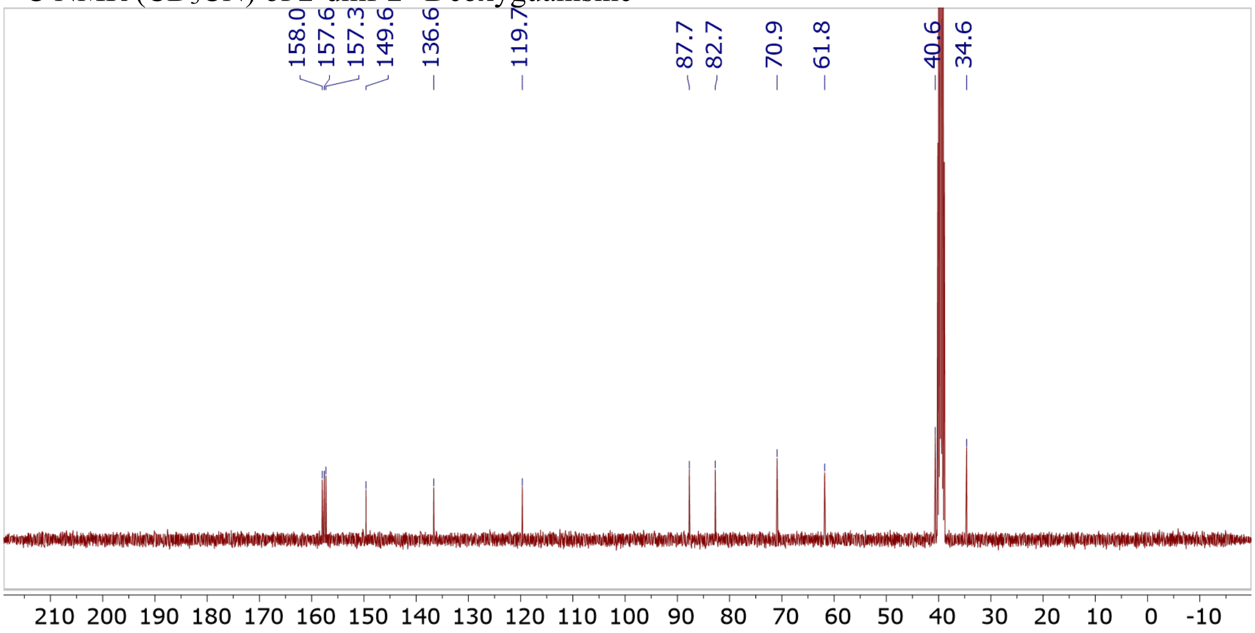

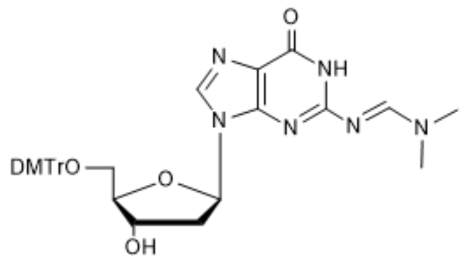

^{1}H NMR (CD_3CN) of **dmf-dG**

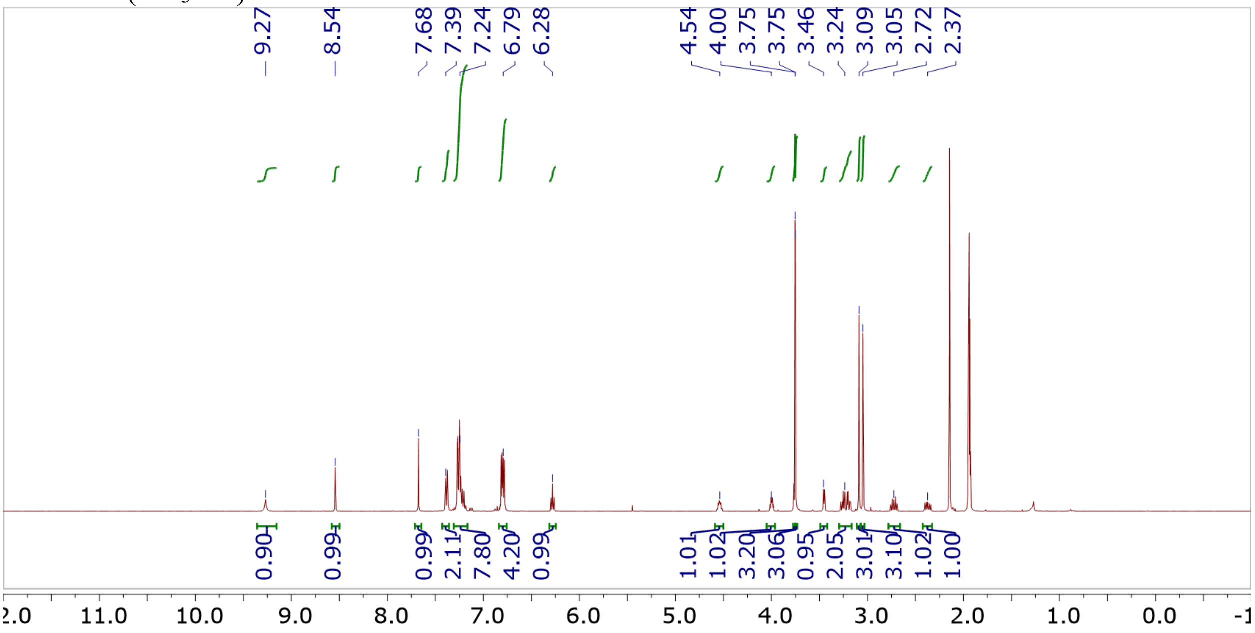

^{13}C NMR (CD_3CN) of **dmf-dG**

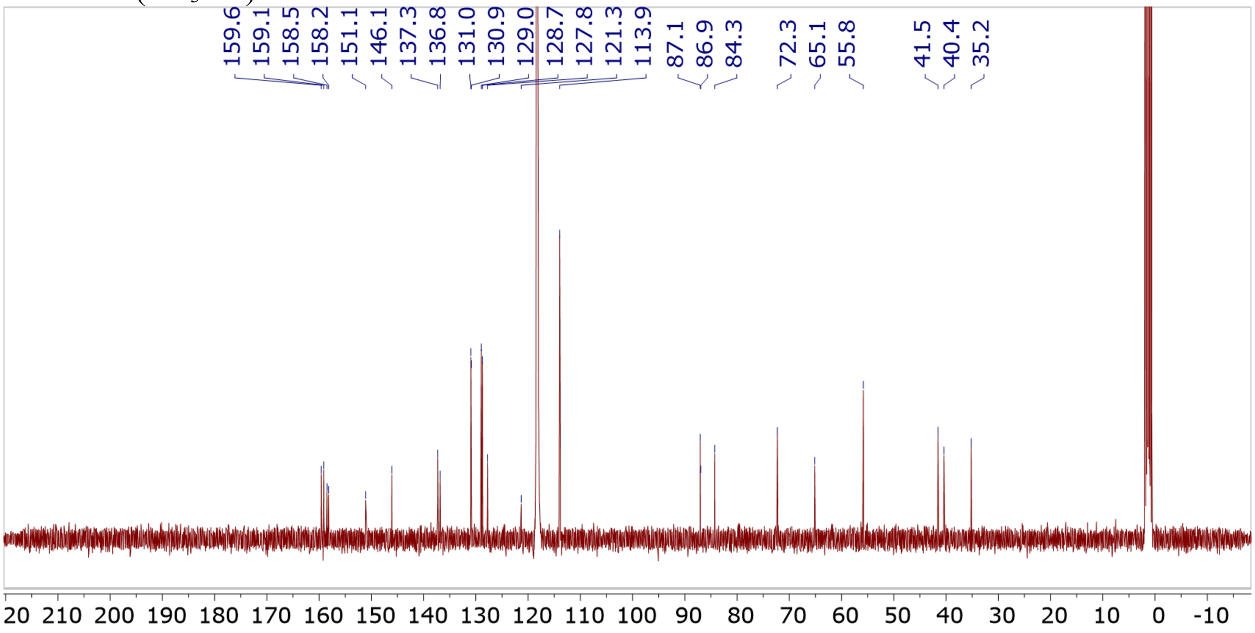

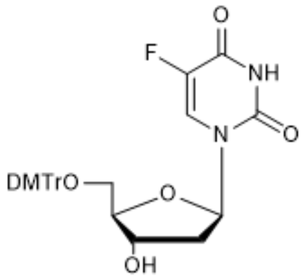

^{1}H NMR (CD_3CN) of **1**

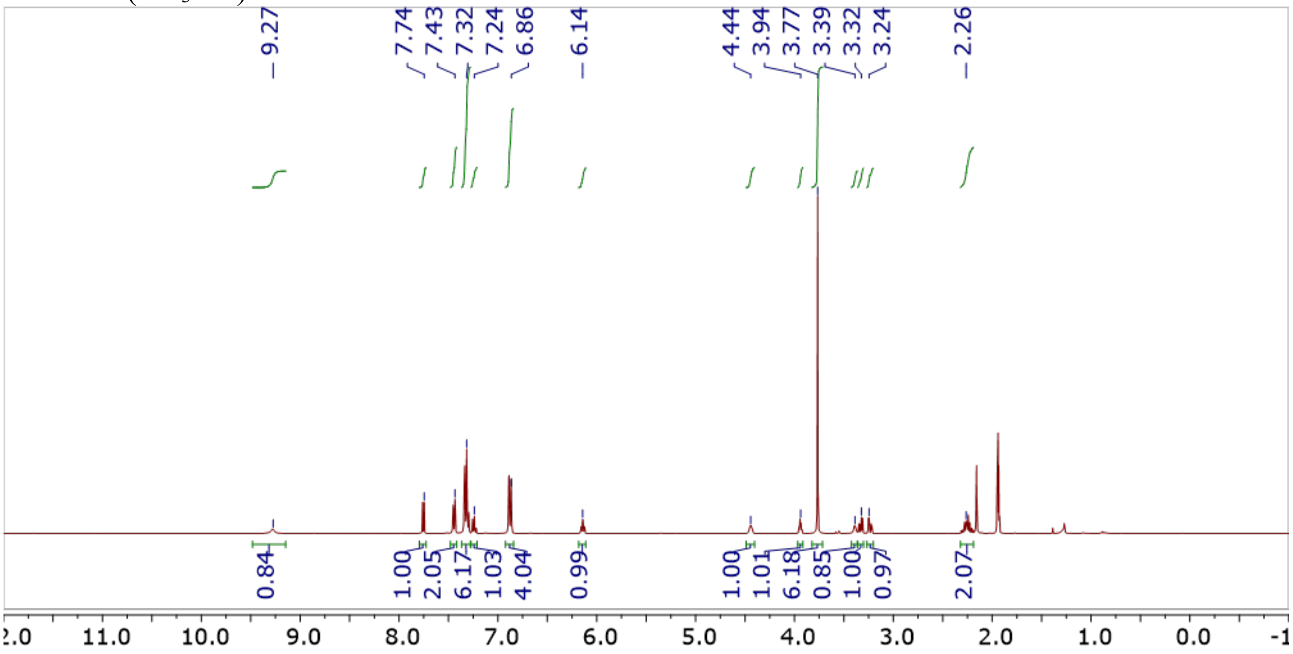

^{13}C NMR (CD_3CN) of **1**

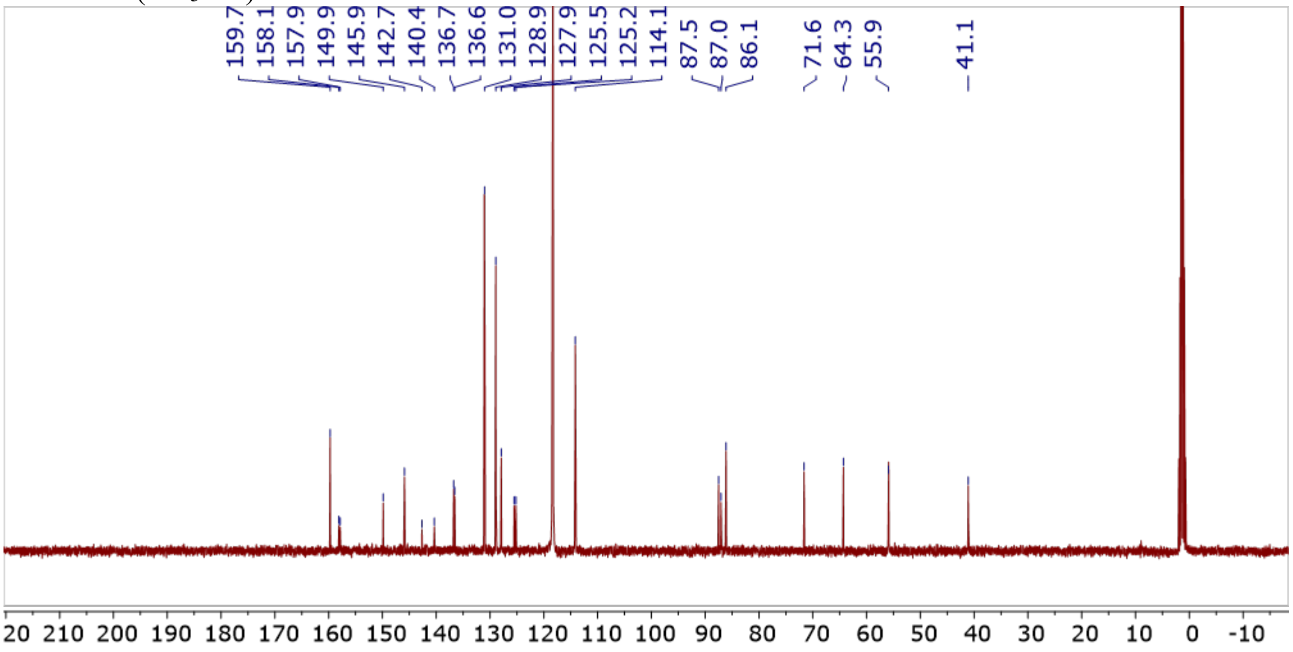

^{19}F NMR (CD_3CN) of **1**

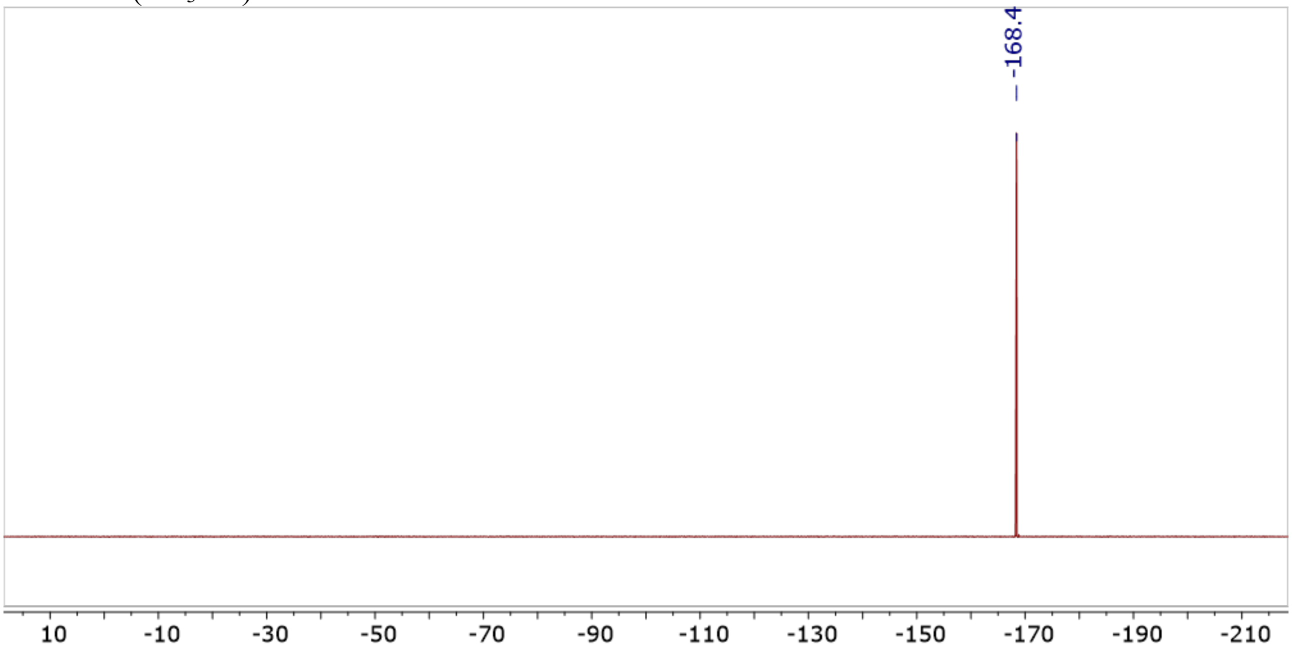

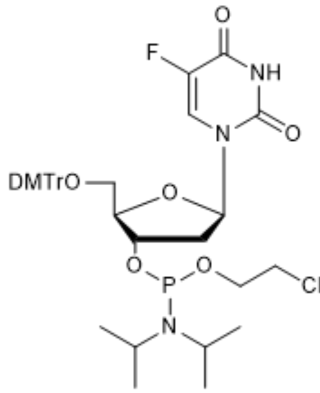

^{1}H NMR (CD_3CN) of **1-P**

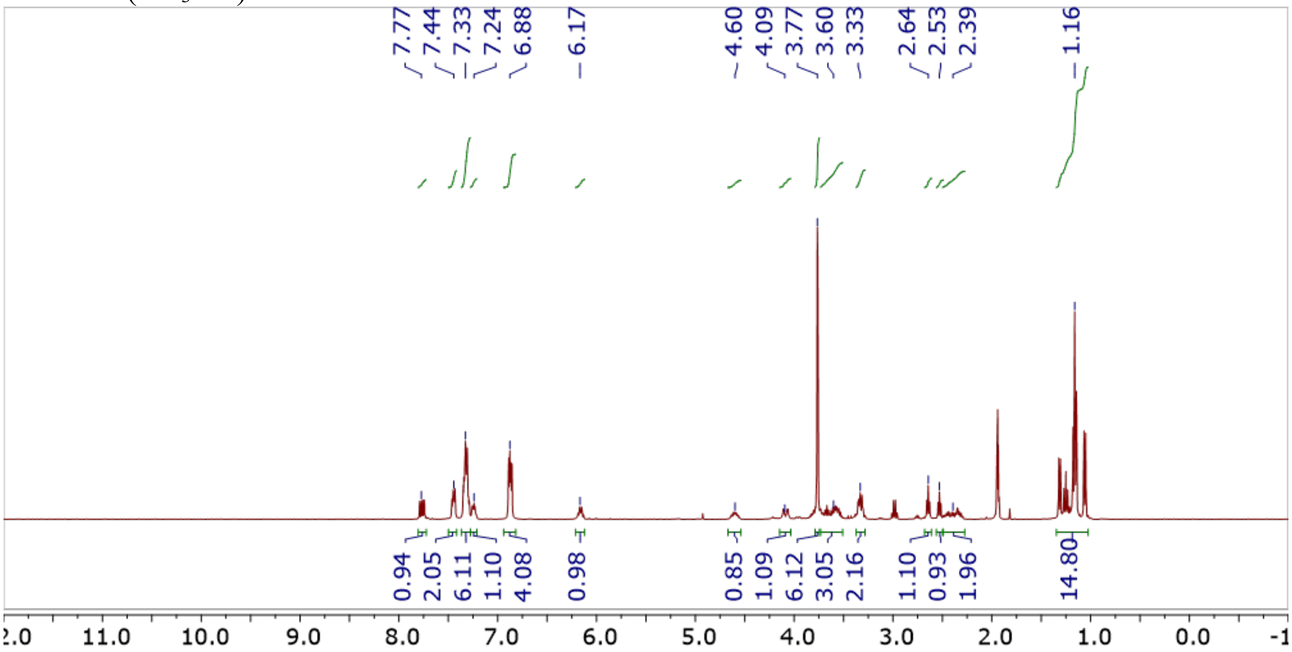

^{13}C NMR (CD_3CN) of **1-P**

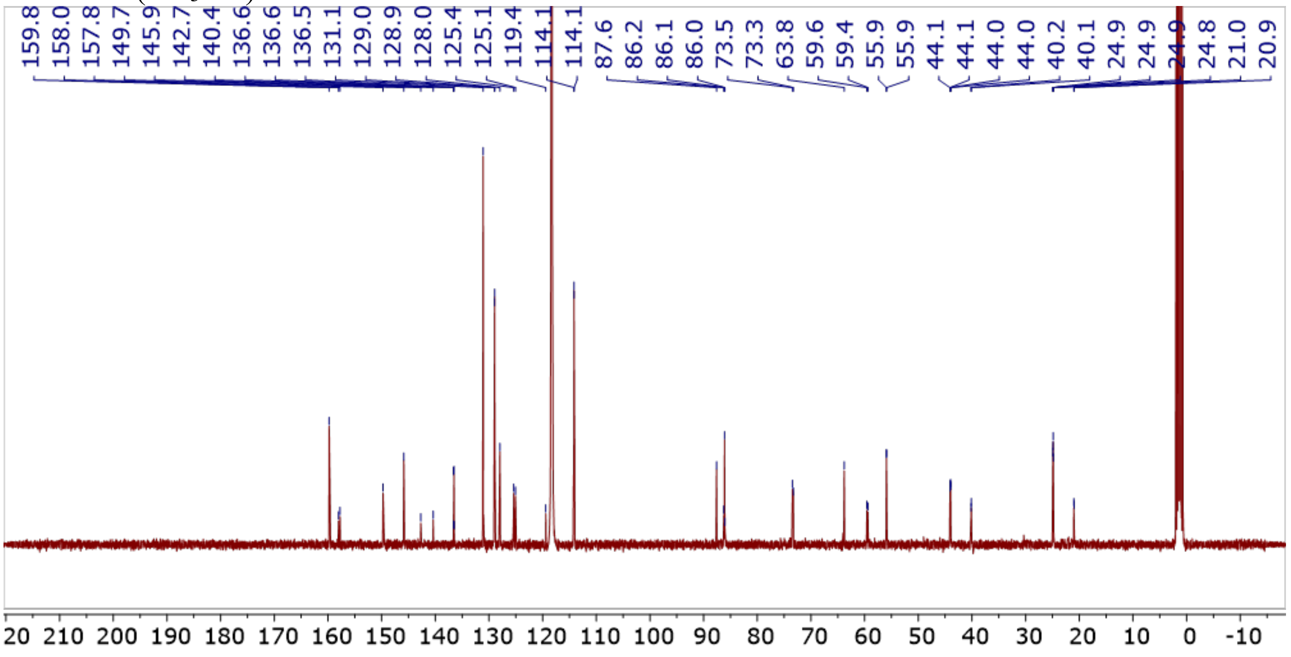

^{19}F NMR (CD_3CN) of **1-P**

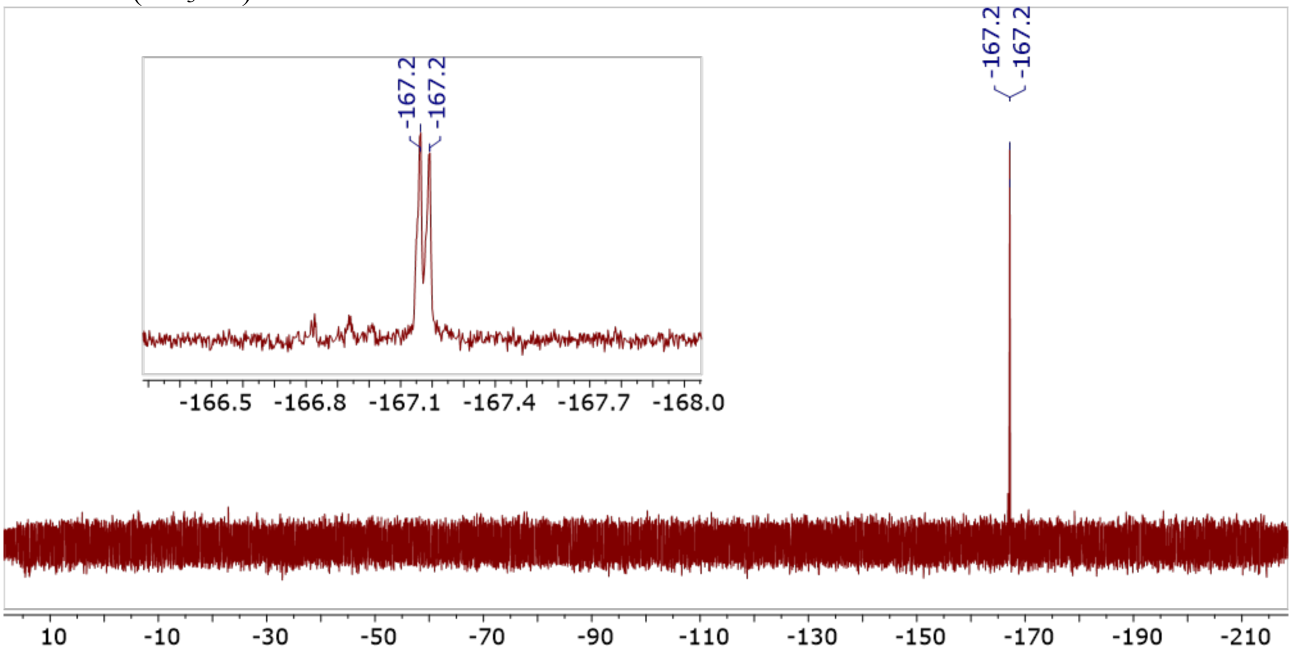

^{31}P NMR (CD_3CN) of **1-P**

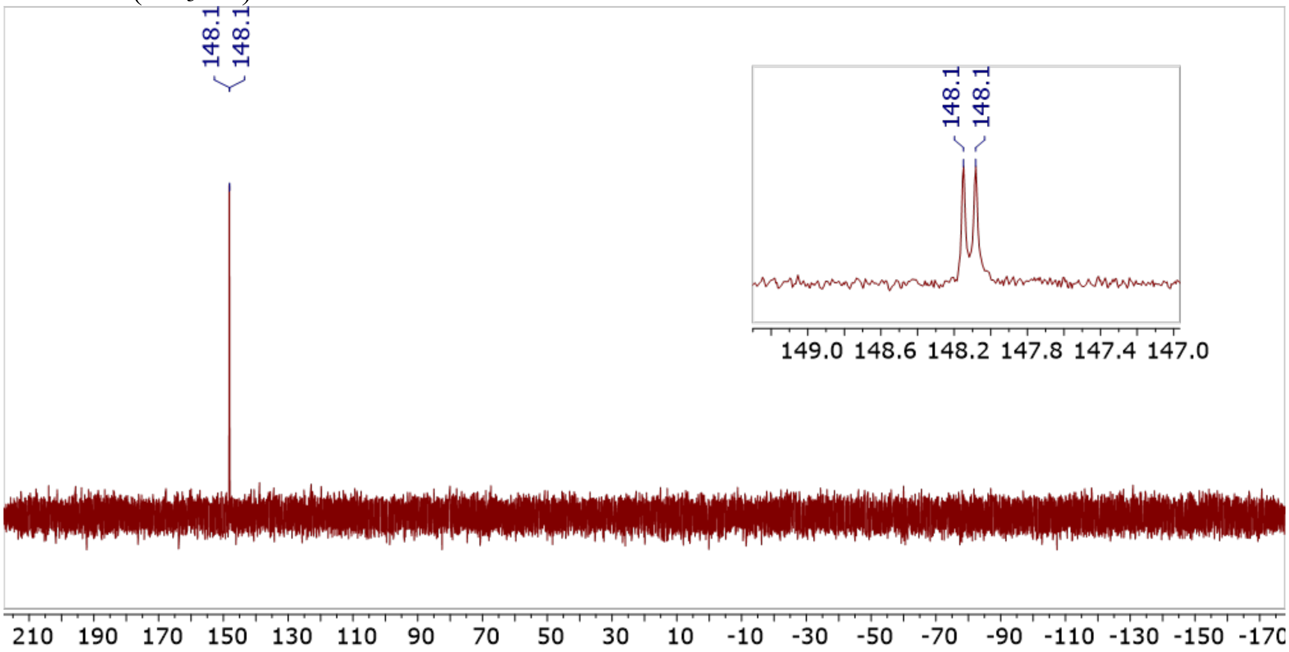

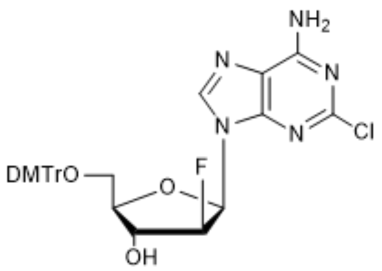

^{1}H NMR (CD_3CN) of **4.1**

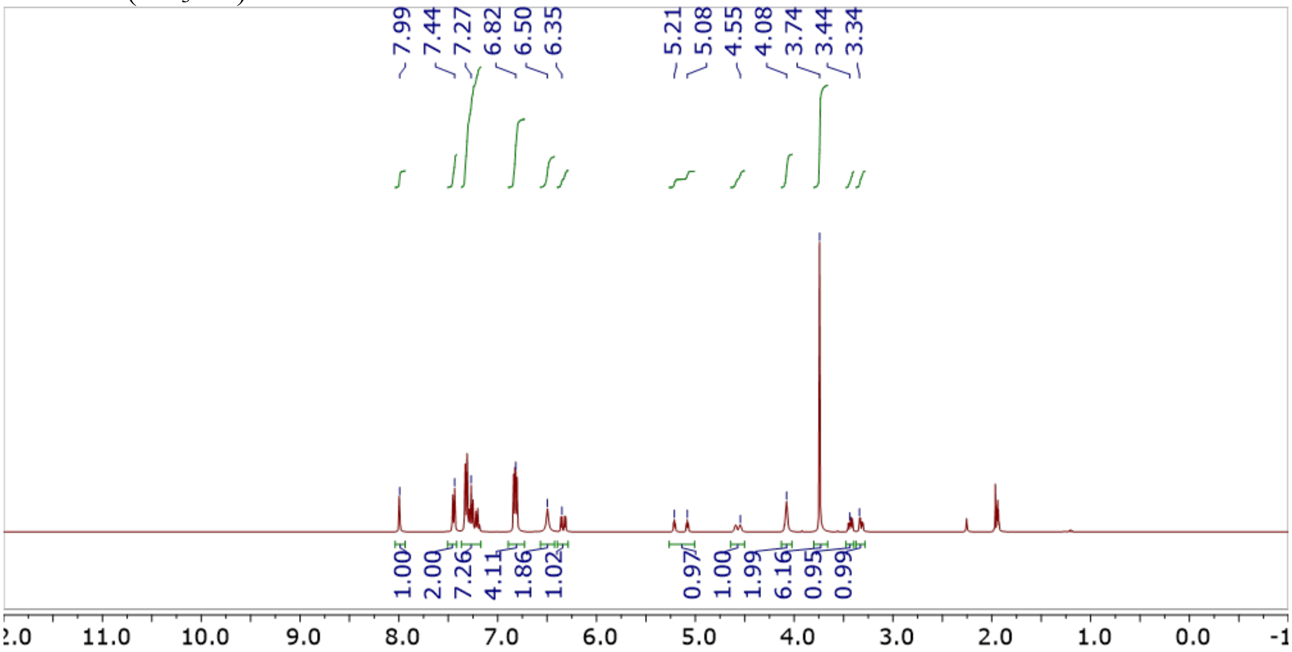

^{13}C NMR (CD_3CN) of **4.1**

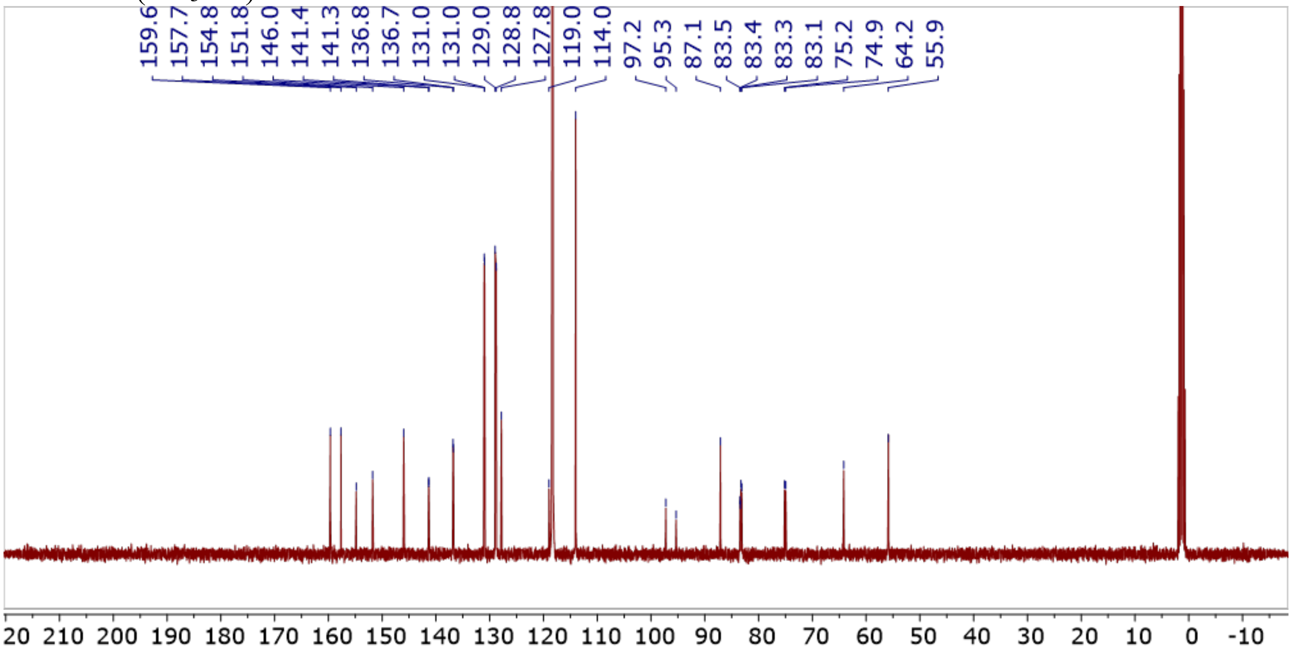

^{19}F NMR (CD_3CN) of **4.1**

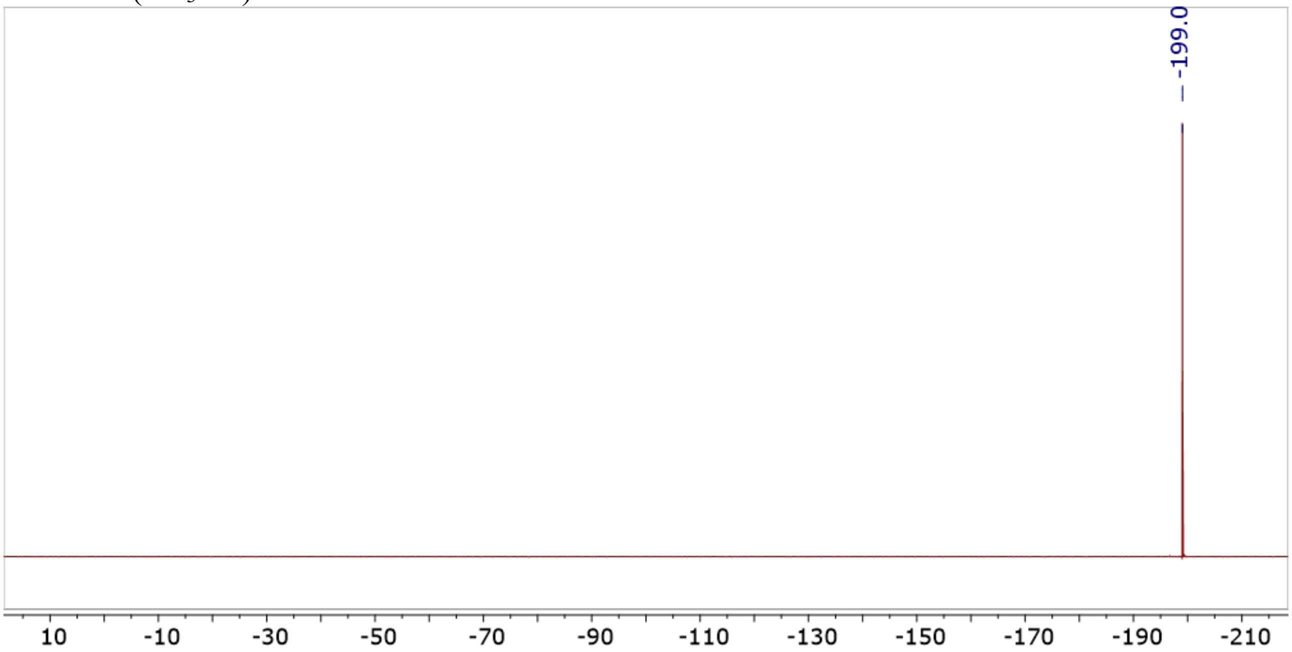

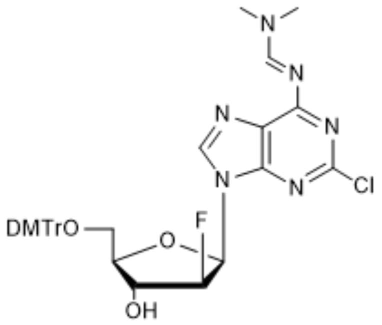

^{1}H NMR (CD_3CN) of **4**

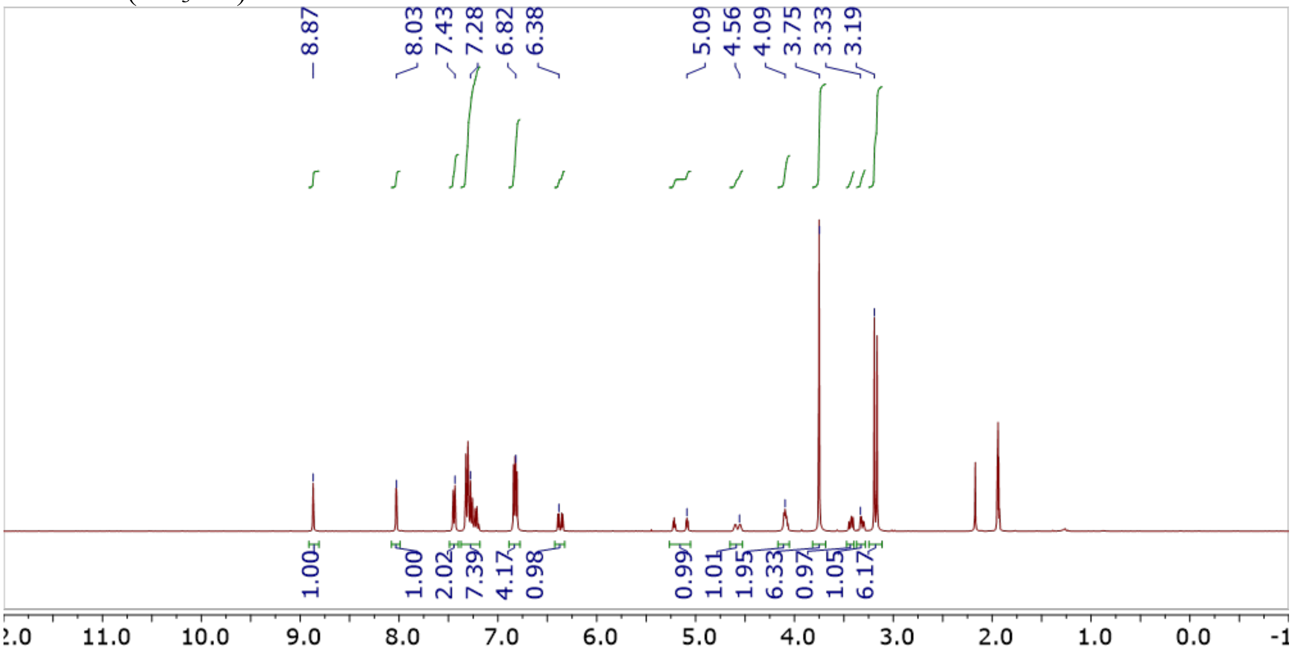

^{13}C NMR (CD_3CN) of **4**

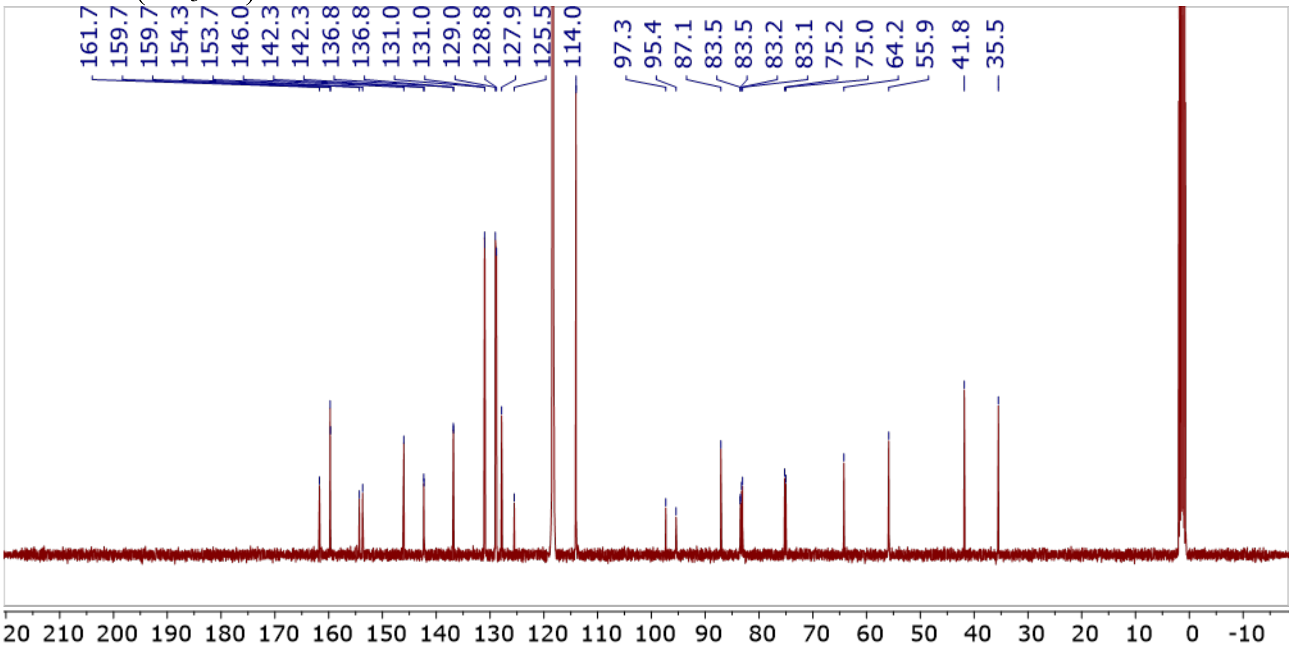

^{19}F NMR (CD_3CN) of **4**

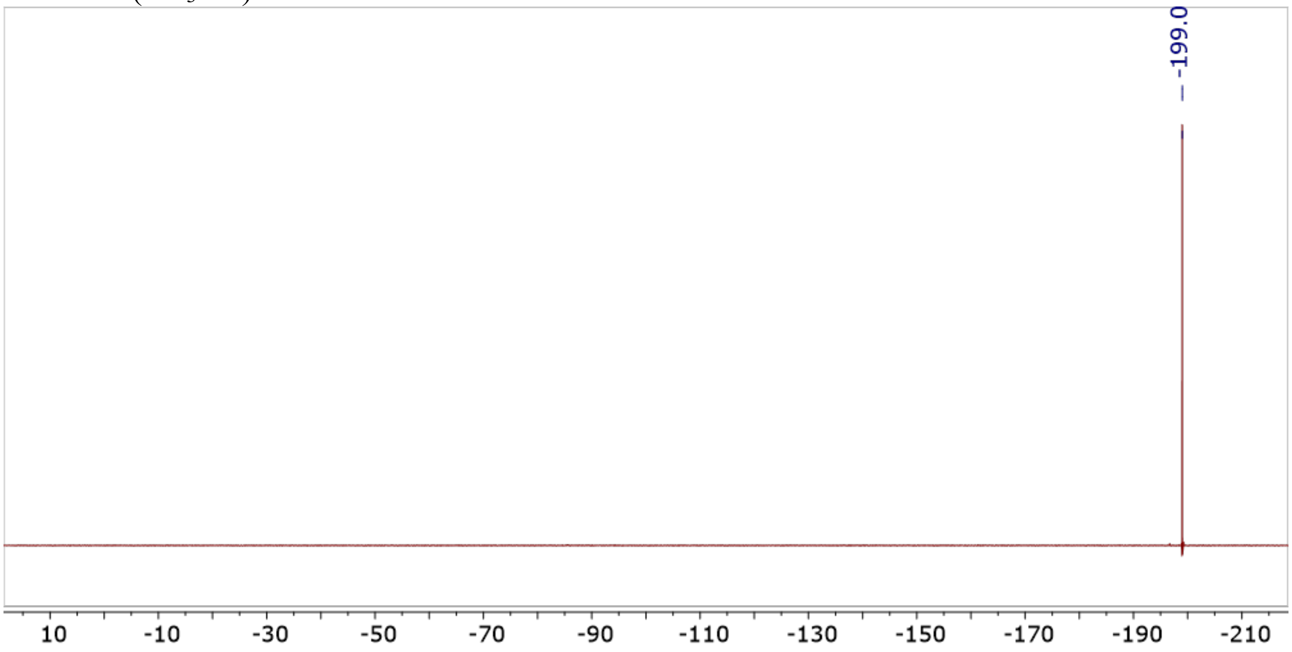

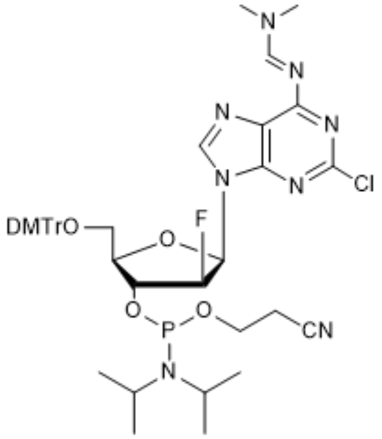

^{1}H NMR (CD_3CN) of **4-P**

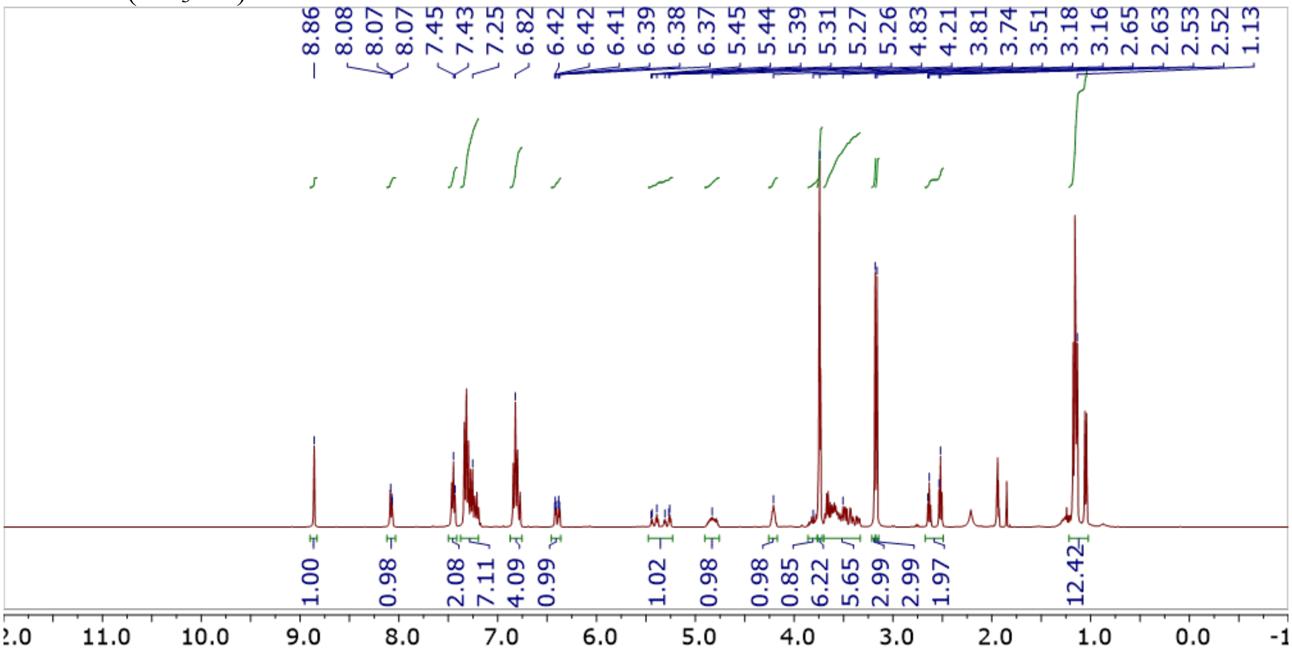

^{13}C NMR (CD_3CN) of **4-P**

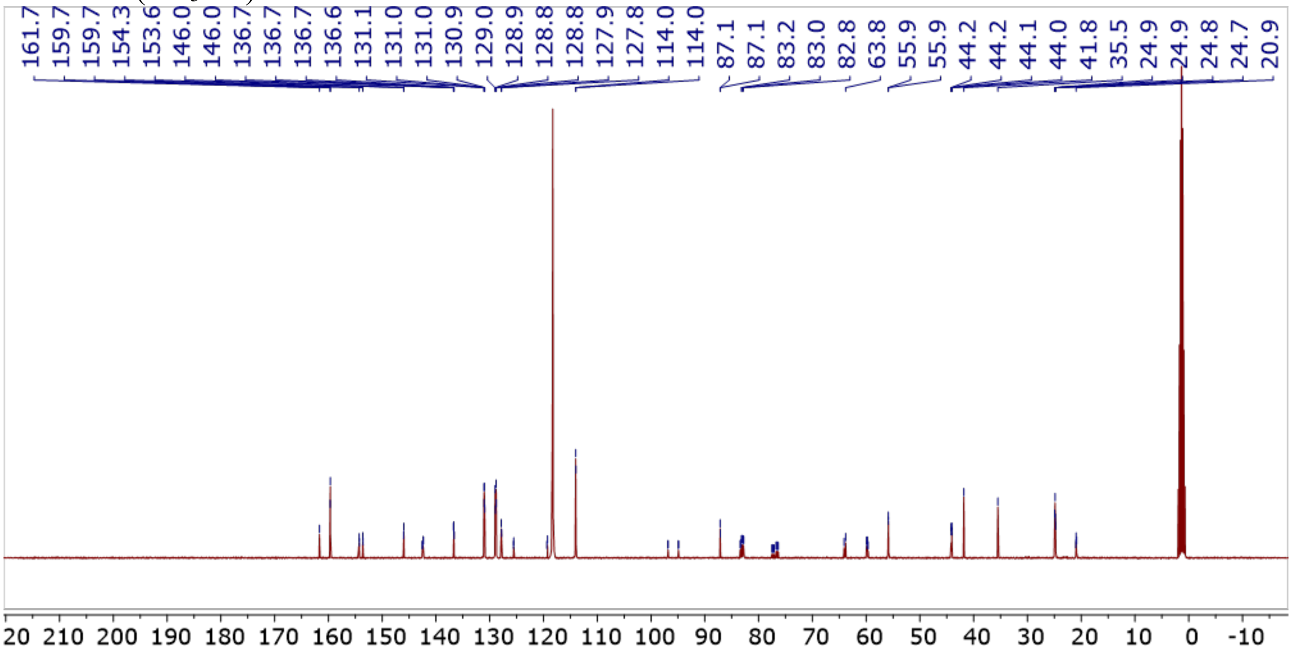

^{19}F NMR (CD_3CN) of **4-P**

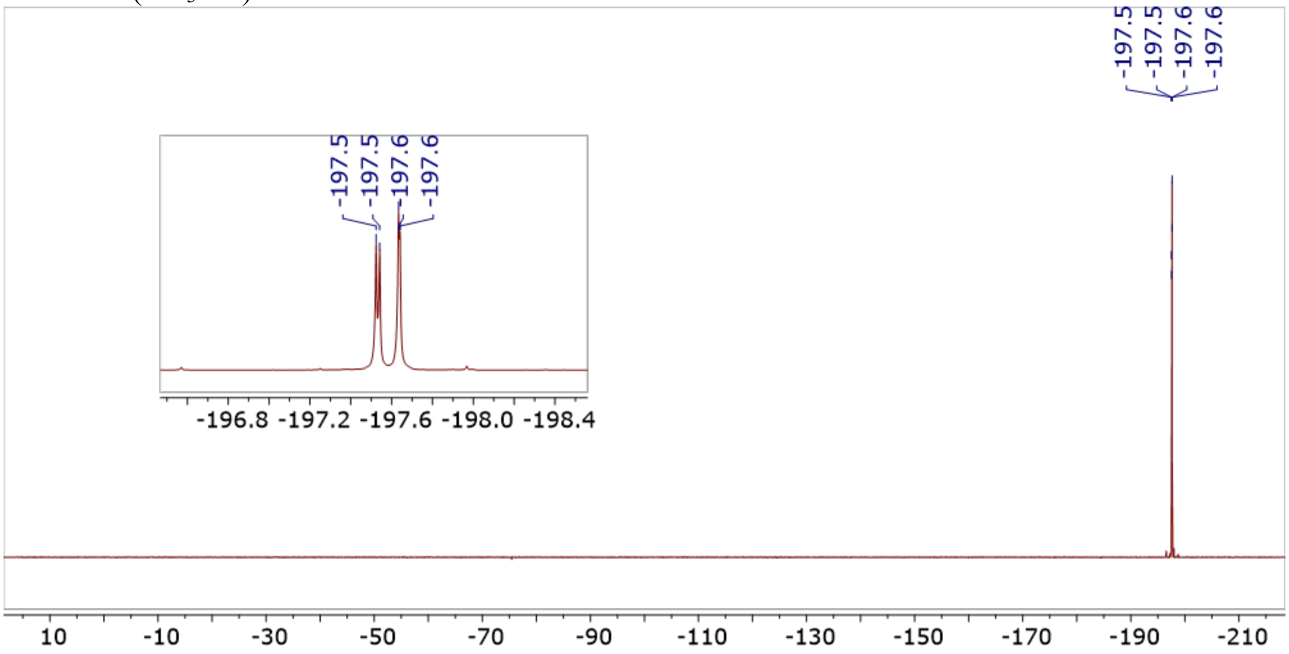

^{31}P NMR (CD_3CN) of **4-P**

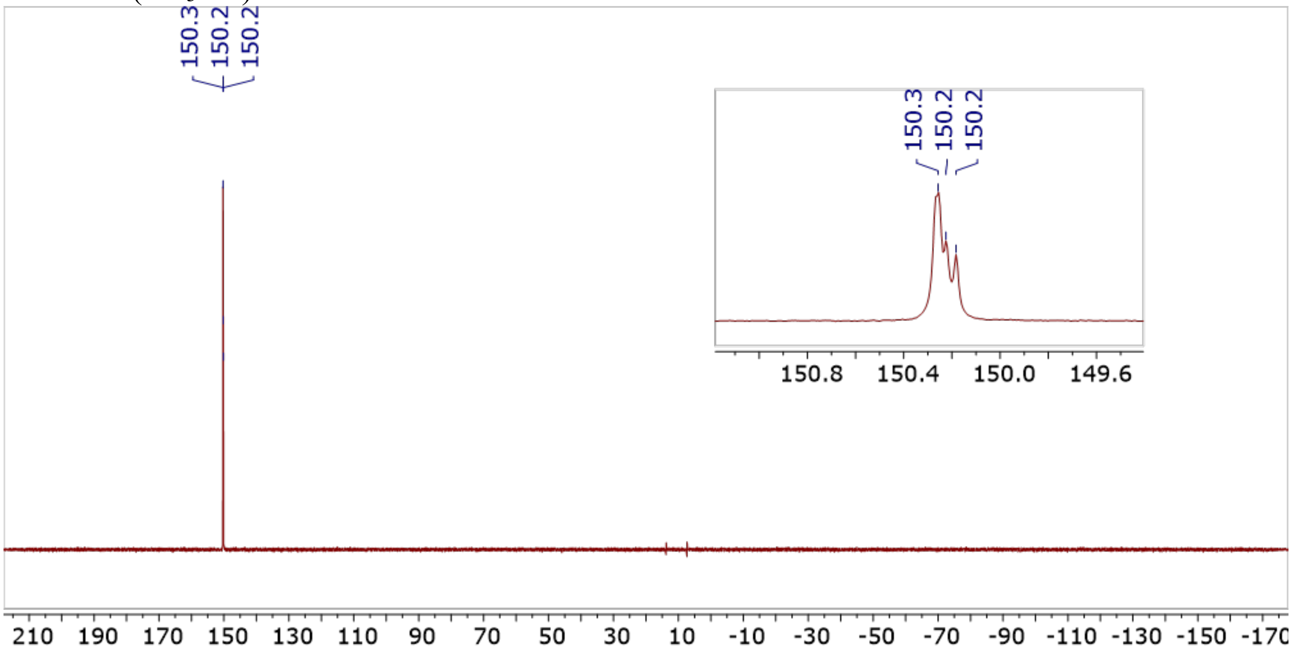

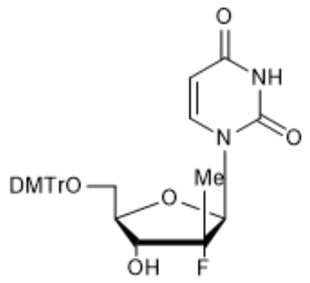

^{1}H NMR (CD_3CN) of **5**

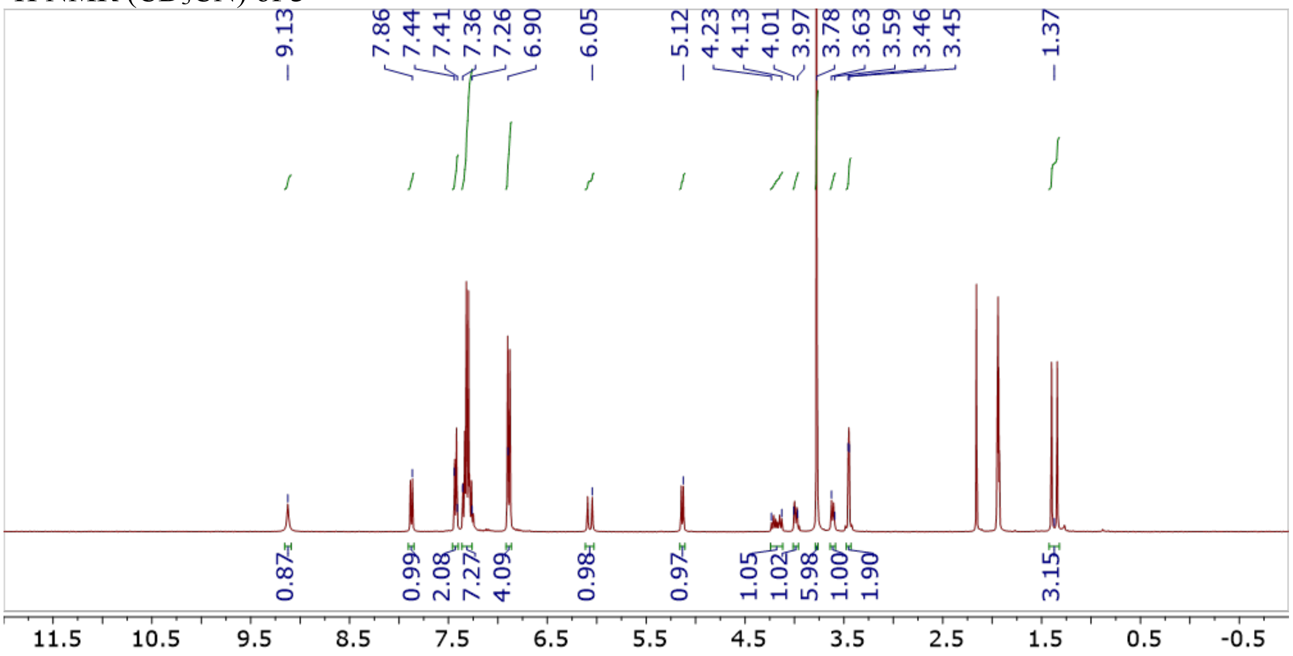

^{13}C NMR (CD_3CN) of **5**

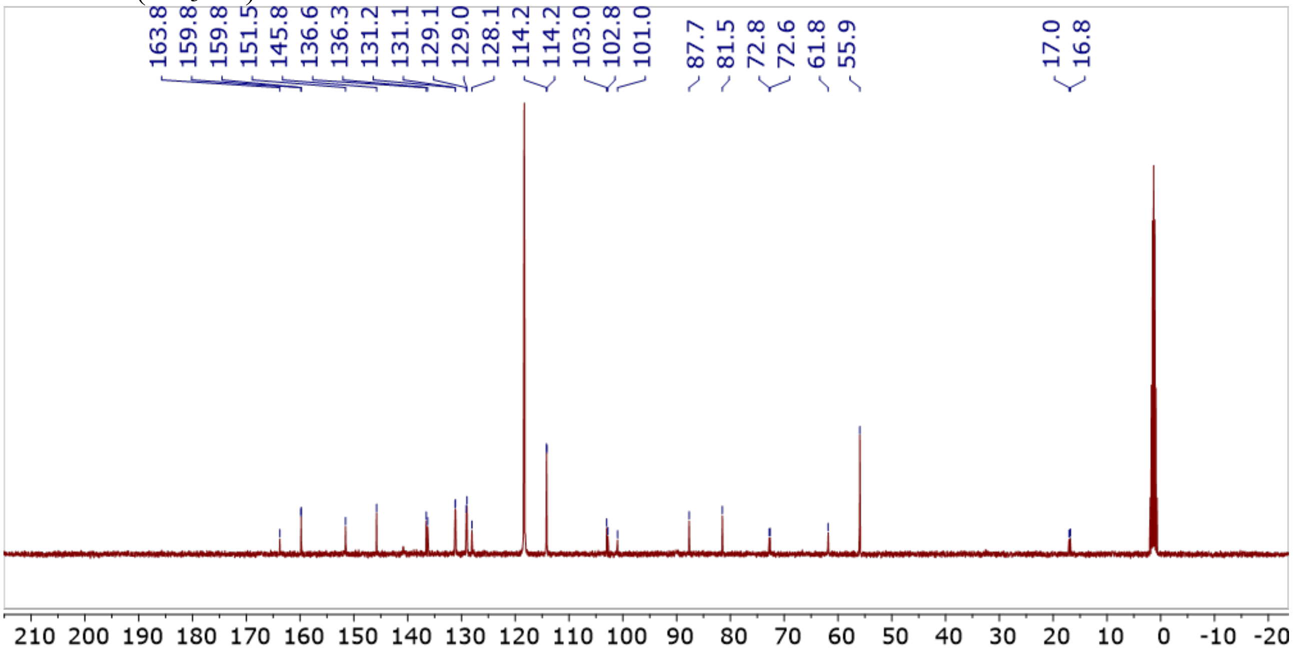

^{19}F NMR (CD_3CN) of **5**

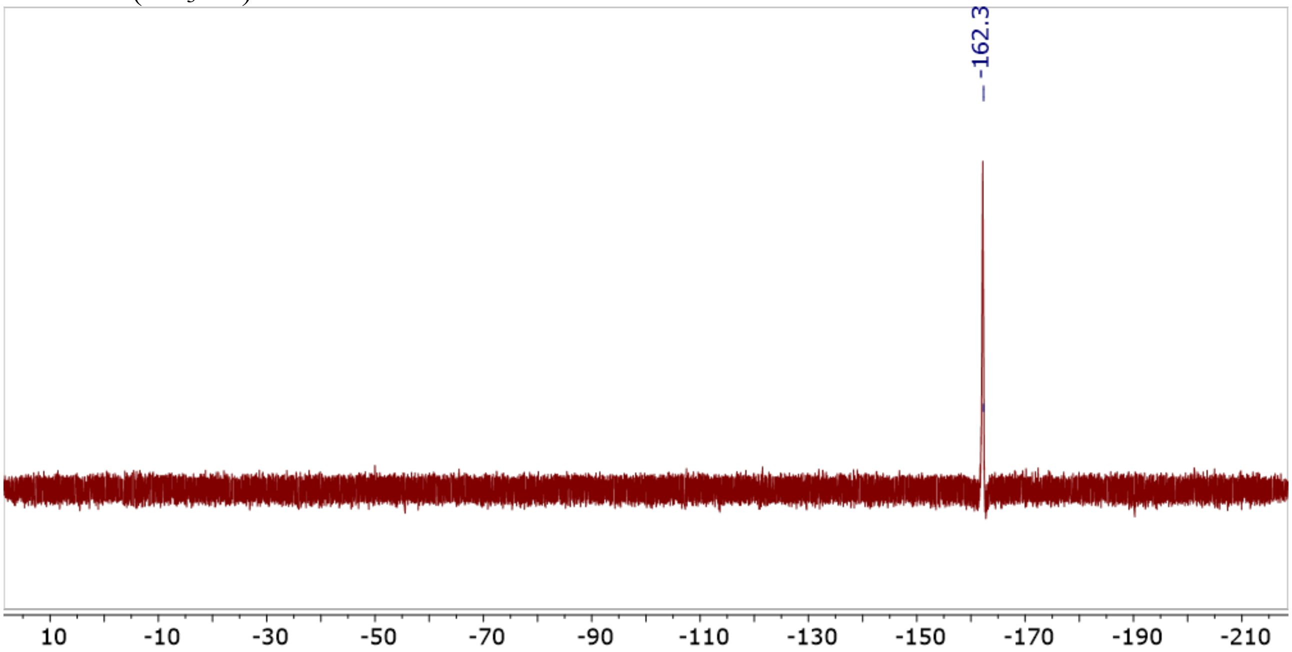

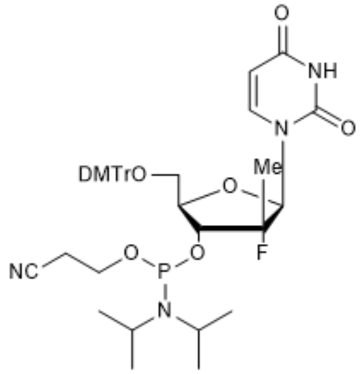

^{1}H NMR (CD_3CN) of **5-P**

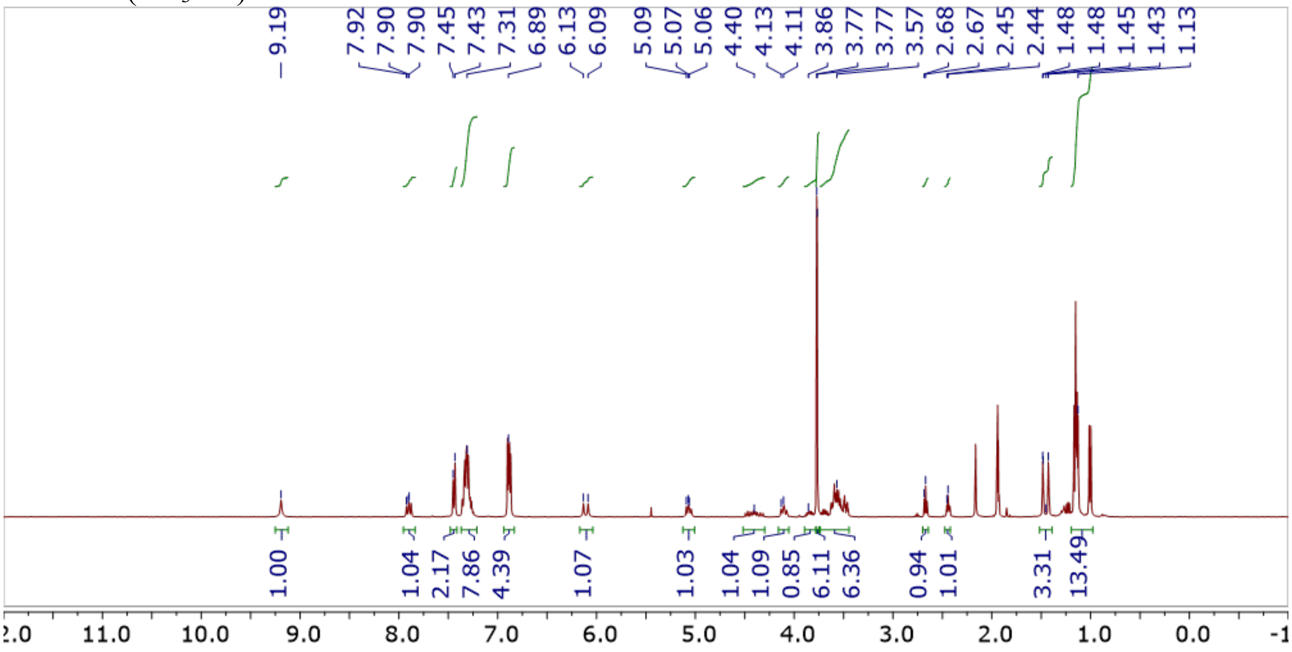

^{13}C NMR (CD_3CN) of **5-P**

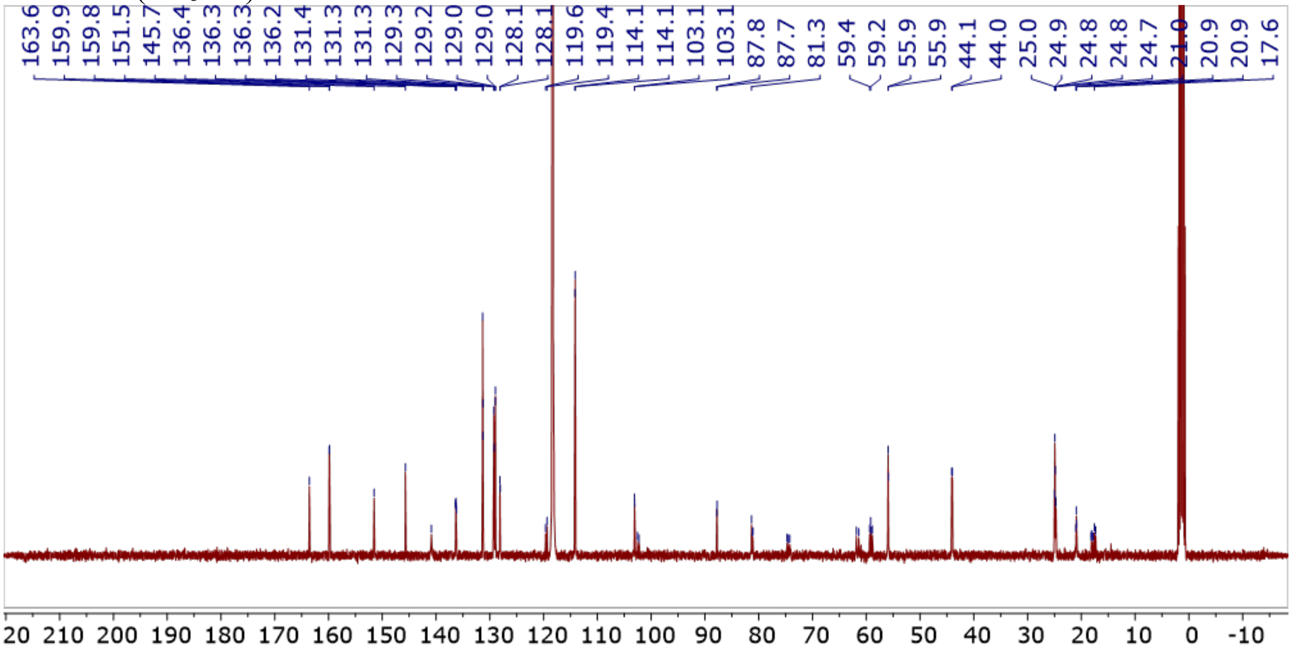

^{19}F NMR (CD_3CN) of **5-P**

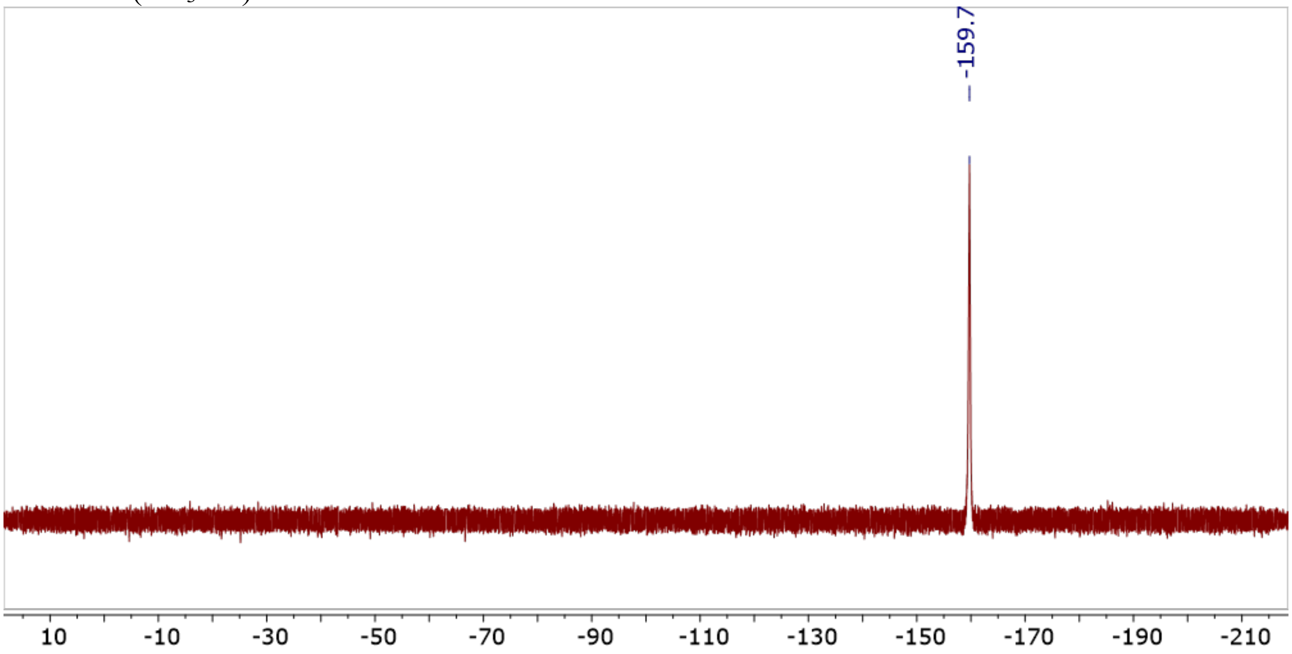

^{31}P NMR (CD_3CN) of **5-P**

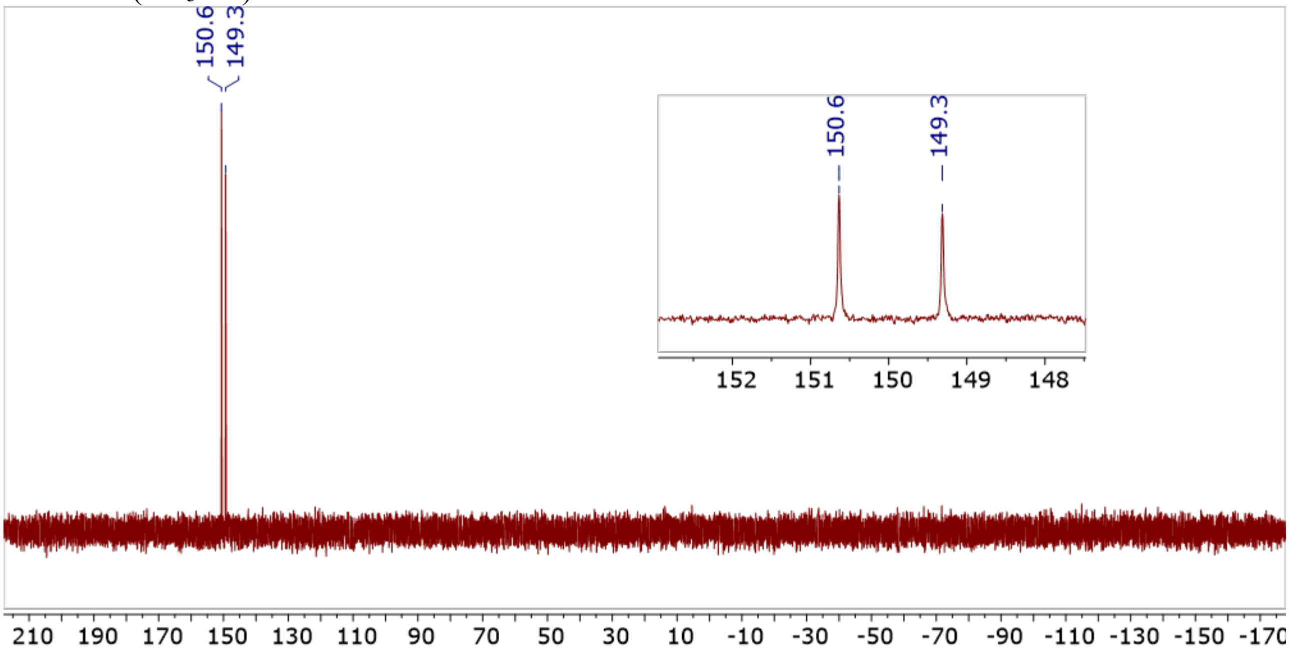

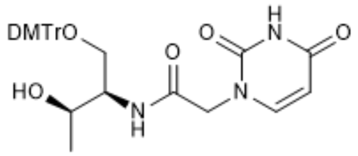

^{1}H NMR (*d6*-DMSO) of **6**

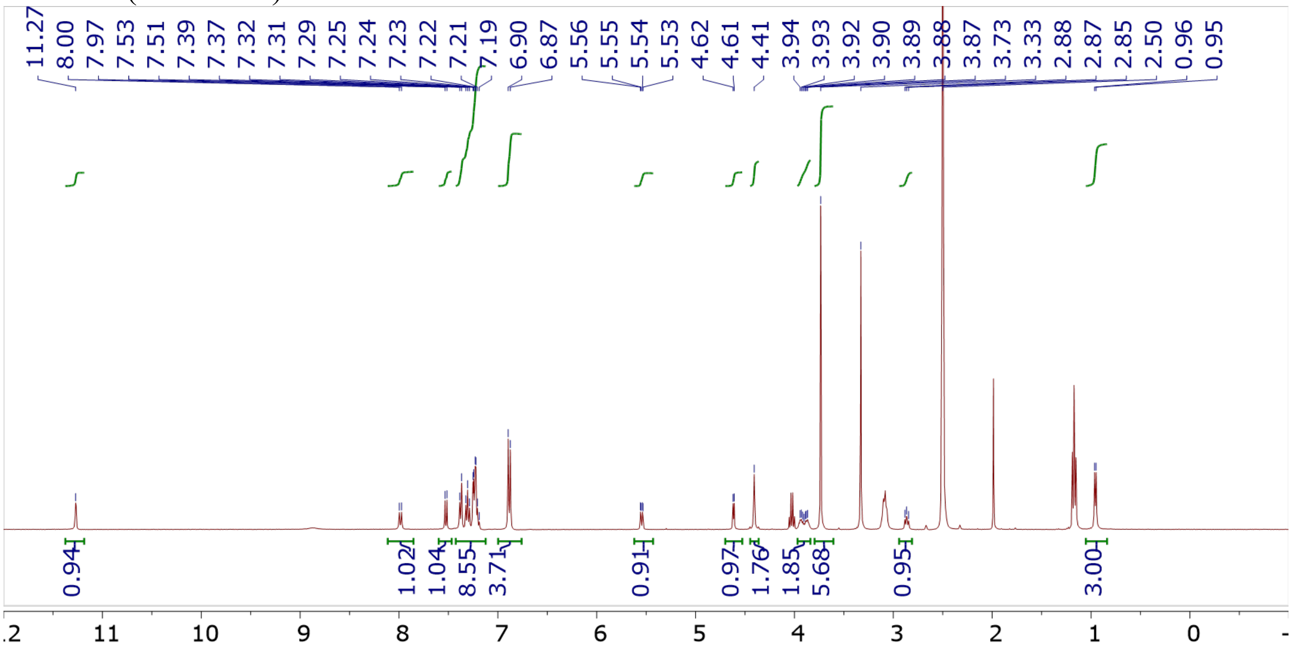

^{13}C NMR (*d6*-DMSO) of **6**

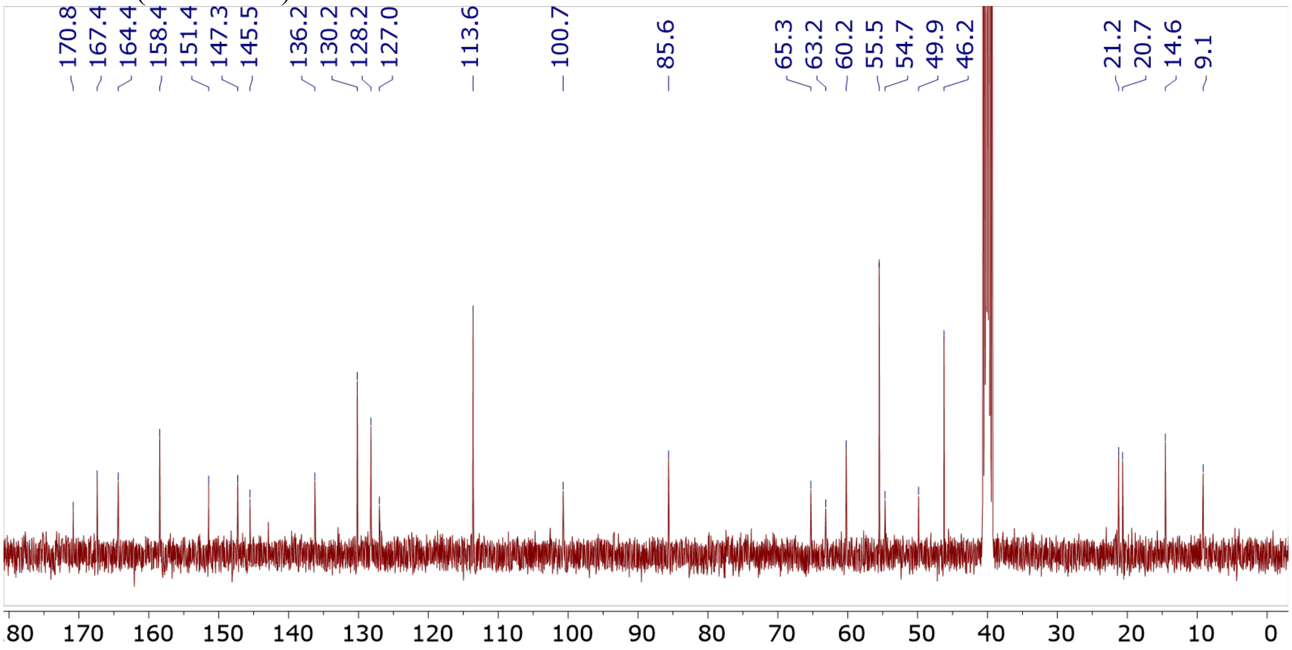

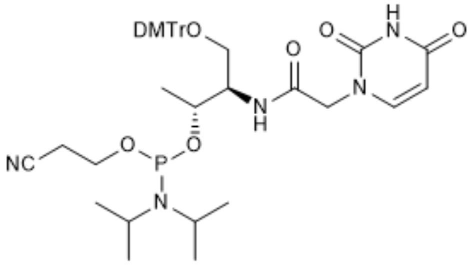

^{1}H NMR (CD_3CN) of **6-P**

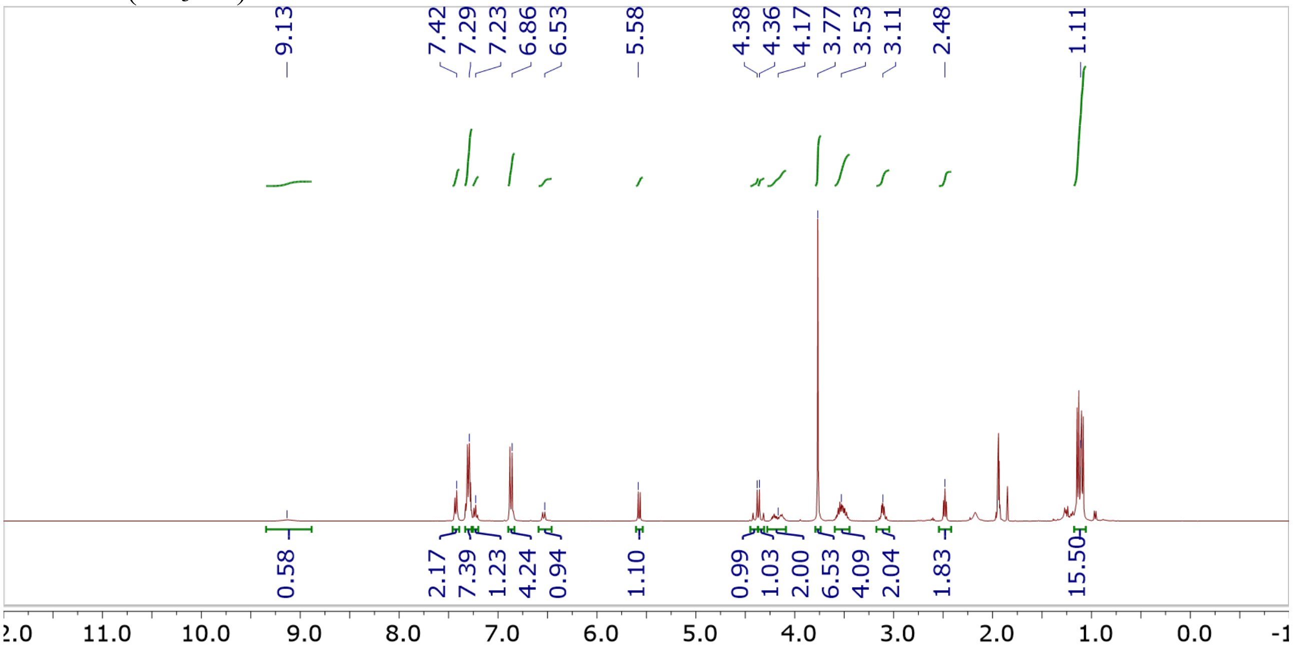

^{13}C NMR (CD_3CN) of **6-P**

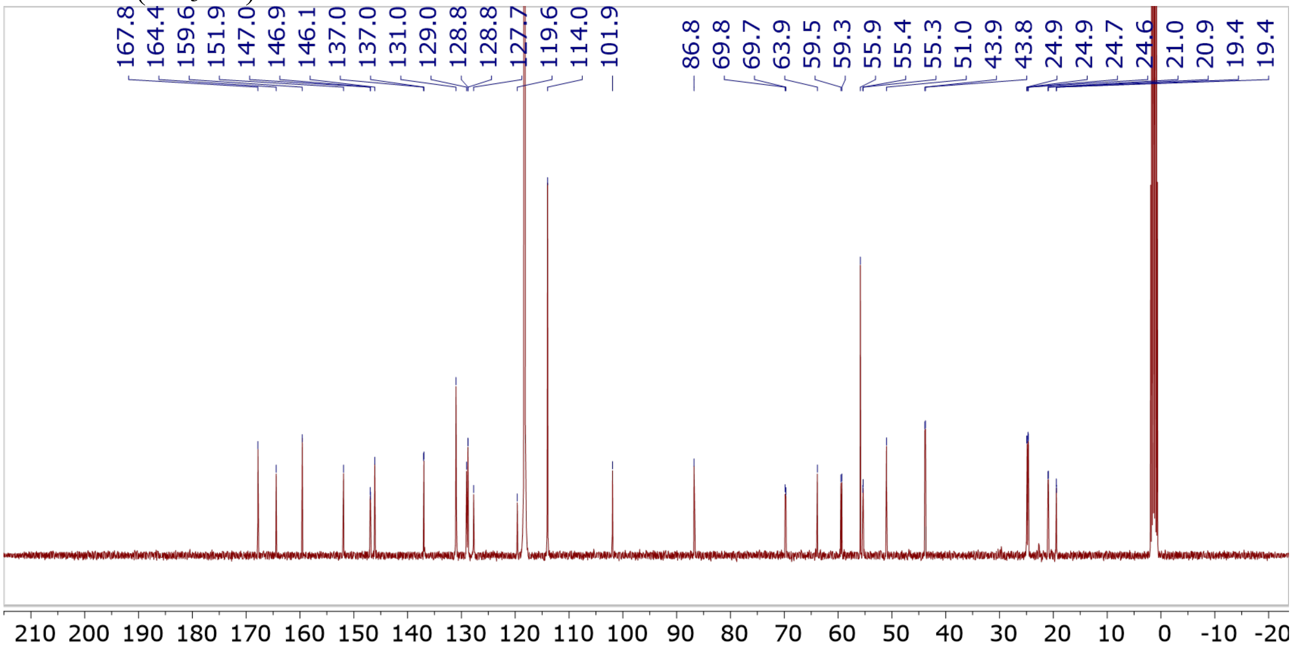

^{31}P NMR (CD_3CN) of **6-P**

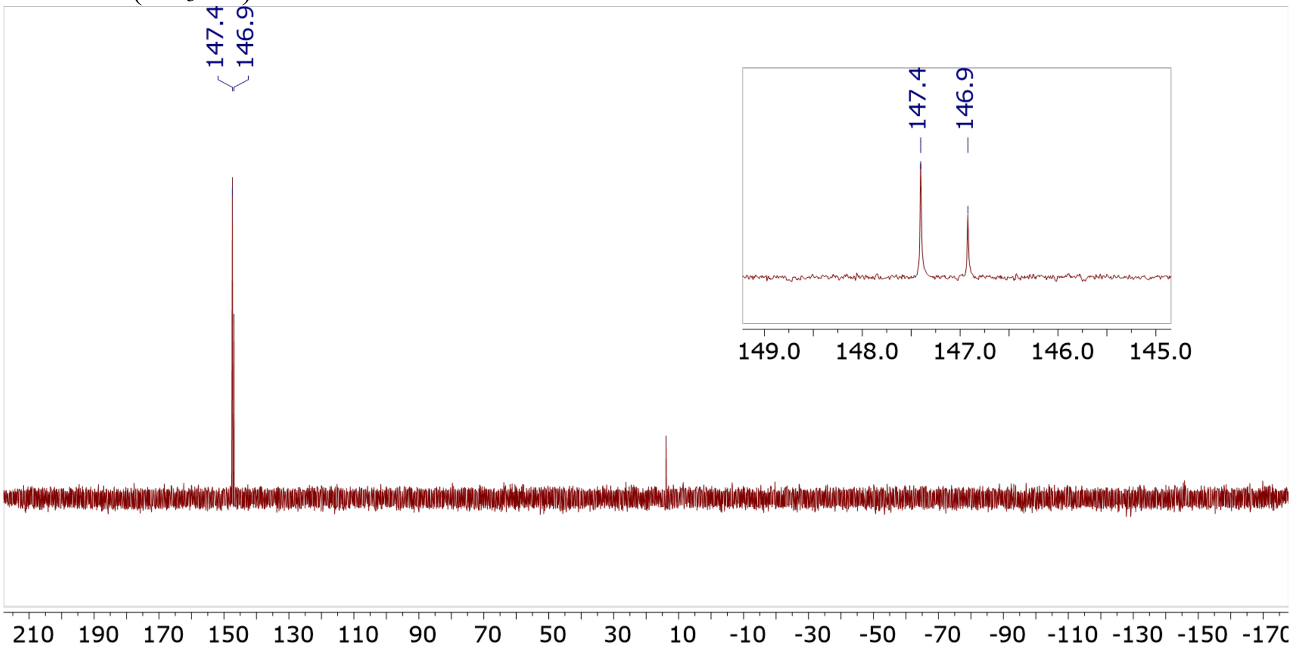

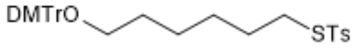

^{1}H NMR ($CDCl_3$) of **8.1**

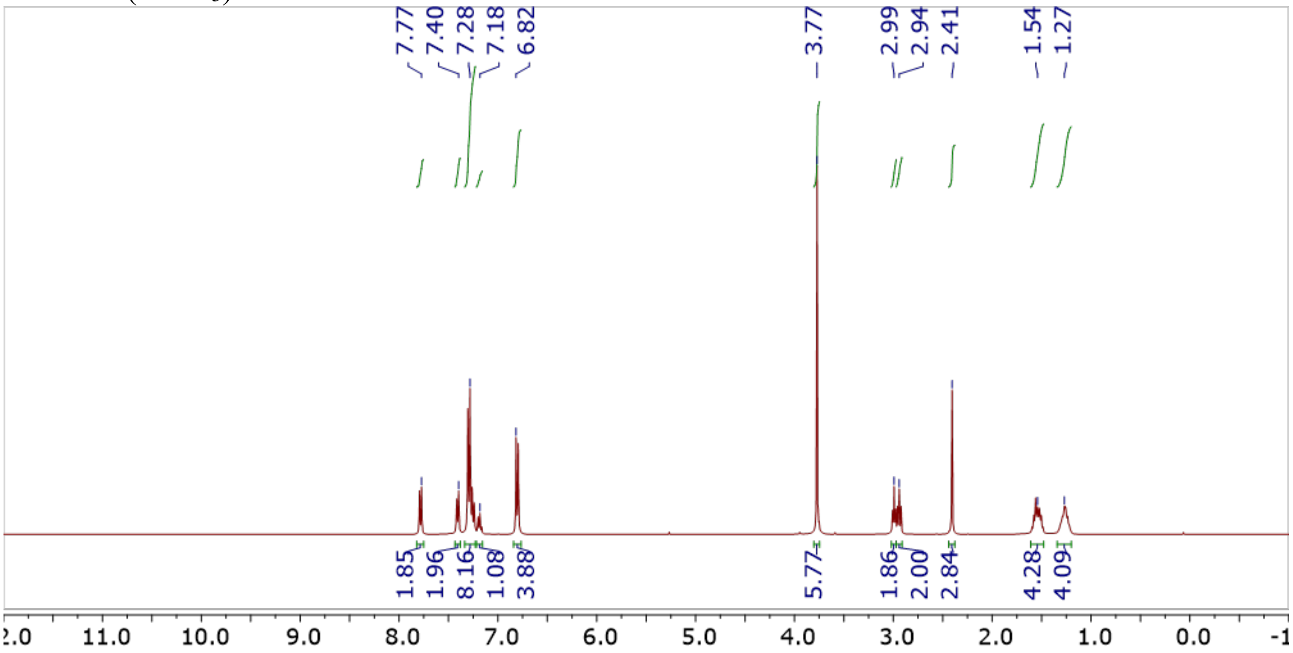

^{13}C NMR ($CDCl_3$) of **8.1**

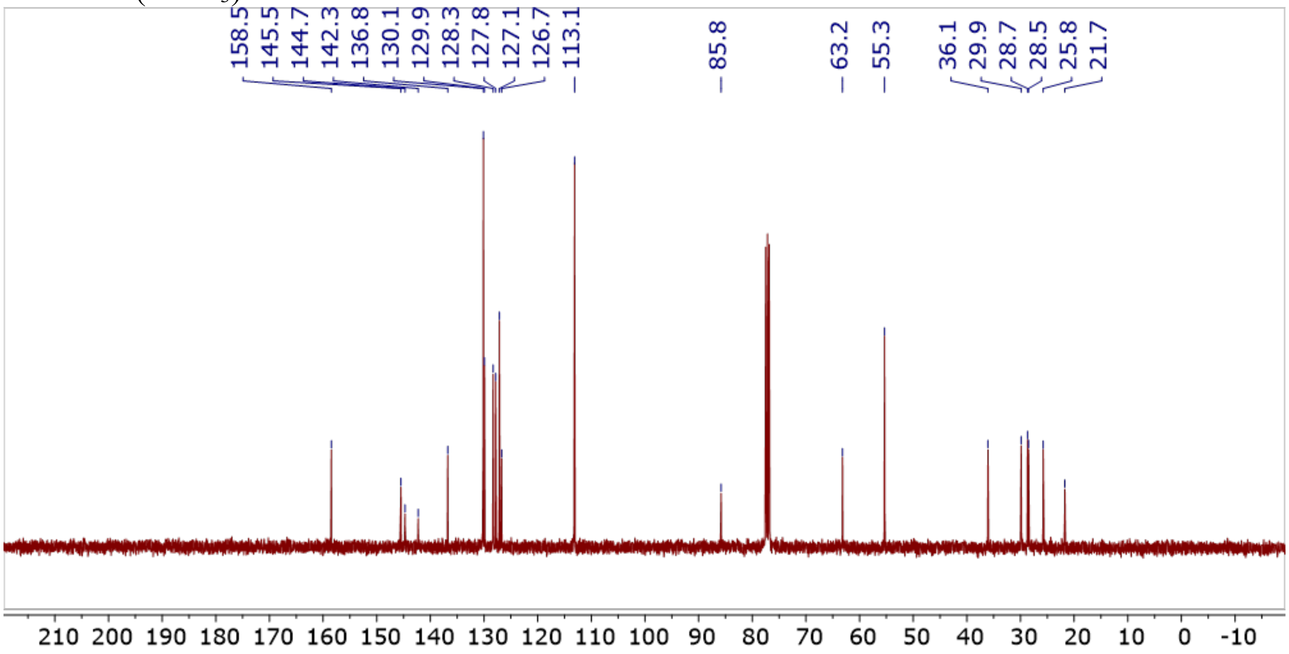

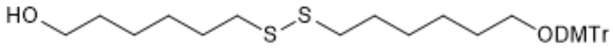

^{1}H NMR ($CDCl_3$) of **8**

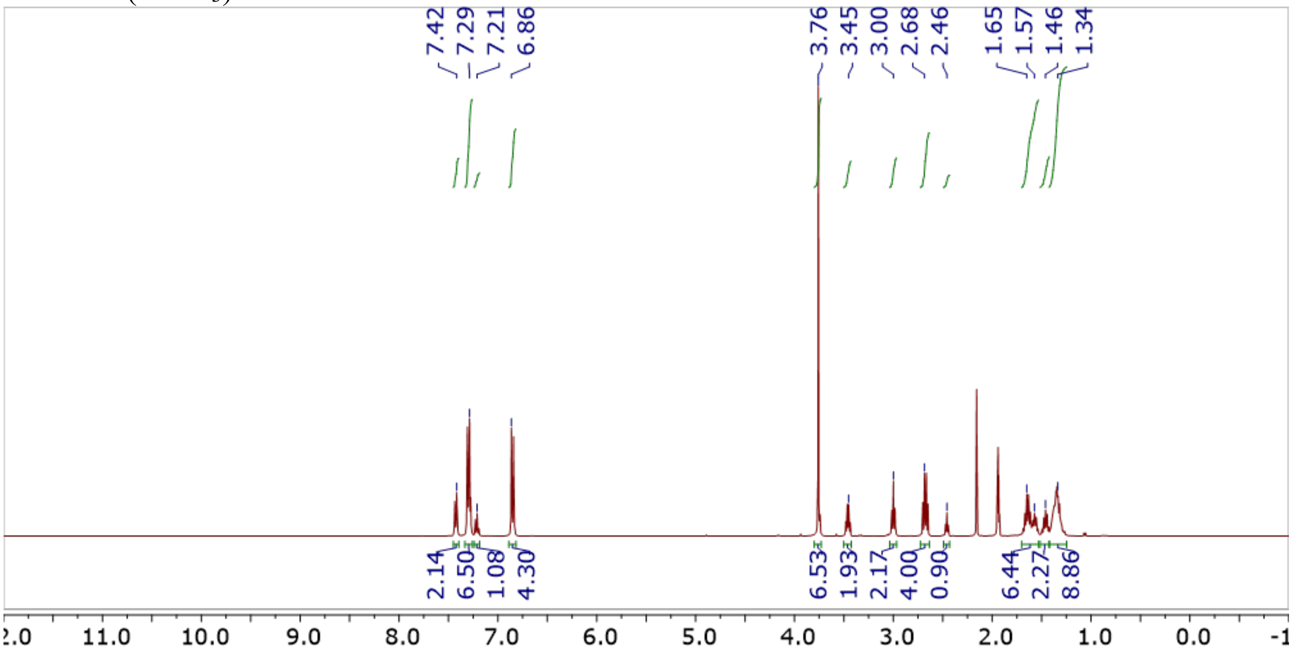

^{13}C NMR ($CDCl_3$) of **8**

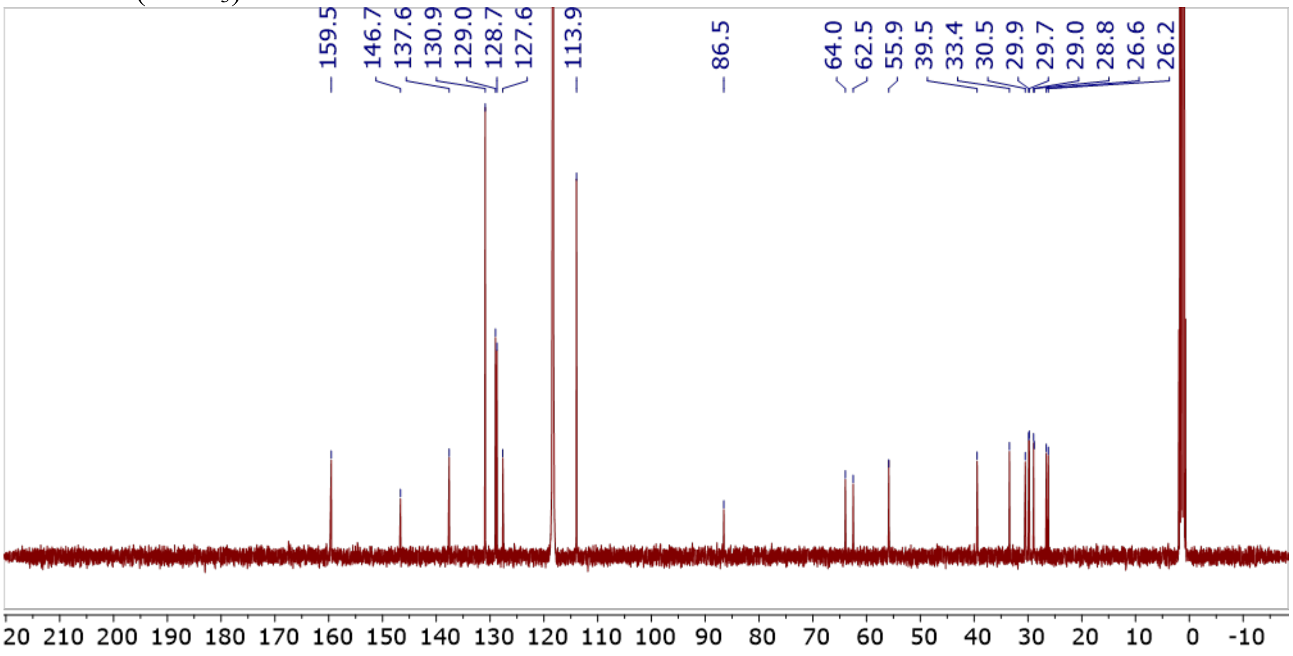

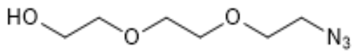

^{1}H NMR (CD_3CN) of **9**

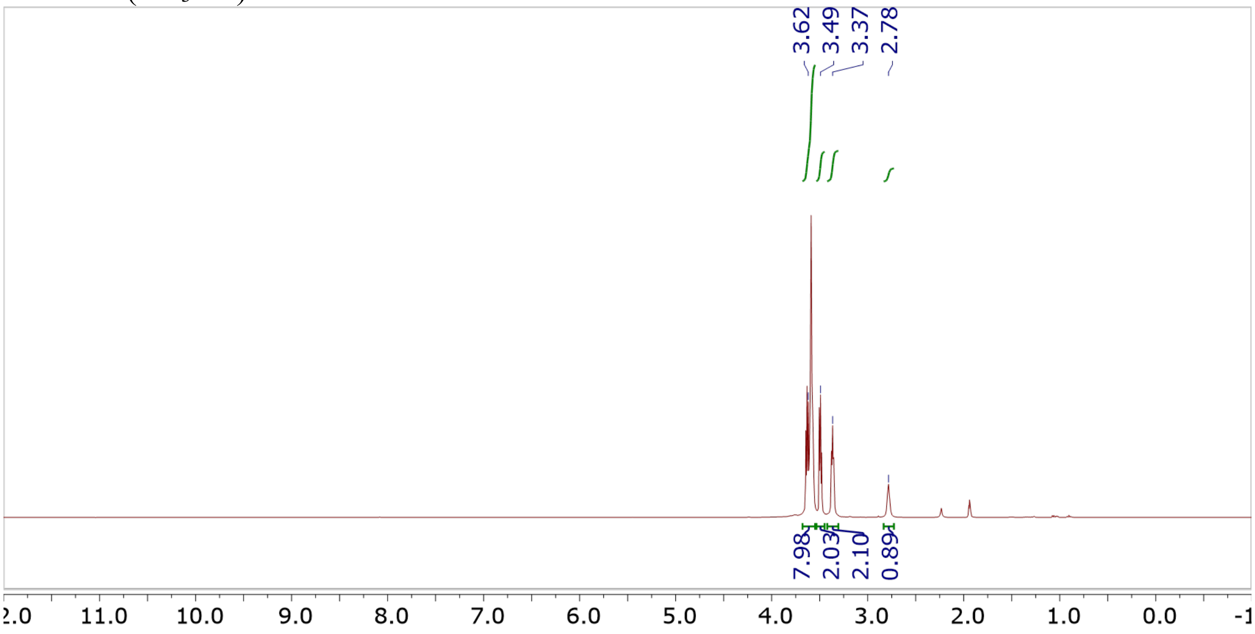

^{13}C NMR (CD_3CN) of **9**

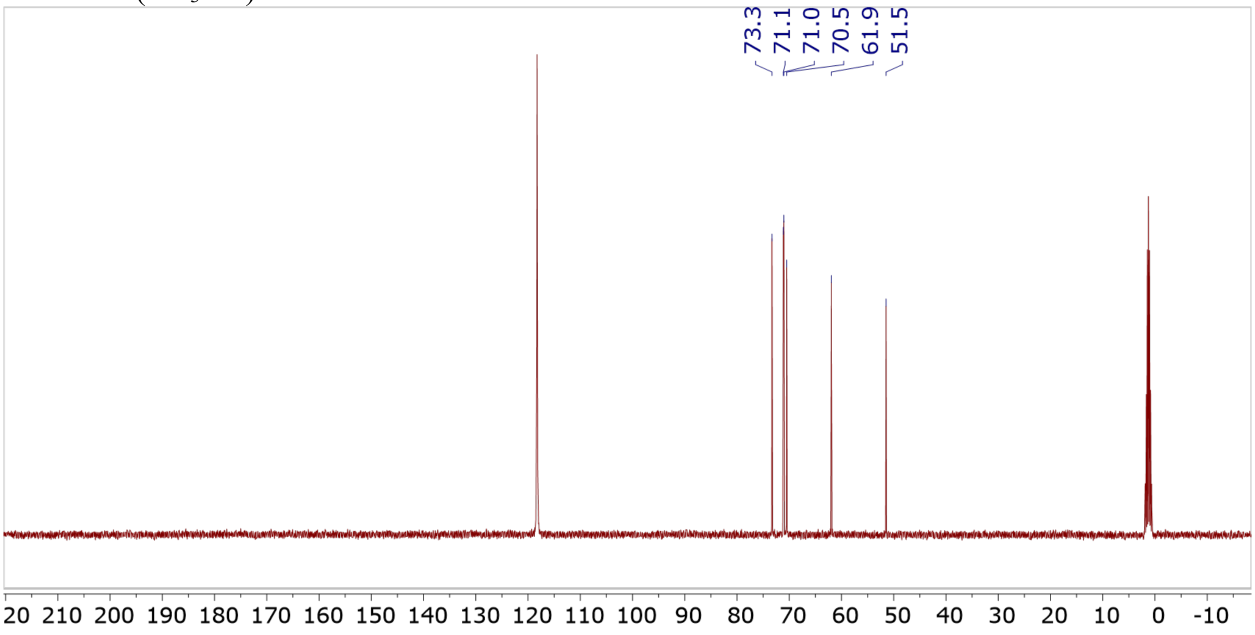

NMR Spectra of 9AJ and 2-cyanoethyl *N*,*N*-diisopropylphosphonamidate

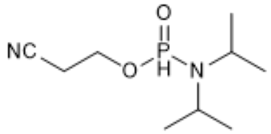

^{1}H NMR (CD_3CN) of 2-cyanoethyl *N*,*N*-diisopropylphosphonamidate

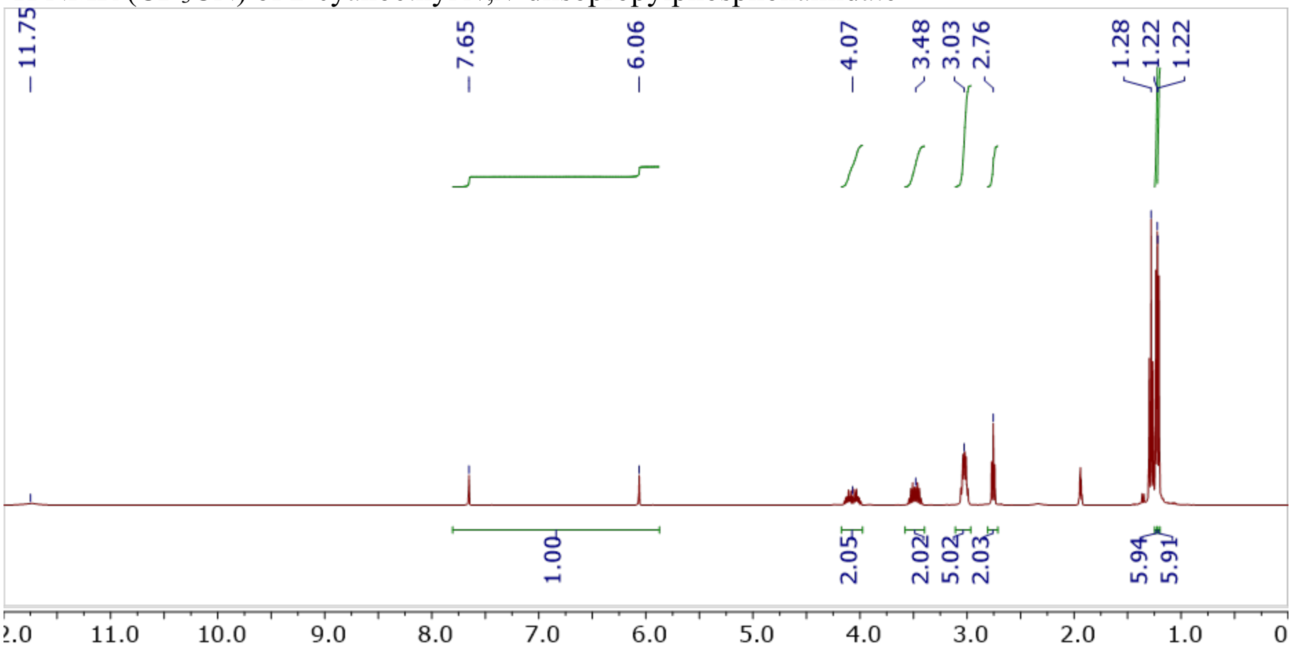

^{31}P NMR (CD_3CN) of 2-cyanoethyl *N*,*N*-diisopropylphosphonamidate

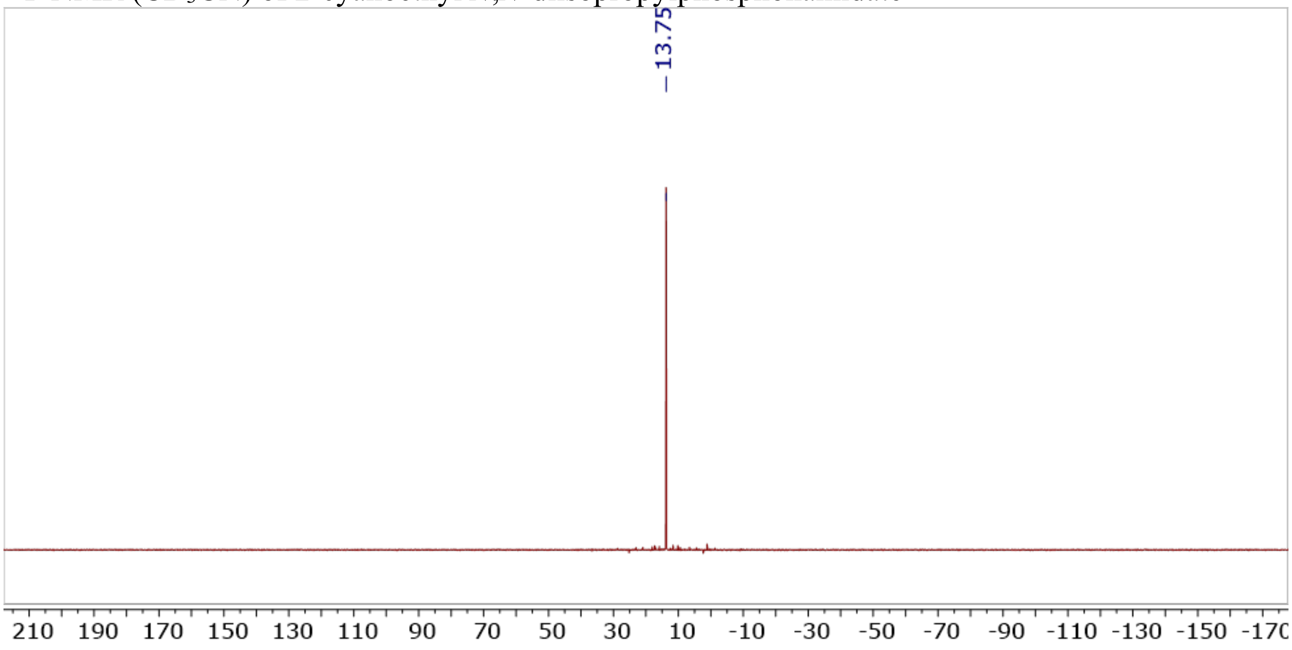

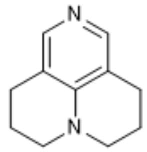

^{1}H NMR (CD_3CN) of 9AJ

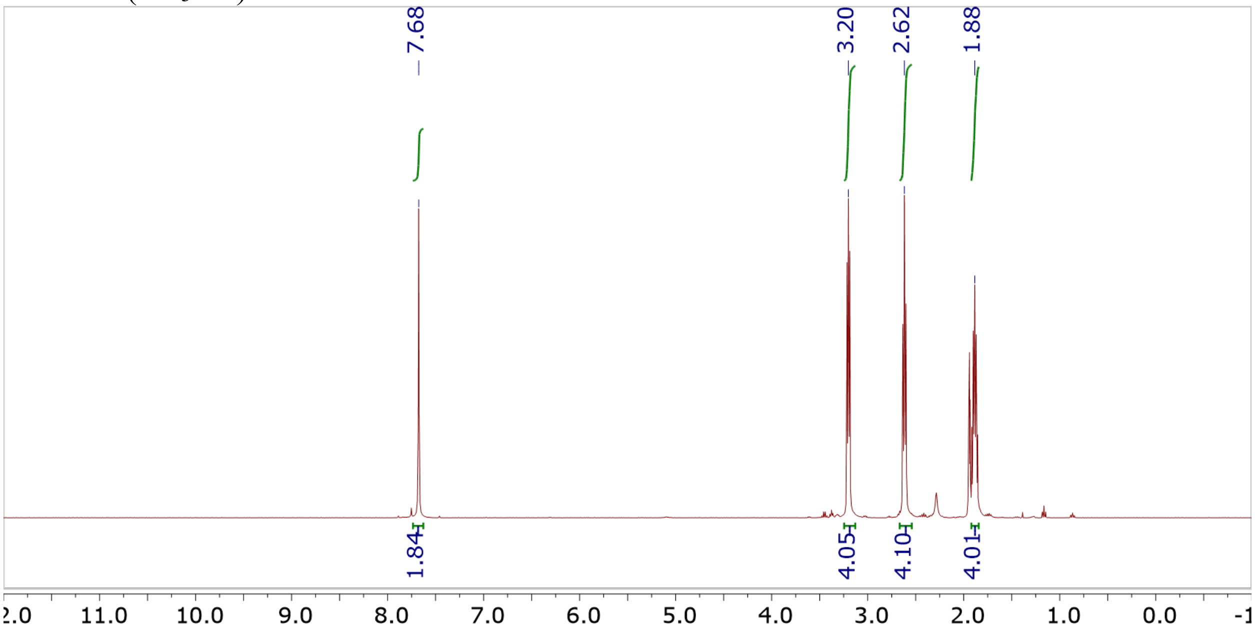

NMR Spectra (Full) of Flowthrough (Stacked with References)

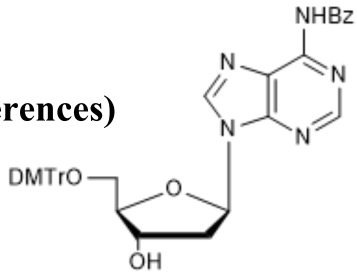

Benzoyl 2'deoxyadenosine ^{1}H NMR (CD_3CN)

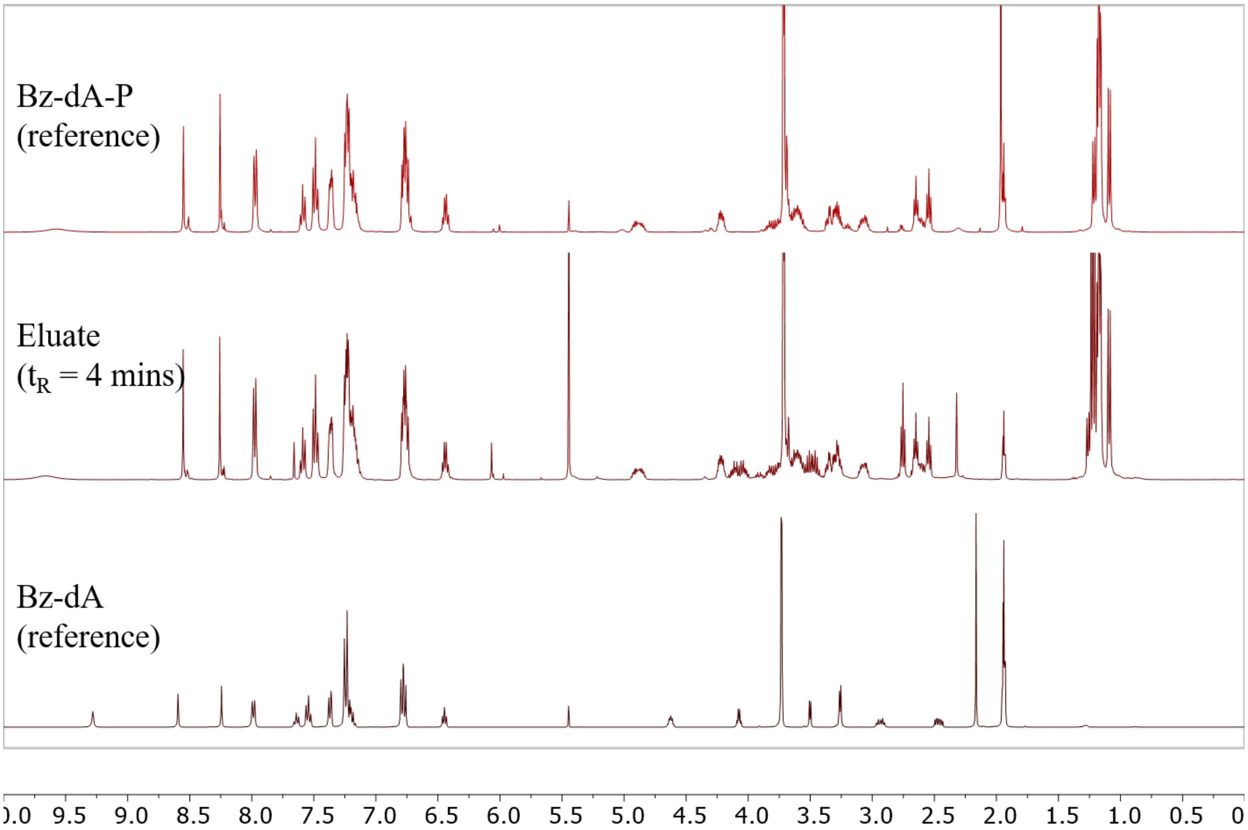

Benzoyl 2'deoxyadenosine ^{31}P NMR (CD_3CN), eluate

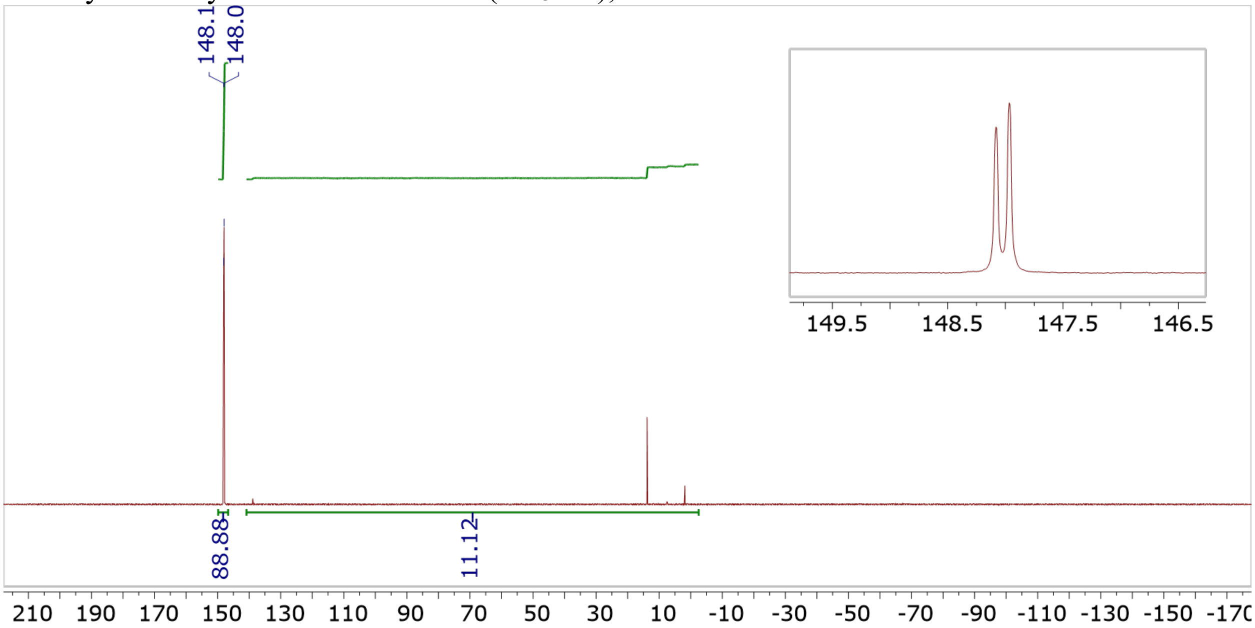

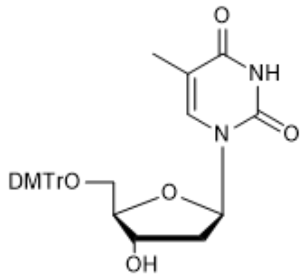

Thymidine ^{1}H NMR (CD_3CN)

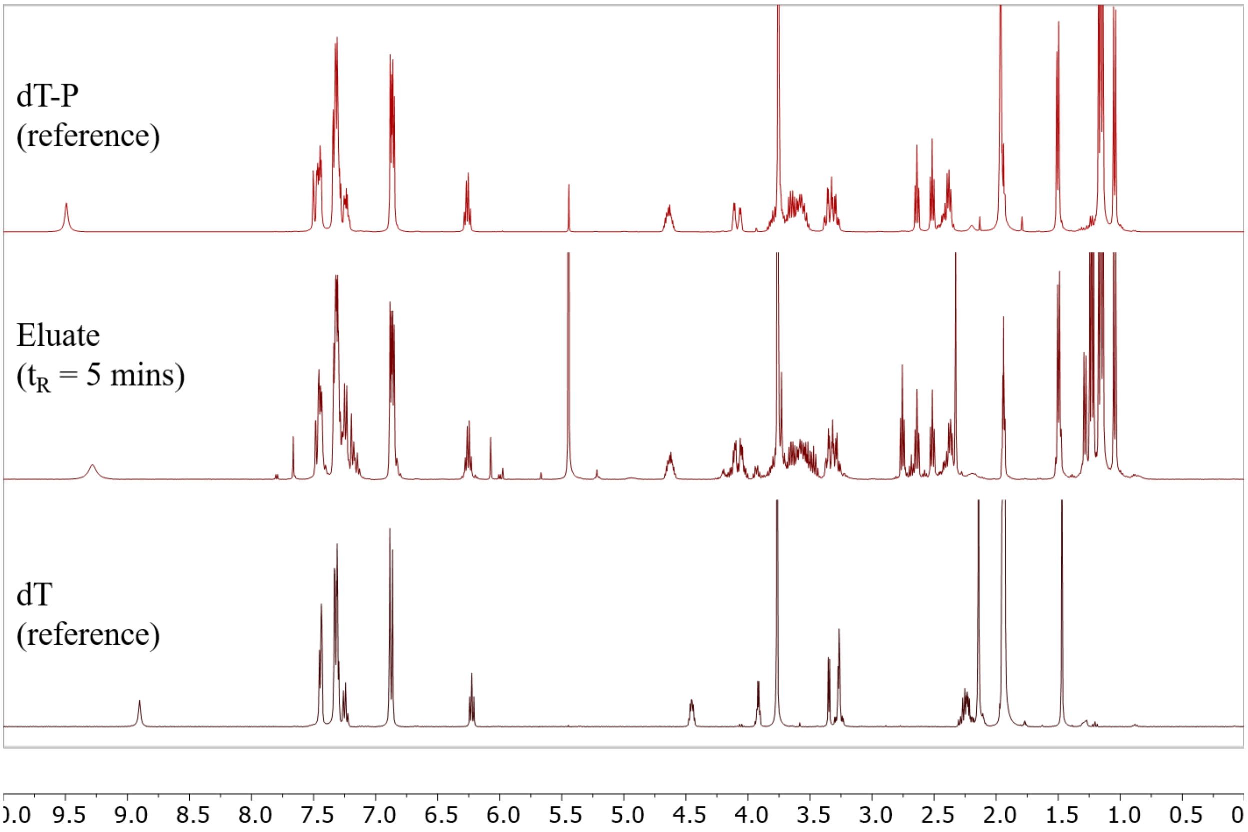

Thymidine ^{31}P NMR (CD_3CN), eluate

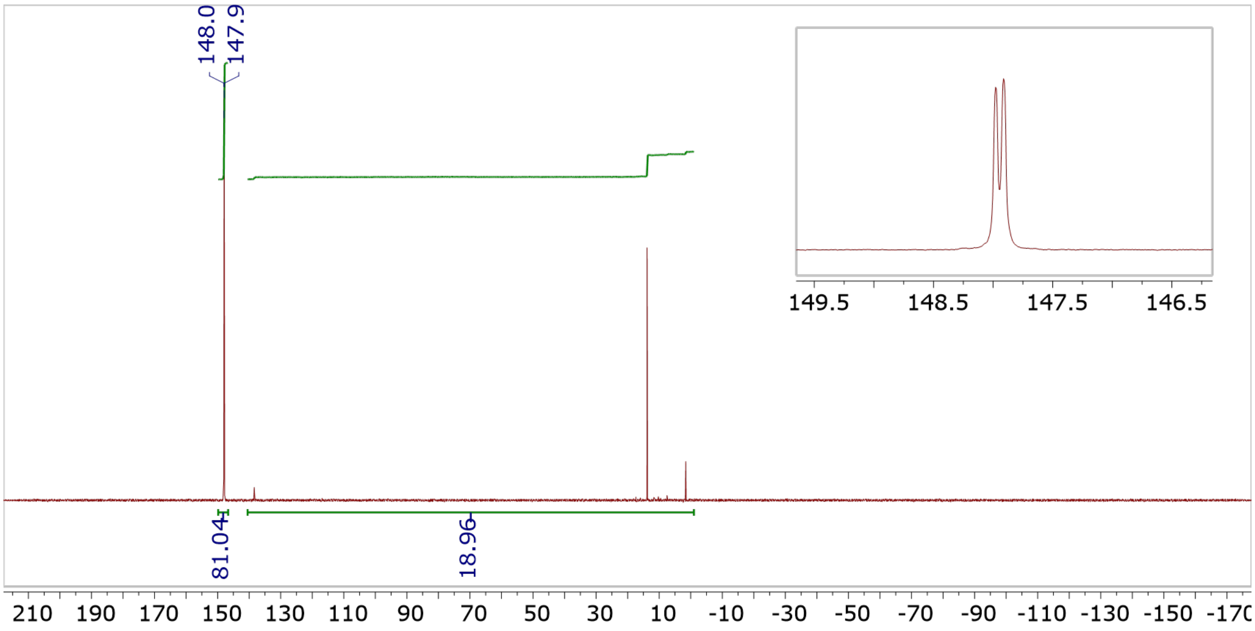

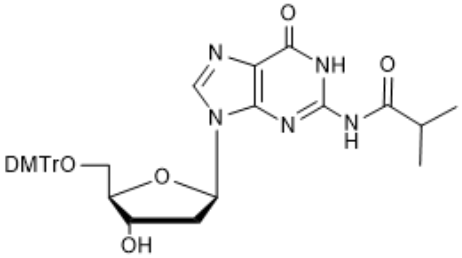

Isobutyryl 2’deoxyguanosine ^{1}H NMR (CD_3CN)

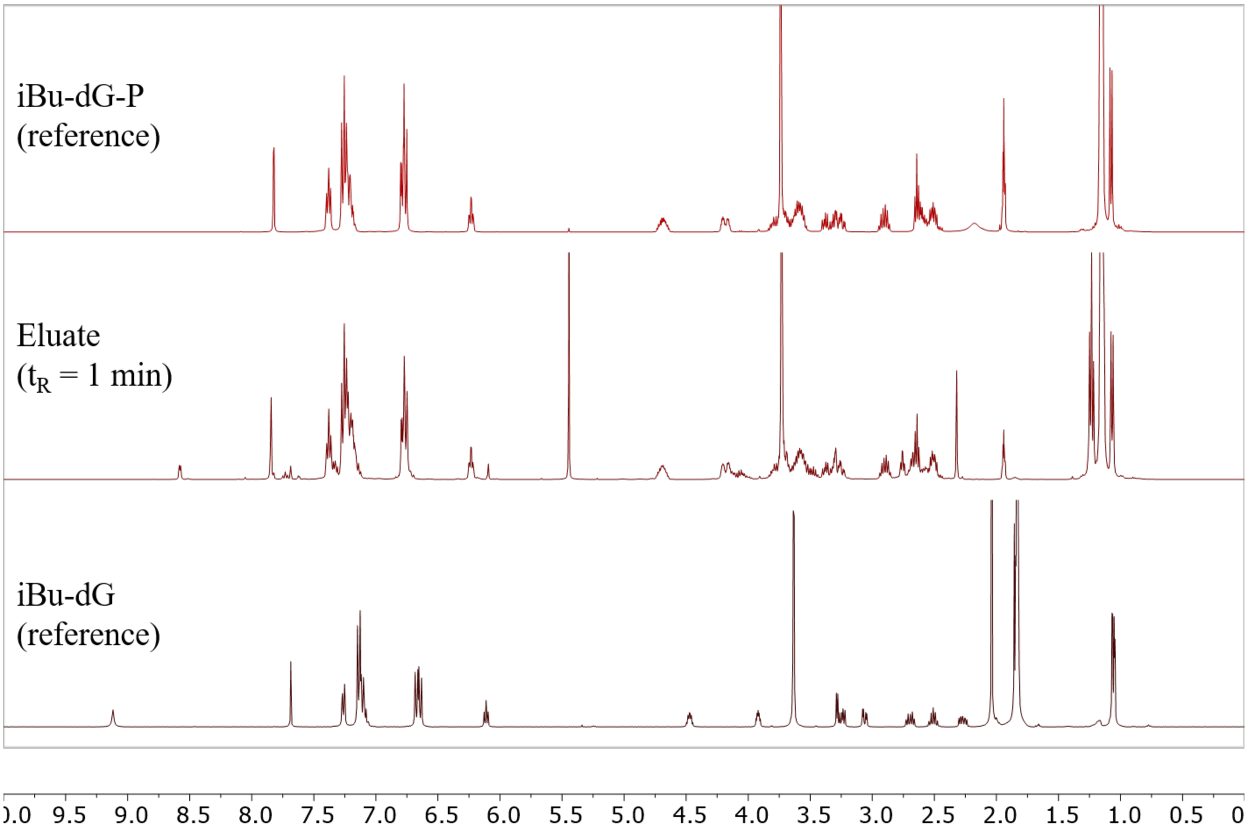

Isobutyryl 2’deoxyguanosine ^{31}P NMR (CD_3CN), eluate

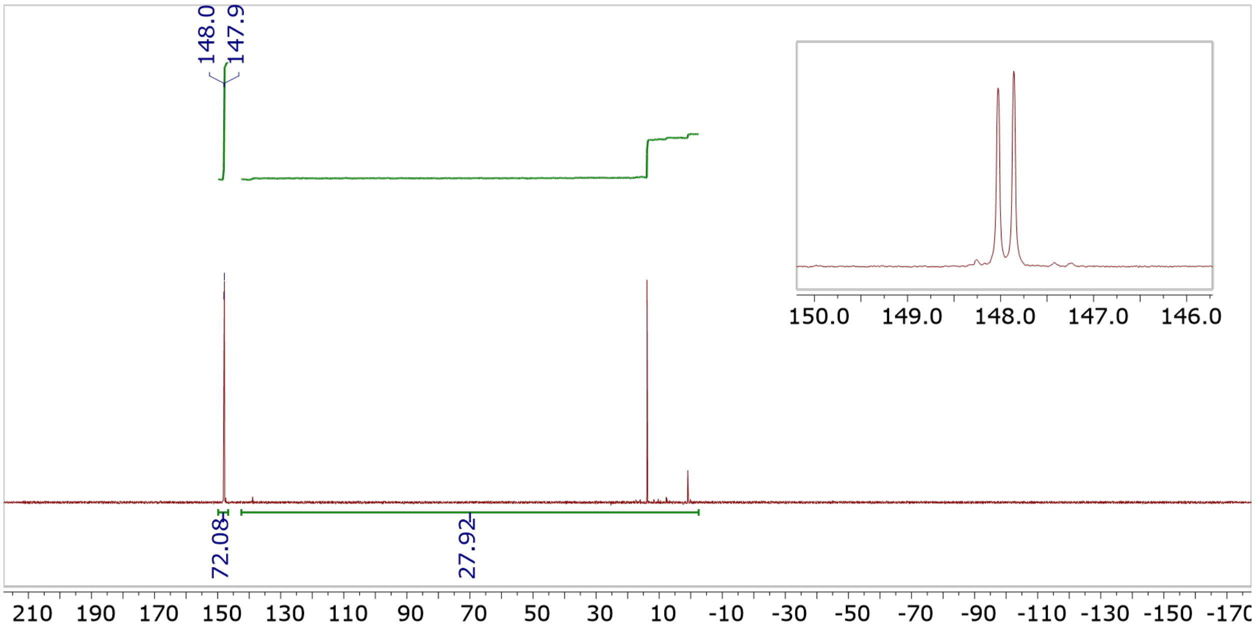

dmf 2’deoxyguanosine ^{1}H NMR (CD_3CN)

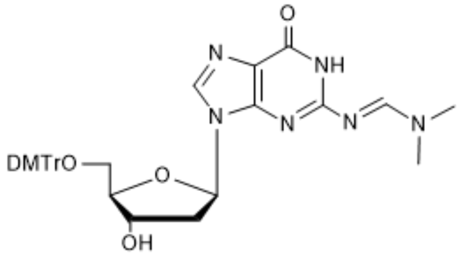

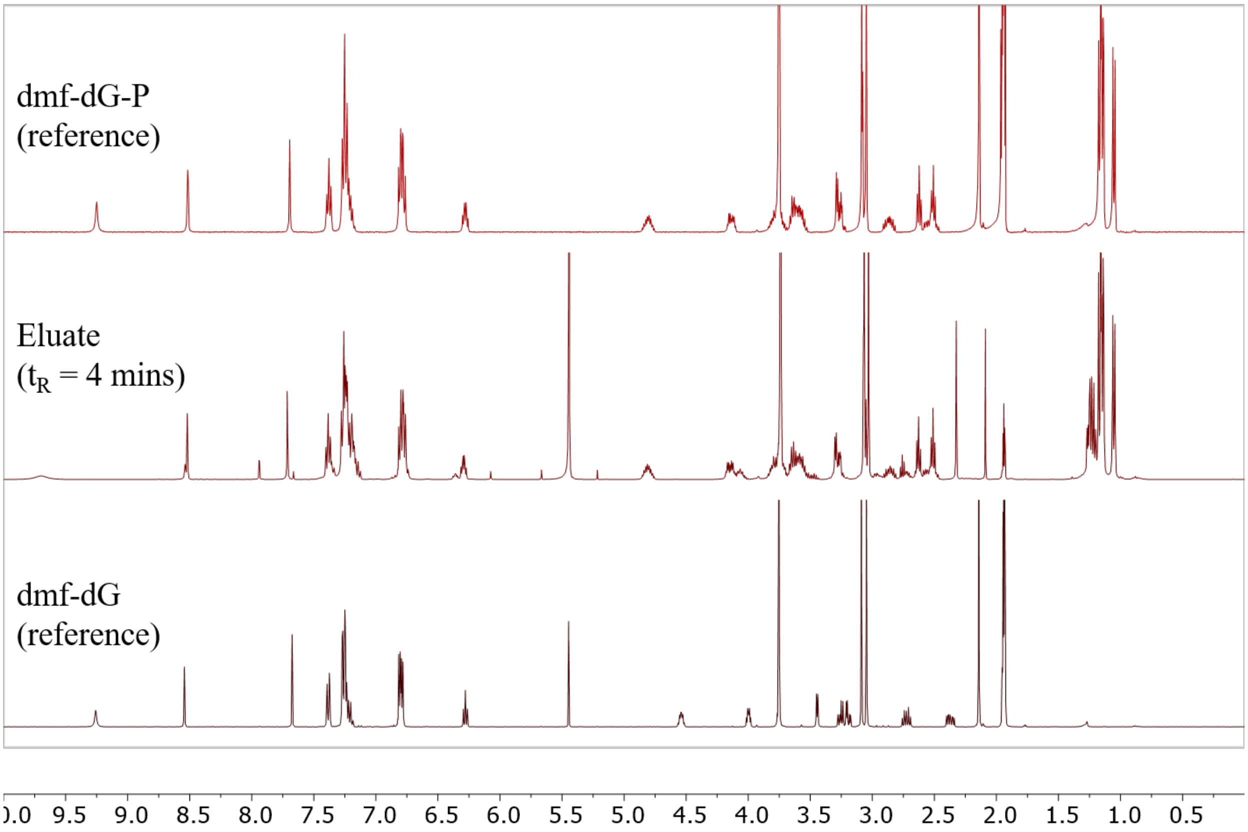

dmf 2’deoxyguanosine ^{31}P NMR (CD_3CN)

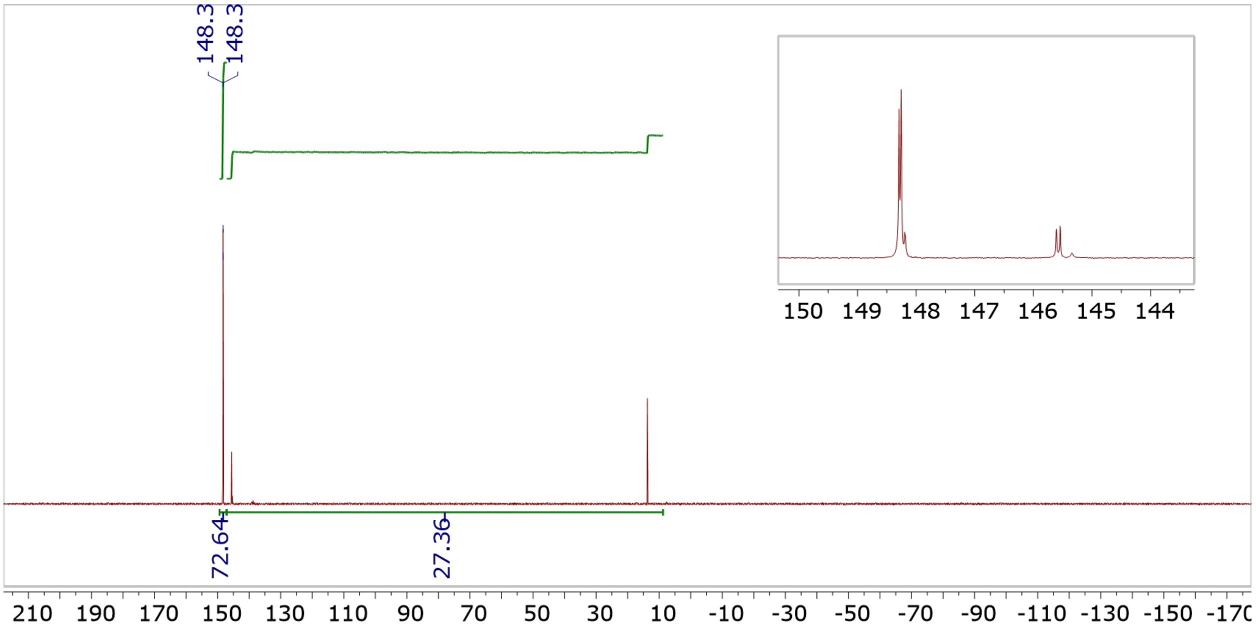

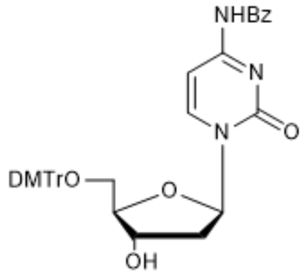

Benzoyl 2’deoxycytidine ¹H NMR (CD_3CN)

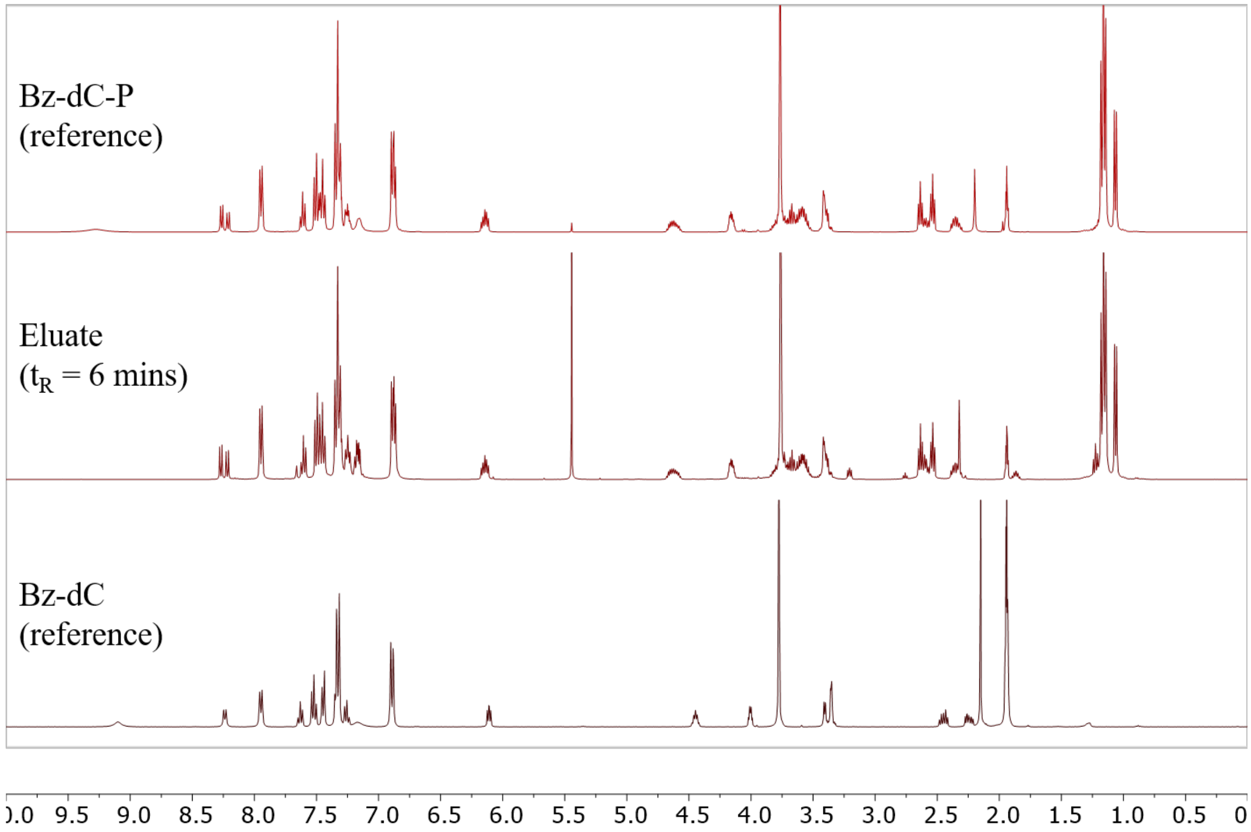

Benzoyl 2’deoxycytidine ³¹P NMR (CD_3CN), eluate

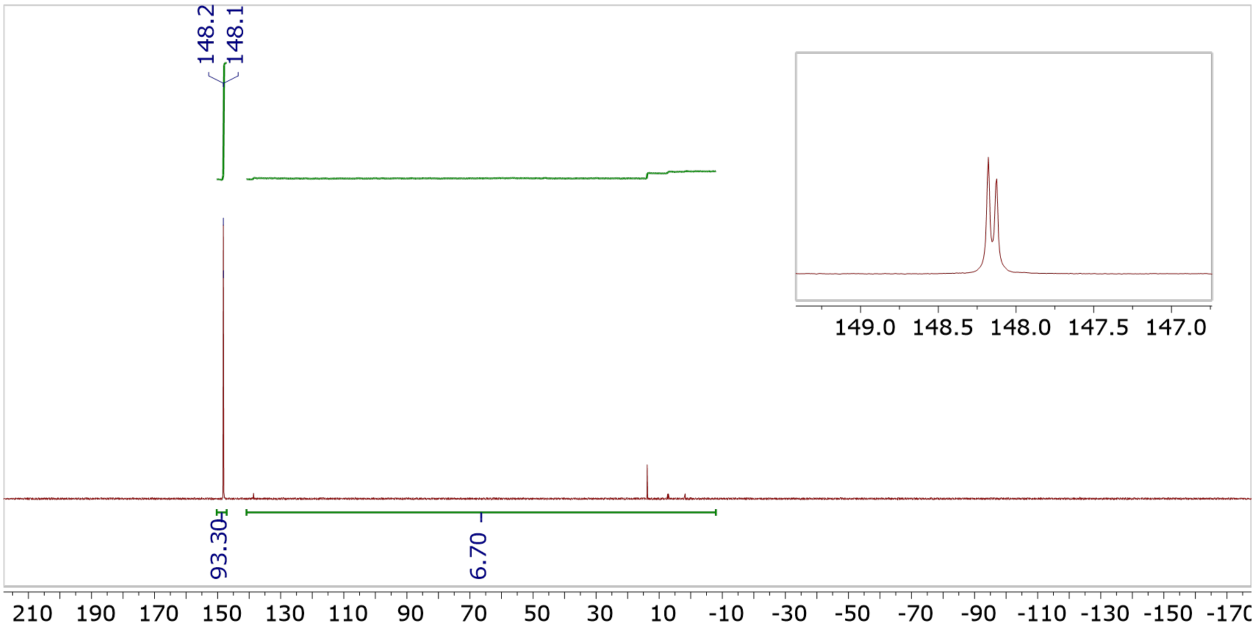

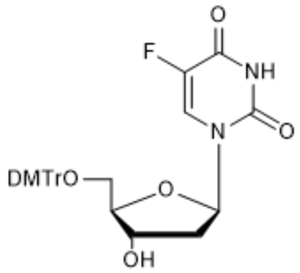

Compound **1**, ^{1}H NMR (CD_3CN)

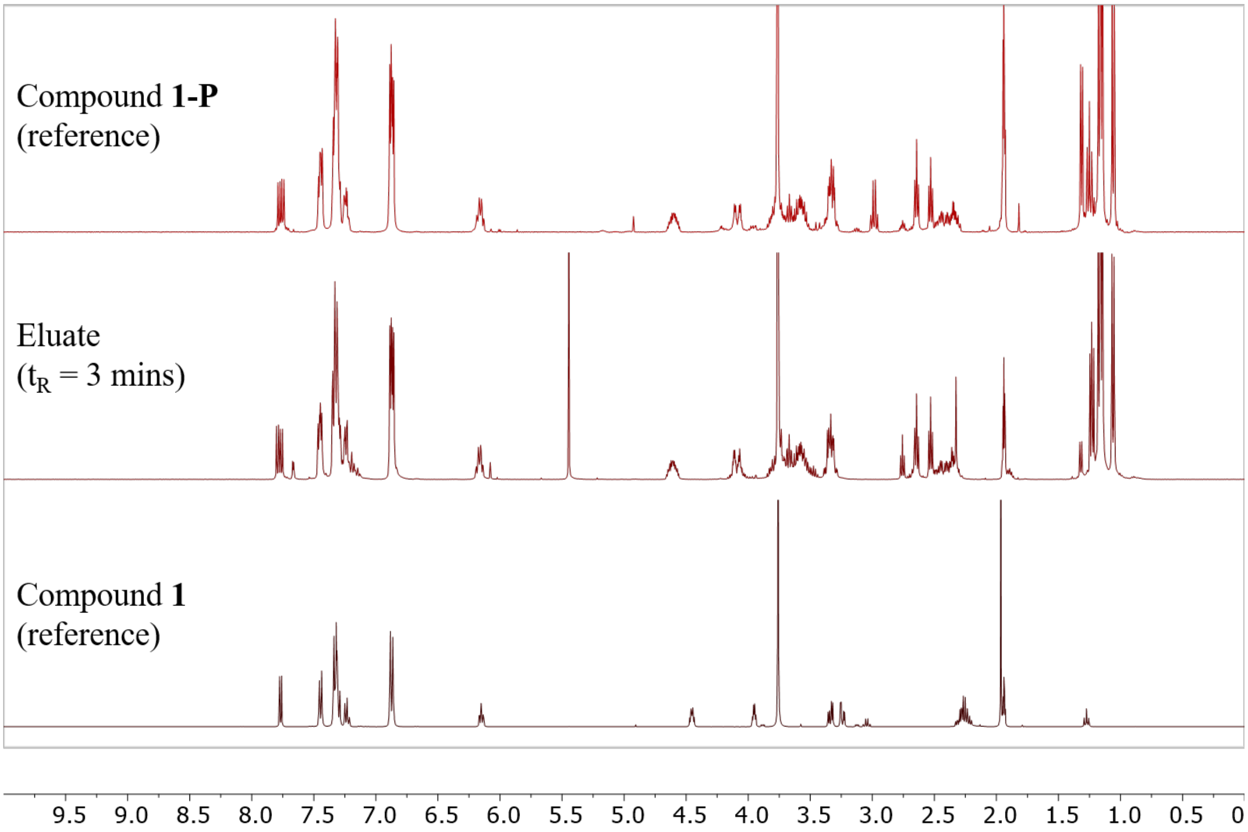

Compound **1**, ^{31}P NMR (CD_3CN), eluate

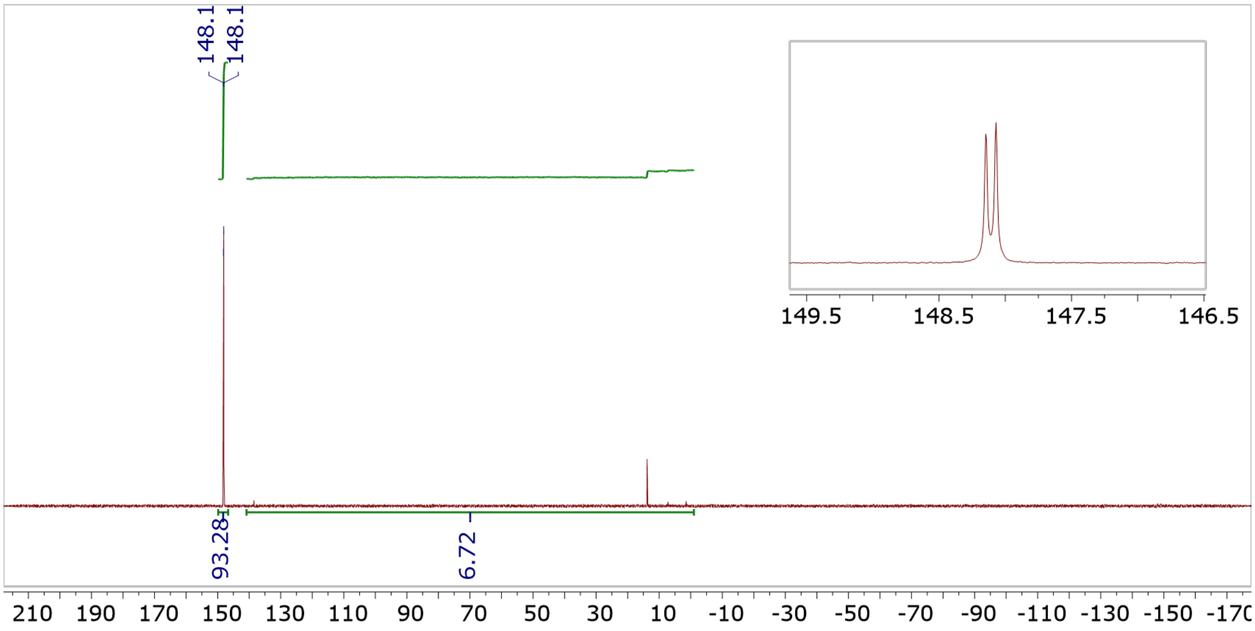

Compound **2**, ^{1}H NMR (CD_3CN)

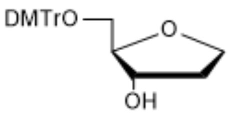

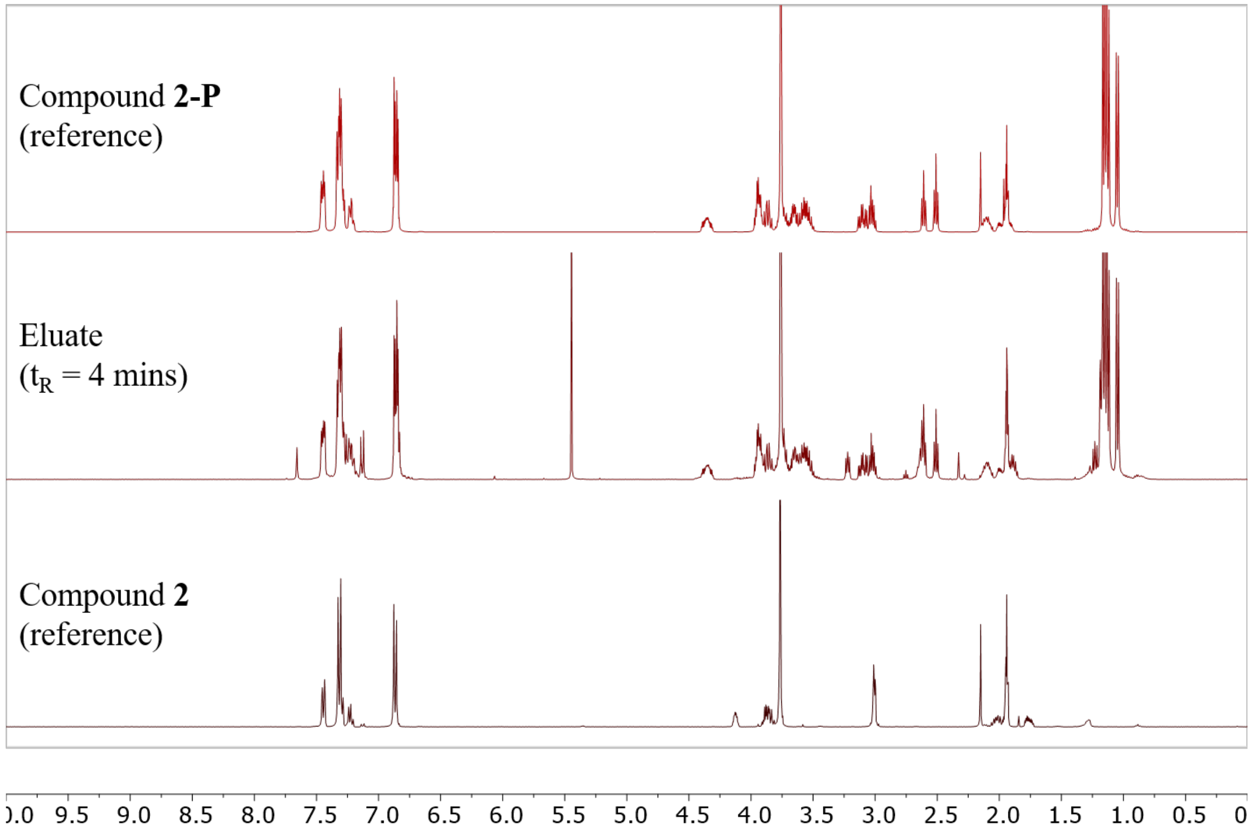

Compound **2**, ^{31}P NMR (CD_3CN), eluate

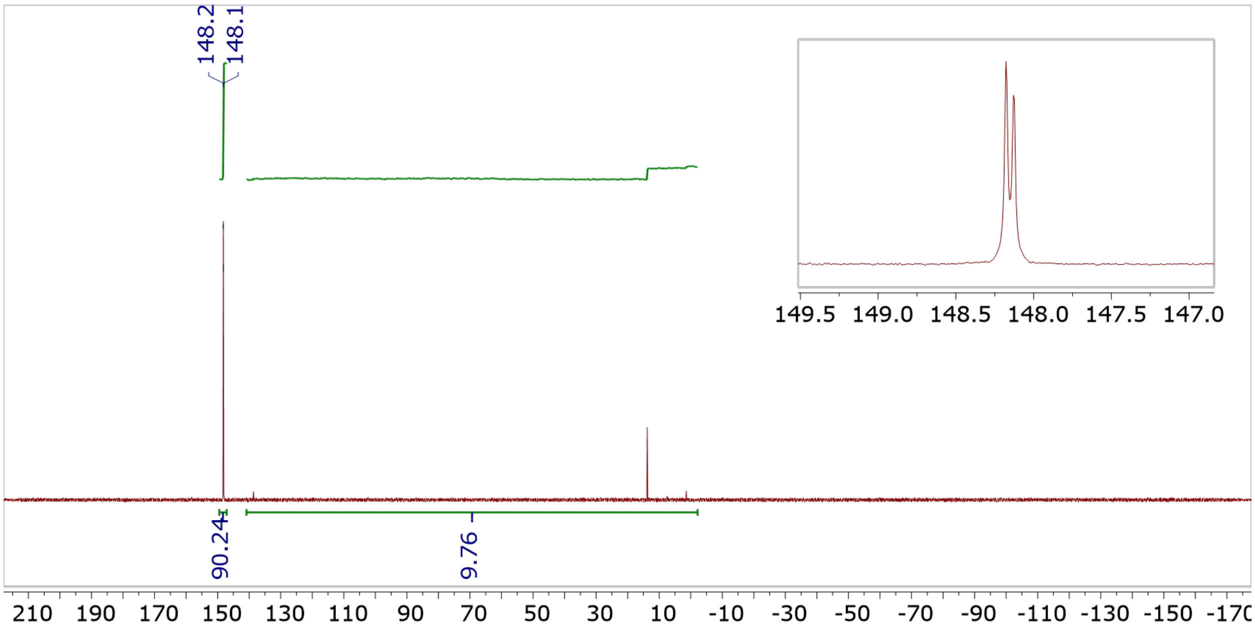

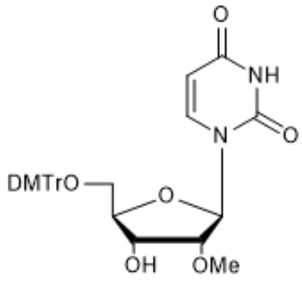

Compound **3**, ^{1}H NMR (CD_3CN)

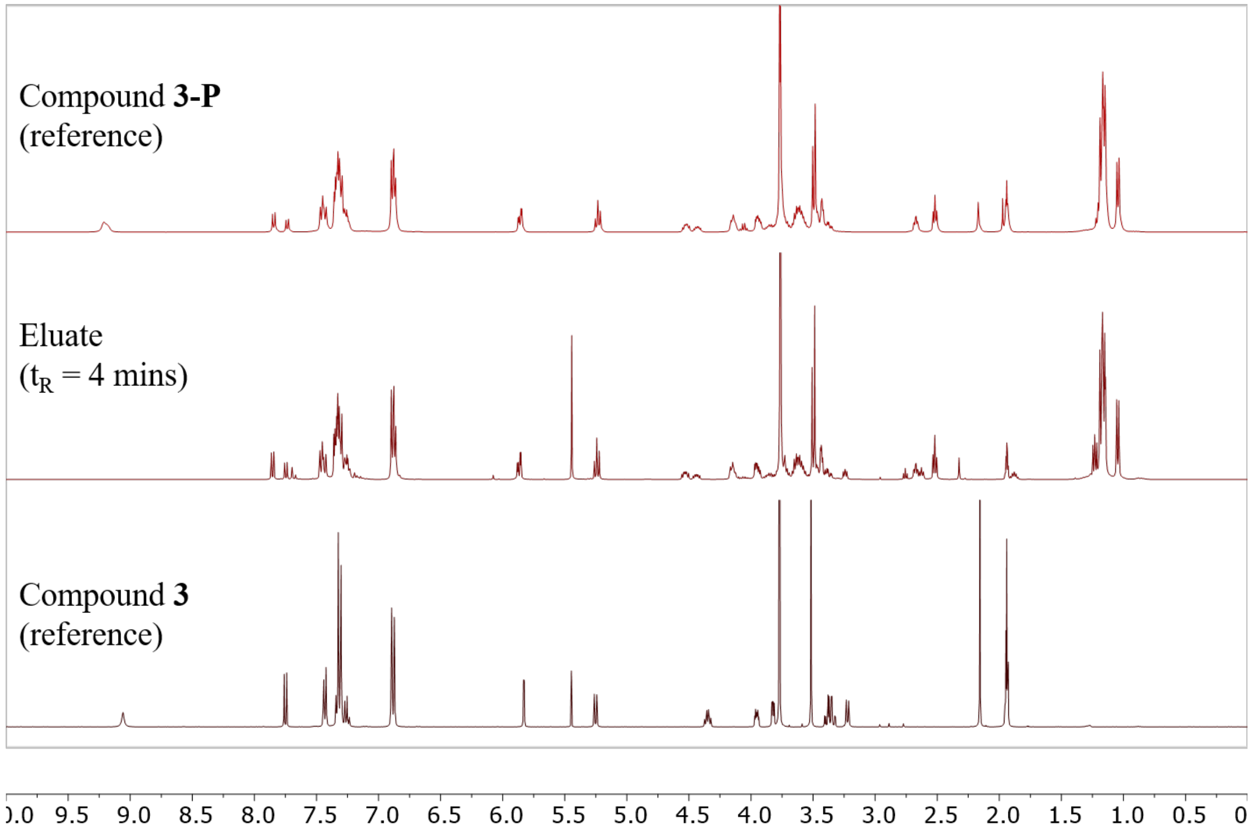

Compound **3**, ^{31}P NMR (CD_3CN), eluate

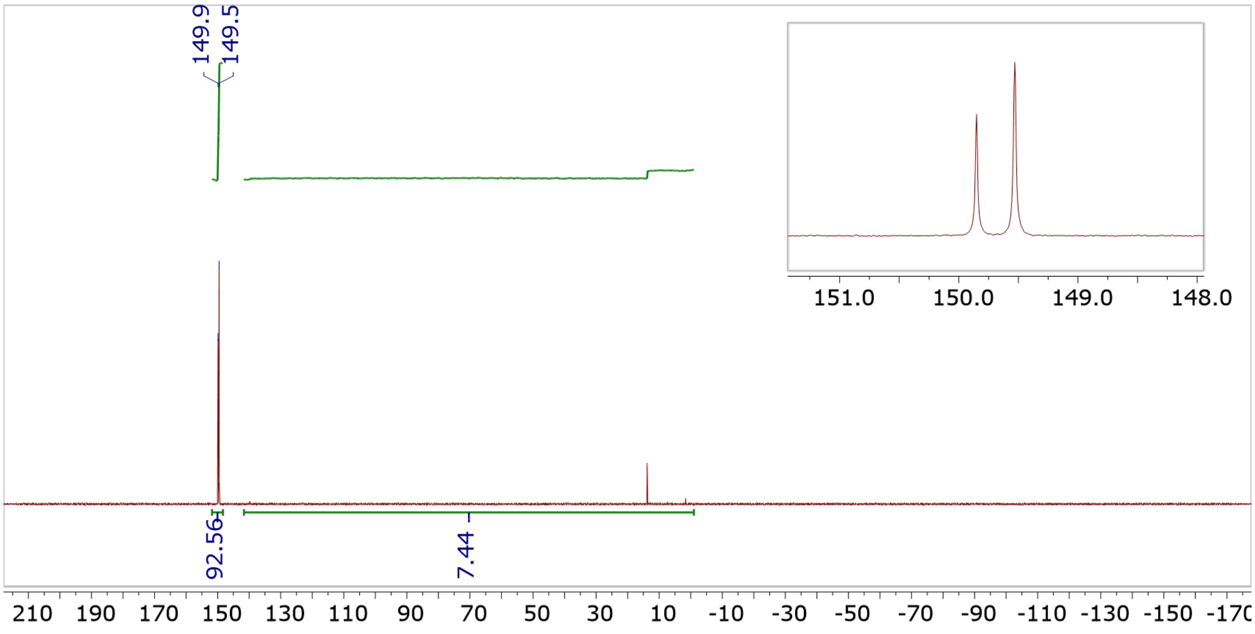

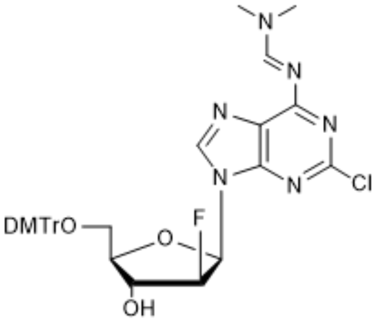

Compound **4**, ^{1}H NMR (CD_3CN)

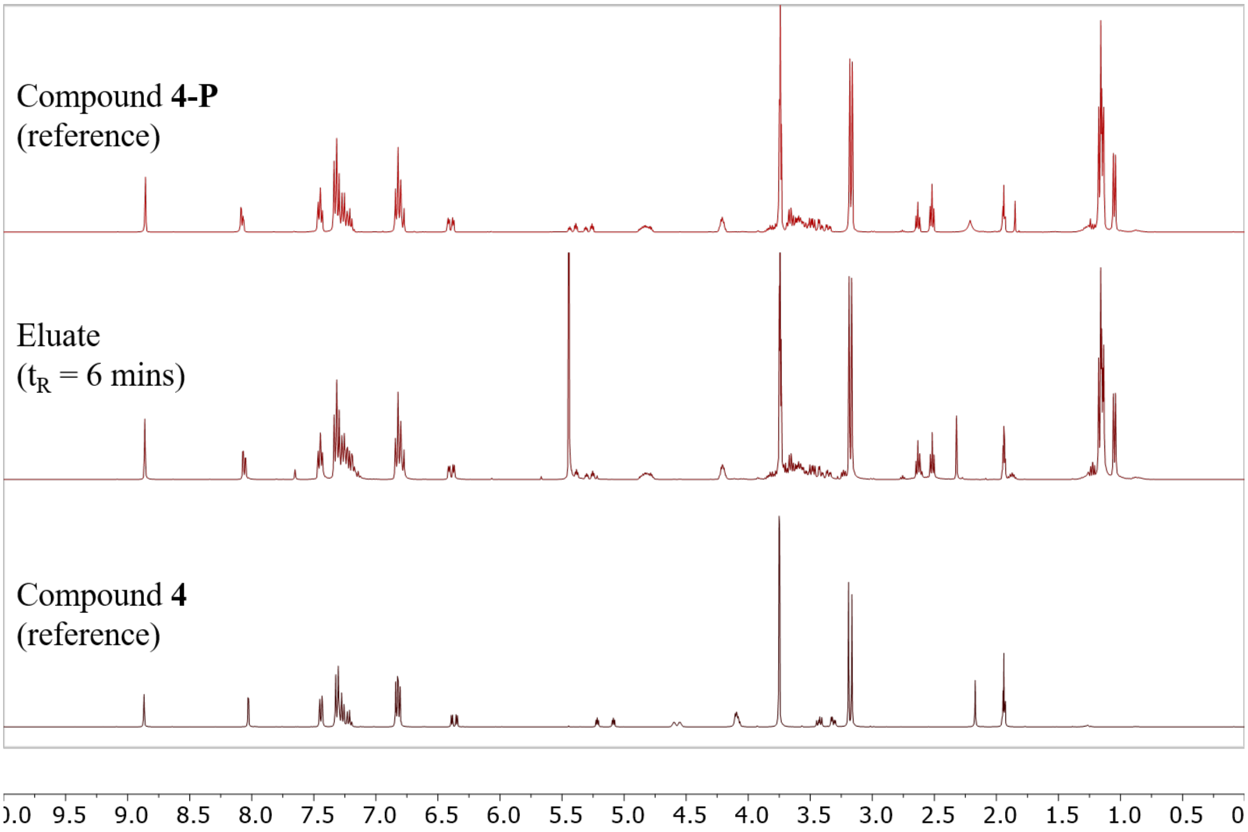

Compound **4**, ^{31}P NMR (CD_3CN), eluate

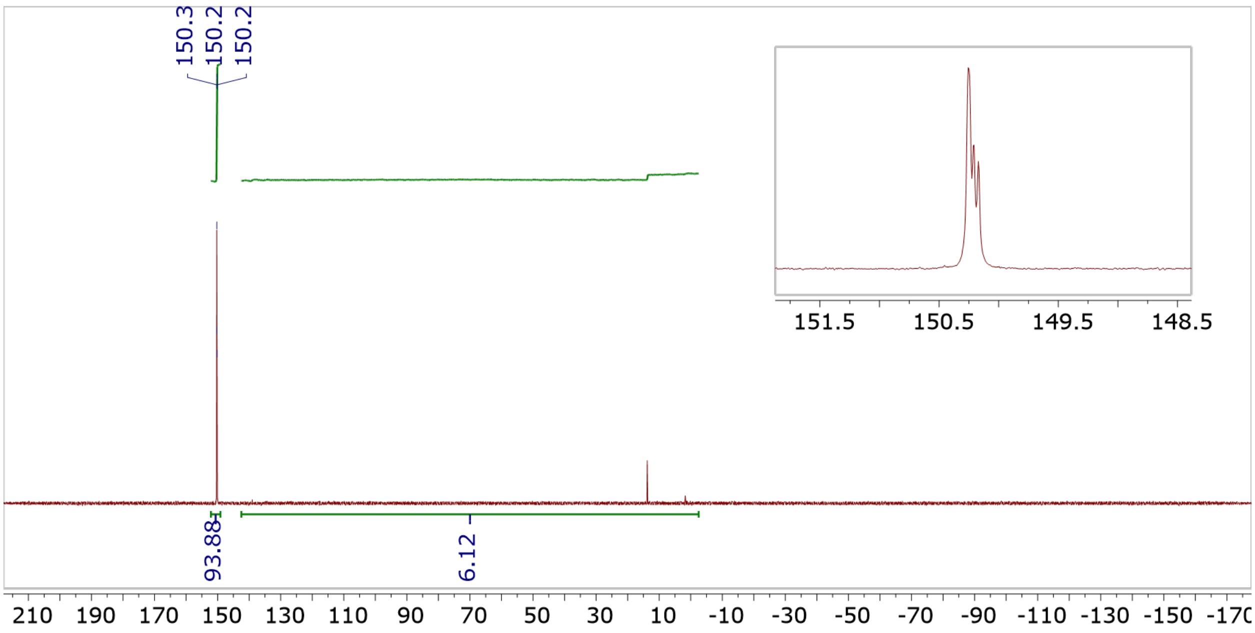

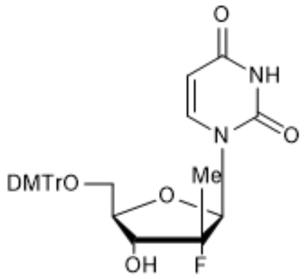

Compound **5**, ^{1}H NMR (CD_3CN)

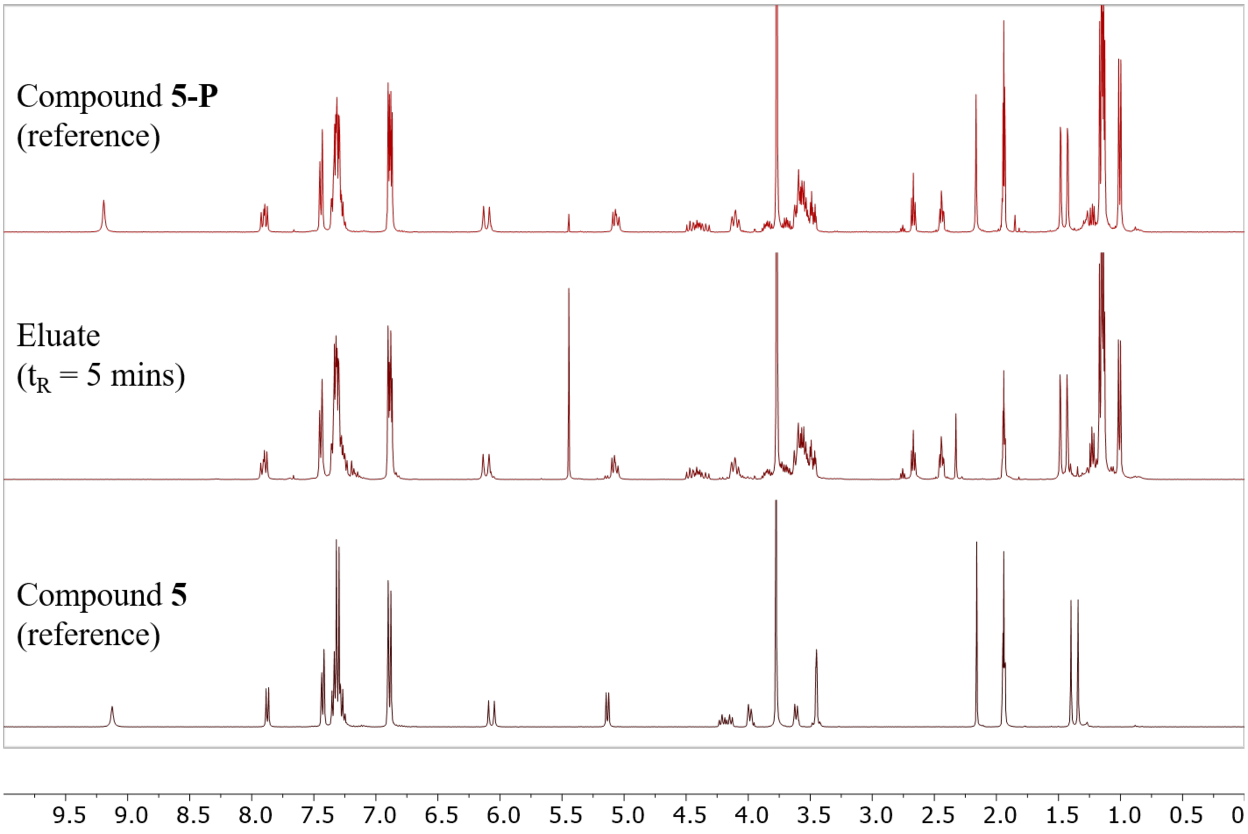

Compound **5**, ^{31}P NMR (CD_3CN), eluate

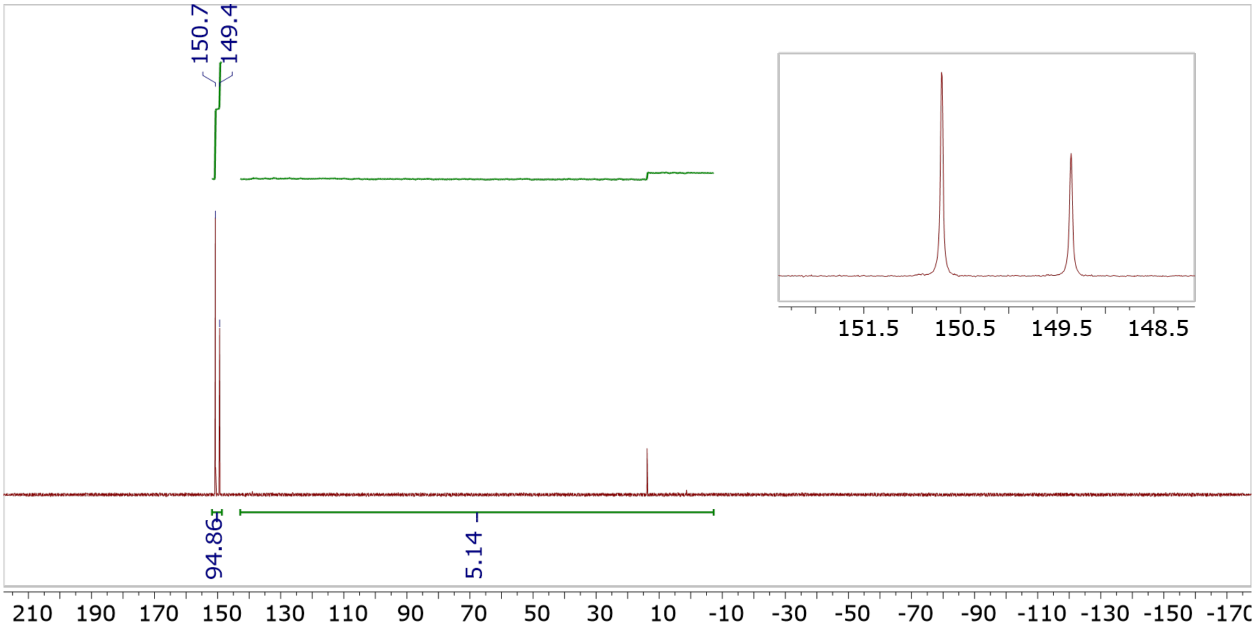

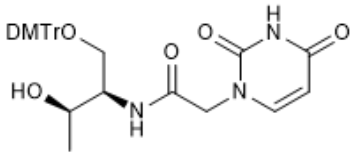

Compound **6**, ^{1}H NMR (CD_3CN)

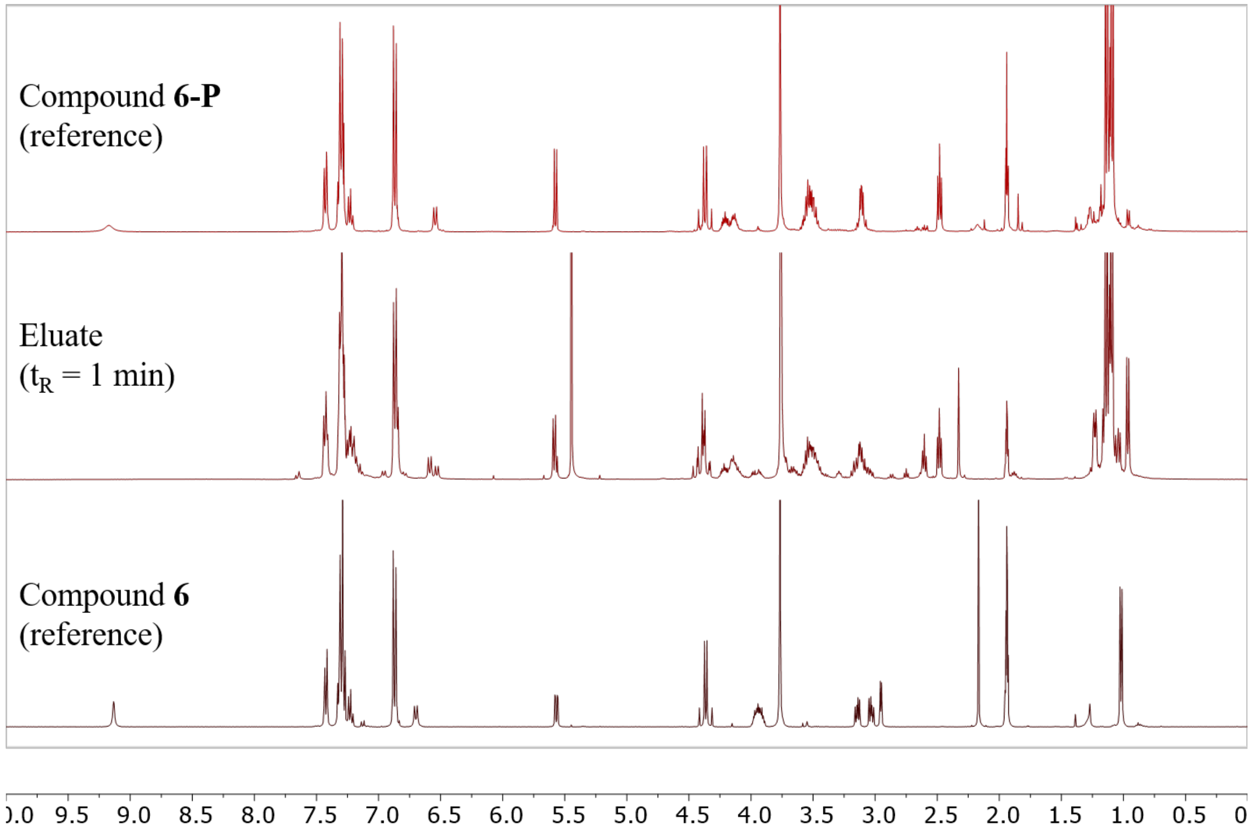

Compound **6**, ^{31}P NMR (CD_3CN), eluate

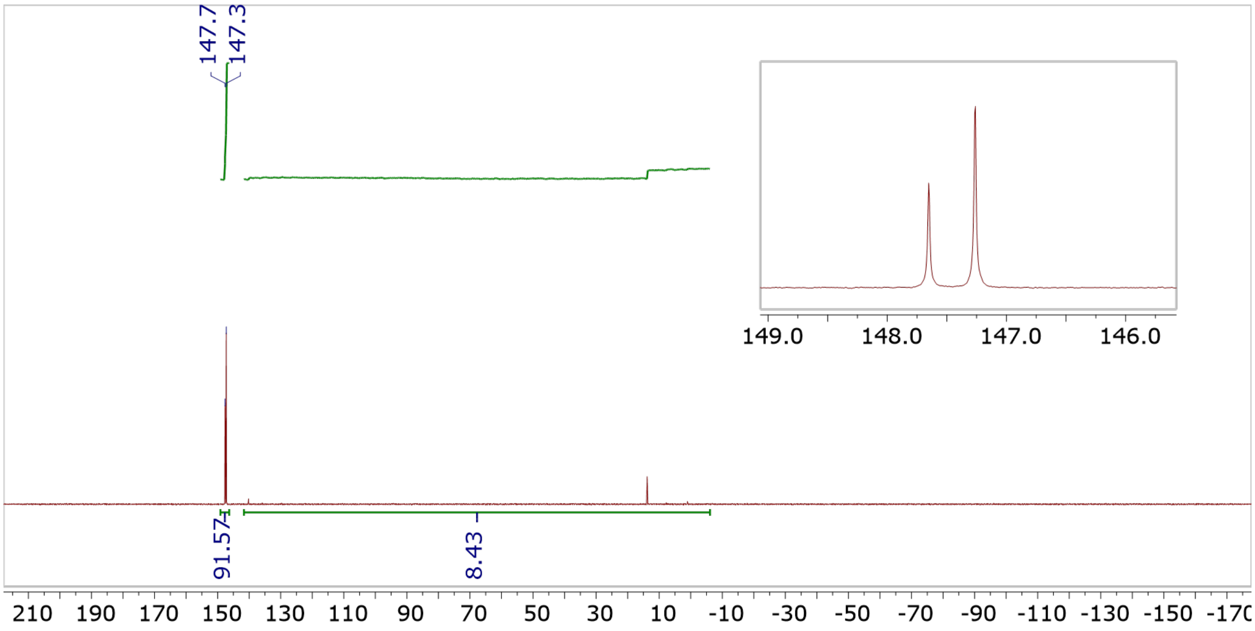

Compound **7**, ^{1}H NMR (CD_3CN)

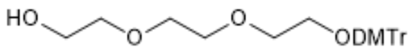

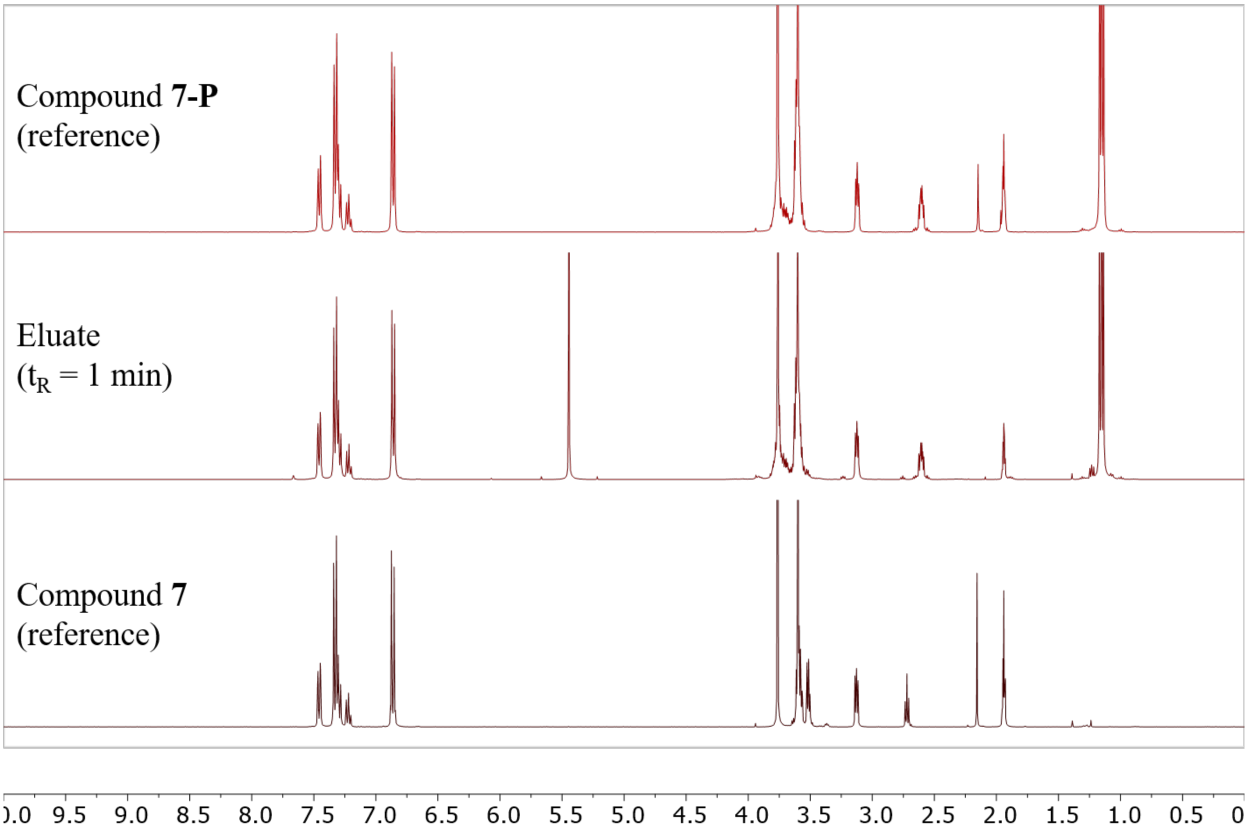

Compound **7**, ^{31}P NMR (CD_3CN), eluate

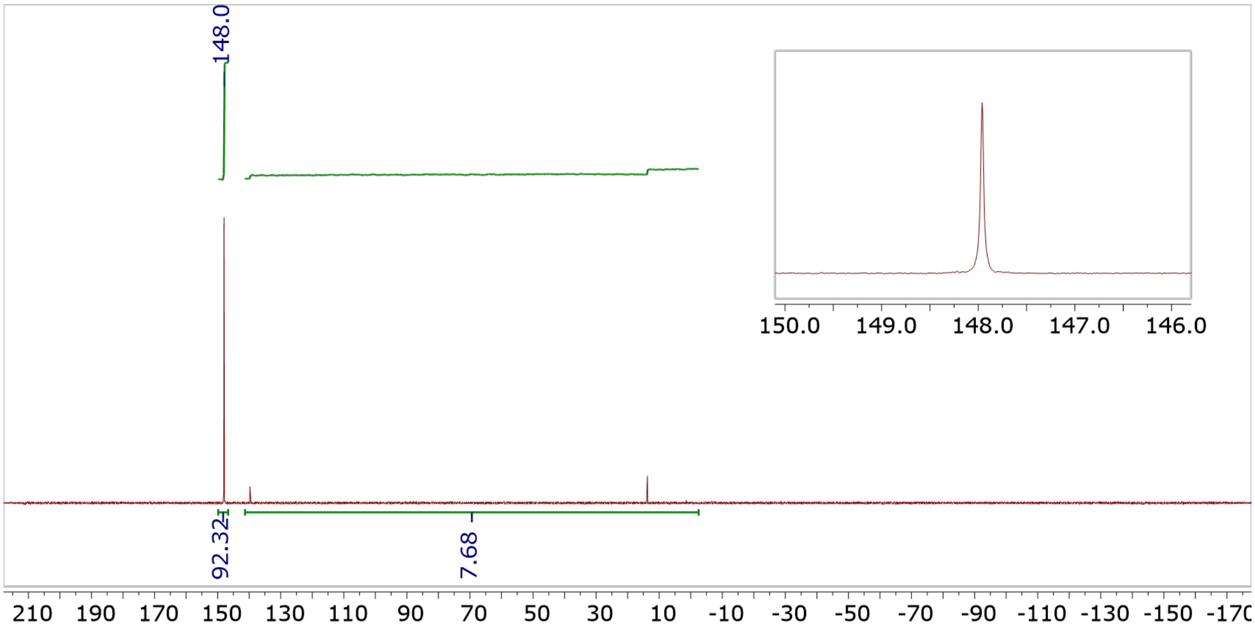

Compound **8**, ^{1}H NMR (CD_3CN)

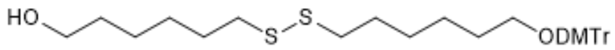

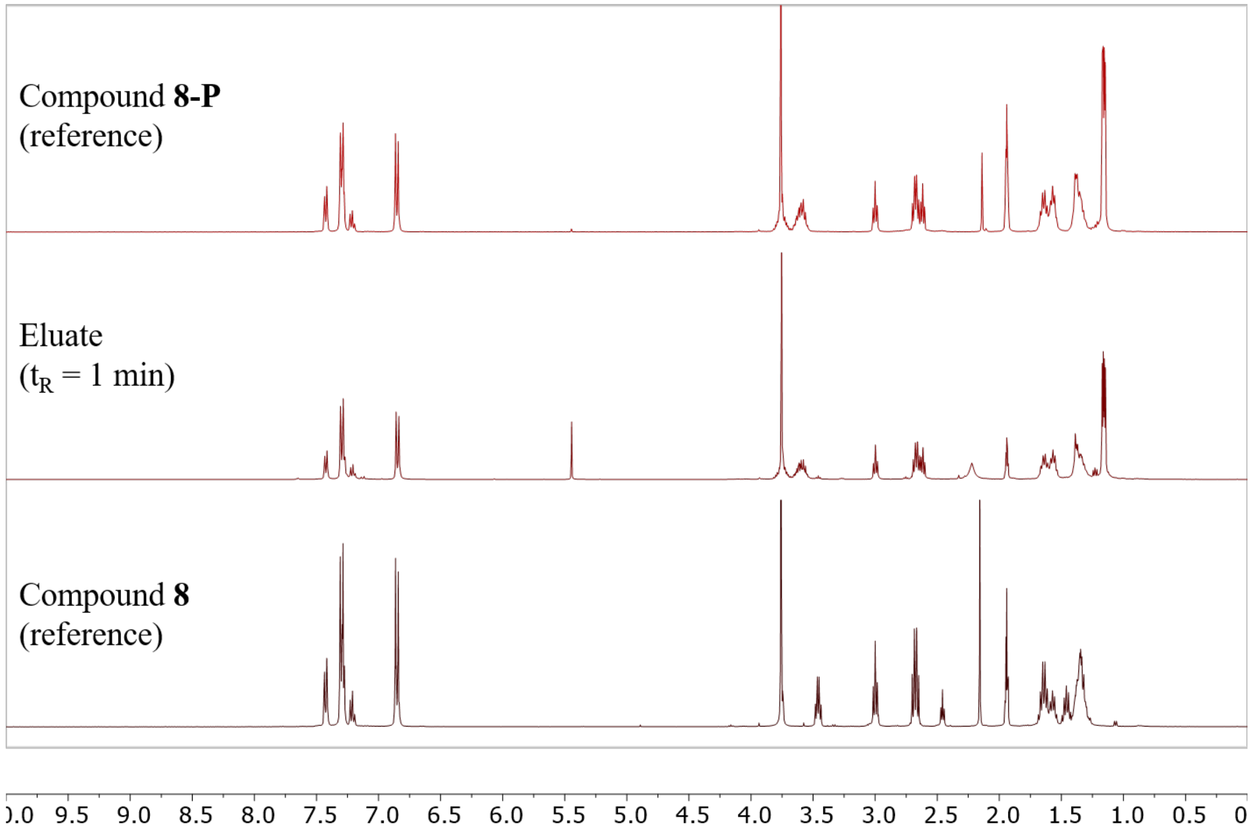

Compound **8**, ^{31}P NMR (CD_3CN), eluate

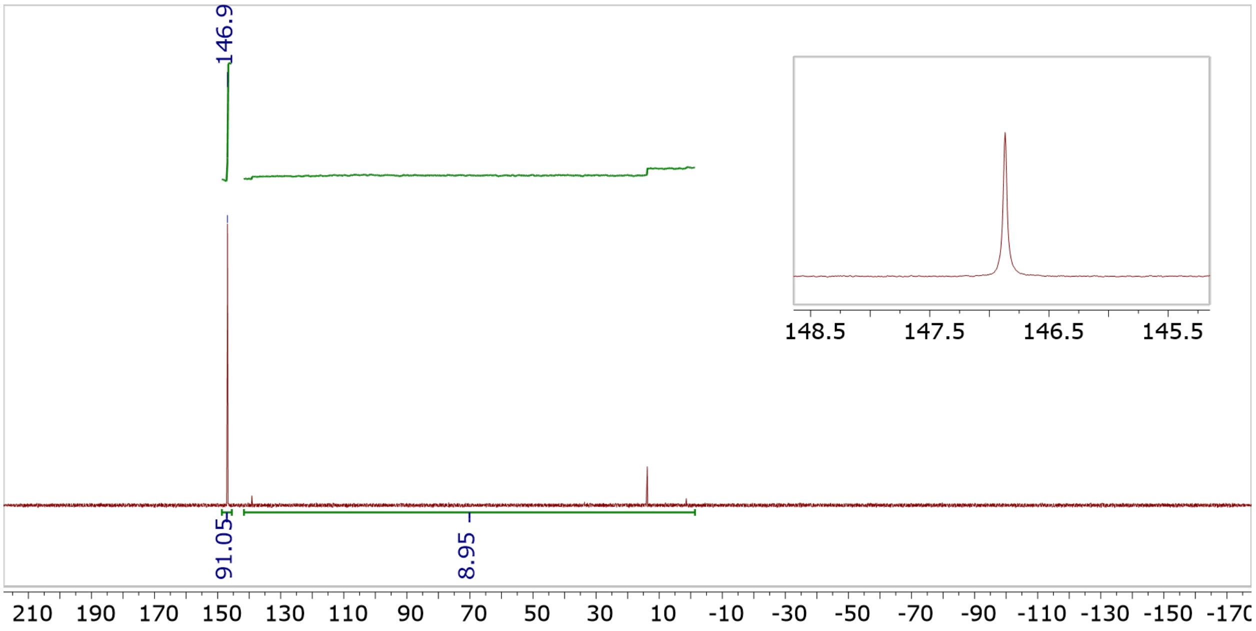

Compound **9**, ^{1}H NMR (CD_3CN)

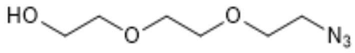

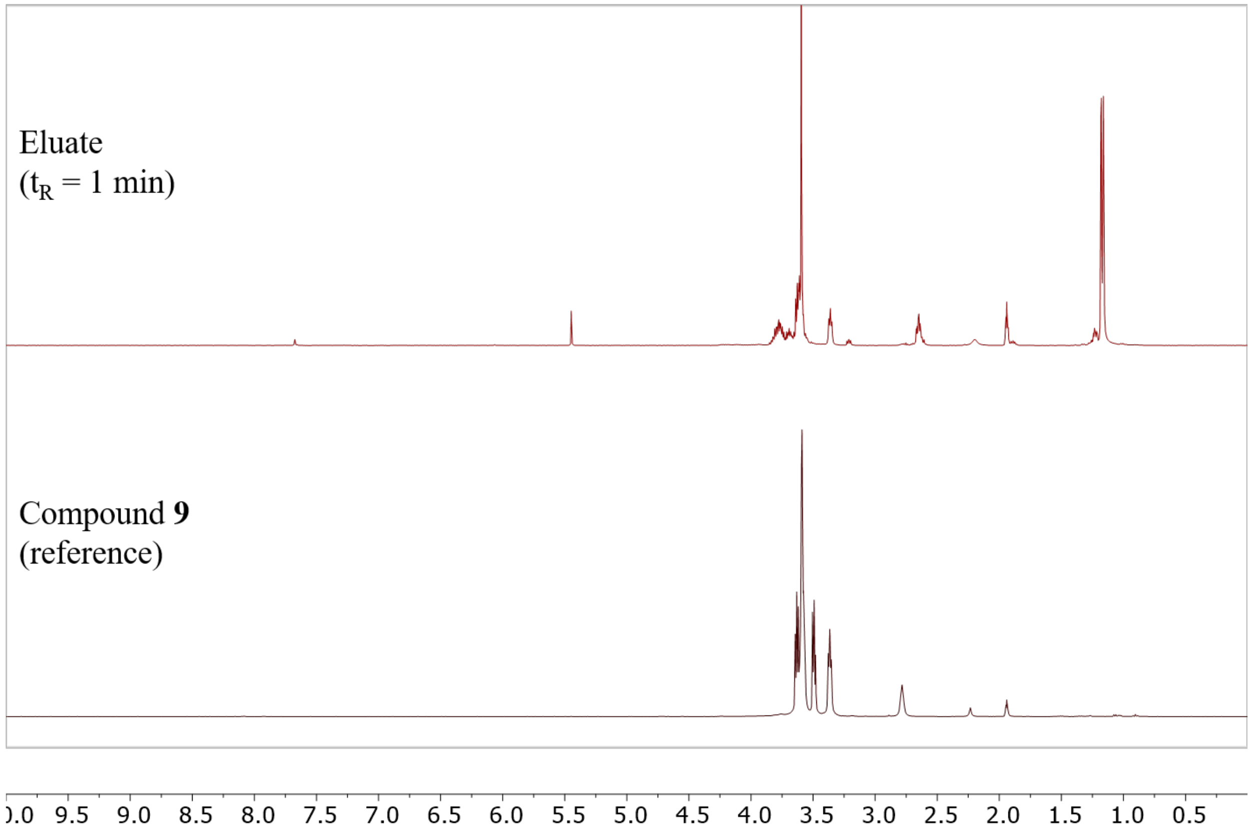

Compound **9**, ^{31}P NMR (CD_3CN), eluate

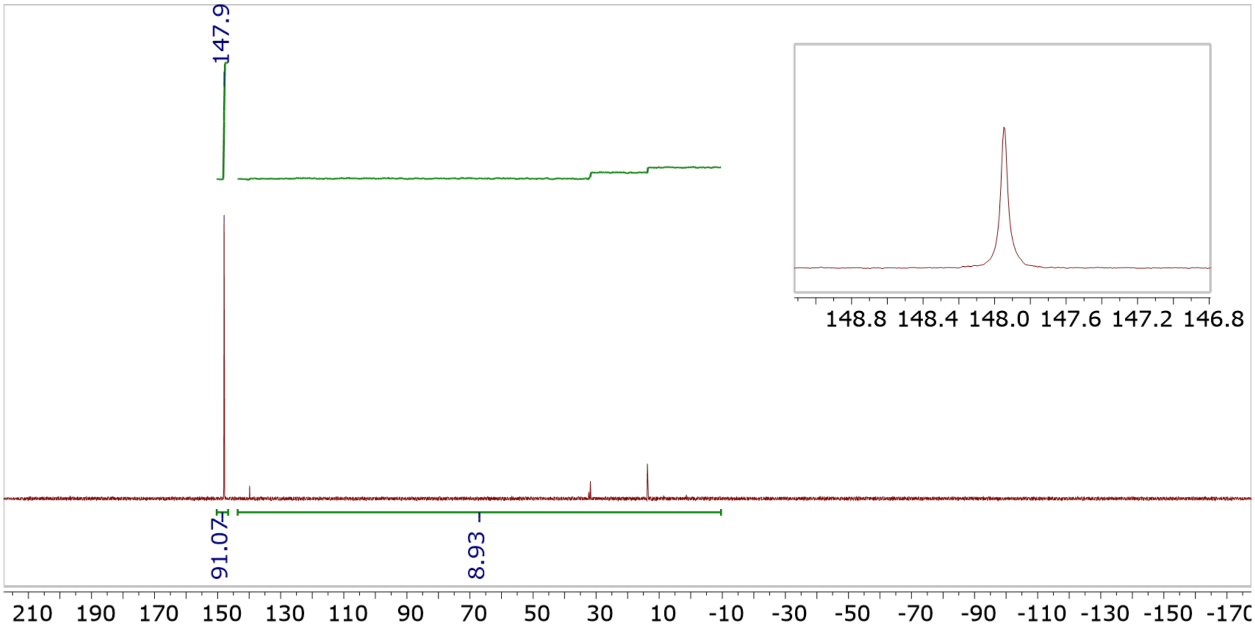

Supplementary References

1. Thomson, C. G. et al. Development of autotaxin inhibitors: A series of zinc binding triazoles, *Bioorg. Med. Chem. Lett.,* **28**, 2279-2284 (2018).

2. Chernyshev, V. M., Chernysheva, A. V., Taranushich, V. A. Optimization of the Synthesis of 5-Amino-1,2,4-triazol-3-ylacetic Acid and Bis(5-amino-1,2,4-triazol-3-yl)methane*, Russ. J. Appl. Chem.,* **82**, 276-281 (2009).

3. Thottempudi, V., Shreeve, J. M. Synthesis and Promising Properties of a New Family of High-Density Energetic Salts of 5-Nitro-3-trinitromethyl-1H-1,2,4-triazole and 5,5′-Bis(trinitromethyl)-3,3′-azo-1H-1,2,4-triazole, *J. Am. Chem. Soc.*, **133**, 19982-19992 (2011).

4. Nguyen, T. J. D., Manuguerra, I., Kumar, V., Gothelf, K. V. Toehold-Mediated Strand Displacement in a Triplex Forming Nucleic Acid Clamp for Reversible Regulation of Polymerase Activity and Protein Expression, *Chem. Eur. J.*, **25**, 12303-12307 (2019).
